# Materials information extraction via automatically generated corpus

**DOI:** 10.1038/s41597-022-01492-2

**Published:** 2022-07-13

**Authors:** Rongen Yan, Xue Jiang, Weiren Wang, Depeng Dang, Yanjing Su

**Affiliations:** 1 School of Artificial Intelligence, Beijing Normal University, Beijing, 100875 China; 2 Beijing Advanced Innovation Center for Materials Genome Engineering, Institute for Advanced Materials and Technology, University of Science and Technology Beijing, Beijing, 100083 China; 3 Collaborative Innovation Center of Steel Technology, University of Science and Technology Beijing, Beijing, 100083 China

**Keywords:** Metals and alloys, Computer science

## Abstract

Information Extraction (IE) in Natural Language Processing (NLP) aims to extract structured information from unstructured text to assist a computer in understanding natural language. Machine learning-based IE methods bring more intelligence and possibilities but require an extensive and accurate labeled corpus. In the materials science domain, giving reliable labels is a laborious task that requires the efforts of many professionals. To reduce manual intervention and automatically generate materials corpus during IE, in this work, we propose a semi-supervised IE framework for materials via automatically generated corpus. Taking the superalloy data extraction in our previous work as an example, the proposed framework using Snorkel automatically labels the corpus containing property values. Then Ordered Neurons-Long Short-Term Memory (ON-LSTM) network is adopted to train an information extraction model on the generated corpus. The experimental results show that the F1-score of *γ*’ solvus temperature, density and solidus temperature of superalloys are 83.90%, 94.02%, 89.27%, respectively. Furthermore, we conduct similar experiments on other materials, the experimental results show that the proposed framework is universal in the field of materials.

## Introduction

Natural Language Processing (NLP) focuses on a computer understanding text knowledge so that a computer can analyze and process natural language^1^. Information Extraction (IE) in NLP is one of the most prominent text mining technologies and aims to extract structured information from unstructured text^2^. Scientific literature in the field of materials contains a large number of reliable data, which promotes data-driven materials research and development^3–5^. It is time-consuming to rely solely on human manual extraction^6^. So, automatic data extraction of organic and inorganic chemical substances from articles in the fields of chemistry and materials science have made their sense using NLP techniques^7–11^.

With the development of machine learning and NLP, IE technology has developed rapidly^6^, particularly in biology and medicine. Sunil *et al*. proposed that IE is a process of detecting and classifying semantic relations and used a Convolutional Neural Network (CNN) to obtain semantic features to extract the information in the biomedical domain^12^. Many papers have applied deep learning models for feature optimization; for example, Xinbo *et al*. used Conditional Random Fields (CRFs) to classify the features of the context and used autoencoders and sparsity limitations to solve the problem of word sparsity^13^. Recently, other IE systems have also been investigated in the search for possible information with Long Short-Term Memory (LSTM). Raghavendra *et al*. embedded words into bidirectional LSTM and CRF. They used a recurrent neural network to obtain features and completed clinical concept extraction^14^. Arshad *et al*. presented an LSTM method for understanding language grammar and deducing the relationship between words^15^. However, all of the above neural networks require an extensive and accurate labeled corpus to train the network.

Unfortunately, there are relatively few papers on many materials subjects, such as superalloys, extracting the required information from the paper becomes a tricky job. In our previous work^11^, we developed an NLP pipeline to capture both chemical composition and property data from superalloys scientific literature. A rule-based Named Entity Recognition (NER) method and a distance-based heuristic multiple-relation extraction algorithm for the pipeline were proposed to overcome the drawback of limited training corpus labels, and achieve high precision and recall simultaneously. The proposed IE algorithm is a rule-based method, while the machine learning method was abandoned after comparison because the labeled corpus was not enough for training. It is a laborious task that requires the efforts of many professionals if completed by humans alone. Rule-based strategy is efficient under such conditions but without the ability to learn and update independently. Therefore, automatically generating corpus in the material domain, enabling to reduce manual intervention, is necessary for machine learning-based IE, which will make it a reality for computers to read papers and extract datasets by themselves.

Two problems are inevitable when faced with machine learning problems: data and algorithms. With the improvement of various machine learning frameworks, the application threshold of algorithms is gradually decreasing. However, the acquisition of data is still a labor-intensive and necessary process. At work, we usually face the following problem: the task has a lot of corpus, but none of them have reliable labels. In response to the above problems, the usual methods are unsupervised learning of transferable features, the combination of rule system and model or simple stacking rule system, semi-supervised methods to expand label data, increase manual verification and annotation^16^. But these methods are either too cumbersome to operate, too expensive, or too inflexible. Based on this, Snorkel^16^ as a data programming framework that enables fast dataset construction and model training has been proposed by a research team at Stanford University.

In this work, we propose a semi-supervised IE framework for materials domain via automatically generated corpus. Taking the superalloy data extraction in the previous work as an example, the proposed framework using Snorkel^17^ automatically labels the corpus containing the name of a superalloy and its corresponding property values. We first put the labeling function written according to the sentence characteristics of scientific literature into the Snorkel function training process and then obtain the accurate training set. Semi-supervised is embodied in human-written labeling functions rather than augmenting the data. Finally, we use the popular Ordered Neurons-LSTM (ON-LSTM)^18^ network to train an information extraction model on this automated training corpus and extract property values in the science literature of materials. We obtain about 18% higher results using ON-LSTM than traditional LSTM on the information extraction task. The code is available at https://github.com/MGEdata/auto-generate-corpus. Our contributions are summarized as follows:

- New IE framework is proposed for materials using the semi-supervised method in machine learning to automatically generate corpus. These works are completed based on the previous work^11^, and further extract the information in the material field.
- ON-LSTM is used to complete the task of IE. To the best of our knowledge, this is the first time that ON-LSTM and IE are combined to explore the possibility of potential integration.
- Experimental results show that the method proposed in this paper can effectively extract information and be applied to broad materials subjects.

## Results

### Information extraction workflow

**Fig. 1:**
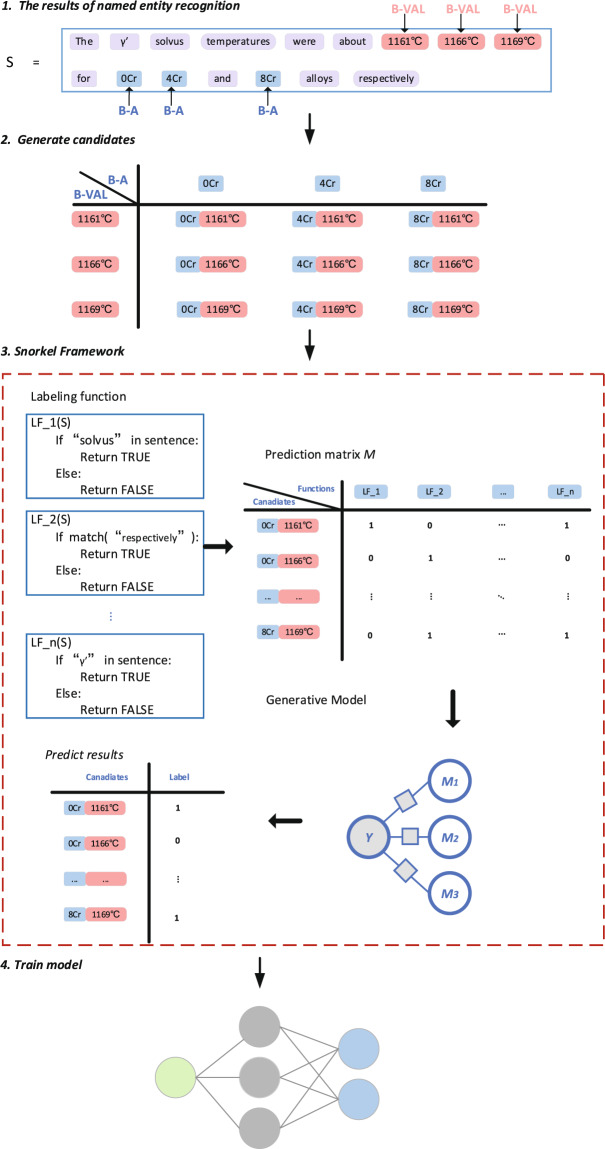
Process for information extraction. Among them, B-A represents the name of the superalloy, and B-Val represents the property value. *LF*_1, *LF*_2, …, *LF*_*n* represent the name of labeling functions.

Our method of extracting material information by automatically generating corpus involves the following steps: NER, generating candidate sets, Snorkel framework and training model, as shown in Fig. 1. In order to explain the algorithm workflow in more detail and more vividly, we take *γ*’ solvus temperature of superalloy as an example. The initial corpus we use is to use the NER method to mark the superalloy name and property value in a sentence. The specific method of NER is detailed in our previous article^11^. However, the initial corpus marks all the superalloy names and property values in a sentence, depending on NER can not accurately find the matching mode of superalloy names and property values if there are multiple superalloy names and property values in a sentence. The next step is to generate candidates. The following is a sample sentence describing the *γ*’ solvus temperature of superalloys:

*The* γ*’ solvus temperatures of X*_1_*, X*_2_ *and X*_3_ *are Y*_1_*, Y*_2_ *and Y*_3_*, respectively*.

This sentence involves three superalloys and their *γ*’ solvus temperatures. In this sentence, *X*_*i*_ represents the i-th superalloy, and *Y*_*i*_ represents the value of the i-th *γ*’ solvus temperature. In this example, the task that we need to complete is to find their correct pairing: (*X*_1_, *Y*_1_), (*X*_2_, *Y*_2_) and (${X}_{3}$, ${Y}_{3}$). We define the candidates as an exhaustive combination of the names of superalloys ${X}_{1}$, ${X}_{2}$, ${X}_{3}$ and *γ*’ solvus temperatures ${Y}_{1}$, ${Y}_{2}$, ${Y}_{3}$. Therefore, there are 9 candidates: (${X}_{1}$, ${Y}_{1}$), (${X}_{1}$, ${Y}_{2}$), (${X}_{1}$, ${Y}_{3}$), (${X}_{2}$, ${Y}_{1}$), (${X}_{2}$, ${Y}_{2}$), (${X}_{2}$, ${Y}_{3}$), (${X}_{3}$, ${Y}_{1}$), (${X}_{3}$, ${Y}_{2}$), (${X}_{3}$, ${Y}_{3}$). If there are $m$ names of superalloy and $n$ *γ*’ solvus temperatures in a sentence, m*n candidates will be generated.

In the third step, we write some labeling functions in the Snorkel framework, a semi-supervised method to screen the candidates, and get the correct pairing of the superalloy name and the *γ*’ solvus temperature. So far, we have accurately found the relationship to be extracted and generated the corpus we need. Finally, we use the deep learning model ON-LSTM training model in these corpora, so that the new corpora directly extract the required relationship by using the training model.

### Automatic generation of training corpus

#### Generation principle

No public corpora of IE can be leveraged due to the few literature in the field of superalloy. Therefore, to train a model in this field, the training corpora problem can be solved through manual search^19^. Snorkel proposes the radical idea that mathematical and systematic structure can be provided for the messy and often entirely manual process of training data creation and management, starting by empowering users to label, build and manage training corpora programmatically.

The third part of Fig. 1 shows the specific process of Snorkel framework. The main advantage of the Snorkel framework is that there is no need to label the dataset manually. When the task changes, the data may need to be relabeled, expanded, or ignored^20^. Users only need to pay attention to the characteristics of each dataset and write labeling functions for the dataset that can automatically determine true and false for candidates. However, Snorkel only proposes a framework for generating the training data and is not designed for a specific field; in previous work^20^, used Snorkel in the field of chemistry. In this work, we develop an application of Snorkel that is a weakly supervised learning framework for generating corpora from the scientific literature.

To generate candidates, we use rules to label all relevant words about superalloys and *γ*’ solvus temperature from the scientific literature. We exhaust all combinations of the marked superalloys and *γ*’ solvus temperature to form candidate sets and then judge them through labeling functions. The generative model in Snorkel calculates the accuracy and relevance of the candidate sets based on the consistency and divergence of the written labeling functions. Based on the labeling functions, the generative model does not require actual data and directly judges whether the candidate is right or wrong. Each candidate will be evaluated by all labeling functions to obtain a reasonable result. Candidates are judged correctly, forming the target corpora.

#### Data source

For superalloys in materials, we use rule-based methods to classify sentences containing the name of the superalloys and corresponding property values from more than 14,425 full texts of scientific journal articles related to material. Similar to our previous work^11^, these articles are accessed through Elsevier Research Products APIs allowing anyone that can obtain an API Key and use the APIs for non-commercial purposes free of charge. The detailed information about Elsevier Research Products APIs can refer https://dev.elsevier.com. After the application is approved, the website will assign an API Key to each user. Through the API Key, we can obtain articles in plain text and XML formats. Once we have the articles, we can perform text mining on the articles. Additionally, we uploaded the dois of 14,425 articles in the supplementary material. The extracted superalloys include two types, Co-based and Ni-based superalloys that account for more than 80% of all superalloys. Sentences containing the property values of superalloys are generally included in the full text, so that we consider the full text of science journal articles. The article about superalloys includes many properties, we focus on three of them: *γ*’ solvus temperature, solidus temperature and density. Among them, 457 sentences related to the *γ*’ solvus temperature. The initial corpus has been published on https://github.com/MGEdata/snorkel. Although only relatively few sentences are obtained, the number of sentences is already quite high for the field of superalloys. In some cases, multiple names and property values are mentioned in one sentence. To accurately match the superalloy and the *γ*’ solvus temperatures, all of the combinations were exhaustively generated to obtain 1,184 pairs. The matched candidate is marked by Snorkel to form corpora. The corpora obtained in this manner reflects the influence of the labeling function on the extraction.

### Effect of labeling functions on candidate

Each dataset has unique characteristics, and labeling functions are customized according to the characteristics of the dataset. If users want to use our proposed framework to extract the relationship in their own corpus, they only need to rewrite labeling functions that match the characteristics of the sentences in their corpus. The labeling functions have nothing to do with the source of the corpus, but only the characteristics of the sentence. The scientific literature on superalloys has a more professional vocabulary. We write more than 10 labeling functions according to their semantic characteristics for extracting *γ*’ solvus temperature. Table 1 provides examples of labeling functions. We adjust the writing of the labeling function according to the coverage, overlaps and conflicts of different labeling functions. The list of labeling functions is shown in Table 2. The coverage of labeling functions refers to the proportion of positive and negative samples that are successfully labeled. At the *γ*’ solvus temperature of the extracted superalloy, the comprehensive coverage of the labeling function we write reaches more than 90%. When users use the framework to write labeling functions, try to make the overall coverage of labeling functions as high as possible. To describe overlaps in a finer-grained way, we illustrate using an example. Suppose there are three candidates $c1$, $c2$, $c3$ and two labeling functions $LF1$, $LF2$. If the labeling function judges the candidate as right, it returns 1, if the candidate is judged as false, it returns 0. If the labeling function does not involve the candidate, it abstains and returns −1. The matrix formed by labeling functions $LF1$ and $LF2$ are [1, −1, 0],[1, −1, −1], respectively. Both $LF1$ and $LF2$ judge the first candidate, which is called overlap. Conflict means that two labeling functions involve the same candidate and the judgment results are inconsistent. The more the conflict tends to 0, the more specific the labeling functions are written. We print out the labeling functions through the labeling function analyzer PandasLFApplier on the official website of the Snorkel framework, and find that the conflict is 0. This indicates that there is no conflict between the labeling functions we write. An examination of the table shows that these labeling functions are comprehensive and precise. These functions have achieved good results. For example, LF_in has a 0.46 coverage of candidates.

**Table 1:** Examples of labeling functions.

Labeling function	Description
LF_temperature_words	If the sentence contains words related to temperature, such as solvus, *γ*’, we label True.
LF_for	If the sentence contains “ ‘*γ*’ solvus temperature’ for ‘superalloy’”, we label True.
LF_oneMatch	If there is only one superalloy and one *γ*’ solvus temperature in a sentence, we label True.
LF_temperature_left	If the *γ*’ solvus temperature is included in the parentheses after the superalloy, we label True.
LF_alloy_twoExpress	If a superalloy has two expressions, we label True.
LF_in	If there is a sentence of “ ‘*γ*’ solvus temperature’ in ‘superalloy’”, we label True.
LF_equal	If there is a keyword “equal” near the superalloy and *γ*’ solvus temperature, we label True.
LF_hasTem	If there is a sentence pattern “has a temperature of”, we label True.
LF_between_and	If the *γ*’ solvus temperature is within a certain interval, we label True.
LF_be	If it is clear what the *γ*’ solvus temperature of the superalloy is, we label True.

‘Description’ is an explanation of what the label function does.

**Table 2:** Coverage, overlaps and conflicts of different labeling functions.

Labeling function	Coverage	Overlaps	Conflicts
LF_temperature_words	0.227451	0.078431	0.0
LF_for	0.027451	0.027451	0.0
LF_oneMatch	0.074510	0.068627	0.0
LF_temperature_left	0.066667	0.064706	0.0
LF_alloy_twoExpress	0.007843	0.007843	0.0
LF_in	0.460784	0.176471	0.0
LF_equal	0.003922	0.003942	0.0
LF_hasTem	0.009804	0.009804	0.0
LF_between_and	0.007843	0.007843	0.0
LF_be	0.033333	0.033333	0.0

### Evaluation for generated corpus

The generative model judges the true or false of each candidate through given labeling functions, thereby transforming the task of generating the corpora into a classification task. It is well-known that the F1-score is a good measure for classification problems, and some classification problems often use the F1-score as the final evaluation metric. F1-score is the harmonic mean of precision and recall, that is, ${\rm{F1}} \mbox{-} {\rm{score}}=2\ast \frac{precision\ast recall}{precision+recall}$. Precision is given by $\frac{TP}{TP+FP}$, and recall is given by $\frac{TP}{TP+FN}$. Here, TP is truly positive, which is judged as a positive sample and in fact is a positive sample. FP is false positive, which is judged to be a positive sample, but in fact is a negative sample. FN is false negative, which is judged to be a negative sample, but in fact is a positive sample. The maximum value of the F1-score is 1, and the minimum value is 0.

In addition to the F1-score, ROC^21^ is also an indicator used to measure the imbalance of classification. In particular, ROC-auc is used to evaluate the pros and cons of a binary classifier. ROC-auc is defined as the area under the ROC curve. The ROC curve is generally on a straight line *y* = *x*, so the value range of all ROC-auc is between 0.5 and 1. In many cases, the ROC curve does not clearly indicate which classifier performs better, and ROC-auc is a numerical value. A larger value corresponds to better classifier effect. For the relationship between the value of ROC-auc and the classifier, we have a rough standard for evaluating the classifier. If ROC-auc is less than 0.5, the model has little discrimination ability. If ROC-auc is greater than 0.5 and less than 0.8, the discrimination ability of the model is acceptable. If the value of ROC-auc is greater than 0.8, the discrimination ability of the model performs better.

We divide the 1184 candidate sets of the *γ*’ solvus temperature into the training set, development set and test set, consisting of 674, 200, and 310 candidate sets, respectively. To verify the effect of using Snorkel to generate the corpora, we invited domain experts to mark the development set and test set manually. Among the 1184 candidate sets, the experts annotate a total of 200 candidate sets as development. Although the manual workload is currently somewhat large, the trained model can generate a larger data set. The manual workload is limited to the early stage and the later use of machine processing will be much faster than manual processing. To date, the training set and test set have not been labeled, and the development set has been manually labeled. We embed the label functions in the Snorkel framework for the development set. The purpose is to extract the correct information from the training set to form the corpora.

**Fig. 2:**
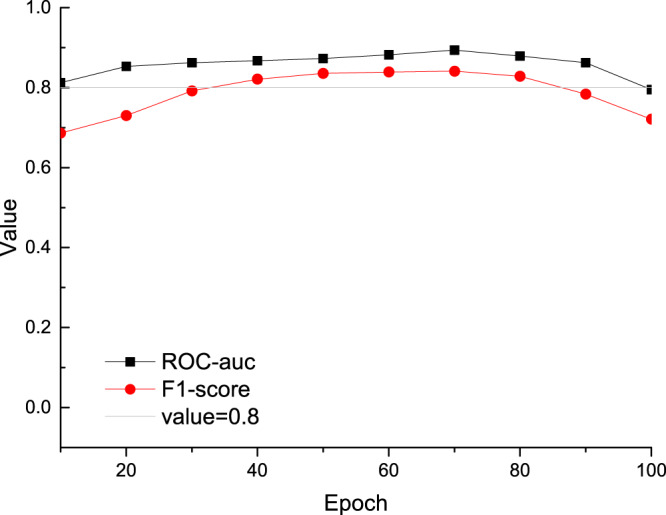
The performance of F1-score and ROC-auc in the generated dataset. If the value is greater than 0.8, the model is working well.

The evaluation results of the automatically generated corpus are shown in Fig. 2. The number at the bottom of the figure is the epoch, and the vertical axis represents the specific value. When using the Snorkel framework, we use different epochs. When the model is trained, the effect of the model will become better as the epoch increases, but if we train too many epochs, the model will overfit the training data and the effect will decrease. Ideally, we want to find the inflection point where the model goes from good to bad to decide whether to stop training. After many experiments, we found that the best results are obtained when the epoch is 70. The best ROC-auc was 0.882, and the best F1-score was 0.839. The corresponding inflection point epoch is 70, and more epochs will cause overfitting, resulting in poorer results. These values indicate that the quality of the generated data set is high. Although these values vary slightly with different epoch, it can be seen from the figure that the difference is not significant. This shows that as long as the label function is written accurately, the snorkel learning ability is not highly correlated with the epoch.

### Validation of the snorkel model

We obtained corpus using Snorkel. When judging whether candidates are right or wrong, we write the label function at the level of the candidate set. Since different candidates may from the same sentence, when verifying on the test set, the sentences in the test set may have been seen by the model during training. To illustrate the generality of our model, we add un-trained 88 sentences about *γ*’ solvus temperature to generate 298 candidate sets.

We put the generated 298 candidate sets directly into the trained model, and judge each candidate. We invite experts to randomly select 50 pieces of corpora automatically generated by Snorkel for manual inspection. Table 3 is an example of the corpora corrected by experts. The correct pairing is selected from a large number of candidates. The results found that using the method of automatically generating corpus tags. The tag accuracy rate reached more than 80%. The first column labeled 1 is the correct pair, and the one labeled 0 is wrong. The ‘name_id’ and ‘attri_id’ respectively represent the position of the superalloy and *γ*’ solvus temperature in a sentence.

**Table 3:** An example of a manually corrected dataset.

correct	name_id	attri_id	sentence	tokens
1	(2:2)	(16:17)	In particular Co-30Ni-12Al-41a-12Cr (12Cr)…	[In,particular,Co-30Ni-12Al-41a-12Cr,(, 12Cr,),…]
1	(4:4)	(16:17)	In particular Co-30Ni-12Al-41a-12Cr (12Cr)…	[In,particular,Co-30Ni-12Al-41a-12Cr,(, 12Cr,),…]
1	(12:12)	(15:16)	Moreover, it is worth noting that the solvus temperature value…	[Moreover,it,is,worth,noting,that,the,solvus,temperature,value,…]
0	(12:12)	(26:27)	Moreover, it is worth noting that the solvus temperature value…	[Moreover,it,is,worth,noting,that,the,solvus,temperature,value,…]
0	(12:12)	(36:36)	Moreover, it is worth noting that the solvus temperature value…	[Moreover,it,is,worth,noting,that,the,solvus,temperature,value,…]

‘name_id’ and ‘attri_id’ represent the position of the name and attribute value in the sentence, respectively.

### Training of relation extraction model

With the large number of labeled corpora produced by the Snorkel, we can use these corpora to train a discriminant model. But we can’t help but wonder why we need to train another discriminant model since the Snorkel can accurately determine the type of the sample? This question needs to start with the difference between the generative and the discriminant model. The generative model in Snorkel learns the joint probability distribution *P*(*X, Y*) from the data, and then obtains the conditional probability distribution *P*(*Y*|*X*) as the predictive model, the formula for generating the model is expressed as follows.

1 $$P(Y| X)=\frac{P(X,Y)}{P(X)}$$

The discriminant model directly learning the conditional probability distribution *P*(*Y*|*X*) from the data is set as a prediction model. Based on the characteriatics of the discriminant and generative models, the corpora produced by the generative model can help the discriminant model improve the coverage of the proposed method. The generative model needs to learn the joint probability distribution *P*(*X, Y*), but for those corpora that cannot be covered by all the labeling functions, it is obviously impossible to get *P*(*X, Y*). On the contrary, the discriminant model only needs the characteristics of *X* itself. *P*(*Y*|*X*) can be calculated, so the discriminant model can cover the data points that the generative model cannot cover. In addition, compared to the probability graph model used in generative model training, discriminant models can be trained with more advanced and complex models, such as the ON-LSTM model we use, which can also improve the accuracy of the model.

**Fig. 3:**
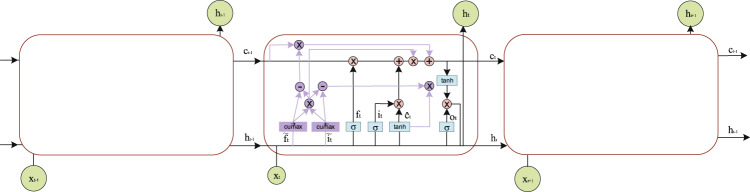
The internal structure of ON-LSTM, where *σ* is the activation function *sigmoid*, *f*_*t*_ is forget gate, *i*_*t*_ is input gate and *o*_*t*_ is output gate.

ON-LSTM integrates the hierarchical structure into the LSTM through specific sorting of neurons, allowing the LSTM to learn the hierarchical structure information automatically. The training method is supervised learning, and the trained model can be used to process a large material corpus. ON-LSTM sorts the neurons inside the LSTM and integrates the hierarchical structure to express richer information^18^. In the original LSTM model, the updates between neurons are not related. For this reason, ON-LSTM adds two gates: the master forget gate $\widetilde{{f}_{t}}$ and the master input gate $\widetilde{{i}_{t}}$. The structure of ON-LSTM is shown in Fig. 3.

**Fig. 4:**
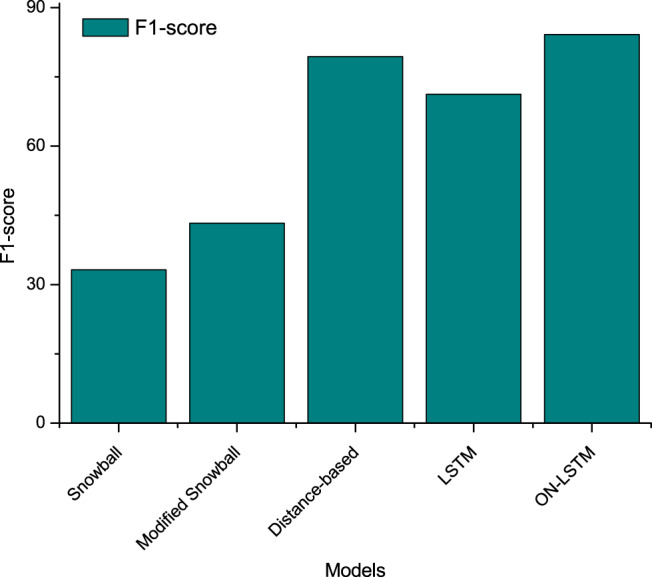
Comparison results of ON-LSTM and the algorithms proposed in previous articles. ON-LSTM is our proposed method.

To demonstrate the superiority of the proposed method, our algorithm is compared with several classical algorithms on our proposed dataset. The comparison results are presented in Fig. 4. Among them, Snowball^22^ is a general information extraction framework. Modified Snowball^23^ is an improvement on the basis of snowball for the material field. Distance-based algorithm is the method proposed in our previous article^11^. LSTM refers to the results obtained after we use Snorke to automatically generate the corpus and then use the LSTM network training. ON-LSTM is the result of training with ON-LSTM after the production corpus. It is obvious that our proposed method performs much better than the previous classical algorithms. The results show that ON-LSTM performs better than LSTM on the IE task. In other words, ordered neurons can express richer information in sentences and capture semantic information between words.

### Generalize validation

The method that we proposed is a general framework for IE without corpora, which is universal in materials. To better illustrate this characteristic, we also extracted other physical properties from the material domain, including density, solidus temperatures of superalloys and hardness information of high entropy alloys. Table 4 shows that F1-score for density, *γ*’ solvus temperature of superalloys and hardness information of high entropy alloys. Experimental results show that our proposed method for relation extraction through automatically generated corpus is versatile and can extract any properties in the material domain.

**Table 4:** F1-score for density, *γ*’ solvus temperature of superalloys and hardness information of high entropy alloys.

Physical properties of materials	number of sentences	Number of candidates	F1-score
density of superalloys	222	846	94.02
solidus temperatures of superalloys	128	348	89.27
hardness of high entropy alloys	155	472	85.81

From Table 4, we can observe that F1-score has good performance in extracting density information from superalloys. We observe the characteristics of sentences containing density and find that these sentences are relatively monotonous compared to other attributes when describing density. This is why the F1-score of the density is relatively high. We summarize several typical sentence patterns as follows, where *A* represents the attribute and *B* represents the property value. *A*_*i*_, *B*_*i*_ represents i-th A or B.

- “More significantly these Co-V-based superalloys have lower density (8.39–8.86 g/cm3)”. When writing a label function, we can describe it in the form of ‘A(B)’.
- “The apparent density of the GTD222 and TiC/GTD222 composite powders were 4.56 g/cm3 and 4.48 g/cm3 respectively”, which can be summarized as the pattern of ‘*A*_1_ and *A*_2_ be verb *B*_1_ and *B*_2_’.
- “While the density of Nimonic 90.0 is 8.2 g/cm3 the layer constituents Ni2Si, Ni5Si2, Cr2B and CrB have density of 7.2 g/cm3 7.0 g/cm3 6.6 g/cm3 and 6.1 g/cm3 respectively.”. Labeling functions can be written as “*A*_1_, *A*_2_, *A*_3_ and *A*_4_ have density of *B*_1_, *B*_2_, *B*_3_, *B*_4_”.

## Discussion

Machine learning methods require large amounts of data for model training. Although machine learning methods have been widely used in many fields, they are still novel methods for extracting the required information in the field of materials. The extracted information can help researchers to determine which materials to use under what circumstances.

In this work, we use semi-supervised Snorkel to generate training sets in the field of materials. We take superalloys as an example, and verify the generality of the proposed method in the field of materials through a number of different material types. When generating the training set, since our dataset is highly unbalanced, even a trivial baseline that always outputs negative can obtain high accuracy. Therefore, we evaluated the dataset using the F1-score and ROC-auc rather than accuracy. In addition, we first investigate the potential integration between ON-LSTM and IE. Although we use more advanced methods to train the model, the results are not particularly satisfactory. This may be due to the small number of datasets and the imbalance of positive and negative samples. Although all our processes extract specific information in the field of materials, the proposed method can also be applied to other fields without datasets. Different labeling functions are written according to the requirements, and then the model is trained according to the generated dataset to increase the robustness of extraction. In all cases, the difficulty of writing labeling functions is related to the corpus’s difficulty and the information extracted.

Using machine learning methods to extract information in the material field still faces many challenges. On the one hand, machine learning requires a large corpus, while the amount of data in the field of superalloys is small due to the difficulty of acquiring accurate and error-free datasets. In the future, we hope to obtain more articles about materials and obtain more sentences containing the physical properties to obtain larger and higher-quality data sets. On the other hand, we do not use a pre-trained model when extracting information due to the limited number of datasets. The pretraining model obtains models that are not related to specific tasks from large-scale data through self-supervised learning methods that can more effectively express the rich semantic features of words or sentences. In the future, it may be possible to introduce pretraining models such as BERT^24^ and XLNet^25,26^ in the information extraction stage to fully take advantage of the context information of sentences and accurately use vectors to express the meaning of words.

## Methods

In this section, we describe the machine learning methods used in this work, namely the Snorkel method for generating datasets and the ON-LSTM method for training the IE models.

### Snorkel

Snorkel is a model that uses weak supervision to generate datasets. It manually labels any outlier data and only requires users to write labeling functions^27^. Snorkel uses data programming^28,29^ to obtain its output. The main purpose of Snorkel is to give an *φ* $\in$ Φ and determine the possible discrete label *τ* $\in$ *T*, where Φ represents the candidate set and *T* represents the set {1, 0}. To achieve this goal, we need to write some labeling functions *λ* based on the specific dataset. For users, the written labeling functions are black-box functions, and they do not need to understand the operation of Snorkel on labeling functions. Upon input of candidate set Φ and labeling functions *λ*, Snorkel outputs labels *T* to which Φ belongs. Users can write labeling functions in the following ways:

1. Pattern-based: The method formulates some rules by observing the characteristics of sentence patterns. Omar *et al*. proposed the basic principles of observation to help users annotate data sets^30^. Sonal *et al*. used the rules of distribution similarity and word-to-word distance for labeling^31^.
2. Distant supervision: Distant supervision refers to an existing knowledge base. Assuming that the knowledge base contains the information to be extracted, it is equivalent to automatically marking a part of the samples; for example, Raphael *et al*. used the information in the knowledge base to extract sentence-level repetitive relationships^32^.
3. Weak classifiers: We call a classifier that is slightly better than a random prediction but not very accurate a weak classifier^33^. We can train weak classifiers on other datasets as labeling functions.

If the candidate set contains *a* data points and the users write *b* labeling functions, then the matrix Γ $\in$ *T*^a*b^ will be generated. Each labeling function may have coverage, overlaps, and conflicts for the same data point. Snorkel automatically solves the above problems internally and finally forms a single label for each data point. The most important component of Snorkel models, integrating multiple labeling functions, is called a generative model. Snorkel implements this component using the method of data programming. For details, please refer to^27–29^.

### LSTM

After the acquired dataset is embedded by the plug-in that comes with TensorFlow^34^, we use the ON-LSTM machine learning algorithm for relation extraction. ON-LSTM is a variant of LSTM. For a clear description of ON-LSTM, we illustrate its process step by step. In this section, we first understand the working principle of LSTM.

**Fig. 5:**
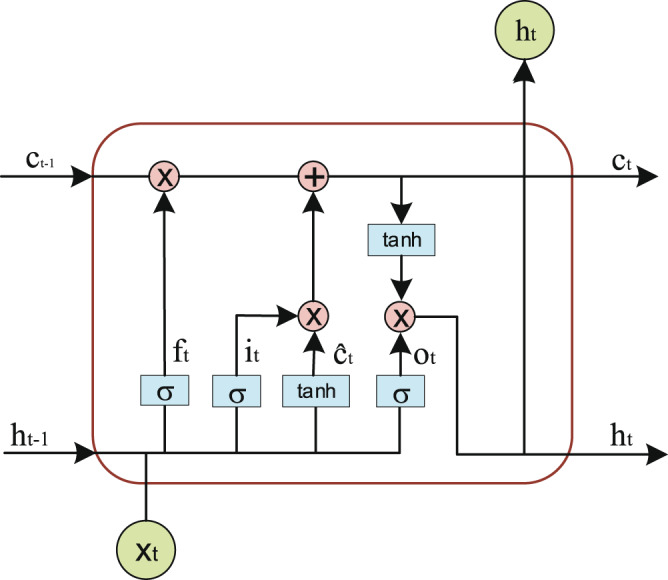
The internal structure of LSTM. An LSTM cell consists of a memory cell *c*_*t*_ and three gates.

LSTM is a special type of recurrent neural network^35^ (RNN) that can learn long-term dependencies. LSTM removes or adds information through its memory cell ${c}_{t}$. As shown in Fig. 5, there are three types of gates, namely forget gate ${f}_{t}$, input gate ${i}_{t}$ and output gate ${o}_{t}$, in *c*_*t*_^36^. The first step of LSTM is to decide what information we will discard from the cell state, which is done through the forget gate. The input is the hidden state ${h}_{t-1}$ of the previous sequence and this sequence of data ${x}_{t}$. The output ${f}_{t}$ of the forget gate represents the probability of forgetting the hidden cell state of the previous layer, and is expressed as follows.

2 $${f}_{t}=\sigma \left({W}_{f}\ast \left[{h}_{t-1},{x}_{t}\right]+{b}_{f}\right)$$

where $\sigma$ is the activation function *sigmoid* and *W*_*f*_ and *b*_*f*_ are the linear correlation coefficient and bias, respectively. The value of *f*_*t*_ is between 0 and 1; here, 0 means that no information is allowed to pass, and 1 means that any information is allowed to pass.

The input gate determines what new information is stored in the cell state. It consists of two parts: the first part uses the *sigmoid* activation function, and its output is ${i}_{t}$. The second part uses the *tanh* activation function, and its output is ${\widehat{c}}_{t}$. The results of the two are multiplied to update the cell state. ${W}_{i}$, ${W}_{c}$, ${b}_{i}$, and ${b}_{c}$ are linearly related coefficients and biases.

3 $${i}_{t}=\sigma \left({W}_{i}\ast \left[{h}_{t-1},{x}_{t}\right]+{b}_{i}\right)$$

4 $${\widehat{c}}_{t}=tanh\left({W}_{c}\ast \left[{h}_{t-1},{x}_{t}\right]+{b}_{c}\right)$$

Next, we need to update the state of the old cell and update ${c}_{t-1}$ to ${c}_{t}$. We multiply the old state by ${f}_{t}$ and discard the information that will certainly be discarded. For the addition of the product of the input gate ${i}_{t}$ and ${\widehat{c}}_{t}$, the formula is as follows.

5 $${c}_{t}={f}_{t}\ast {c}_{t-1}+{i}_{t}\ast {\widehat{c}}_{t}$$

Finally, we need to determine the value to output. The formula for the calculation of *o*_*t*_ is as follows. Here, *w*_0_, and *b*_*o*_ indicate the correlation coefficient and bias.

6 $${o}_{t}=\sigma \left({W}_{o}\ast \left[{h}_{t-1},{x}_{t}\right]+{b}_{0}\right)$$

The update of the hidden state *h*_*t*_ consists of two parts: the first part is *o*_*t*_, and the second part is composed of *c*_*t*_ and activation functions *tanh*.

7 $${h}_{t}={o}_{t}\ast tanh({c}_{t})$$

### ON-LSTM

The new cumax activation function was used according to the previously reported work. The neuron state controls the information to be stored and forgotten. By introducing such a gate mechanism, interdependent update rules between neurons are established so that neurons have an order and a hierarchy of differences.

The object of ON-LSTM thinking is natural language, and nature can usually express some hierarchical structure. In English sentences, letters can be considered the lowest level structure, and words and phrases have a higher level. The higher the level, the coarser the granularity and the greater the span of the sentence. In the ON-LSTM structure, high-level information may retain a considerable distance because the historical information directly copied by the high-level information may cause historical information to be repeated without changing. The low-level information may be updated at each step of input because the low-level information directly duplicates the input. The input is constantly changing, so that the hierarchical structure is embedded through information grading.

The forget gate ${f}_{t}$, input gate ${i}_{t}$, output gate ${o}_{t}$ and ${\widehat{c}}_{t}$ of ON-LSTM given by the same formulas as ct and LSTM, but the update mechanism from ${\widehat{c}}_{t}$ to ${c}_{t}$ is different. The following is the updated formula of the entire ON-LSTM:

8 $${h}_{t}={o}_{t}\ast tanh({c}_{t})$$

9 $${f}_{t}=\sigma \left({W}_{f}\ast \left[{h}_{t-1},{x}_{t}\right]+{b}_{f}\right)$$

10 $${i}_{t}=\sigma \left({W}_{i}\ast \left[{h}_{t-1},{x}_{t}\right]+{b}_{i}\right)$$

11 $${o}_{t}=\sigma \left({W}_{o}\ast \left[{h}_{t-1},{x}_{t}\right]+{b}_{0}\right)$$

12 $${\widehat{c}}_{t}=tanh\left({W}_{c}\ast \left[{h}_{t-1},{x}_{t}\right]+{b}_{c}\right)$$

13 $$\widetilde{{f}_{t}}=cumax\left({W}_{\widehat{f}}\ast \left[{h}_{t-1},{x}_{t}\right]+{b}_{\widehat{f}}\right)$$

14 $$\widetilde{{i}_{t}}=1-cumax\left({W}_{\widehat{i}}\ast \left[{h}_{t-1},{x}_{t}\right]+{b}_{\widehat{i}}\right)$$

15 $${w}_{t}=\widetilde{{f}_{t}}\ast \widetilde{{i}_{t}}$$

16 $${c}_{t}=\widetilde{{f}_{t}}\ast {c}_{t-1}+\widetilde{{i}_{t}}\ast {\widehat{c}}_{t}$$

17 $${h}_{t}={o}_{t}\ast tanh({c}_{t})$$

The value of the *cumax* activation function decreases monotonically from 1 to 0. Within a certain range, its value tends to 0, indicating that the previous information has been forgotten; if its value tends to 1, the new input content becomes increasingly important. When training the model, we set the dropout as 0.4, the learning rate is 0.1, and the dimension of the word vector is 64.

## Supplementary information

DOIs

## Data Availability

Our initial data and extracted data are available at https://github.com/MGEdata/snorkel.

## Code availability

The code is available at https://github.com/MGEdata/auto-generate-corpus. When researchers extract their own corpus, they only need to write labeling functions that meet the characteristics of their own corpus in the framework we write, which is very simple to use.
