# Supplementary material for: Materials information extraction via automatically generated corpus
Source: Sci Data. 2022 Jul 13;9:401. doi: 10.1038/s41597-022-01492-2 (PMC9279422; doi:10.1038/s41597-022-01492-2)
Supplement: Supplementary file 1 — DOIs [file 41597_2022_1492_MOESM1_ESM.pdf]

10.1115/1.1287509
10.1115/1.1287591
10.1115/1.1287593
10.1115/1.1308032
10.1115/1.1372709
10.1115/1.1412453
10.1115/1.1413767
10.1115/1.1423914
10.1115/1.1446865
10.1115/1.1447238
10.1115/1.1455638
10.1115/1.1470480
10.1115/1.1481035
10.1115/1.1494096
10.1115/1.1501075
10.1115/1.1533809
10.1115/1.1584493
10.1115/1.1619428
10.1115/1.1644548
10.1115/1.1651096
10.1115/1.1690768
10.1115/1.1760763
10.1115/1.1763181
10.1115/1.1787508
10.1115/1.1807414
10.1115/1.1836768
10.1115/1.1836793
10.1115/1.1839193
10.1115/1.1850939
10.1115/1.1924560
10.1115/1.2019983
10.1115/1.2180276
10.1115/1.2197843
10.1115/1.2345408
10.1115/1.2709660
10.1115/1.2720867
10.1115/1.2747642
10.1115/1.27476421
10.1115/1.2752831
10.1115/1.2772324
10.1115/1.2789061
10.1115/1.2804529
10.1115/1.2804530
10.1115/1.2804543
10.1115/1.2805928
10.1115/1.2805939
10.1115/1.2805940
10.1115/1.2805984
10.1115/1.2805988
10.1115/1.2806838
10.1115/1.2806840
10.1115/1.2807004
10.1115/1.2812350
10.1115/1.2816301

10.3390/ma10050457
10.3390/ma12152461
10.3390/ma11101978
10.3390/coatings8080264
10.3390/mi9080402
10.3390/met8100748
10.3390/app8112016
10.3390/coatings9020071
10.3390/met9020201
10.3390/ma12060870
10.3390/ma12121905
10.3390/met9060711
10.3390/mi10070476
10.3390/ma12162604
10.3390/coatings9100598
10.3390/coatings7020030
10.3390/ma10030250
10.3390/ma6115275
10.3390/met4010001
10.3390/ma11040561
10.3390/met8060376
10.3390/ma11020240
10.3390/ma8095299
10.3390/met5042428
10.3390/icem18-05415
10.3390/nano9040658
10.3390/ma12091439
10.3390/met8100787
10.3390/ma13092149
10.3390/ma12071172
10.3390/ma11122398
10.3390/met10050629
10.3390/ma13092072
10.3390/cryst10040303
10.3390/coatings10040393
10.3390/en12244610
10.3390/met9111191
10.3390/ma12233829
10.3390/ma13020341
10.3390/app10031187
10.3390/ma13041005
10.3390/met10030321
10.3390/ma13061437
10.3390/ma10050521
10.3390/met8050361
10.3390/coatings10040370
10.3390/ma12122034
10.3390/cryst10070572
10.3390/ma13132990
10.3390/coatings1010003
10.3390/nano10010100
10.3390/ma13143240
10.3390/cryst10080641
10.3390/met10081007

10.1115/1.2816600	10.3390/ma7031625
10.1115/1.2816603	10.3390/met9101130
10.1115/1.2817074	10.3390/met9070720
10.1115/1.2817122	10.3390/ma12121920
10.1115/1.2818131	10.3390/ma12060998
10.1115/1.2818132	10.3390/cryst9030125
10.1115/1.2818133	10.3390/ma11091527
10.1115/1.2818180	10.3390/ma10030279
10.1115/1.2818462	10.3390/met7120561
10.1115/1.2818469	10.3390/chemosensors8030062
10.1115/1.2818473	10.3390/cryst9080420
10.1115/1.2818499	10.3390/met6010002
10.1115/1.2818526	10.3390/ma13163470
10.1115/1.2818527	10.3390/jmmp4010015
10.1115/1.2824211	10.3390/ma12203422
10.1115/1.2831285	10.3390/app6030066
10.1115/1.2834432	10.3390/ma13163570
10.1115/1.2836743	10.3390/ma12193088
10.1115/1.2838990	10.3390/ma11020328
10.1115/1.2840958	10.3390/ma8063428
10.1115/1.2840966	10.3390/ma13173770
10.1115/1.2902142	10.3390/met6030054
10.1115/1.2903178	10.3390/jmmp4030084
10.1115/1.2903180	10.3390/met6070159
10.1115/1.2903190	10.3390/met10091171
10.1115/1.2904149	10.3390/ma13173863
10.1115/1.2904153	10.3390/met10091178
10.1115/1.2904156	10.3390/met8020141
10.1115/1.2904170	10.3390/ma6031118
10.1115/1.2904174	10.3390/met8090723
10.1115/1.2904183	10.3390/ma11081388
10.1115/1.2904229	10.3390/ma13102300
10.1115/1.2904230	10.3390/ma11091528
10.1115/1.2904253	10.3390/met9111179
10.1115/1.2904318	10.3390/met10070915
10.1115/1.2927447	10.3390/ma13184166
10.1115/1.2931148	10.3390/ma11010098
10.1115/1.2943158	10.3390/met9030360
10.1115/1.2967495	10.3390/ma11091525
10.1115/1.2967496	10.3390/ma11060879
10.1115/1.2982152	10.3390/ma11071242
10.1115/1.3007430	10.3390/cryst10100884
10.1115/1.3019056	10.3390/met9070782
10.1115/1.3026576	10.3390/asi1030032
10.1115/1.3094032	10.3390/ma10111236
10.1115/1.3120393	10.3390/ma11061024
10.1115/1.3141437	10.3390/ma12111852
10.1115/1.3148086	10.3390/ma12132096
10.1115/1.3155397	10.3390/ma13194256
10.1115/1.3155793	10.3390/ma13194418
10.1115/1.3173678	10.3390/met6020037
10.1115/1.3205027	10.3390/met6030064
10.1115/1.3224997	10.3390/ma13194452
10.1115/1.3225007	10.3390/ma10040341

10.1115/1.3225009	10.3390/ma13040959
10.1115/1.3225652	10.3390/cryst10100908
10.1115/1.3225675	10.3390/met10101346
10.1115/1.3225680	10.3390/ma12223667
10.1115/1.3225696	10.3390/ma13204553
10.1115/1.3225697	10.3390/met10030362
10.1115/1.3225707	10.3390/cryst9060312
10.1115/1.3225795	10.3390/ma13102397
10.1115/1.3225840	10.3390/ma13204643
10.1115/1.3225952	10.3390/met10060799
10.1115/1.3225980	10.3390/ma13163629
10.1115/1.3226003	10.3390/ma13214758
10.1115/1.3226050	10.3390/app9061112
10.1115/1.3226066	10.3390/app7020124
10.1115/1.3226471	10.3390/met6010005
10.1115/1.3239686	10.3390/met10111453
10.1115/1.3240013	10.3390/met10010021
10.1115/1.3240120	10.3390/met10111460
10.1115/1.3264242	10.3390/ma6115016
10.1115/1.3408607	10.3390/met7090367
10.1115/1.3423888	10.3390/ma12060974
10.1115/1.3439065	10.3390/nano8110873
10.1115/1.3443705	10.3390/ma13214859
10.1115/1.3443710	10.3390/app8122439
10.1115/1.3445912	10.3390/met10070920
10.1115/1.3453235	10.3390/met8100843
10.1115/1.4000102	10.3390/met10040466
10.1115/1.4000136	10.3390/met9111153
10.1115/1.4000149	10.3390/met10111466
10.1115/1.4000666	10.3390/ma13214930
10.1115/1.4001085	10.3390/met6070161
10.1115/1.4001301	10.3390/met8060387
10.1115/1.4001938	10.3390/met8121007
10.1115/1.4002154	10.3390/ma10020161
10.1115/1.4002177	10.3390/met6100238
10.1115/1.4002455	10.3390/ma11101838
10.1115/1.4002497	10.3390/ma13225172
10.1115/1.4002675	10.3390/cryst10111047
10.1115/1.4002679	10.3390/met8080632
10.1115/1.4002816	10.3390/met8080613
10.1115/1.4002821	10.3390/met9010047
10.1115/1.4002824	10.3390/ma11081288
10.1115/1.4002828	10.3390/ma6020437
10.1115/1.4003111	10.3390/e18030102
10.1115/1.4003334	10.3390/ma13235358
10.1115/1.4003602	10.3390/ma12142272
10.1115/1.4003777	10.3390/met10121588
10.1115/1.4003833	10.3390/ma12091390
10.1115/1.4003962	10.3390/ma13102367
10.1115/1.4004050	10.3390/met10040454
10.1115/1.4004131	10.3390/met10040426
10.1115/1.4004731	10.3390/met5042236
10.1115/1.4005308	10.3390/met8010004
10.1115/1.4005946	10.3390/met10121606

10.1115/1.4006228	10.3390/ma13235478
10.1115/1.4006461	10.3390/ma12071144
10.1115/1.4006639	10.3390/ma13153392
10.1115/1.4007686	10.3390/ma13214927
10.1115/1.4007785	10.3390/met10121600
10.1115/1.4025080	10.3390/met6110258
10.1115/1.4025264	10.3390/met7100441
10.1115/1.4025496	10.3390/met9080813
10.1115/1.4025555	10.3390/met9091001
10.1115/1.4025782	10.3390/met9111146
10.1115/1.4026035	10.3390/lubricants8010004
10.1115/1.4026271	10.3390/ma13173849
10.1115/1.4027186	10.3390/ma13183924
10.1115/1.4027207	10.3390/ma13183959
10.1115/1.4027619	10.3390/ma11102065
10.1115/1.4027857	10.3390/app10238761

10.1115/1.4027929
10.1115/1.4028509
10.1115/1.4028936
10.1115/1.4029908
10.1115/1.4030319
10.1115/1.4031155
10.1115/1.4031157
10.1115/1.4031158
10.1115/1.4031240
10.1115/1.4031318
10.1115/1.4031396
10.1115/1.4031712
10.1115/1.4032438
10.1115/1.4032704
10.1115/1.4034147
10.1115/1.4034712
10.1115/1.4034850
10.1115/1.4034934
10.1115/1.4035560
10.1115/1.4035903
10.1115/1.4036714
10.1115/1.4037231
10.1115/1.4037250
10.1115/1.4037275
10.1115/1.4037604
10.1115/1.4037660
10.1115/1.4038351
10.1115/1.4039109
10.1115/1.4039779
10.1115/1.4040011
10.1115/1.4040087
10.1115/1.4040117
10.1115/1.4040222
10.1115/1.4040333
10.1115/1.4040554
10.1115/1.4041009
10.1115/1.4041072
10.1115/1.4041309
10.1115/1.4041385
10.1115/1.4041949
10.1115/1.4042009
10.1115/1.4042274
10.1115/1.4043159
10.1115/1.4043555
10.1115/1.4043626
10.1115/1.4044007
10.1115/1.482801
10.1115/1.482803
10.1115/1.483175

10.1038/am.2015.96
10.1038/nature19313
10.1038/nature23894
10.1038/ncomms13434
10.1038/ncomms3955
10.1038/ncomms7164
10.1038/ncomms8267
10.1038/s41467-017-00383-0
10.1038/s41467-017-00814-y
10.1038/s41467-018-04473-5
10.1038/s41467-018-05549-y
10.1038/s41467-019-13874-z
10.1038/s41467-019-14062-9
10.1038/s41467-020-14820-0
10.1038/s41524-018-0072-0
10.1038/s41526-019-0067-2
10.1038/s41529-018-0046-1
10.1038/s41529-018-0060-3
10.1038/s41563-018-0105-6
10.1038/s41586-018-0593-1
10.1038/s41598-017-03051-x
10.1038/s41598-017-03770-1
10.1038/s41598-017-04163-0
10.1038/s41598-017-07884-4
10.1038/s41598-017-10091-w
10.1038/s41598-017-11540-2
10.1038/s41598-017-13026-7
10.1038/s41598-017-17456-1
10.1038/s41598-018-19800-5
10.1038/s41598-018-23556-3
10.1038/s41598-018-26426-0
10.1038/s41598-018-26917-0
10.1038/s41598-019-43819-x
10.1038/s41598-019-50361-3
10.1038/s41598-019-55653-2
10.1038/s41598-020-59968-3
10.1038/srep06200
10.1038/srep07367
10.1038/srep11772
10.1038/srep14903
10.1038/srep16081
10.1038/srep16446
10.1038/srep17960
10.1038/srep19363
10.1038/srep21232
10.1038/srep22306
10.1038/srep23324
10.1038/srep26535
10.1038/srep28144
10.1038/srep28650
10.1038/srep29019
10.1038/srep29941
10.1038/srep45965
10.1007/bf00012425
10.1007/bf00017827
10.1007/bf00018655
10.1007/bf00018922
10.1007/bf00019920
10.1007/bf00035369
10.1007/bf00053517
10.1007/bf00241016
10.1007/bf00271015
10.1007/bf00277989
10.1007/bf00278146
10.1007/bf00291435
10.1007/bf00321330
10.1007/bf00322050
10.1007/bf00349910
10.1007/bf00351289
10.1007/bf00352065
10.1007/bf00353050
10.1007/bf00354696
10.1007/bf00355152
10.1007/bf00355867
10.1007/bf00355908
10.1007/bf00355929
10.1007/bf00356005
10.1007/bf00356063
10.1007/bf00356148
10.1007/bf00356225
10.1007/bf00356645
10.1007/bf00356688
10.1007/bf00356723
10.1007/bf00356809
10.1007/bf00357329
10.1007/bf00361524
10.1007/bf00363439
10.1007/bf00365177
10.1007/bf00366345
10.1007/bf00366358
10.1007/bf00369679
10.1007/bf00405044
10.1007/bf00414229
10.1007/bf00414271
10.1007/bf00420203
10.1007/bf00420520
10.1007/bf00420541
10.1007/bf00420543
10.1007/bf00461387
10.1007/bf00462203
10.1007/bf00540170
10.1007/bf00540433
10.1007/bf00540467
10.1007/bf00540691
10.1007/bf00540748
10.1007/bf00541420
10.1080/00084433.2015.1104059
10.1080/00084433.2016.1267298
10.1080/00084433.2020.1741899
10.1080/00150193.2017.1391591
10.1080/00150193.2018.1453123
10.1080/00150193.2019.1592466
10.1080/00150193.2019.1592543
10.1080/00194506.2016.1256236
10.1080/00202967.2003.11871479
10.1080/00202967.2016.1270616
10.1080/00207549208948164
10.1080/00223131.2012.669238
10.1080/00223131.2012.703948
10.1080/00319104.2012.673613
10.1080/00319104.2015.1095640
10.1080/00325899.2015.1138022
10.1080/00325899.2016.1142058
10.1080/00325899.2016.1243338
10.1080/00325899.2016.1269457
10.1080/00325899.2016.1270389
10.1080/00325899.2017.1298875
10.1080/00325899.2018.1546921
10.1080/00325899.2019.1616367
10.1080/00325899.2019.1637171
10.1080/00986440802557518
10.1080/01411594.2011.582379
10.1080/01411594.2011.594985
10.1080/01411594.2014.906039
10.1080/01411594.2019.1692015
10.1080/01418610.1985.12069159
10.1080/01418610108216658
10.1080/01418610108217148
10.1080/01418610110102482
10.1080/01418610110105650
10.1080/01418610208235690
10.1080/01418610210125783
10.1080/01418610210145358
10.1080/01418610210146050
10.1080/01418610210161602
10.1080/0141861031000109573
10.1080/01418618008239328
10.1080/01418618108235799
10.1080/01418618408244216
10.1080/01418618508237581
10.1080/01418618508237591
10.1080/01418618508237592
10.1080/01418618608242884
10.1080/01418618708214380
10.1080/01418618708214399
10.1080/01418618808204522
10.1080/01418618808204525
10.1080/01418618808209940
10.1080/01418618908209820

10.1002/(sici)1096-99
10.1002/(sici)1097-45
10.1002/(sici)1097-46
10.1002/(sici)1521-39
10.1002/(sici)1521-41
10.1002/(sici)1527-26
10.1002/1096-9918(200
10.1002/1097-0363(200
10.1002/1521-396x(199
10.1002/1521-396x(200
10.1002/1521-4052(200
10.1002/1521-4176(200
10.1002/1527-2648(200
10.1002/adem.200300322
10.1002/adem.200310083
10.1002/adem.200310095
10.1002/adem.200500277
10.1002/adem.200900042
10.1002/adem.201000089
10.1002/adem.201000145
10.1002/adem.201000163
10.1002/adem.201000232
10.1002/adem.201000366
10.1002/adem.201100150
10.1002/adem.201100194
10.1002/adem.201100349
10.1002/adem.201200055
10.1002/adem.201300136
10.1002/adem.201300430
10.1002/adem.201400136
10.1002/adem.201400223
10.1002/adem.201400249
10.1002/adem.201400266
10.1002/adem.201400299
10.1002/adem.201400365
10.1002/adem.201400400
10.1002/adem.201400589
10.1002/adem.201500037
10.1002/adem.201500088
10.1002/adem.201500146
10.1002/adem.201500158
10.1002/adem.201500159
10.1002/adem.201500168
10.1002/adem.201500171
10.1002/adem.201500173
10.1002/adem.201500296
10.1002/adem.201500547
10.1002/adem.201600071
10.1002/adem.201600127
10.1002/adem.201600237
10.1002/adem.201600270
10.1002/adem.201600320
10.1002/adem.201600475

10.1007/bf00542087	10.1080/01418618908209821	10.1002/adem.201600529
10.1007/bf00542317	10.1080/01418619008243921	10.1002/adem.201600535
10.1007/bf00542381	10.1080/01418619008244336	10.1002/adem.201600635
10.1007/bf00542912	10.1080/01418619108204866	10.1002/adem.201600672
10.1007/bf00543618	10.1080/01418619108204867	10.1002/adem.201600690
10.1007/bf00544162	10.1080/01418619108204868	10.1002/adem.201600721
10.1007/bf00544198	10.1080/01418619108205586	10.1002/adem.201700041
10.1007/bf00544199	10.1080/01418619208201587	10.1002/adem.201700150
10.1007/bf00544533	10.1080/01418619208205607	10.1002/adem.201700182
10.1007/bf00545469	10.1080/01418619208205609	10.1002/adem.201700297
10.1007/bf00545480	10.1080/01418619208205611	10.1002/adem.201700502
10.1007/bf00549796	10.1080/01418619308219366	10.1002/adem.201700568
10.1007/bf00550537	10.1080/01418619308222923	10.1002/adem.201700645
10.1007/bf00551018	10.1080/01418619408242213	10.1002/adem.201700820
10.1007/bf00551281	10.1080/01418619508236228	10.1002/adem.201700930
10.1007/bf00551321	10.1080/01418619508236233	10.1002/adem.201700960
10.1007/bf00551462	10.1080/01418619508236248	10.1002/adem.201700973
10.1007/bf00551493	10.1080/01418619508239932	10.1002/adem.201700987
10.1007/bf00551505	10.1080/01418619508239952	10.1002/adem.201701011
10.1007/bf00551985	10.1080/01418619508243792	10.1002/adem.201701043
10.1007/bf00552407	10.1080/01418619608239688	10.1002/adem.201701114
10.1007/bf00553215	10.1080/01418619608239699	10.1002/adem.201701189
10.1007/bf00553420	10.1080/01418619708210282	10.1002/adem.201800129
10.1007/bf00553814	10.1080/01418619708214021	10.1002/adem.201800234
10.1007/bf00554772	10.1080/01418619908210378	10.1002/adem.201800279
10.1007/bf00556076	10.1080/01418619908212011	10.1002/adem.201800307
10.1007/bf00557126	10.1080/01418619908212034	10.1002/adem.201800351
10.1007/bf00560640	10.1080/01495728108961787	10.1002/adem.201800647
10.1007/bf00570396	10.1080/01495739.2017.1352463	10.1002/adem.201800652
10.1007/bf00570398	10.1080/014957390523651	10.1002/adem.201800793
10.1007/bf00576274	10.1080/01495739408946255	10.1002/adem.201800856
10.1007/bf00576299	10.1080/01694243.2016.1146393	10.1002/adem.201800933
10.1007/bf00576301	10.1080/01694243.2017.1310172	10.1002/adem.201801022
10.1007/bf00581093	10.1080/01694243.2019.1620424	10.1002/adem.201801318
10.1007/bf00582481	10.1080/01932691.2019.1649154	10.1002/adem.201801354
10.1007/bf00587693	10.1080/02533839.2007.9671247	10.1002/adem.201900054
10.1007/bf00595740	10.1080/02533839.2010.9671680	10.1002/adem.201900267
10.1007/bf00603531	10.1080/02533839.2012.701896	10.1002/adem.201900426
10.1007/bf00603578	10.1080/02619180.1985.11753275	10.1002/adem.201900499
10.1007/bf00603610	10.1080/02619180.1985.11753276	10.1002/adem.201900543
10.1007/bf00605108	10.1080/02619180.1986.11753313	10.1002/adem.201900558
10.1007/bf00606191	10.1080/02619180.1986.11753319	10.1002/adem.201900641
10.1007/bf00608013	10.1080/02619180.1986.11753320	10.1002/adem.201900823
10.1007/bf00610640	10.1080/02619180.1987.11753335	10.1002/adem.201900892
10.1007/bf00611474	10.1080/02619180.1987.11753357	10.1002/adem.201900930
10.1007/bf00611601	10.1080/02619180.1988.11753371	10.1002/adem.201901151
10.1007/bf00625005	10.1080/02619180.1988.11753384	10.1002/adem.201901228
10.1007/bf00638021	10.1080/02619180.1988.11753389	10.1002/adem.201901237
10.1007/bf00644668	10.1080/02619180.1988.11753391	10.1002/adem.201901244
10.1007/bf00644688	10.1080/02619180.1989.11753418	10.1002/adem.201901445
10.1007/bf00647782	10.1080/02619180.1989.11753426	10.1002/adma.19940060
10.1007/bf00655894	10.1080/02619180.1989.11753437	10.1002/adma.19960080
10.1007/bf00655896	10.1080/02619180.1990.11753477	10.1002/adma.200702461
10.1007/bf00656576	10.1080/02619180.1990.11753492	10.1002/adma.201003600

10.1007/bf00656643	10.1080/02619180.1990.11753493	10.1002/adma.201200764
10.1007/bf00656646	10.1080/02670836.1987.11782259	10.1002/adma.201907164
10.1007/bf00656727	10.1080/02670836.1987.11782262	10.1002/aic.16383
10.1007/bf00656844	10.1080/02670836.1987.11782264	10.1002/bbpc.19970000
10.1007/bf00656898	10.1080/02670836.1987.11782265	10.1002/bbpc.19981020
10.1007/bf00656900	10.1080/02670836.1987.11782266	10.1002/ceat.201700680
10.1007/bf00659249	10.1080/02670836.1987.11782269	10.1002/chem.201701081
10.1007/bf00662037	10.1080/02670836.1987.11782270	10.1002/chem.201702231
10.1007/bf00664272	10.1080/02670836.1996.11665715	10.1002/cite.201300191
10.1007/bf00664273	10.1080/02670836.2016.1139225	10.1002/cjce.23019
10.1007/bf00664277	10.1080/02670836.2016.1148227	10.1002/cnm.752
10.1007/bf00664423	10.1080/02670836.2016.1149277	10.1002/crat.19780131
10.1007/bf00664494	10.1080/02670836.2016.1159002	10.1002/crat.201000389
10.1007/bf00664496	10.1080/02670836.2016.1187335	10.1002/crat.201100391
10.1007/bf00664662	10.1080/02670836.2016.1204070	10.1002/crat.201600372
10.1007/bf00664711	10.1080/02670836.2016.1215961	10.1002/crat.201700012
10.1007/bf00664797	10.1080/02670836.2016.1216029	10.1002/crat.201700187
10.1007/bf00664803	10.1080/02670836.2016.1230168	10.1002/crat.201800108
10.1007/bf00665022	10.1080/02670836.2016.1242827	10.1002/crat.201800177
10.1007/bf00665047	10.1080/02670836.2016.1273866	10.1002/cvde.201307013
10.1007/bf00665258	10.1080/02670836.2017.1282668	10.1002/cvde.201307090
10.1007/bf00665259	10.1080/02670836.2017.1288674	10.1002/er.1457
10.1007/bf00665269	10.1080/02670836.2017.1295212	10.1002/fam.906
10.1007/bf00665447	10.1080/02670836.2017.1300365	10.1002/htj.10088
10.1007/bf00665490	10.1080/02670836.2017.1300419	10.1002/jbm.820220706
10.1007/bf00665491	10.1080/02670836.2017.1333222	10.1002/jbm.b.30585
10.1007/bf00665612	10.1080/02670836.2017.1337299	10.1002/jemt.10600704
10.1007/bf00665614	10.1080/02670836.2017.1345823	10.1002/jemt.10601102
10.1007/bf00665662	10.1080/02670836.2017.1353662	10.1002/jemt.22359
10.1007/bf00665670	10.1080/02670836.2017.1389118	10.1002/jemt.23417
10.1007/bf00665673	10.1080/02670836.2017.1393977	10.1002/ls.1254
10.1007/bf00666595	10.1080/02670836.2017.1407566	10.1002/maco.19840350
10.1007/bf00666598	10.1080/02670836.2017.1410929	10.1002/maco.19900410
10.1007/bf00666599	10.1080/02670836.2017.1410953	10.1002/maco.19920430
10.1007/bf00666645	10.1080/02670836.2017.1419612	10.1002/maco.19950460
10.1007/bf00666806	10.1080/02670836.2018.1424379	10.1002/maco.19950461
10.1007/bf00666913	10.1080/02670836.2018.1428405	10.1002/maco.19960471
10.1007/bf00667421	10.1080/02670836.2018.1429043	10.1002/maco.200303709
10.1007/bf00687309	10.1080/02670836.2018.1461594	10.1002/maco.200303728
10.1007/bf00699521	10.1080/02670836.2018.1471436	10.1002/maco.200303759
10.1007/bf00699522	10.1080/02670836.2018.1505227	10.1002/maco.200390027
10.1007/bf00699523	10.1080/02670836.2018.1523518	10.1002/maco.200403808
10.1007/bf00699525	10.1080/02670836.2018.1528733	10.1002/maco.200503870
10.1007/bf00699584	10.1080/02670836.2018.1563982	10.1002/maco.200503879
10.1007/bf00700772	10.1080/02670836.2019.1570440	10.1002/maco.200503896
10.1007/bf00700780	10.1080/02670836.2019.1570662	10.1002/maco.200503898
10.1007/bf00701068	10.1080/02670836.2019.1572298	10.1002/maco.200503900
10.1007/bf00702542	10.1080/02670836.2019.1590503	10.1002/maco.200503901
10.1007/bf00705400	10.1080/02670836.2019.1591031	10.1002/maco.200503902
10.1007/bf00705444	10.1080/02670836.2019.1618048	10.1002/maco.200503907
10.1007/bf00705445	10.1080/02670836.2019.1619304	10.1002/maco.200503924
10.1007/bf00705448	10.1080/02670836.2019.1619305	10.1002/maco.200503926
10.1007/bf00712552	10.1080/02670836.2019.1633726	10.1002/maco.200603985
10.1007/bf00713730	10.1080/02670836.2019.1651475	10.1002/maco.200604012

10.1007/bf00719692	10.1080/02670836.2019.1667672	10.1002/maco.200604013
10.1007/bf00720079	10.1080/02670836.2019.1681157	10.1002/maco.200704072
10.1007/bf00720082	10.1080/02670836.2019.1706906	10.1002/maco.200804119
10.1007/bf00720170	10.1080/02670836.2019.1710927	10.1002/maco.200804123
10.1007/bf00720492	10.1080/02670836.2020.1740380	10.1002/maco.200804130
10.1007/bf00721922	10.1080/02670836.2020.1740864	10.1002/maco.200804131
10.1007/bf00722338	10.1080/02670844.2015.1119507	10.1002/maco.200804141
10.1007/bf00722876	10.1080/02670844.2016.1148381	10.1002/maco.200804144
10.1007/bf00723460	10.1080/02670844.2016.1182959	10.1002/maco.200805009
10.1007/bf00725861	10.1080/02670844.2016.1199128	10.1002/maco.200805174
10.1007/bf00725881	10.1080/02670844.2016.1200847	10.1002/maco.200905376
10.1007/bf00727699	10.1080/02670844.2016.1258770	10.1002/maco.200905519
10.1007/bf00728886	10.1080/02670844.2016.1259731	10.1002/maco.201005854
10.1007/bf00729263	10.1080/02670844.2017.1287555	10.1002/maco.201005877
10.1007/bf00729374	10.1080/02670844.2017.1312220	10.1002/maco.201005879
10.1007/bf00729398	10.1080/02670844.2017.1369667	10.1002/maco.201005881
10.1007/bf00729412	10.1080/02670844.2017.1371872	10.1002/maco.201106250
10.1007/bf00729659	10.1080/02670844.2017.1416944	10.1002/maco.201206694
10.1007/bf00731099	10.1080/02670844.2018.1501936	10.1002/maco.201206773
10.1007/bf00737038	10.1080/02670844.2018.1564474	10.1002/maco.201307070
10.1007/bf00737041	10.1080/02670844.2019.1611195	10.1002/maco.201307106
10.1007/bf00738291	10.1080/02670844.2019.1653597	10.1002/maco.201307127
10.1007/bf00738383	10.1080/02670844.2019.1662225	10.1002/maco.201307143
10.1007/bf00742189	10.1080/02670844.2020.1741212	10.1002/maco.201307231
10.1007/bf00742190	10.1080/02726351.2016.1150371	10.1002/maco.201307299
10.1007/bf00751802	10.1080/03019233.2016.1253447	10.1002/maco.201307331
10.1007/bf00752193	10.1080/0371750x.2011.10600158	10.1002/maco.201307425
10.1007/bf00752209	10.1080/0371750x.2018.1455537	10.1002/maco.201307534
10.1007/bf00767219	10.1080/05698198508981629	10.1002/maco.201407954
10.1007/bf00767436	10.1080/08827508.2018.1514300	10.1002/maco.201408046
10.1007/bf00767554	10.1080/08927022.2015.1012642	10.1002/maco.201408056
10.1007/bf00767735	10.1080/08927022.2018.1521969	10.1002/maco.201508779
10.1007/bf00769428	10.1080/08927022.2019.1614177	10.1002/maco.201608837
10.1007/bf00769429	10.1080/08981500210000030111	10.1002/maco.201608998
10.1007/bf00769431	10.1080/09276440.2020.1747346	10.1002/maco.201609024
10.1007/bf00771271	10.1080/09349840600787931	10.1002/maco.201609265
10.1007/bf00771272	10.1080/09500830110084993	10.1002/maco.201609276
10.1007/bf00774634	10.1080/0950083031000064399	10.1002/maco.201709488
10.1007/bf00774908	10.1080/09500830600618215	10.1002/maco.201709513
10.1007/bf00775119	10.1080/09500830801905445	10.1002/maco.201709820
10.1007/bf00775120	10.1080/09500830802311080	10.1002/maco.201810153
10.1007/bf00775311	10.1080/09500830902877790	10.1002/maco.201810159
10.1007/bf00778266	10.1080/09500830903292346	10.1002/maco.201810720
10.1007/bf00780090	10.1080/09500838708210432	10.1002/maco.201810751
10.1007/bf00780805	10.1080/09500839.2010.541164	10.1002/maco.201910856
10.1007/bf00802107	10.1080/09500839.2011.565812	10.1002/maco.201910971
10.1007/bf00802271	10.1080/09500839.2011.587463	10.1002/maco.201911116
10.1007/bf00802573	10.1080/09500839.2011.597358	10.1002/maco.201911167
10.1007/bf00806269	10.1080/09500839.2012.687838	10.1002/mawe.19880190
10.1007/bf00806803	10.1080/09500839.2012.690904	10.1002/mawe.19910221
10.1007/bf00808050	10.1080/09500839.2012.699686	10.1002/mawe.19960270
10.1007/bf00809036	10.1080/09500839.2012.700409	10.1002/mawe.19970280
10.1007/bf00872818	10.1080/09500839.2013.816446	10.1002/mawe.19970281
10.1007/bf00980749	10.1080/09500839.2013.870670	10.1002/mawe.19980290

10.1007/bf00980754	10.1080/09500839.2014.920538	10.1002/mawe.200300663
10.1007/bf01026314	10.1080/09500839.2014.987841	10.1002/mawe.200600067
10.1007/bf01031858	10.1080/09500839.2014.995740	10.1002/mawe.200700193
10.1007/bf01045364	10.1080/09500839.2015.1039621	10.1002/mawe.200700194
10.1007/bf01046725	10.1080/09500839.2015.1039622	10.1002/mawe.200700234
10.1007/bf01046727	10.1080/09500839.2015.1076175	10.1002/mawe.200800453
10.1007/bf01046827	10.1080/09500839.2015.1080389	10.1002/mawe.201000694
10.1007/bf01046985	10.1080/09500839.2015.1100763	10.1002/mawe.201300165
10.1007/bf01047033	10.1080/09500839.2015.1109716	10.1002/mawe.201300984
10.1007/bf01047034	10.1080/09500839.2015.1125539	10.1002/mawe.201400195
10.1007/bf01047036	10.1080/09500839.2015.1134832	10.1002/mawe.201400196
10.1007/bf01048641	10.1080/09500839.2016.1195931	10.1002/mawe.201400204
10.1007/bf01058247	10.1080/09500839.2016.1200756	10.1002/mawe.201400235
10.1007/bf01058828	10.1080/09500839.2016.1244358	10.1002/mawe.201400263
10.1007/bf01072916	10.1080/09500839.2016.1252860	10.1002/mawe.201400312
10.1007/bf01080780	10.1080/09500839.2016.1273554	10.1002/mawe.201400371
10.1007/bf01080783	10.1080/09500839.2017.1288941	10.1002/mawe.201500379
10.1007/bf01080784	10.1080/09500839.2017.1311427	10.1002/mawe.201500406
10.1007/bf01082142	10.1080/09500839.2017.1356478	10.1002/mawe.201500408
10.1007/bf01103510	10.1080/09500839.2017.1396373	10.1002/mawe.201600534
10.1007/bf01103527	10.1080/09500839.2017.1406193	10.1002/mawe.201600718
10.1007/bf01105045	10.1080/09500839.2018.1554913	10.1002/mawe.201600726
10.1007/bf01105682	10.1080/09500839.2019.1597991	10.1002/mawe.201600740
10.1007/bf01106536	10.1080/09500839.2020.1741043	10.1002/mawe.201700072
10.1007/bf01111880	10.1080/09500839108201962	10.1002/mawe.201700113
10.1007/bf01111928	10.1080/09500839108214621	10.1002/mawe.201700155
10.1007/bf01112297	10.1080/09500839108214675	10.1002/mawe.201700170
10.1007/bf01113760	10.1080/09500839208207547	10.1002/mawe.201700187
10.1007/bf01114297	10.1080/09500839208219032	10.1002/mawe.201700222
10.1007/bf01115723	10.1080/09500839208229276	10.1002/mawe.201700259
10.1007/bf01115749	10.1080/09500839408240974	10.1002/mawe.201800108
10.1007/bf01115770	10.1080/095008396180290	10.1002/mawe.201800135
10.1007/bf01117940	10.1080/09506608.2015.1109214	10.1002/mawe.201800144
10.1007/bf01120032	10.1080/09506608.2017.1410944	10.1002/mawe.201800153
10.1007/bf01122466	10.1080/09507110701774040	10.1002/mawe.201900019
10.1007/bf01122472	10.1080/09507111003655226	10.1002/nme.162030070
10.1007/bf01124690	10.1080/09507116.2011.600039	10.1002/nme.3172
10.1007/bf01129930	10.1080/09507116.2014.897805	10.1002/pamm.201410208
10.1007/bf01129945	10.1080/09507116.2015.1096558	10.1002/pamm.201510127
10.1007/bf01132012	10.1080/09507116.2017.1318496	10.1002/pamm.201710187
10.1007/bf01132050	10.1080/09603409.1991.11689634	10.1002/pamm.201800389
10.1007/bf01132373	10.1080/09603409.1991.11689644	10.1002/ppap.200931502
10.1007/bf01132914	10.1080/09603409.1991.11689653	10.1002/pssa.22107201
10.1007/bf01139050	10.1080/09603409.1991.11689655	10.1002/pssa.22107402
10.1007/bf01139073	10.1080/09603409.1991.11689663	10.1002/pssa.22108102
10.1007/bf01141961	10.1080/09603409.1992.11689394	10.1002/pssa.22108302
10.1007/bf01145517	10.1080/09603409.1992.11689404	10.1002/pssa.22109902
10.1007/bf01151091	10.1080/09603409.1992.11689408	10.1002/pssa.22110901
10.1007/bf01151264	10.1080/09603409.1992.11689412	10.1002/pssa.22111002
10.1007/bf01151498	10.1080/09603409.1992.11689416	10.1002/pssa.22111901
10.1007/bf01151796	10.1080/09603409.1994.11689477	10.1002/pssa.22112501
10.1007/bf01152222	10.1080/09603409.1994.11689483	10.1002/pssa.22112801
10.1007/bf01152223	10.1080/09603409.1994.11752529	10.1002/pssa.22112802
10.1007/bf01152957	10.1080/09603409.1994.11752532	10.1002/pssa.22113001

10.1007/bf01152962	10.1080/09603409.1995.11689500	10.1002/pssa.22113102
10.1007/bf01154109	10.1080/09603409.1995.11689501	10.1002/pssa.22113302
10.1007/bf01154941	10.1080/09603409.1995.11689508	10.1002/pssa.22113702
10.1007/bf01156810	10.1080/09603409.1997.11689525	10.1002/pssa.22113801
10.1007/bf01162514	10.1080/09603409.1997.11689526	10.1002/pssa.22114201
10.1007/bf01168952	10.1080/09603409.1997.11689527	10.1002/pssa.22114401
10.1007/bf01171556	10.1080/09603409.1997.11689537	10.1002/pssa.22114802
10.1007/bf01174060	10.1080/09603409.1997.11689555	10.1002/pssa.22114901
10.1007/bf01174485	10.1080/09603409.1997.11689567	10.1002/pssa.22114902
10.1007/bf01174505	10.1080/09603409.1998.11689571	10.1002/pssb.201552149
10.1007/bf01174710	10.1080/09603409.2015.1106786	10.1002/pssb.201600634
10.1007/bf01178179	10.1080/09603409.2015.1132528	10.1002/pssb.201600839
10.1007/bf01184591	10.1080/09603409.2016.1139336	10.1002/pssb.201600860
10.1007/bf01184986	10.1080/09603409.2016.1143149	10.1002/qua.22962
10.1007/bf01191970	10.1080/09603409.2016.1152421	10.1002/sia.1086
10.1007/bf01196644	10.1080/09603409.2016.1155689	10.1002/sia.1225
10.1007/bf01197661	10.1080/09603409.2016.1159836	10.1002/sia.1349
10.1007/bf01225978	10.1080/09603409.2016.1160501	10.1002/sia.1696
10.1007/bf01233155	10.1080/09603409.2016.1161945	10.1002/sia.1708
10.1007/bf01395222	10.1080/09603409.2016.1163810	10.1002/sia.2515
10.1007/bf01401081	10.1080/09603409.2016.1165449	10.1002/sia.2586
10.1007/bf01410513	10.1080/09603409.2016.1166686	10.1002/sia.3031
10.1007/bf01522739	10.1080/09603409.2016.1169665	10.1002/sia.3332
10.1007/bf01522772	10.1080/09603409.2016.1171952	10.1002/sia.3811
10.1007/bf01522927	10.1080/09603409.2016.1176753	10.1002/sia.4844
10.1007/bf01523038	10.1080/09603409.2016.1177997	10.1002/sia.5041
10.1007/bf01523058	10.1080/09603409.2016.1179000	10.1002/sia.5307
10.1007/bf01523112	10.1080/09603409.2016.1180276	10.1002/sia.5526
10.1007/bf01523147	10.1080/09603409.2016.1180857	10.1002/sia.5680
10.1007/bf01523238	10.1080/09603409.2016.1180858	10.1002/sia.5718
10.1007/bf01523373	10.1080/09603409.2016.1182250	10.1002/sia.5720
10.1007/bf01523374	10.1080/09603409.2016.1182259	10.1002/sia.5784
10.1007/bf01523403	10.1080/09603409.2016.1182960	10.1002/sia.5946
10.1007/bf01523476	10.1080/09603409.2016.1186414	10.1002/sia.6389
10.1007/bf01523477	10.1080/09603409.2016.1187464	10.1002/sia.740050502
10.1007/bf01523487	10.1080/09603409.2016.1189022	10.1002/sia.740090312
10.1007/bf01524181	10.1080/09603409.2016.1190147	10.1002/sia.740100212
10.1007/bf01524464	10.1080/09603409.2016.1190161	10.1002/sia.740100604
10.1007/bf01525356	10.1080/09603409.2016.1193995	10.1002/sia.740120304
10.1007/bf01528545	10.1080/09603409.2016.1222051	10.1002/sia.740120713
10.1007/bf01528675	10.1080/09603409.2016.1231861	10.1002/sia.740141103
10.1007/bf01528678	10.1080/09603409.2016.1234663	10.1002/sia.740181005
10.1007/bf01528960	10.1080/09603409.2016.1244373	10.1002/srin.19760378
10.1007/bf01529109	10.1080/09603409.2016.1252164	10.1002/srin.19810490
10.1007/bf01529259	10.1080/09603409.2016.1258153	10.1002/srin.19870148
10.1007/bf01529407	10.1080/09603409.2016.1271763	10.1002/srin.19900034
10.1007/bf01529631	10.1080/09603409.2017.1281869	10.1002/srin.19920173
10.1007/bf01529739	10.1080/09603409.2017.1355091	10.1002/srin.19930105
10.1007/bf01529940	10.1080/09603409.2017.1360435	10.1002/srin.19960546
10.1007/bf01530137	10.1080/09603409.2017.1366402	10.1002/srin.19960547
10.1007/bf01530199	10.1080/09603409.2017.1367155	10.1002/srin.19960550
10.1007/bf01530852	10.1080/09603409.2017.1369665	10.1002/srin.19980557
10.1007/bf01530855	10.1080/09603409.2017.1380943	10.1002/srin.200405927
10.1007/bf01532356	10.1080/09603409.2017.1381498	10.1002/srin.200405929

10.1007/bf01532782	10.1080/09603409.2017.1386344	10.1002/srin.200506024
10.1007/bf01533398	10.1080/09603409.2017.1389100	10.1002/srin.200506062
10.1007/bf01533646	10.1080/09603409.2017.1389132	10.1002/srin.200706255
10.1007/bf01571674	10.1080/09603409.2017.1392111	10.1002/srin.201300116
10.1007/bf01668514	10.1080/09603409.2017.1392114	10.1002/srin.201500410
10.1007/bf01670500	10.1080/09603409.2017.1392413	10.1002/srin.201600433
10.1007/bf01675260	10.1080/09603409.2017.1392414	10.1002/srin.201700565
10.1007/bf01694839	10.1080/09603409.2017.1393145	10.1002/srin.201800053
10.1007/bf01730060	10.1080/09603409.2017.1396650	10.1002/srin.201800208
10.1007/bf01730459	10.1080/09603409.2018.1429042	10.1002/xrs.130008020
10.1007/bf01742231	10.1080/09603409.2018.1435203	10.1002/xrs.130015041
10.1007/bf01748098	10.1080/09603409.2018.1448529	10.1002/zamm.201800342
10.1007/bf01755912	10.1080/09603409.2018.1456508	10.1111/ffe.12065
10.1007/bf01755916	10.1080/09603409.2018.1465712	10.1111/ffe.12069
10.1007/bf01855598	10.1080/09603409.2018.1466500	10.1111/ffe.12166
10.1007/bf01855601	10.1080/09603409.2018.1476808	10.1111/ffe.12168
10.1007/bf02134783	10.1080/09603409.2018.1482077	10.1111/ffe.12169
10.1007/bf02134784	10.1080/09603409.2018.1503444	10.1111/ffe.12218
10.1007/bf02153462	10.1080/09603409.2018.1513675	10.1111/ffe.12236
10.1007/bf02206238	10.1080/09603409.2018.1548682	10.1111/ffe.12261
10.1007/bf02209391	10.1080/09603409.2018.1556435	10.1111/ffe.12273
10.1007/bf02209477	10.1080/09603409.2019.1591064	10.1111/ffe.12286
10.1007/bf02320412	10.1080/09603409.2019.1631587	10.1111/ffe.12295
10.1007/bf02321420	10.1080/09603409.2019.1642556	10.1111/ffe.12330
10.1007/bf02322696	10.1080/09603409.2020.1718332	10.1111/ffe.12391
10.1007/bf02322827	10.1080/10402000008982337	10.1111/ffe.12396
10.1007/bf02323109	10.1080/10402000008982347	10.1111/ffe.12406
10.1007/bf02324983	10.1080/10402000008982348	10.1111/ffe.12414
10.1007/bf02325713	10.1080/10402000008982407	10.1111/ffe.12430
10.1007/bf02327781	10.1080/10402000208982540	10.1111/ffe.12465
10.1007/bf02328588	10.1080/10402004.2013.820372	10.1111/ffe.12475
10.1007/bf02385733	10.1080/10402004.2014.927547	10.1111/ffe.12551
10.1007/bf02396781	10.1080/10402004.2014.996308	10.1111/ffe.12584
10.1007/bf02397060	10.1080/10402004.2015.1131350	10.1111/ffe.12594
10.1007/bf02402788	10.1080/10402004.2016.1231357	10.1111/ffe.12615
10.1007/bf02402854	10.1080/10402004.2018.1486494	10.1111/ffe.12623
10.1007/bf02466269	10.1080/10402009008981997	10.1111/ffe.12630
10.1007/bf02466270	10.1080/10402009708983642	10.1111/ffe.12670
10.1007/bf02466274	10.1080/10402009708983695	10.1111/ffe.12722
10.1007/bf02466275	10.1080/10402009908982226	10.1111/ffe.12739
10.1007/bf02466287	10.1080/104077801300004230	10.1111/ffe.12749
10.1007/bf02467285	10.1080/10407780290059440	10.1111/ffe.12759
10.1007/bf02467286	10.1080/10407782.2020.1713685	10.1111/ffe.12851
10.1007/bf02468315	10.1080/10408436.2011.613492	10.1111/ffe.12880
10.1007/bf02468316	10.1080/10408436.2016.1243090	10.1111/ffe.12907
10.1007/bf02468511	10.1080/10420150.2019.1596110	10.1111/ffe.12982
10.1007/bf02586097	10.1080/10426910008913019	10.1111/ffe.12989
10.1007/bf02586111	10.1080/10426910500411561	10.1111/ffe.13028
10.1007/bf02586125	10.1080/10426910500411652	10.1111/ffe.13049
10.1007/bf02586152	10.1080/10426910500471433	10.1111/ffe.13071
10.1007/bf02586170	10.1080/10426910701235900	10.1111/ffe.13098
10.1007/bf02586222	10.1080/10426910701451697	10.1111/ffe.13127
10.1007/bf02586227	10.1080/10426910802384953	10.1111/ffe.13169
10.1007/bf02595432	10.1080/10426910802714571	10.1111/ffe.13181

10.1007/bf02595452	10.1080/10426910902746820	10.1111/ijac.12055
10.1007/bf02595461	10.1080/10426910902769103	10.1111/ijac.12496
10.1007/bf02595629	10.1080/10426910902979439	10.1111/ijac.13063
10.1007/bf02595635	10.1080/10426914.2010.527415	10.1111/ijac.13072
10.1007/bf02595651	10.1080/10426914.2010.537420	10.1111/ijac.13216
10.1007/bf02595656	10.1080/10426914.2011.551910	10.1111/ijac.13268
10.1007/bf02601608	10.1080/10426914.2011.610088	10.1111/j.1151-2916.1982.tb10
10.1007/bf02608555	10.1080/10426914.2011.654158	10.1111/j.1151-2916.1986.tb07
10.1007/bf02628368	10.1080/10426914.2012.689456	10.1111/j.1151-2916.1994.tb07
10.1007/bf02628369	10.1080/10426914.2012.718469	10.1111/j.1151-2916.1995.tb08
10.1007/bf02628370	10.1080/10426914.2012.727122	10.1111/j.1151-2916.2001.tb00
10.1007/bf02642331	10.1080/10426914.2013.792414	10.1111/j.1151-2916.2002.tb00
10.1007/bf02642410	10.1080/10426914.2013.822985	10.1111/j.1151-2916.2002.tb00
10.1007/bf02642413	10.1080/10426914.2013.852213	10.1111/j.1151-2916.2003.tb03
10.1007/bf02642414	10.1080/10426914.2013.864397	10.1111/j.1365-2818.1983.tb04
10.1007/bf02642425	10.1080/10426914.2013.864413	10.1111/j.1365-2818.1987.tb02
10.1007/bf02642426	10.1080/10426914.2014.892977	10.1111/j.1365-2818.1993.tb03
10.1007/bf02642576	10.1080/10426914.2014.901530	10.1111/j.1365-2818.1995.tb03
10.1007/bf02642841	10.1080/10426914.2014.901536	10.1111/j.1365-2818.2006.0163
10.1007/bf02642884	10.1080/10426914.2014.912313	10.1111/j.1365-2818.2006.0164
10.1007/bf02642885	10.1080/10426914.2014.912319	10.1111/j.1365-2818.2006.0165
10.1007/bf02642887	10.1080/10426914.2014.921694	10.1111/j.1365-2818.2007.0171
10.1007/bf02643103	10.1080/10426914.2014.921701	10.1111/j.1365-2818.2007.0181
10.1007/bf02643227	10.1080/10426914.2014.930897	10.1111/j.1365-2818.2007.0183
10.1007/bf02643237	10.1080/10426914.2014.952025	10.1111/j.1365-2818.2009.0313
10.1007/bf02643239	10.1080/10426914.2014.973575	10.1111/j.1365-2818.2009.0313
10.1007/bf02643343	10.1080/10426914.2014.973579	10.1111/j.1365-2818.2009.0313
10.1007/bf02643356	10.1080/10426914.2014.973588	10.1111/j.1365-2818.2009.0328
10.1007/bf02643391	10.1080/10426914.2014.973599	10.1111/j.1365-2818.2012.0364
10.1007/bf02643399	10.1080/10426914.2014.984203	10.1111/j.1460-2695.1979.tb01
10.1007/bf02643406	10.1080/10426914.2014.984216	10.1111/j.1460-2695.1979.tb01
10.1007/bf02643484	10.1080/10426914.2014.994766	10.1111/j.1460-2695.1979.tb01
10.1007/bf02643571	10.1080/10426914.2014.994769	10.1111/j.1460-2695.1980.tb01
10.1007/bf02643573	10.1080/10426914.2014.994770	10.1111/j.1460-2695.1980.tb01
10.1007/bf02643739	10.1080/10426914.2015.1019090	10.1111/j.1460-2695.1981.tb01
10.1007/bf02643746	10.1080/10426914.2015.1019091	10.1111/j.1460-2695.1981.tb01
10.1007/bf02643747	10.1080/10426914.2015.1019092	10.1111/j.1460-2695.1981.tb01
10.1007/bf02643777	10.1080/10426914.2015.1037906	10.1111/j.1460-2695.1982.tb01
10.1007/bf02643789	10.1080/10426914.2015.1037913	10.1111/j.1460-2695.1983.tb01
10.1007/bf02643790	10.1080/10426914.2015.1048462	10.1111/j.1460-2695.1985.tb00
10.1007/bf00542087	10.1080/01418618908209821	10.1002/adem.201600529
10.1007/bf00542317	10.1080/01418619008243921	10.1002/adem.201600535
10.1007/bf00542381	10.1080/01418619008244336	10.1002/adem.201600635
10.1007/bf00542912	10.1080/01418619108204866	10.1002/adem.201600672
10.1007/bf00543618	10.1080/01418619108204867	10.1002/adem.201600690
10.1007/bf00544162	10.1080/01418619108204868	10.1002/adem.201600721
10.1007/bf00544198	10.1080/01418619108205586	10.1002/adem.201700041
10.1007/bf00544199	10.1080/01418619208201587	10.1002/adem.201700150
10.1007/bf00544533	10.1080/01418619208205607	10.1002/adem.201700182
10.1007/bf00545469	10.1080/01418619208205609	10.1002/adem.201700297
10.1007/bf00545480	10.1080/01418619208205611	10.1002/adem.201700502
10.1007/bf00549796	10.1080/01418619308219366	10.1002/adem.201700568
10.1007/bf00550537	10.1080/01418619308222923	10.1002/adem.201700645
10.1007/bf00551018	10.1080/01418619408242213	10.1002/adem.201700820

10.1007/bf00551281	10.1080/01418619508236228	10.1002/adem.201700930
10.1007/bf00551321	10.1080/01418619508236233	10.1002/adem.201700960
10.1007/bf00551462	10.1080/01418619508236248	10.1002/adem.201700973
10.1007/bf00551493	10.1080/01418619508239932	10.1002/adem.201700987
10.1007/bf00551505	10.1080/01418619508239952	10.1002/adem.201701011
10.1007/bf00551985	10.1080/01418619508243792	10.1002/adem.201701043
10.1007/bf00552407	10.1080/01418619608239688	10.1002/adem.201701114
10.1007/bf00553215	10.1080/01418619608239699	10.1002/adem.201701189
10.1007/bf00553420	10.1080/01418619708210282	10.1002/adem.201800129
10.1007/bf00553814	10.1080/01418619708214021	10.1002/adem.201800234
10.1007/bf00554772	10.1080/01418619908210378	10.1002/adem.201800279
10.1007/bf00556076	10.1080/01418619908212011	10.1002/adem.201800307
10.1007/bf00557126	10.1080/01418619908212034	10.1002/adem.201800351
10.1007/bf00560640	10.1080/01495728108961787	10.1002/adem.201800647
10.1007/bf00570396	10.1080/01495739.2017.1352464	10.1002/adem.201800652
10.1007/bf00570398	10.1080/014957390523651	10.1002/adem.201800793
10.1007/bf00576274	10.1080/01495739408946255	10.1002/adem.201800856
10.1007/bf00576299	10.1080/01694243.2016.1146394	10.1002/adem.201800933
10.1007/bf00576301	10.1080/01694243.2017.1310173	10.1002/adem.201801022
10.1007/bf00581093	10.1080/01694243.2019.1620425	10.1002/adem.201801318
10.1007/bf00582481	10.1080/01932691.2019.1649155	10.1002/adem.201801354
10.1007/bf00587693	10.1080/02533839.2007.9671248	10.1002/adem.201900054
10.1007/bf00595740	10.1080/02533839.2010.9671681	10.1002/adem.201900267
10.1007/bf00603531	10.1080/02533839.2012.701897	10.1002/adem.201900426
10.1007/bf00603578	10.1080/02619180.1985.11753277	10.1002/adem.201900499
10.1007/bf00603610	10.1080/02619180.1985.11753278	10.1002/adem.201900543
10.1007/bf00605108	10.1080/02619180.1986.11753313	10.1002/adem.201900558
10.1007/bf00606191	10.1080/02619180.1986.11753319	10.1002/adem.201900641
10.1007/bf00608013	10.1080/02619180.1986.11753320	10.1002/adem.201900823
10.1007/bf00610640	10.1080/02619180.1987.11753379	10.1002/adem.201900892
10.1007/bf00611474	10.1080/02619180.1987.11753401	10.1002/adem.201900930
10.1007/bf00611601	10.1080/02619180.1988.11753371	10.1002/adem.201901151
10.1007/bf00625005	10.1080/02619180.1988.11753384	10.1002/adem.201901228
10.1007/bf00638021	10.1080/02619180.1988.11753389	10.1002/adem.201901237
10.1007/bf00644668	10.1080/02619180.1988.11753391	10.1002/adem.201901244
10.1007/bf00644688	10.1080/02619180.1989.11753418	10.1002/adem.201901445
10.1007/bf00647782	10.1080/02619180.1989.11753426	10.1002/adma.19940060
10.1007/bf00655894	10.1080/02619180.1989.11753437	10.1002/adma.19960080
10.1007/bf00655896	10.1080/02619180.1990.11753477	10.1002/adma.200702461
10.1007/bf00656576	10.1080/02619180.1990.11753492	10.1002/adma.201003600
10.1007/bf00656643	10.1080/02619180.1990.11753493	10.1002/adma.201200764
10.1007/bf00656646	10.1080/02670836.1987.11782259	10.1002/adma.201907164
10.1007/bf00656727	10.1080/02670836.1987.11782262	10.1002/aic.16384
10.1007/bf00656844	10.1080/02670836.1987.11782264	10.1002/bbpc.19992040
10.1007/bf00656898	10.1080/02670836.1987.11782265	10.1002/bbpc.20003060
10.1007/bf00656900	10.1080/02670836.1987.11782266	10.1002/ceat.201700681
10.1007/bf00659249	10.1080/02670836.1987.11782269	10.1002/chem.201703381
10.1007/bf00662037	10.1080/02670836.1987.11782270	10.1002/chem.201704531
10.1007/bf00664272	10.1080/02670836.1996.11665716	10.1002/cite.201300192
10.1007/bf00664273	10.1080/02670836.2016.1139225	10.1002/cjce.23020
10.1007/bf00664277	10.1080/02670836.2016.1148227	10.1002/cnm.753
10.1007/bf00664423	10.1080/02670836.2016.1149277	10.1002/crat.19780131
10.1007/bf00664494	10.1080/02670836.2016.1159002	10.1002/crat.201000389
10.1007/bf00664496	10.1080/02670836.2016.1187335	10.1002/crat.201100391

10.1007/bf00664662	10.1080/02670836.2016.1204070	10.1002/crat.201600372
10.1007/bf00664711	10.1080/02670836.2016.1215961	10.1002/crat.201700012
10.1007/bf00664797	10.1080/02670836.2016.1216029	10.1002/crat.201700187
10.1007/bf00664803	10.1080/02670836.2016.1230168	10.1002/crat.201800108
10.1007/bf00665022	10.1080/02670836.2016.1242827	10.1002/crat.201800177
10.1007/bf00665047	10.1080/02670836.2016.1273866	10.1002/cvde.201307167
10.1007/bf00665258	10.1080/02670836.2017.1282668	10.1002/cvde.201307244
10.1007/bf00665259	10.1080/02670836.2017.1288674	10.1002/er.1458
10.1007/bf00665269	10.1080/02670836.2017.1295212	10.1002/fam.907
10.1007/bf00665447	10.1080/02670836.2017.1300365	10.1002/htj.10089
10.1007/bf00665490	10.1080/02670836.2017.1300419	10.1002/jbm.820220707
10.1007/bf00665491	10.1080/02670836.2017.1333222	10.1002/jbm.b.30586
10.1007/bf00665612	10.1080/02670836.2017.1337299	10.1002/jemt.10600704
10.1007/bf00665614	10.1080/02670836.2017.1345823	10.1002/jemt.10601102
10.1007/bf00665662	10.1080/02670836.2017.1353662	10.1002/jemt.22359
10.1007/bf00665670	10.1080/02670836.2017.1389118	10.1002/jemt.23417
10.1007/bf00665673	10.1080/02670836.2017.1393977	10.1002/ls.1255
10.1007/bf00666595	10.1080/02670836.2017.1407566	10.1002/maco.19840350
10.1007/bf00666598	10.1080/02670836.2017.1410929	10.1002/maco.19900410
10.1007/bf00666599	10.1080/02670836.2017.1410953	10.1002/maco.19920430
10.1007/bf00666645	10.1080/02670836.2017.1419612	10.1002/maco.19950460
10.1007/bf00666806	10.1080/02670836.2018.1424379	10.1002/maco.19950461
10.1007/bf00666913	10.1080/02670836.2018.1428405	10.1002/maco.19960471
10.1007/bf00667421	10.1080/02670836.2018.1429043	10.1002/maco.200303709
10.1007/bf00687309	10.1080/02670836.2018.1461594	10.1002/maco.200303728

10.1007/bf02647085	10.1080/13621718.2019.1600822
10.1007/bf02647181	10.1080/13621718.2019.1608406
10.1007/bf02647183	10.1080/13640461.2000.11819381
10.1007/bf02647238	10.1080/13640461.2000.11819391
10.1007/bf02647239	10.1080/13640461.2001.11819428
10.1007/bf02647269	10.1080/13640461.2016.1166726
10.1007/bf02647272	10.1080/13640461.2016.1182785
10.1007/bf02647308	10.1080/13640461.2016.1261519
10.1007/bf02647316	10.1080/13640461.2019.1674476
10.1007/bf02647405	10.1080/13642819108207609
10.1007/bf02647413	10.1080/13642819108207610
10.1007/bf02647500	10.1080/13642819308207693
10.1007/bf02647521	10.1080/14328917.2016.1141086
10.1007/bf02647549	10.1080/14686996.2017.1312519
10.1007/bf02647605	10.1080/14686996.2017.1371559
10.1007/bf02647723	10.1080/1478422x.2015.1104066
10.1007/bf02647750	10.1080/1478422x.2016.1158226
10.1007/bf02647755	10.1080/1478422x.2016.1263456
10.1007/bf02647821	10.1080/1478422x.2017.1350327
10.1007/bf02647830	10.1080/1478422x.2018.1473318
10.1007/bf02647831	10.1080/1478422x.2018.1511314
10.1007/bf02647832	10.1080/1478422x.2018.1519102
10.1007/bf02647833	10.1080/1478422x.2018.1525829
10.1007/bf02647868	10.1080/1478422x.2019.1699261
10.1007/bf02647878	10.1080/1478643031000094749
10.1007/bf02647881	10.1080/1478643031000149108
10.1007/bf02647974	10.1080/14786430310001603445
10.1007/bf02647976	10.1080/14786430310001605407
10.1007/bf02648342	10.1080/14786430310001621472
10.1007/bf02648344	10.1080/14786430310001646718
10.1007/bf02648351	10.1080/14786430410001663240
10.1007/bf02648404	10.1080/14786430412331283064
10.1007/bf02648410	10.1080/14786430412331284432
10.1007/bf02648412	10.1080/14786430412331309344
10.1007/bf02648413	10.1080/14786430500254685
10.1007/bf02648422	10.1080/14786430600740658
10.1007/bf02648432	10.1080/14786430600767750
10.1007/bf02648514	10.1080/14786430600796551
10.1007/bf02648518	10.1080/14786430601032360
10.1007/bf02648539	10.1080/14786430701203184
10.1007/bf02648542	10.1080/14786430701393159
10.1007/bf02648545	10.1080/14786430701541120
10.1007/bf02648573	10.1080/14786430802243857
10.1007/bf02648962	10.1080/14786430802337089
10.1007/bf02648966	10.1080/14786430902877810
10.1007/bf02649087	10.1080/14786430903140747
10.1007/bf02649089	10.1080/14786435.2010.521527
10.1007/bf02649091	10.1080/14786435.2011.580287
10.1007/bf02649236	10.1080/14786435.2011.586158
10.1007/bf02649259	10.1080/14786435.2011.607139
10.1007/bf02649262	10.1080/14786435.2011.609151
10.1007/bf02649282	10.1080/14786435.2011.630691
10.1007/bf02649474	10.1080/14786435.2011.634853
10.1007/bf02649475	10.1080/14786435.2011.648227

10.1007/bf02649635	10.1080/14786435.2011.652690
10.1007/bf02649637	10.1080/14786435.2012.669073
10.1007/bf02649675	10.1080/14786435.2012.676212
10.1007/bf02649740	10.1080/14786435.2012.700416
10.1007/bf02649750	10.1080/14786435.2012.709949
10.1007/bf02649752	10.1080/14786435.2012.746794
10.1007/bf02649755	10.1080/14786435.2013.765989
10.1007/bf02649768	10.1080/14786435.2013.773106
10.1007/bf02649770	10.1080/14786435.2013.778427
10.1007/bf02649777	10.1080/14786435.2013.852287
10.1007/bf02649806	10.1080/14786435.2013.878052
10.1007/bf02649876	10.1080/14786435.2014.885136
10.1007/bf02650035	10.1080/14786435.2014.903342
10.1007/bf02650066	10.1080/14786435.2014.906756
10.1007/bf02650094	10.1080/14786435.2014.914261
10.1007/bf02650143	10.1080/14786435.2014.956838
10.1007/bf02650292	10.1080/14786435.2015.1010622
10.1007/bf02650300	10.1080/14786435.2015.1049236
10.1007/bf02651595	10.1080/14786435.2015.1073858
10.1007/bf02651604	10.1080/14786435.2015.1109152
10.1007/bf02651605	10.1080/14786435.2015.1132017
10.1007/bf02651607	10.1080/14786435.2016.1143127
10.1007/bf02651609	10.1080/14786435.2016.1192298
10.1007/bf02651614	10.1080/14786435.2016.1215607
10.1007/bf02651618	10.1080/14786435.2016.1243266
10.1007/bf02651630	10.1080/14786435.2017.1344787
10.1007/bf02651730	10.1080/14786435.2017.1407880
10.1007/bf02651762	10.1080/14786435.2018.1447159
10.1007/bf02652249	10.1080/14786435.2018.1493236
10.1007/bf02652257	10.1080/14786435.2018.1510189
10.1007/bf02652327	10.1080/14786435.2018.1520403
10.1007/bf02652334	10.1080/14786435.2018.1524161
10.1007/bf02652539	10.1080/14786435.2018.1539263
10.1007/bf02652857	10.1080/14786435.2018.1540891
10.1007/bf02653502	10.1080/14786435.2019.1576936
10.1007/bf02654381	10.1080/14786435.2019.1582850
10.1007/bf02654423	10.1080/14786435.2019.1696023
10.1007/bf02654499	10.1080/14786435.2020.1714089
10.1007/bf02654502	10.1080/15376490306735
10.1007/bf02654504	10.1080/15376494.2018.1444221
10.1007/bf02654523	10.1080/17515831.2016.1146476
10.1007/bf02654525	10.1080/18811248.2000.9714977
10.1007/bf02654573	10.1080/21663831.2015.1045630
10.1007/bf02654702	10.1080/21663831.2018.1538021
10.1007/bf02654716	10.1080/21663831.2019.1568315
10.1007/bf02654718	10.1080/23311916.2018.1501864

10.1007/bf02655082
10.1007/bf02655105
10.1007/bf02655117
10.1007/bf02655188
10.1007/bf02655191
10.1007/bf02655192
10.1007/bf02655199
10.1007/bf02655203
10.1007/bf02655208
10.1007/bf02656435
10.1007/bf02656499
10.1007/bf02656579
10.1007/bf02656590
10.1007/bf02656598
10.1007/bf02656604
10.1007/bf02656614
10.1007/bf02656637
10.1007/bf02656645
10.1007/bf02656706
10.1007/bf02656707
10.1007/bf02656808
10.1007/bf02656815
10.1007/bf02656817
10.1007/bf02656819
10.1007/bf02656820
10.1007/bf02656823
10.1007/bf02657235
10.1007/bf02657248
10.1007/bf02657253
10.1007/bf02657334
10.1007/bf02657848
10.1007/bf02658061
10.1007/bf02658069
10.1007/bf02658258
10.1007/bf02658304
10.1007/bf02658320
10.1007/bf02658331
10.1007/bf02658388
10.1007/bf02658409
10.1007/bf02658447
10.1007/bf02658676
10.1007/bf02658678
10.1007/bf02658683
10.1007/bf02658808
10.1007/bf02658829
10.1007/bf02658993
10.1007/bf02658999
10.1007/bf02659922
10.1007/bf02660519
10.1007/bf02660534
10.1007/bf02660636
10.1007/bf02660676
10.1007/bf02660857
10.1007/bf02660868

10.1007/bf02661276
10.1007/bf02661340
10.1007/bf02661638
10.1007/bf02661730
10.1007/bf02661731
10.1007/bf02661732
10.1007/bf02661734
10.1007/bf02661735
10.1007/bf02661736
10.1007/bf02661737
10.1007/bf02661738
10.1007/bf02661739
10.1007/bf02661740
10.1007/bf02661741
10.1007/bf02661745
10.1007/bf02662367
10.1007/bf02662397
10.1007/bf02662398
10.1007/bf02662399
10.1007/bf02662400
10.1007/bf02662579
10.1007/bf02662588
10.1007/bf02662590
10.1007/bf02663024
10.1007/bf02663204
10.1007/bf02663221
10.1007/bf02663305
10.1007/bf02663407
10.1007/bf02663428
10.1007/bf02663432
10.1007/bf02663435
10.1007/bf02663438
10.1007/bf02663837
10.1007/bf02663910
10.1007/bf02663917
10.1007/bf02664679
10.1007/bf02664680
10.1007/bf02664810
10.1007/bf02664827
10.1007/bf02664831
10.1007/bf02664839
10.1007/bf02664997
10.1007/bf02665056
10.1007/bf02665063
10.1007/bf02665403
10.1007/bf02665423
10.1007/bf02665447
10.1007/bf02665510
10.1007/bf02665511
10.1007/bf02665823
10.1007/bf02666282
10.1007/bf02666333
10.1007/bf02666340
10.1007/bf02666344

10.1007/bf02666354
10.1007/bf02666358
10.1007/bf02666359
10.1007/bf02666360
10.1007/bf02666662
10.1007/bf02667291
10.1007/bf02667378
10.1007/bf02667513
10.1007/bf02667583
10.1007/bf02667584
10.1007/bf02667585
10.1007/bf02668209
10.1007/bf02668534
10.1007/bf02668538
10.1007/bf02669400
10.1007/bf02669422
10.1007/bf02669450
10.1007/bf02669816
10.1007/bf02669818
10.1007/bf02669867
10.1007/bf02669870
10.1007/bf02670146
10.1007/bf02670181
10.1007/bf02670251
10.1007/bf02670252
10.1007/bf02670296
10.1007/bf02670326
10.1007/bf02670333
10.1007/bf02670334
10.1007/bf02670335
10.1007/bf02670358
10.1007/bf02670365
10.1007/bf02670372
10.1007/bf02670373
10.1007/bf02670621
10.1007/bf02670678
10.1007/bf02670767
10.1007/bf02670769
10.1007/bf02671240
10.1007/bf02671927
10.1007/bf02672297
10.1007/bf02672298
10.1007/bf02672577
10.1007/bf02672587
10.1007/bf02673686
10.1007/bf02673690
10.1007/bf02675552
10.1007/bf02675570
10.1007/bf02675578
10.1007/bf02676987
10.1007/bf02682684
10.1007/bf02682685
10.1007/bf02696935
10.1007/bf02698254

10.1007/bf02698605
10.1007/bf02701074
10.1007/bf02704253
10.1007/bf02710102
10.1007/bf02717129
10.1007/bf02717130
10.1007/bf02717131
10.1007/bf02744633
10.1007/bf02748493
10.1007/bf02757549
10.1007/bf02767571
10.1007/bf02782415
10.1007/bf02782416
10.1007/bf02782418
10.1007/bf02782419
10.1007/bf02782428
10.1007/bf02801169
10.1007/bf02801173
10.1007/bf02804368
10.1007/bf02811669
10.1007/bf02811674
10.1007/bf02811675
10.1007/bf02811680
10.1007/bf02811710
10.1007/bf02811985
10.1007/bf02811986
10.1007/bf02812013
10.1007/bf02814225
10.1007/bf02814240
10.1007/bf02814341
10.1007/bf02814828
10.1007/bf02814832
10.1007/bf02814833
10.1007/bf02817272
10.1007/bf02817889
10.1007/bf02818376
10.1007/bf02818377
10.1007/bf02822603
10.1007/bf02822605
10.1007/bf02830350
10.1007/bf02830351
10.1007/bf02830355
10.1007/bf02834122
10.1007/bf02834138
10.1007/bf02834497
10.1007/bf02851353
10.1007/bf02882642
10.1007/bf02914402
10.1007/bf02914651
10.1007/bf02914652
10.1007/bf02914765
10.1007/bf02914771
10.1007/bf02945937
10.1007/bf02984447

10.1007/bf03026343
10.1007/bf03027051
10.1007/bf03027224
10.1007/bf03027245
10.1007/bf03027472
10.1007/bf03027515
10.1007/bf03027824
10.1007/bf03161838
10.1007/bf03161839
10.1007/bf03186114
10.1007/bf03220467
10.1007/bf03222914
10.1007/bf03222941
10.1007/bf03222995
10.1007/bf03223247
10.1007/bf03257838
10.1007/bf03258034
10.1007/bf03258035
10.1007/bf03258604
10.1007/bf03258605
10.1007/bf03258708
10.1007/bf03258745
10.1007/bf03258764
10.1007/bf03259691
10.1007/bf03259692
10.1007/bf03338341
10.1007/bf03339508
10.1007/bf03339911
10.1007/bf03344917
10.1007/bf03354338
10.1007/bf03354420
10.1007/bf03354535
10.1007/bf03355529
10.1007/s00161-013-0317-6
10.1007/s00161-015-0471-0
10.1007/s00161-016-0533-y
10.1007/s00161-017-0608-4
10.1007/s00161-018-0720-0
10.1007/s00161-018-0721-z
10.1007/s001610050137
10.1007/s00164-002-0001-x
10.1007/s00170-004-2257-6
10.1007/s00170-004-2442-7
10.1007/s00170-005-0402-5
10.1007/s00170-006-0572-9
10.1007/s00170-006-0657-5
10.1007/s00170-007-1084-y
10.1007/s00170-008-1561-y
10.1007/s00170-008-1789-6
10.1007/s00170-008-1821-x
10.1007/s00170-008-1913-7
10.1007/s00170-009-1987-x
10.1007/s00170-010-2538-1
10.1007/s00170-010-2581-y

10.1007/s00170-010-2590-x
10.1007/s00170-010-2643-1
10.1007/s00170-010-2703-6
10.1007/s00170-010-2777-1
10.1007/s00170-010-2945-3
10.1007/s00170-010-3036-1
10.1007/s00170-010-3062-z
10.1007/s00170-011-3196-7
10.1007/s00170-011-3423-2
10.1007/s00170-011-3455-7
10.1007/s00170-011-3497-x
10.1007/s00170-011-3529-6
10.1007/s00170-011-3679-6
10.1007/s00170-011-3714-7
10.1007/s00170-011-3812-6
10.1007/s00170-011-3830-4
10.1007/s00170-012-3907-8
10.1007/s00170-012-4012-8
10.1007/s00170-012-4236-7
10.1007/s00170-012-4385-8
10.1007/s00170-012-4477-5
10.1007/s00170-012-4548-7
10.1007/s00170-012-4567-4
10.1007/s00170-012-4584-3
10.1007/s00170-013-4745-z
10.1007/s00170-013-4823-2
10.1007/s00170-013-4899-8
10.1007/s00170-013-4917-x
10.1007/s00170-013-4944-7
10.1007/s00170-013-4979-9
10.1007/s00170-013-4981-2
10.1007/s00170-013-5049-z
10.1007/s00170-013-5171-y
10.1007/s00170-013-5198-0
10.1007/s00170-013-5225-1
10.1007/s00170-013-5361-7
10.1007/s00170-013-5408-9
10.1007/s00170-013-5441-8
10.1007/s00170-013-5471-2
10.1007/s00170-013-5528-2
10.1007/s00170-013-5557-x
10.1007/s00170-013-5569-6
10.1007/s00170-014-5764-0
10.1007/s00170-014-5911-7
10.1007/s00170-014-6047-5
10.1007/s00170-014-6115-x
10.1007/s00170-014-6126-7
10.1007/s00170-014-6279-4
10.1007/s00170-014-6369-3
10.1007/s00170-014-6403-5
10.1007/s00170-014-6433-z
10.1007/s00170-014-6588-7
10.1007/s00170-014-6606-9
10.1007/s00170-015-6797-8

10.1007/s00170-015-6849-0
10.1007/s00170-015-6891-y
10.1007/s00170-015-6904-x
10.1007/s00170-015-6909-5
10.1007/s00170-015-7013-6
10.1007/s00170-015-7125-z
10.1007/s00170-015-7153-8
10.1007/s00170-015-7257-1
10.1007/s00170-015-7265-1
10.1007/s00170-015-7270-4
10.1007/s00170-015-7310-0
10.1007/s00170-015-7356-z
10.1007/s00170-015-7414-6
10.1007/s00170-015-7481-8
10.1007/s00170-015-7519-y
10.1007/s00170-015-7550-z
10.1007/s00170-015-7575-3
10.1007/s00170-015-7603-3
10.1007/s00170-015-7658-1
10.1007/s00170-015-7685-y
10.1007/s00170-015-7697-7
10.1007/s00170-015-7699-5
10.1007/s00170-015-7732-8
10.1007/s00170-015-8006-1
10.1007/s00170-015-8056-4
10.1007/s00170-015-8085-z
10.1007/s00170-015-8121-z
10.1007/s00170-015-8171-2
10.1007/s00170-015-8203-y
10.1007/s00170-015-8318-1
10.1007/s00170-015-8323-4
10.1007/s00170-016-8342-9
10.1007/s00170-016-8350-9
10.1007/s00170-016-8352-7
10.1007/s00170-016-8360-7
10.1007/s00170-016-8402-1
10.1007/s00170-016-8405-y
10.1007/s00170-016-8438-2
10.1007/s00170-016-8568-6
10.1007/s00170-016-8642-0
10.1007/s00170-016-8668-3
10.1007/s00170-016-8686-1
10.1007/s00170-016-8796-9
10.1007/s00170-016-8884-x
10.1007/s00170-016-8966-9
10.1007/s00170-016-8998-1
10.1007/s00170-016-9030-5
10.1007/s00170-016-9037-y
10.1007/s00170-016-9091-5
10.1007/s00170-016-9192-1
10.1007/s00170-016-9196-x
10.1007/s00170-016-9271-3
10.1007/s00170-016-9332-7
10.1007/s00170-016-9401-y

10.1007/s00170-016-9415-5
10.1007/s00170-016-9429-z
10.1007/s00170-016-9485-4
10.1007/s00170-016-9515-2
10.1007/s00170-016-9717-7
10.1007/s00170-016-9728-4
10.1007/s00170-016-9737-3
10.1007/s00170-016-9795-6
10.1007/s00170-016-9897-1
10.1007/s00170-016-9901-9
10.1007/s00170-016-9907-3
10.1007/s00170-017-0001-2
10.1007/s00170-017-0079-6
10.1007/s00170-017-0080-0
10.1007/s00170-017-0084-9
10.1007/s00170-017-0266-5
10.1007/s00170-017-0280-7
10.1007/s00170-017-0298-x
10.1007/s00170-017-0316-z
10.1007/s00170-017-0329-7
10.1007/s00170-017-0372-4
10.1007/s00170-017-0512-x
10.1007/s00170-017-0654-x
10.1007/s00170-017-0680-8
10.1007/s00170-017-0704-4
10.1007/s00170-017-0727-x
10.1007/s00170-017-0758-3
10.1007/s00170-017-0773-4
10.1007/s00170-017-0775-2
10.1007/s00170-017-0787-y
10.1007/s00170-017-0806-z
10.1007/s00170-017-0855-3
10.1007/s00170-017-0873-1
10.1007/s00170-017-0875-z
10.1007/s00170-017-0899-4
10.1007/s00170-017-0984-8
10.1007/s00170-017-0999-1
10.1007/s00170-017-1001-y
10.1007/s00170-017-1005-7
10.1007/s00170-017-1014-6
10.1007/s00170-017-1019-1
10.1007/s00170-017-1090-7
10.1007/s00170-017-1100-9
10.1007/s00170-017-1133-0
10.1007/s00170-017-1159-3
10.1007/s00170-017-1162-8
10.1007/s00170-017-1224-y
10.1007/s00170-017-1333-7
10.1007/s00170-017-1339-1
10.1007/s00170-017-1407-6
10.1007/s00170-017-1464-x
10.1007/s00170-017-1481-9
10.1007/s00170-017-1489-1
10.1007/s00170-017-1522-4

10.1007/s00170-017-1549-6
10.1007/s00170-017-9989-6
10.1007/s00170-017-9993-x
10.1007/s00170-018-03211-x
10.1007/s00170-018-03245-1
10.1007/s00170-018-03280-y
10.1007/s00170-018-1581-1
10.1007/s00170-018-1592-y
10.1007/s00170-018-1721-7
10.1007/s00170-018-1773-8
10.1007/s00170-018-1804-5
10.1007/s00170-018-1809-0
10.1007/s00170-018-1842-z
10.1007/s00170-018-1849-5
10.1007/s00170-018-1902-4
10.1007/s00170-018-1911-3
10.1007/s00170-018-1931-z
10.1007/s00170-018-1993-y
10.1007/s00170-018-2014-x
10.1007/s00170-018-2032-8
10.1007/s00170-018-2043-5
10.1007/s00170-018-2105-8
10.1007/s00170-018-2134-3
10.1007/s00170-018-2169-5
10.1007/s00170-018-2196-2
10.1007/s00170-018-2270-9
10.1007/s00170-018-2371-5
10.1007/s00170-018-2484-x
10.1007/s00170-018-2588-3
10.1007/s00170-018-2616-3
10.1007/s00170-018-2686-2
10.1007/s00170-018-2697-z
10.1007/s00170-018-2704-4
10.1007/s00170-018-2713-3
10.1007/s00170-018-2746-7
10.1007/s00170-018-2792-1
10.1007/s00170-018-2807-y
10.1007/s00170-018-2839-3
10.1007/s00170-018-2846-4
10.1007/s00170-018-2850-8
10.1007/s00170-018-2883-z
10.1007/s00170-018-2935-4
10.1007/s00170-018-3032-4
10.1007/s00170-018-3075-6
10.1007/s00170-018-3117-0
10.1007/s00170-018-3166-4
10.1007/s00170-019-03389-8
10.1007/s00170-019-03414-w
10.1007/s00170-019-03453-3
10.1007/s00170-019-03456-0
10.1007/s00170-019-03481-z
10.1007/s00170-019-03492-w
10.1007/s00170-019-03503-w
10.1007/s00170-019-03545-0

10.1007/s00170-019-03555-y
10.1007/s00170-019-03591-8
10.1007/s00170-019-03611-7
10.1007/s00170-019-03616-2
10.1007/s00170-019-03645-x
10.1007/s00170-019-03695-1
10.1007/s00170-019-03698-y
10.1007/s00170-019-03716-z
10.1007/s00170-019-03767-2
10.1007/s00170-019-03828-6
10.1007/s00170-019-03931-8
10.1007/s00170-019-03949-y
10.1007/s00170-019-03983-w
10.1007/s00170-019-04024-2
10.1007/s00170-019-04117-y
10.1007/s00170-019-04126-x
10.1007/s00170-019-04187-y
10.1007/s00170-019-04199-8
10.1007/s00170-019-04210-2
10.1007/s00170-019-04250-8
10.1007/s00170-019-04290-0
10.1007/s00170-019-04328-3
10.1007/s00170-019-04330-9
10.1007/s00170-019-04501-8
10.1007/s00170-019-04541-0
10.1007/s00170-019-04616-y
10.1007/s00170-019-04632-y
10.1007/s00170-019-04694-y
10.1007/s00170-019-04719-6
10.1007/s00170-019-04769-w
10.1007/s00170-019-04793-w
10.1007/s00170-019-04805-9
10.1007/s00170-020-04931-9
10.1007/s00170-020-05081-8
10.1007/s001700200052
10.1007/s00205-007-0054-8
10.1007/s00216-002-1459-8
10.1007/s00216-010-4605-8
10.1007/s00216-011-4666-3
10.1007/s002160050362
10.1007/s002160050363
10.1007/s002160051464
10.1007/s00231-017-2105-5
10.1007/s00231-018-02551-4
10.1007/s00339-008-4417-2
10.1007/s00339-008-4885-4
10.1007/s00339-011-6302-7
10.1007/s00339-013-7968-9
10.1007/s00339-013-8046-z
10.1007/s00339-013-8062-z
10.1007/s00339-014-8604-z
10.1007/s00339-014-8898-x
10.1007/s00339-015-9148-6
10.1007/s00339-015-9290-1

10.1007/s00339-015-9291-0
10.1007/s00339-016-0129-1
10.1007/s00339-016-0333-z
10.1007/s00339-016-0371-6
10.1007/s00339-016-0385-0
10.1007/s00339-016-0404-1
10.1007/s00339-016-0683-6
10.1007/s00339-016-9599-4
10.1007/s00339-017-0814-8
10.1007/s00339-017-1004-4
10.1007/s00339-017-1208-7
10.1007/s00339-018-1737-8
10.1007/s00339-019-2741-3
10.1007/s00339-019-2745-z
10.1007/s00339-019-3019-5
10.1007/s00339-019-3084-9
10.1007/s003390050292
10.1007/s003390050778
10.1007/s003390051253
10.1007/s003390101165
10.1007/s003390101195
10.1007/s003390101208
10.1007/s003390201680
10.1007/s003390201681
10.1007/s003390201742
10.1007/s003390201870
10.1007/s00419-004-0320-2
10.1007/s00466-018-1598-1
10.1007/s00521-016-2188-9
10.1007/s00521-016-2556-5
10.1007/s00521-016-2581-4
10.1007/s00521-016-2635-7
10.1007/s00521-019-04450-z
10.1007/s00604-003-0144-8
10.1007/s006040170005
10.1007/s00707-013-0820-7
10.1007/s100190050120
10.1007/s100190050137
10.1007/s10035-020-0997-0
10.1007/s10311-015-0517-3
10.1007/s10338-008-0833-2
10.1007/s10409-015-0499-1
10.1007/s10409-019-00876-9
10.1007/s10704-005-3149-y
10.1007/s10704-006-0102-7
10.1007/s10704-008-9294-3
10.1007/s10704-012-9699-x
10.1007/s10704-013-9813-8
10.1007/s10704-013-9905-5
10.1007/s10704-015-0016-3
10.1007/s10704-017-0213-3
10.1007/s10704-017-0241-z
10.1007/s10704-018-0311-x
10.1007/s10704-019-00344-2

10.1007/s10704-020-00434-6
10.1007/s10765-004-7743-4
10.1007/s10765-006-0029-2
10.1007/s10765-007-0163-5
10.1007/s10765-007-0257-0
10.1007/s10765-009-0604-4
10.1007/s10765-010-0848-z
10.1007/s10765-014-1706-1
10.1007/s10765-015-1834-2
10.1007/s10765-017-2242-6
10.1007/s10765-017-2267-x
10.1007/s10765-018-2388-x
10.1007/s10765-018-2390-3
10.1007/s10765-019-2490-8
10.1007/s10800-008-9516-4
10.1007/s10800-017-1090-1
10.1007/s10800-017-1096-8
10.1007/s10800-019-01386-z
10.1007/s10845-014-0942-3
10.1007/s10845-016-1225-y
10.1007/s10853-005-1217-3
10.1007/s10853-005-1294-3
10.1007/s10853-005-1548-0
10.1007/s10853-005-1930-y
10.1007/s10853-005-2418-5
10.1007/s10853-005-2848-0
10.1007/s10853-005-3154-6
10.1007/s10853-005-6501-8
10.1007/s10853-005-6503-6
10.1007/s10853-005-6938-9
10.1007/s10853-005-6940-2
10.1007/s10853-005-6941-1
10.1007/s10853-005-6942-0
10.1007/s10853-006-0166-9
10.1007/s10853-006-0168-7
10.1007/s10853-006-0483-z
10.1007/s10853-006-0806-0
10.1007/s10853-006-0948-0
10.1007/s10853-006-1033-4
10.1007/s10853-007-1615-9
10.1007/s10853-007-1645-3
10.1007/s10853-007-1661-3
10.1007/s10853-007-1922-1
10.1007/s10853-007-1929-7
10.1007/s10853-007-2001-3
10.1007/s10853-007-2130-8
10.1007/s10853-007-2206-5
10.1007/s10853-007-2342-y
10.1007/s10853-007-2363-6
10.1007/s10853-008-2575-4
10.1007/s10853-008-2604-3
10.1007/s10853-008-2666-2
10.1007/s10853-008-2781-0
10.1007/s10853-008-2783-y

10.1007/s10853-008-2866-9
10.1007/s10853-008-3025-z
10.1007/s10853-008-3084-1
10.1007/s10853-008-3108-x
10.1007/s10853-008-3221-x
10.1007/s10853-009-3251-z
10.1007/s10853-009-3391-1
10.1007/s10853-009-3426-7
10.1007/s10853-009-3445-4
10.1007/s10853-009-3446-3
10.1007/s10853-009-3738-7
10.1007/s10853-009-3806-z
10.1007/s10853-009-3858-0
10.1007/s10853-010-4227-8
10.1007/s10853-010-4236-7
10.1007/s10853-010-4337-3
10.1007/s10853-010-4372-0
10.1007/s10853-010-4471-y
10.1007/s10853-010-4481-9
10.1007/s10853-010-4502-8
10.1007/s10853-010-4546-9
10.1007/s10853-010-4630-1
10.1007/s10853-010-4696-9
10.1007/s10853-010-4831-7
10.1007/s10853-010-4875-8
10.1007/s10853-011-5395-x
10.1007/s10853-011-5414-y
10.1007/s10853-011-5462-3
10.1007/s10853-011-5493-9
10.1007/s10853-011-5501-0
10.1007/s10853-011-5543-3
10.1007/s10853-011-5544-2
10.1007/s10853-011-5561-1
10.1007/s10853-011-5732-0
10.1007/s10853-011-5792-1
10.1007/s10853-011-5854-4
10.1007/s10853-011-6041-3
10.1007/s10853-012-6250-4
10.1007/s10853-012-6323-4
10.1007/s10853-012-6336-z
10.1007/s10853-012-6455-6
10.1007/s10853-012-6488-x
10.1007/s10853-012-6684-8
10.1007/s10853-012-6752-0
10.1007/s10853-012-6802-7
10.1007/s10853-012-6840-1
10.1007/s10853-012-6915-z
10.1007/s10853-012-6993-y
10.1007/s10853-012-7021-y
10.1007/s10853-013-7201-4
10.1007/s10853-013-7212-1
10.1007/s10853-013-7763-1
10.1007/s10853-013-8009-y
10.1007/s10853-014-8219-y

10.1007/s10853-014-8418-6
10.1007/s10853-015-8853-z
10.1007/s10853-015-8855-x
10.1007/s10853-015-8941-0
10.1007/s10853-015-8992-2
10.1007/s10853-015-9068-z
10.1007/s10853-015-9177-8
10.1007/s10853-015-9200-0
10.1007/s10853-015-9307-3
10.1007/s10853-015-9452-8
10.1007/s10853-015-9554-3
10.1007/s10853-015-9579-7
10.1007/s10853-016-0026-1
10.1007/s10853-016-0089-z
10.1007/s10853-016-0363-0
10.1007/s10853-016-0703-0
10.1007/s10853-016-9815-9
10.1007/s10853-016-9995-3
10.1007/s10853-017-1091-9
10.1007/s10853-017-1159-6
10.1007/s10853-017-1365-2
10.1007/s10853-017-1437-3
10.1007/s10853-017-1470-2
10.1007/s10853-017-1549-9
10.1007/s10853-017-1682-5
10.1007/s10853-017-1699-9
10.1007/s10853-018-03274-x
10.1007/s10853-018-2144-4
10.1007/s10853-018-2236-1
10.1007/s10853-018-2701-x
10.1007/s10853-018-2851-x
10.1007/s10853-018-2891-2
10.1007/s10853-018-2919-7
10.1007/s10853-018-2941-9
10.1007/s10853-018-3002-0
10.1007/s10853-018-3081-y
10.1007/s10853-019-03566-w
10.1007/s10853-019-03569-7
10.1007/s10853-019-03639-w
10.1007/s10853-019-03821-0
10.1007/s10853-019-03840-x
10.1007/s10853-019-03884-z
10.1007/s10853-020-04375-2
10.1007/s10854-015-3946-7
10.1007/s10854-019-01390-1
10.1007/s10876-016-1025-8
10.1007/s10891-016-1389-5
10.1007/s10894-016-0060-7
10.1007/s10921-005-8783-9
10.1007/s10921-006-0003-8
10.1007/s10921-006-0009-2
10.1007/s10921-010-0072-6
10.1007/s10921-011-0125-5
10.1007/s10921-015-0332-6

10.1007/s10921-016-0354-8
10.1007/s10921-017-0393-9
10.1007/s10921-019-0640-3
10.1007/s10946-011-9247-6
10.1007/s10965-019-1806-5
10.1007/s10967-005-0055-8
10.1007/s10967-015-4063-z
10.1007/s10971-015-3668-6
10.1007/s10973-005-0701-y
10.1007/s10973-012-2304-8
10.1007/s10973-012-2685-8
10.1007/s10973-016-5785-z
10.1007/s10973-018-7085-2
10.1007/s10973-018-7420-7
10.1007/s10973-018-7431-4
10.1007/s10973-019-08121-y
10.1007/s10973-020-09375-7
10.1007/s11003-006-0091-8
10.1007/s11003-018-0146-7
10.1007/s11003-019-00233-y
10.1007/s11012-008-9126-6
10.1007/s11015-011-9472-9
10.1007/s11015-012-9579-7
10.1007/s11015-013-9767-0
10.1007/s11015-014-9813-6
10.1007/s11015-014-9870-x
10.1007/s11015-014-9916-0
10.1007/s11015-014-9984-1
10.1007/s11015-015-0034-4
10.1007/s11015-016-0179-9
10.1007/s11015-016-0340-5
10.1007/s11015-017-0397-9
10.1007/s11015-017-0398-8
10.1007/s11015-017-0412-1
10.1007/s11015-017-0470-4
10.1007/s11015-017-0506-9
10.1007/s11015-017-0507-8
10.1007/s11015-017-0533-6
10.1007/s11015-018-0564-7
10.1007/s11015-018-0596-z
10.1007/s11015-018-0627-9
10.1007/s11015-018-0638-6
10.1007/s11015-019-00837-4
10.1007/s11018-015-0844-6
10.1007/s11041-006-0041-y
10.1007/s11041-006-0096-9
10.1007/s11041-006-0097-8
10.1007/s11041-006-0101-3
10.1007/s11041-008-9023-6
10.1007/s11041-009-9118-8
10.1007/s11041-010-9201-1
10.1007/s11041-011-9355-5
10.1007/s11041-014-9685-1
10.1007/s11041-015-9794-5

10.1007/s11041-016-0037-1
10.1007/s11041-016-9950-6
10.1007/s11041-017-0113-1
10.1007/s11041-018-0232-3
10.1007/s11041-018-0247-9
10.1007/s11041-018-0277-3
10.1007/s11041-018-0286-2
10.1007/s11041-019-00343-z
10.1007/s11043-008-9049-6
10.1007/s11043-008-9065-6
10.1007/s11043-018-09406-9
10.1007/s11082-016-0779-4
10.1007/s11085-004-7807-7
10.1007/s11085-005-1951-6
10.1007/s11085-005-1953-4
10.1007/s11085-005-8529-1
10.1007/s11085-006-9002-5
10.1007/s11085-006-9004-3
10.1007/s11085-006-9016-z
10.1007/s11085-006-9019-9
10.1007/s11085-006-9020-3
10.1007/s11085-006-9024-z
10.1007/s11085-006-9029-7
10.1007/s11085-006-9036-8
10.1007/s11085-006-9041-y
10.1007/s11085-006-9044-8
10.1007/s11085-006-9048-4
10.1007/s11085-007-9052-3
10.1007/s11085-007-9056-z
10.1007/s11085-007-9062-1
10.1007/s11085-007-9066-x
10.1007/s11085-007-9067-9
10.1007/s11085-007-9069-7
10.1007/s11085-007-9072-z
10.1007/s11085-007-9077-7
10.1007/s11085-007-9079-5
10.1007/s11085-007-9085-7
10.1007/s11085-007-9088-4
10.1007/s11085-008-9092-3
10.1007/s11085-008-9133-y
10.1007/s11085-009-9159-9
10.1007/s11085-010-9206-6
10.1007/s11085-010-9207-5
10.1007/s11085-010-9210-x
10.1007/s11085-010-9211-9
10.1007/s11085-010-9214-6
10.1007/s11085-010-9217-3
10.1007/s11085-010-9221-7
10.1007/s11085-010-9223-5
10.1007/s11085-010-9228-0
10.1007/s11085-010-9230-6
10.1007/s11085-011-9234-x
10.1007/s11085-011-9245-7
10.1007/s11085-011-9246-6

10.1007/s11085-011-9251-9
10.1007/s11085-011-9257-3
10.1007/s11085-011-9262-6
10.1007/s11085-011-9264-4
10.1007/s11085-012-9283-9
10.1007/s11085-012-9288-4
10.1007/s11085-012-9292-8
10.1007/s11085-012-9297-3
10.1007/s11085-012-9322-6
10.1007/s11085-012-9354-y
10.1007/s11085-013-9359-1
10.1007/s11085-013-9361-7
10.1007/s11085-013-9362-6
10.1007/s11085-013-9373-3
10.1007/s11085-013-9393-z
10.1007/s11085-013-9399-6
10.1007/s11085-013-9405-z
10.1007/s11085-013-9412-0
10.1007/s11085-013-9414-y
10.1007/s11085-013-9416-9
10.1007/s11085-013-9417-8
10.1007/s11085-013-9418-7
10.1007/s11085-013-9422-y
10.1007/s11085-013-9425-8
10.1007/s11085-013-9426-7
10.1007/s11085-013-9427-6
10.1007/s11085-013-9428-5
10.1007/s11085-013-9440-9
10.1007/s11085-013-9449-0
10.1007/s11085-013-9454-3
10.1007/s11085-014-9476-5
10.1007/s11085-014-9483-6
10.1007/s11085-014-9487-2
10.1007/s11085-014-9491-6
10.1007/s11085-014-9500-9
10.1007/s11085-014-9502-7
10.1007/s11085-014-9503-6
10.1007/s11085-014-9505-4
10.1007/s11085-014-9509-0
10.1007/s11085-014-9513-4
10.1007/s11085-014-9517-0
10.1007/s11085-014-9522-3
10.1007/s11085-014-9524-1
10.1007/s11085-014-9525-0
10.1007/s11085-015-9529-4
10.1007/s11085-015-9537-4
10.1007/s11085-015-9543-6
10.1007/s11085-015-9555-2
10.1007/s11085-015-9556-1
10.1007/s11085-015-9566-z
10.1007/s11085-015-9568-x
10.1007/s11085-015-9569-9
10.1007/s11085-015-9570-3
10.1007/s11085-015-9580-1

10.1007/s11085-015-9590-z
10.1007/s11085-015-9599-3
10.1007/s11085-015-9601-0
10.1007/s11085-015-9604-x
10.1007/s11085-015-9606-8
10.1007/s11085-016-9619-y
10.1007/s11085-016-9623-2
10.1007/s11085-016-9627-y
10.1007/s11085-016-9633-0
10.1007/s11085-016-9635-y
10.1007/s11085-016-9646-8
10.1007/s11085-016-9655-7
10.1007/s11085-016-9656-6
10.1007/s11085-016-9657-5
10.1007/s11085-016-9692-2
10.1007/s11085-016-9702-4
10.1007/s11085-016-9704-2
10.1007/s11085-016-9707-z
10.1007/s11085-016-9710-4
10.1007/s11085-017-9732-6
10.1007/s11085-017-9744-2
10.1007/s11085-017-9746-0
10.1007/s11085-017-9759-8
10.1007/s11085-017-9783-8
10.1007/s11085-017-9787-4
10.1007/s11085-017-9789-2
10.1007/s11085-017-9790-9
10.1007/s11085-017-9791-8
10.1007/s11085-017-9792-7
10.1007/s11085-017-9797-2
10.1007/s11085-017-9804-7
10.1007/s11085-017-9808-3
10.1007/s11085-017-9813-6
10.1007/s11085-017-9822-5
10.1007/s11085-017-9825-2
10.1007/s11085-017-9828-z
10.1007/s11085-017-9830-5
10.1007/s11085-017-9831-4
10.1007/s11085-018-9838-5
10.1007/s11085-018-9839-4
10.1007/s11085-018-9842-9
10.1007/s11085-018-9846-5
10.1007/s11085-018-9847-4
10.1007/s11085-018-9849-2
10.1007/s11085-018-9853-6
10.1007/s11085-018-9856-3
10.1007/s11085-018-9857-2
10.1007/s11085-018-9865-2
10.1007/s11085-018-9866-1
10.1007/s11085-018-9867-0
10.1007/s11085-018-9868-z
10.1007/s11085-018-9869-y
10.1007/s11085-018-9871-4
10.1007/s11085-019-09888-z

10.1007/s11085-019-09911-3
10.1007/s11085-019-09913-1
10.1007/s11085-019-09914-0
10.1007/s11085-019-09922-0
10.1007/s11085-019-09930-0
10.1007/s11085-019-09937-7
10.1007/s11085-019-09945-7
10.1007/s11085-019-09946-6
10.1007/s11085-020-09960-z
10.1007/s11085-020-09961-y
10.1007/s11090-013-9445-9
10.1007/s11106-007-0056-y
10.1007/s11106-009-9150-7
10.1007/s11106-017-9866-8
10.1007/s11106-017-9919-z
10.1007/s11148-007-0026-4
10.1007/s11148-016-9925-6
10.1007/s11148-016-9981-y
10.1007/s11164-010-0182-x
10.1007/s11182-009-9240-5
10.1007/s11182-015-0510-0
10.1007/s11182-019-01658-3
10.1007/s11223-008-0041-9
10.1007/s11223-008-9068-1
10.1007/s11223-014-9513-2
10.1007/s11223-014-9588-9
10.1007/s11223-015-9657-8
10.1007/s11223-015-9716-1
10.1007/s11223-016-9744-5
10.1007/s11223-017-9825-0
10.1007/s11223-017-9903-3
10.1007/s11223-018-0002-x
10.1007/s11223-018-0008-4
10.1007/s11223-018-9947-z
10.1007/s11223-018-9995-4
10.1007/s11223-019-00055-y
10.1007/s11223-019-00066-9
10.1007/s11223-020-00135-4
10.1007/s11249-004-3204-2
10.1007/s11249-004-8096-7
10.1007/s11249-006-9099-3
10.1007/s11249-008-9372-8
10.1007/s11249-008-9384-4
10.1007/s11249-011-9912-5
10.1007/s11249-012-0068-8
10.1007/s11249-013-0126-x
10.1007/s11249-013-0269-9
10.1007/s11249-014-0299-y
10.1007/s11249-017-0881-1
10.1007/s11249-018-0994-1
10.1007/s11249-020-1266-4
10.1007/s11265-015-1029-x
10.1007/s11340-009-9271-4
10.1007/s11340-011-9499-7

10.1007/s11340-011-9505-0
10.1007/s11340-011-9557-1
10.1007/s11340-013-9771-0
10.1007/s11340-013-9793-7
10.1007/s11340-014-9926-7
10.1007/s11340-014-9940-9
10.1007/s11340-014-9954-3
10.1007/s11340-014-9963-2
10.1007/s11340-015-0009-1
10.1007/s11340-015-0083-4
10.1007/s11340-015-0092-3
10.1007/s11340-015-0104-3
10.1007/s11340-015-9998-z
10.1007/s11340-016-0203-9
10.1007/s11340-016-0234-2
10.1007/s11340-017-0286-y
10.1007/s11340-018-0418-z
10.1007/s11340-019-00490-7
10.1007/s11431-006-0238-8
10.1007/s11431-009-0091-7
10.1007/s11431-009-0217-y
10.1007/s11431-010-3223-1
10.1007/s11431-011-4607-6
10.1007/s11431-012-4764-2
10.1007/s11431-014-5555-8
10.1007/s11431-014-5610-5
10.1007/s11431-014-5679-x
10.1007/s11431-016-0101-5
10.1007/s11431-017-9123-5
10.1007/s11431-018-9351-9
10.1007/s11431-018-9353-2
10.1007/s11431-018-9372-8
10.1007/s11431-018-9477-6
10.1007/s11431-019-1452-8
10.1007/s11431-019-9519-9
10.1007/s11431-019-9525-3
10.1007/s11431-019-9548-x
10.1007/s11433-011-4317-x
10.1007/s11433-012-4630-z
10.1007/s11433-013-5013-9
10.1007/s11433-013-5364-2
10.1007/s11434-014-0140-x
10.1007/s11434-014-0228-3
10.1007/s11434-016-1098-7
10.1007/s11465-007-0048-0
10.1007/s11465-008-0040-3
10.1007/s11465-014-0301-2
10.1007/s11465-015-0362-x
10.1007/s11465-018-0475-0
10.1007/s11465-018-0489-7
10.1007/s11595-009-5796-8
10.1007/s11595-010-0004-4
10.1007/s11595-010-0025-8
10.1007/s11595-011-0335-9

10.1007/s11595-013-0812-4
10.1007/s11595-015-1160-3
10.1007/s11595-015-1232-4
10.1007/s11595-016-1465-x
10.1007/s11595-016-1540-3
10.1007/s11595-017-1729-0
10.1007/s11595-018-1821-z
10.1007/s11633-016-1035-x
10.1007/s11661-000-0002-9
10.1007/s11661-000-0046-x
10.1007/s11661-000-0051-0
10.1007/s11661-000-0058-6
10.1007/s11661-000-0063-9
10.1007/s11661-000-0080-8
10.1007/s11661-000-0131-1
10.1007/s11661-000-0139-6
10.1007/s11661-000-0143-x
10.1007/s11661-000-0147-6
10.1007/s11661-000-0199-7
10.1007/s11661-000-0230-z
10.1007/s11661-000-0231-y
10.1007/s11661-000-0252-6
10.1007/s11661-000-0255-3
10.1007/s11661-000-0258-0
10.1007/s11661-000-0279-8
10.1007/s11661-000-0285-x
10.1007/s11661-001-0002-4
10.1007/s11661-001-0014-0
10.1007/s11661-001-0019-8
10.1007/s11661-001-0032-y
10.1007/s11661-001-0034-9
10.1007/s11661-001-0045-6
10.1007/s11661-001-0078-x
10.1007/s11661-001-0107-9
10.1007/s11661-001-0121-y
10.1007/s11661-001-0126-6
10.1007/s11661-001-0149-z
10.1007/s11661-001-0150-6
10.1007/s11661-001-0151-5
10.1007/s11661-001-0169-8
10.1007/s11661-001-0182-y
10.1007/s11661-001-0203-x
10.1007/s11661-001-0217-4
10.1007/s11661-001-0225-4
10.1007/s11661-001-0235-2
10.1007/s11661-001-0268-6
10.1007/s11661-001-0350-0
10.1007/s11661-001-1014-9
10.1007/s11661-002-0011-y
10.1007/s11661-002-0028-2
10.1007/s11661-002-0033-5
10.1007/s11661-002-0058-9
10.1007/s11661-002-0067-8
10.1007/s11661-002-0102-9

10.1007/s11661-002-0105-6
10.1007/s11661-002-0177-3
10.1007/s11661-002-0180-8
10.1007/s11661-002-0181-7
10.1007/s11661-002-0188-0
10.1007/s11661-002-0189-z
10.1007/s11661-002-0246-7
10.1007/s11661-002-0268-1
10.1007/s11661-002-0277-0
10.1007/s11661-002-0302-3
10.1007/s11661-002-0307-y
10.1007/s11661-002-0308-x
10.1007/s11661-002-0325-9
10.1007/s11661-002-0334-8
10.1007/s11661-002-0376-y
10.1007/s11661-002-0393-x
10.1007/s11661-003-0017-0
10.1007/s11661-003-0023-2
10.1007/s11661-003-0129-6
10.1007/s11661-003-0160-7
10.1007/s11661-003-0179-9
10.1007/s11661-003-0193-y
10.1007/s11661-003-0227-5
10.1007/s11661-003-0257-z
10.1007/s11661-003-0264-0
10.1007/s11661-003-0269-8
10.1007/s11661-003-0284-9
10.1007/s11661-003-0285-8
10.1007/s11661-003-0288-5
10.1007/s11661-003-0301-z
10.1007/s11661-003-0309-4
10.1007/s11661-003-0321-8
10.1007/s11661-004-0001-3
10.1007/s11661-004-0005-z
10.1007/s11661-004-0010-2
10.1007/s11661-004-0014-y
10.1007/s11661-004-0018-7
10.1007/s11661-004-0057-0
10.1007/s11661-004-0066-z
10.1007/s11661-004-0076-x
10.1007/s11661-004-0079-7
10.1007/s11661-004-0086-8
10.1007/s11661-004-0104-x
10.1007/s11661-004-0155-z
10.1007/s11661-004-0157-x
10.1007/s11661-004-0159-8
10.1007/s11661-004-0185-6
10.1007/s11661-004-0308-0
10.1007/s11661-004-0364-5
10.1007/s11661-004-0379-y
10.1007/s11661-004-1008-5
10.1007/s11661-004-1014-7
10.1007/s11661-005-0007-5
10.1007/s11661-005-0013-7

10.1007/s11661-005-0017-3
10.1007/s11661-005-0051-1
10.1007/s11661-005-0061-z
10.1007/s11661-005-0073-8
10.1007/s11661-005-0076-5
10.1007/s11661-005-0111-6
10.1007/s11661-005-0120-5
10.1007/s11661-005-0123-2
10.1007/s11661-005-0137-9
10.1007/s11661-005-0168-2
10.1007/s11661-005-0169-1
10.1007/s11661-005-0171-7
10.1007/s11661-005-0181-5
10.1007/s11661-005-0216-y
10.1007/s11661-005-0246-5
10.1007/s11661-005-0264-3
10.1007/s11661-005-0272-3
10.1007/s11661-005-0284-z
10.1007/s11661-005-0294-x
10.1007/s11661-005-0338-2
10.1007/s11661-005-1009-z
10.1007/s11661-006-0005-2
10.1007/s11661-006-0011-4
10.1007/s11661-006-0013-2
10.1007/s11661-006-0017-y
10.1007/s11661-006-0026-x
10.1007/s11661-006-0083-1
10.1007/s11661-006-0091-1
10.1007/s11661-006-0126-7
10.1007/s11661-006-0137-4
10.1007/s11661-006-0161-4
10.1007/s11661-006-0174-z
10.1007/s11661-006-0186-8
10.1007/s11661-006-0207-7
10.1007/s11661-006-0211-y
10.1007/s11661-006-0235-3
10.1007/s11661-006-0250-4
10.1007/s11661-006-1041-7
10.1007/s11661-006-1078-7
10.1007/s11661-006-9002-8
10.1007/s11661-006-9007-3
10.1007/s11661-006-9021-5
10.1007/s11661-006-9025-1
10.1007/s11661-006-9032-2
10.1007/s11661-006-9063-8
10.1007/s11661-006-9085-2
10.1007/s11661-007-9089-6
10.1007/s11661-007-9090-0
10.1007/s11661-007-9091-z
10.1007/s11661-007-9172-z
10.1007/s11661-007-9188-4
10.1007/s11661-007-9194-6
10.1007/s11661-007-9231-5
10.1007/s11661-007-9257-8

10.1007/s11661-007-9260-0
10.1007/s11661-007-9266-7
10.1007/s11661-007-9356-6
10.1007/s11661-007-9375-3
10.1007/s11661-007-9378-0
10.1007/s11661-007-9381-5
10.1007/s11661-007-9387-z
10.1007/s11661-008-9490-9
10.1007/s11661-008-9501-x
10.1007/s11661-008-9520-7
10.1007/s11661-008-9534-1
10.1007/s11661-008-9553-y
10.1007/s11661-008-9564-8
10.1007/s11661-008-9585-3
10.1007/s11661-008-9588-0
10.1007/s11661-008-9598-y
10.1007/s11661-008-9618-y
10.1007/s11661-008-9646-7
10.1007/s11661-008-9662-7
10.1007/s11661-008-9667-2
10.1007/s11661-008-9691-2
10.1007/s11661-008-9694-z
10.1007/s11661-008-9700-5
10.1007/s11661-008-9702-3
10.1007/s11661-008-9726-8
10.1007/s11661-008-9744-6
10.1007/s11661-008-9746-4
10.1007/s11661-008-9768-y
10.1007/s11661-009-0001-4
10.1007/s11661-009-0003-2
10.1007/s11661-009-0027-7
10.1007/s11661-009-0032-x
10.1007/s11661-009-0047-3
10.1007/s11661-009-0075-z
10.1007/s11661-009-9822-4
10.1007/s11661-009-9829-x
10.1007/s11661-009-9844-y
10.1007/s11661-009-9855-8
10.1007/s11661-009-9858-5
10.1007/s11661-009-9914-1
10.1007/s11661-009-9915-0
10.1007/s11661-009-9938-6
10.1007/s11661-009-9949-3
10.1007/s11661-009-9962-6
10.1007/s11661-009-9979-x
10.1007/s11661-010-0189-3
10.1007/s11661-010-0241-3
10.1007/s11661-010-0251-1
10.1007/s11661-010-0299-y
10.1007/s11661-010-0319-y
10.1007/s11661-010-0331-2
10.1007/s11661-010-0390-4
10.1007/s11661-010-0457-2
10.1007/s11661-010-0466-1

10.1007/s11661-010-0483-0
10.1007/s11661-010-0486-x
10.1007/s11661-010-0503-0
10.1007/s11661-010-0508-8
10.1007/s11661-010-0534-6
10.1007/s11661-010-0544-4
10.1007/s11661-010-0566-y
10.1007/s11661-010-0585-8
10.1007/s11661-010-0587-6
10.1007/s11661-011-0613-3
10.1007/s11661-011-0626-y
10.1007/s11661-011-0664-5
10.1007/s11661-011-0673-4
10.1007/s11661-011-0715-y
10.1007/s11661-011-0738-4
10.1007/s11661-011-0748-2
10.1007/s11661-011-0760-6
10.1007/s11661-011-0774-0
10.1007/s11661-011-0779-8
10.1007/s11661-011-0802-0
10.1007/s11661-011-0803-z
10.1007/s11661-011-0823-8
10.1007/s11661-011-0833-6
10.1007/s11661-011-0837-2
10.1007/s11661-011-0838-1
10.1007/s11661-011-0851-4
10.1007/s11661-011-0877-7
10.1007/s11661-011-0889-3
10.1007/s11661-011-0897-3
10.1007/s11661-011-0920-8
10.1007/s11661-011-0926-2
10.1007/s11661-011-0928-0
10.1007/s11661-011-0969-4
10.1007/s11661-011-0972-9
10.1007/s11661-011-0979-2
10.1007/s11661-011-0990-7
10.1007/s11661-011-0995-2
10.1007/s11661-011-1010-7
10.1007/s11661-011-1013-4
10.1007/s11661-011-1023-2
10.1007/s11661-011-1025-0
10.1007/s11661-011-1027-y
10.1007/s11661-011-1031-2
10.1007/s11661-011-1035-y
10.1007/s11661-011-1047-7
10.1007/s11661-011-1055-7
10.1007/s11661-011-1058-4
10.1007/s11661-012-1085-9
10.1007/s11661-012-1104-x
10.1007/s11661-012-1146-0
10.1007/s11661-012-1162-0
10.1007/s11661-012-1163-z
10.1007/s11661-012-1164-y
10.1007/s11661-012-1165-x

10.1007/s11661-012-1187-4
10.1007/s11661-012-1207-4
10.1007/s11661-012-1212-7
10.1007/s11661-012-1216-3
10.1007/s11661-012-1255-9
10.1007/s11661-012-1256-8
10.1007/s11661-012-1260-z
10.1007/s11661-012-1285-3
10.1007/s11661-012-1318-y
10.1007/s11661-012-1327-x
10.1007/s11661-012-1343-x
10.1007/s11661-012-1363-6
10.1007/s11661-012-1397-9
10.1007/s11661-012-1412-1
10.1007/s11661-012-1456-2
10.1007/s11661-012-1457-1
10.1007/s11661-012-1459-z
10.1007/s11661-012-1491-z
10.1007/s11661-012-1527-4
10.1007/s11661-012-1538-1
10.1007/s11661-012-1540-7
10.1007/s11661-012-1569-7
10.1007/s11661-012-1575-9
10.1007/s11661-012-1597-3
10.1007/s11661-013-1618-x
10.1007/s11661-013-1631-0
10.1007/s11661-013-1645-7
10.1007/s11661-013-1657-3
10.1007/s11661-013-1660-8
10.1007/s11661-013-1668-0
10.1007/s11661-013-1675-1
10.1007/s11661-013-1677-z
10.1007/s11661-013-1679-x
10.1007/s11661-013-1680-4
10.1007/s11661-013-1704-0
10.1007/s11661-013-1737-4
10.1007/s11661-013-1767-y
10.1007/s11661-013-1779-7
10.1007/s11661-013-1803-y
10.1007/s11661-013-1816-6
10.1007/s11661-013-1840-6
10.1007/s11661-013-1849-x
10.1007/s11661-013-1858-9
10.1007/s11661-013-1859-8
10.1007/s11661-013-1887-4
10.1007/s11661-013-1903-8
10.1007/s11661-013-1944-z
10.1007/s11661-013-1954-x
10.1007/s11661-013-1959-5
10.1007/s11661-013-1961-y
10.1007/s11661-013-1962-x
10.1007/s11661-013-1971-9
10.1007/s11661-013-1975-5
10.1007/s11661-013-1984-4

10.1007/s11661-013-1985-3
10.1007/s11661-013-2005-3
10.1007/s11661-013-2088-x
10.1007/s11661-013-2097-9
10.1007/s11661-013-2098-8
10.1007/s11661-013-2138-4
10.1007/s11661-013-2147-3
10.1007/s11661-013-2157-1
10.1007/s11661-013-2172-2
10.1007/s11661-013-2178-9
10.1007/s11661-014-2190-8
10.1007/s11661-014-2197-1
10.1007/s11661-014-2198-0
10.1007/s11661-014-2199-z
10.1007/s11661-014-2212-6
10.1007/s11661-014-2244-y
10.1007/s11661-014-2256-7
10.1007/s11661-014-2271-8
10.1007/s11661-014-2284-3
10.1007/s11661-014-2285-2
10.1007/s11661-014-2291-4
10.1007/s11661-014-2304-3
10.1007/s11661-014-2309-y
10.1007/s11661-014-2312-3
10.1007/s11661-014-2325-y
10.1007/s11661-014-2357-3
10.1007/s11661-014-2391-1
10.1007/s11661-014-2397-8
10.1007/s11661-014-2439-2
10.1007/s11661-014-2462-3
10.1007/s11661-014-2469-9
10.1007/s11661-014-2499-3
10.1007/s11661-014-2506-8
10.1007/s11661-014-2508-6
10.1007/s11661-014-2509-5
10.1007/s11661-014-2512-x
10.1007/s11661-014-2554-0
10.1007/s11661-014-2558-9
10.1007/s11661-014-2559-8
10.1007/s11661-014-2564-y
10.1007/s11661-014-2572-y
10.1007/s11661-014-2602-9
10.1007/s11661-014-2614-5
10.1007/s11661-014-2617-2
10.1007/s11661-014-2652-z
10.1007/s11661-014-2669-3
10.1007/s11661-014-2676-4
10.1007/s11661-014-2682-6
10.1007/s11661-014-2706-2
10.1007/s11661-014-2709-z
10.1007/s11661-014-2727-x
10.1007/s11661-014-2733-z
10.1007/s11661-015-2748-0
10.1007/s11661-015-2752-4

10.1007/s11661-015-2787-6
10.1007/s11661-015-2794-7
10.1007/s11661-015-2815-6
10.1007/s11661-015-2818-3
10.1007/s11661-015-2831-6
10.1007/s11661-015-2857-9
10.1007/s11661-015-2860-1
10.1007/s11661-015-2869-5
10.1007/s11661-015-2898-0
10.1007/s11661-015-2912-6
10.1007/s11661-015-2964-7
10.1007/s11661-015-2996-z
10.1007/s11661-015-3000-7
10.1007/s11661-015-3030-1
10.1007/s11661-015-3031-0
10.1007/s11661-015-3033-y
10.1007/s11661-015-3035-9
10.1007/s11661-015-3046-6
10.1007/s11661-015-3064-4
10.1007/s11661-015-3092-0
10.1007/s11661-015-3114-y
10.1007/s11661-015-3126-7
10.1007/s11661-015-3130-y
10.1007/s11661-015-3154-3
10.1007/s11661-015-3193-9
10.1007/s11661-015-3201-0
10.1007/s11661-015-3244-2
10.1007/s11661-015-3252-2
10.1007/s11661-015-3253-1
10.1007/s11661-015-3269-6
10.1007/s11661-015-3273-x
10.1007/s11661-015-3289-2
10.1007/s11661-015-3291-8
10.1007/s11661-015-3300-y
10.1007/s11661-015-3315-4
10.1007/s11661-016-3329-6
10.1007/s11661-016-3346-5
10.1007/s11661-016-3361-6
10.1007/s11661-016-3408-8
10.1007/s11661-016-3415-9
10.1007/s11661-016-3416-8
10.1007/s11661-016-3417-7
10.1007/s11661-016-3421-y
10.1007/s11661-016-3422-x
10.1007/s11661-016-3466-y
10.1007/s11661-016-3480-0
10.1007/s11661-016-3495-6
10.1007/s11661-016-3501-z
10.1007/s11661-016-3505-8
10.1007/s11661-016-3542-3
10.1007/s11661-016-3544-1
10.1007/s11661-016-3571-y
10.1007/s11661-016-3593-5
10.1007/s11661-016-3600-x

10.1007/s11661-016-3603-7
10.1007/s11661-016-3609-1
10.1007/s11661-016-3619-z
10.1007/s11661-016-3623-3
10.1007/s11661-016-3664-7
10.1007/s11661-016-3694-1
10.1007/s11661-016-3712-3
10.1007/s11661-016-3717-y
10.1007/s11661-016-3718-x
10.1007/s11661-016-3751-9
10.1007/s11661-016-3755-5
10.1007/s11661-016-3775-1
10.1007/s11661-016-3784-0
10.1007/s11661-016-3790-2
10.1007/s11661-016-3793-z
10.1007/s11661-016-3796-9
10.1007/s11661-016-3808-9
10.1007/s11661-016-3837-4
10.1007/s11661-016-3873-0
10.1007/s11661-016-3904-x
10.1007/s11661-016-3907-7
10.1007/s11661-016-3929-1
10.1007/s11661-016-3931-7
10.1007/s11661-017-3977-1
10.1007/s11661-017-3999-8
10.1007/s11661-017-4007-z
10.1007/s11661-017-4015-z
10.1007/s11661-017-4019-8
10.1007/s11661-017-4027-8
10.1007/s11661-017-4037-6
10.1007/s11661-017-4040-y
10.1007/s11661-017-4044-7
10.1007/s11661-017-4057-2
10.1007/s11661-017-4066-1
10.1007/s11661-017-4067-0
10.1007/s11661-017-4113-y
10.1007/s11661-017-4114-x
10.1007/s11661-017-4115-9
10.1007/s11661-017-4123-9
10.1007/s11661-017-4135-5
10.1007/s11661-017-4138-2
10.1007/s11661-017-4141-7
10.1007/s11661-017-4151-5
10.1007/s11661-017-4162-2
10.1007/s11661-017-4173-z
10.1007/s11661-017-4194-7
10.1007/s11661-017-4223-6
10.1007/s11661-017-4224-5
10.1007/s11661-017-4243-2
10.1007/s11661-017-4255-y
10.1007/s11661-017-4256-x
10.1007/s11661-017-4296-2
10.1007/s11661-017-4300-x
10.1007/s11661-017-4305-5

10.1007/s11661-017-4312-6
10.1007/s11661-017-4317-1
10.1007/s11661-017-4322-4
10.1007/s11661-017-4323-3
10.1007/s11661-017-4336-y
10.1007/s11661-017-4339-8
10.1007/s11661-017-4346-9
10.1007/s11661-017-4355-8
10.1007/s11661-017-4356-7
10.1007/s11661-017-4367-4
10.1007/s11661-017-4380-7
10.1007/s11661-017-4389-y
10.1007/s11661-017-4392-3
10.1007/s11661-017-4399-9
10.1007/s11661-017-4422-1
10.1007/s11661-017-4431-0
10.1007/s11661-017-4445-7
10.1007/s11661-017-4455-5
10.1007/s11661-017-4465-3
10.1007/s11661-018-05104-w
10.1007/s11661-018-05109-5
10.1007/s11661-018-4467-9
10.1007/s11661-018-4469-7
10.1007/s11661-018-4474-x
10.1007/s11661-018-4483-9
10.1007/s11661-018-4493-7
10.1007/s11661-018-4514-6
10.1007/s11661-018-4515-5
10.1007/s11661-018-4519-1
10.1007/s11661-018-4526-2
10.1007/s11661-018-4533-3
10.1007/s11661-018-4534-2
10.1007/s11661-018-4539-x
10.1007/s11661-018-4545-z
10.1007/s11661-018-4558-7
10.1007/s11661-018-4567-6
10.1007/s11661-018-4572-9
10.1007/s11661-018-4574-7
10.1007/s11661-018-4575-6
10.1007/s11661-018-4579-2
10.1007/s11661-018-4587-2
10.1007/s11661-018-4619-y
10.1007/s11661-018-4635-y
10.1007/s11661-018-4640-1
10.1007/s11661-018-4643-y
10.1007/s11661-018-4644-x
10.1007/s11661-018-4648-6
10.1007/s11661-018-4652-x
10.1007/s11661-018-4669-1
10.1007/s11661-018-4671-7
10.1007/s11661-018-4672-6
10.1007/s11661-018-4673-5
10.1007/s11661-018-4681-5
10.1007/s11661-018-4682-4

10.1007/s11661-018-4683-3
10.1007/s11661-018-4685-1
10.1007/s11661-018-4689-x
10.1007/s11661-018-4691-3
10.1007/s11661-018-4701-5
10.1007/s11661-018-4702-4
10.1007/s11661-018-4703-3
10.1007/s11661-018-4704-2
10.1007/s11661-018-4705-1
10.1007/s11661-018-4706-0
10.1007/s11661-018-4707-z
10.1007/s11661-018-4708-y
10.1007/s11661-018-4709-x
10.1007/s11661-018-4710-4
10.1007/s11661-018-4711-3
10.1007/s11661-018-4712-2
10.1007/s11661-018-4726-9
10.1007/s11661-018-4727-8
10.1007/s11661-018-4729-6
10.1007/s11661-018-4730-0
10.1007/s11661-018-4734-9
10.1007/s11661-018-4736-7
10.1007/s11661-018-4737-6
10.1007/s11661-018-4745-6
10.1007/s11661-018-4746-5
10.1007/s11661-018-4748-3
10.1007/s11661-018-4752-7
10.1007/s11661-018-4755-4
10.1007/s11661-018-4756-3
10.1007/s11661-018-4757-2
10.1007/s11661-018-4758-1
10.1007/s11661-018-4759-0
10.1007/s11661-018-4760-7
10.1007/s11661-018-4761-6
10.1007/s11661-018-4762-5
10.1007/s11661-018-4763-4
10.1007/s11661-018-4764-3
10.1007/s11661-018-4767-0
10.1007/s11661-018-4768-z
10.1007/s11661-018-4769-y
10.1007/s11661-018-4770-5
10.1007/s11661-018-4776-z
10.1007/s11661-018-4777-y
10.1007/s11661-018-4778-x
10.1007/s11661-018-4779-9
10.1007/s11661-018-4780-3
10.1007/s11661-018-4788-8
10.1007/s11661-018-4793-y
10.1007/s11661-018-4795-9
10.1007/s11661-018-4826-6
10.1007/s11661-018-4835-5
10.1007/s11661-018-4842-6
10.1007/s11661-018-4853-3
10.1007/s11661-018-4857-z

10.1007/s11661-018-4858-y
10.1007/s11661-018-4876-9
10.1007/s11661-018-4878-7
10.1007/s11661-018-4896-5
10.1007/s11661-018-4898-3
10.1007/s11661-018-4902-y
10.1007/s11661-018-4914-7
10.1007/s11661-018-4918-3
10.1007/s11661-018-4923-6
10.1007/s11661-018-4926-3
10.1007/s11661-018-4971-y
10.1007/s11661-018-4997-1
10.1007/s11661-018-5036-y
10.1007/s11661-018-5049-6
10.1007/s11661-018-5060-y
10.1007/s11661-018-5092-3
10.1007/s11661-018-5098-x
10.1007/s11661-019-05113-3
10.1007/s11661-019-05141-z
10.1007/s11661-019-05149-5
10.1007/s11661-019-05163-7
10.1007/s11661-019-05164-6
10.1007/s11661-019-05246-5
10.1007/s11661-019-05252-7
10.1007/s11661-019-05297-8
10.1007/s11661-019-05298-7
10.1007/s11661-019-05299-6
10.1007/s11661-019-05302-0
10.1007/s11661-019-05309-7
10.1007/s11661-019-05330-w
10.1007/s11661-019-05332-8
10.1007/s11661-019-05336-4
10.1007/s11661-019-05380-0
10.1007/s11661-019-05386-8
10.1007/s11661-019-05393-9
10.1007/s11661-019-05413-8
10.1007/s11661-019-05422-7
10.1007/s11661-019-05427-2
10.1007/s11661-019-05429-0
10.1007/s11661-019-05432-5
10.1007/s11661-019-05442-3
10.1007/s11661-019-05454-z
10.1007/s11661-019-05494-5
10.1007/s11661-019-05498-1
10.1007/s11661-019-05499-0
10.1007/s11661-019-05508-2
10.1007/s11661-019-05510-8
10.1007/s11661-019-05513-5
10.1007/s11661-019-05516-2
10.1007/s11661-019-05536-y
10.1007/s11661-019-05548-8
10.1007/s11661-019-05549-7
10.1007/s11661-019-05577-3
10.1007/s11661-019-05581-7

10.1007/s11661-019-05592-4
10.1007/s11661-019-05595-1
10.1007/s11661-019-05600-7
10.1007/s11661-019-05611-4
10.1007/s11661-019-05619-w
10.1007/s11661-020-05626-2
10.1007/s11661-020-05627-1
10.1007/s11661-020-05633-3
10.1007/s11661-020-05634-2
10.1007/s11661-020-05646-y
10.1007/s11661-020-05652-0
10.1007/s11661-020-05681-9
10.1007/s11661-020-05690-8
10.1007/s11661-020-05696-2
10.1007/s11661-020-05710-7
10.1007/s11661-020-05724-1
10.1007/s11661-020-05734-z
10.1007/s11661-997-0001-1
10.1007/s11661-997-0050-5
10.1007/s11661-997-0052-3
10.1007/s11661-997-0125-3
10.1007/s11661-997-0151-1
10.1007/s11661-997-0156-9
10.1007/s11661-997-0172-9
10.1007/s11661-997-0214-3
10.1007/s11661-997-0215-2
10.1007/s11661-997-0220-5
10.1007/s11661-997-0230-3
10.1007/s11661-997-0231-2
10.1007/s11661-997-0241-0
10.1007/s11661-997-0271-7
10.1007/s11661-997-0273-5
10.1007/s11661-997-0288-y
10.1007/s11661-997-1009-2
10.1007/s11661-998-0020-6
10.1007/s11661-998-0032-2
10.1007/s11661-998-0039-8
10.1007/s11661-998-0042-0
10.1007/s11661-998-0096-z
10.1007/s11661-998-0135-9
10.1007/s11661-998-0161-7
10.1007/s11661-998-0207-x
10.1007/s11661-998-0214-y
10.1007/s11661-998-0239-2
10.1007/s11661-998-0243-6
10.1007/s11661-998-0253-4
10.1007/s11661-998-0271-2
10.1007/s11661-998-0274-z
10.1007/s11661-998-0309-5
10.1007/s11661-998-0319-3
10.1007/s11661-998-0320-x
10.1007/s11661-998-0330-8
10.1007/s11661-998-0360-2
10.1007/s11661-998-0370-0

10.1007/s11661-999-0029-5
10.1007/s11661-999-0038-4
10.1007/s11661-999-0043-7
10.1007/s11661-999-0044-6
10.1007/s11661-999-0047-3
10.1007/s11661-999-0095-8
10.1007/s11661-999-0116-7
10.1007/s11661-999-0117-6
10.1007/s11661-999-0128-3
10.1007/s11661-999-0174-x
10.1007/s11661-999-0186-6
10.1007/s11661-999-0194-6
10.1007/s11661-999-0274-7
10.1007/s11661-999-0303-6
10.1007/s11661-999-0308-1
10.1007/s11661-999-0310-7
10.1007/s11661-999-0331-2
10.1007/s11661-999-0332-1
10.1007/s11663-000-0016-0
10.1007/s11663-000-0017-z
10.1007/s11663-000-0117-9
10.1007/s11663-001-0030-x
10.1007/s11663-004-0014-8
10.1007/s11663-004-0100-y
10.1007/s11663-005-0009-0
10.1007/s11663-005-0026-z
10.1007/s11663-005-0027-y
10.1007/s11663-005-0043-y
10.1007/s11663-005-0056-6
10.1007/s11663-006-0060-5
10.1007/s11663-007-9118-2
10.1007/s11663-008-9208-9
10.1007/s11663-009-9227-1
10.1007/s11663-010-9410-4
10.1007/s11663-010-9426-9
10.1007/s11663-011-9577-3
10.1007/s11663-011-9608-0
10.1007/s11663-012-9655-1
10.1007/s11663-012-9691-x
10.1007/s11663-012-9723-6
10.1007/s11663-013-0003-x
10.1007/s11663-013-9795-y
10.1007/s11663-013-9846-4
10.1007/s11663-013-9883-z
10.1007/s11663-013-9909-6
10.1007/s11663-014-0054-7
10.1007/s11663-014-0086-z
10.1007/s11663-014-0117-9
10.1007/s11663-014-0183-z
10.1007/s11663-015-0461-4
10.1007/s11663-015-0530-8
10.1007/s11663-015-0580-y
10.1007/s11663-016-0665-2
10.1007/s11663-016-0667-0

10.1007/s11663-016-0688-8
10.1007/s11663-016-0790-y
10.1007/s11663-016-0823-6
10.1007/s11663-016-0847-y
10.1007/s11663-016-0854-z
10.1007/s11663-016-0892-6
10.1007/s11663-017-0926-8
10.1007/s11663-017-0941-9
10.1007/s11663-017-0994-9
10.1007/s11663-017-1039-0
10.1007/s11663-017-1068-8
10.1007/s11663-017-1098-2
10.1007/s11663-017-1110-x
10.1007/s11663-017-1137-z
10.1007/s11663-018-1169-z
10.1007/s11663-018-1201-3
10.1007/s11663-018-1202-2
10.1007/s11663-018-1256-1
10.1007/s11663-018-1293-9
10.1007/s11663-018-1313-9
10.1007/s11663-018-1347-z
10.1007/s11663-018-1383-8
10.1007/s11663-018-1407-4
10.1007/s11663-018-1415-4
10.1007/s11663-018-1458-6
10.1007/s11663-018-1489-z
10.1007/s11663-019-01606-z
10.1007/s11663-019-01614-z
10.1007/s11663-019-01672-3
10.1007/s11663-019-01716-8
10.1007/s11663-019-01729-3
10.1007/s11663-020-01833-9
10.1007/s11663-997-0117-0
10.1007/s11663-998-0144-5
10.1007/s11664-016-4789-6
10.1007/s11664-997-0252-z
10.1007/s11665-002-0003-5
10.1007/s11665-006-9008-9
10.1007/s11665-007-9046-y
10.1007/s11665-007-9108-1
10.1007/s11665-007-9112-5
10.1007/s11665-007-9171-7
10.1007/s11665-008-9213-9
10.1007/s11665-008-9297-2
10.1007/s11665-009-9395-9
10.1007/s11665-009-9424-8
10.1007/s11665-009-9431-9
10.1007/s11665-009-9436-4
10.1007/s11665-009-9522-7
10.1007/s11665-009-9525-4
10.1007/s11665-009-9533-4
10.1007/s11665-009-9567-7
10.1007/s11665-009-9584-6
10.1007/s11665-009-9587-3

10.1007/s11665-010-9605-5
10.1007/s11665-010-9728-8
10.1007/s11665-010-9731-0
10.1007/s11665-010-9749-3
10.1007/s11665-011-0067-1
10.1007/s11665-012-0153-z
10.1007/s11665-012-0183-6
10.1007/s11665-012-0188-1
10.1007/s11665-012-0191-6
10.1007/s11665-012-0206-3
10.1007/s11665-012-0246-8
10.1007/s11665-012-0385-y
10.1007/s11665-012-0398-6
10.1007/s11665-012-0412-z
10.1007/s11665-012-0422-x
10.1007/s11665-012-0433-7
10.1007/s11665-012-0439-1
10.1007/s11665-012-0453-3
10.1007/s11665-013-0477-3
10.1007/s11665-013-0520-4
10.1007/s11665-013-0531-1
10.1007/s11665-013-0532-0
10.1007/s11665-013-0558-3
10.1007/s11665-013-0565-4
10.1007/s11665-013-0592-1
10.1007/s11665-013-0611-2
10.1007/s11665-013-0665-1
10.1007/s11665-013-0677-x
10.1007/s11665-013-0699-4
10.1007/s11665-013-0702-0
10.1007/s11665-013-0704-y
10.1007/s11665-013-0706-9
10.1007/s11665-013-0714-9
10.1007/s11665-013-0721-x
10.1007/s11665-013-0731-8
10.1007/s11665-013-0771-0
10.1007/s11665-013-0772-z
10.1007/s11665-013-0774-x
10.1007/s11665-013-0809-3
10.1007/s11665-013-0814-6
10.1007/s11665-013-0820-8
10.1007/s11665-013-0841-3
10.1007/s11665-013-0843-1
10.1007/s11665-014-0863-5
10.1007/s11665-014-0913-z
10.1007/s11665-014-0981-0
10.1007/s11665-014-1028-2
10.1007/s11665-014-1035-3
10.1007/s11665-014-1048-y
10.1007/s11665-014-1063-z
10.1007/s11665-014-1067-8
10.1007/s11665-014-1099-0
10.1007/s11665-014-1123-4
10.1007/s11665-014-1126-1

10.1007/s11665-014-1130-5
10.1007/s11665-014-1172-8
10.1007/s11665-014-1177-3
10.1007/s11665-014-1238-7
10.1007/s11665-014-1240-0
10.1007/s11665-014-1255-6
10.1007/s11665-014-1272-5
10.1007/s11665-014-1273-4
10.1007/s11665-014-1278-z
10.1007/s11665-014-1279-y
10.1007/s11665-014-1307-y
10.1007/s11665-014-1310-3
10.1007/s11665-014-1339-3
10.1007/s11665-014-1351-7
10.1007/s11665-014-1368-y
10.1007/s11665-014-1379-8
10.1007/s11665-015-1400-x
10.1007/s11665-015-1413-5
10.1007/s11665-015-1421-5
10.1007/s11665-015-1504-3
10.1007/s11665-015-1513-2
10.1007/s11665-015-1538-6
10.1007/s11665-015-1542-x
10.1007/s11665-015-1588-9
10.1007/s11665-015-1617-8
10.1007/s11665-015-1634-7
10.1007/s11665-015-1666-z
10.1007/s11665-015-1678-8
10.1007/s11665-015-1711-y
10.1007/s11665-015-1756-y
10.1007/s11665-015-1774-9
10.1007/s11665-015-1828-z
10.1007/s11665-015-1835-0
10.1007/s11665-015-1846-x
10.1007/s11665-016-1884-z
10.1007/s11665-016-1887-9
10.1007/s11665-016-1933-7
10.1007/s11665-016-1967-x
10.1007/s11665-016-2012-9
10.1007/s11665-016-2032-5
10.1007/s11665-016-2049-9
10.1007/s11665-016-2051-2
10.1007/s11665-016-2081-9
10.1007/s11665-016-2091-7
10.1007/s11665-016-2104-6
10.1007/s11665-016-2122-4
10.1007/s11665-016-2138-9
10.1007/s11665-016-2150-0
10.1007/s11665-016-2154-9
10.1007/s11665-016-2159-4
10.1007/s11665-016-2169-2
10.1007/s11665-016-2216-z
10.1007/s11665-016-2287-x
10.1007/s11665-016-2301-3

10.1007/s11665-016-2330-y
10.1007/s11665-016-2377-9
10.1007/s11665-016-2391-y
10.1007/s11665-016-2412-x
10.1007/s11665-016-2414-8
10.1007/s11665-016-2417-5
10.1007/s11665-016-2451-3
10.1007/s11665-016-2457-x
10.1007/s11665-016-2462-0
10.1007/s11665-016-2487-4
10.1007/s11665-016-2491-8
10.1007/s11665-016-2492-7
10.1007/s11665-017-2547-4
10.1007/s11665-017-2550-9
10.1007/s11665-017-2551-8
10.1007/s11665-017-2562-5
10.1007/s11665-017-2570-5
10.1007/s11665-017-2586-x
10.1007/s11665-017-2594-x
10.1007/s11665-017-2601-2
10.1007/s11665-017-2620-z
10.1007/s11665-017-2628-4
10.1007/s11665-017-2630-x
10.1007/s11665-017-2631-9
10.1007/s11665-017-2646-2
10.1007/s11665-017-2654-2
10.1007/s11665-017-2675-x
10.1007/s11665-017-2695-6
10.1007/s11665-017-2701-z
10.1007/s11665-017-2718-3
10.1007/s11665-017-2747-y
10.1007/s11665-017-2749-9
10.1007/s11665-017-2757-9
10.1007/s11665-017-2767-7
10.1007/s11665-017-2774-8
10.1007/s11665-017-2810-8
10.1007/s11665-017-2824-2
10.1007/s11665-017-2866-5
10.1007/s11665-017-2954-6
10.1007/s11665-017-2984-0
10.1007/s11665-017-2990-2
10.1007/s11665-017-2991-1
10.1007/s11665-017-2995-x
10.1007/s11665-017-3005-z
10.1007/s11665-017-3008-9
10.1007/s11665-017-3009-8
10.1007/s11665-017-3017-8
10.1007/s11665-017-3030-y
10.1007/s11665-017-3042-7
10.1007/s11665-017-3045-4
10.1007/s11665-017-3046-3
10.1007/s11665-017-3068-x
10.1007/s11665-017-3084-x
10.1007/s11665-018-3127-y

10.1007/s11665-018-3176-2
10.1007/s11665-018-3193-1
10.1007/s11665-018-3220-2
10.1007/s11665-018-3254-5
10.1007/s11665-018-3270-5
10.1007/s11665-018-3274-1
10.1007/s11665-018-3302-1
10.1007/s11665-018-3309-7
10.1007/s11665-018-3325-7
10.1007/s11665-018-3331-9
10.1007/s11665-018-3335-5
10.1007/s11665-018-3337-3
10.1007/s11665-018-3345-3
10.1007/s11665-018-3372-0
10.1007/s11665-018-3440-5
10.1007/s11665-018-3446-z
10.1007/s11665-018-3447-y
10.1007/s11665-018-3501-9
10.1007/s11665-018-3527-z
10.1007/s11665-018-3572-7
10.1007/s11665-018-3592-3
10.1007/s11665-018-3594-1
10.1007/s11665-018-3612-3
10.1007/s11665-018-3620-3
10.1007/s11665-018-3621-2
10.1007/s11665-018-3641-y
10.1007/s11665-018-3668-0
10.1007/s11665-018-3699-6
10.1007/s11665-018-3722-y
10.1007/s11665-018-3728-5
10.1007/s11665-018-3732-9
10.1007/s11665-018-3761-4
10.1007/s11665-018-3799-3
10.1007/s11665-018-3817-5
10.1007/s11665-018-3818-4
10.1007/s11665-018-3845-1
10.1007/s11665-018-3853-1
10.1007/s11665-019-03910-w
10.1007/s11665-019-03919-1
10.1007/s11665-019-03950-2
10.1007/s11665-019-03974-8
10.1007/s11665-019-03980-w
10.1007/s11665-019-03994-4
10.1007/s11665-019-03996-2
10.1007/s11665-019-04014-1
10.1007/s11665-019-04016-z
10.1007/s11665-019-04098-9
10.1007/s11665-019-04103-1
10.1007/s11665-019-04107-x
10.1007/s11665-019-04124-w
10.1007/s11665-019-04164-2
10.1007/s11665-019-04176-y
10.1007/s11665-019-04179-9
10.1007/s11665-019-04195-9

10.1007/s11665-019-04222-9
10.1007/s11665-019-04230-9
10.1007/s11665-019-04235-4
10.1007/s11665-019-04238-1
10.1007/s11665-019-04267-w
10.1007/s11665-019-04326-2
10.1007/s11665-019-04328-0
10.1007/s11665-019-04370-y
10.1007/s11665-019-04383-7
10.1007/s11665-019-04385-5
10.1007/s11665-019-04419-y
10.1007/s11665-019-04443-y
10.1007/s11665-019-04459-4
10.1007/s11665-019-04468-3
10.1007/s11665-019-04475-4
10.1007/s11665-019-04515-z
10.1007/s11665-019-04525-x
10.1007/s11665-019-3858-4
10.1007/s11665-019-3866-4
10.1007/s11665-019-3886-0
10.1007/s11665-020-04583-6
10.1007/s11665-020-04617-z
10.1007/s11665-020-04620-4
10.1007/s11665-020-04660-w
10.1007/s11665-020-04678-0
10.1007/s11665-020-04687-z
10.1007/s11665-020-04754-5
10.1007/s11665-997-0018-z
10.1007/s11665-997-0055-7
10.1007/s11665-997-0125-x
10.1007/s11666-007-9106-8
10.1007/s11666-007-9142-4
10.1007/s11666-007-9150-4
10.1007/s11666-008-9167-3
10.1007/s11666-008-9225-x
10.1007/s11666-008-9277-y
10.1007/s11666-009-9300-y
10.1007/s11666-010-9534-8
10.1007/s11666-011-9647-8
10.1007/s11666-011-9657-6
10.1007/s11666-011-9705-2
10.1007/s11666-012-9771-0
10.1007/s11666-012-9811-9
10.1007/s11666-013-9883-1
10.1007/s11666-013-9887-x
10.1007/s11666-013-9965-0
10.1007/s11666-014-0077-2
10.1007/s11666-014-0102-5
10.1007/s11666-014-0155-5
10.1007/s11666-014-0192-0
10.1007/s11666-014-0209-8
10.1007/s11666-014-0213-z
10.1007/s11666-015-0222-6
10.1007/s11666-015-0235-1

10.1007/s11666-015-0237-z
10.1007/s11666-015-0254-y
10.1007/s11666-015-0265-8
10.1007/s11666-015-0287-2
10.1007/s11666-015-0293-4
10.1007/s11666-015-0299-y
10.1007/s11666-015-0321-4
10.1007/s11666-015-0344-x
10.1007/s11666-015-0357-5
10.1007/s11666-015-0365-5
10.1007/s11666-015-0374-4
10.1007/s11666-016-0433-5
10.1007/s11666-017-0554-5
10.1007/s11666-017-0565-2
10.1007/s11666-017-0572-3
10.1007/s11666-017-0576-z
10.1007/s11666-017-0593-y
10.1007/s11666-017-0612-z
10.1007/s11666-017-0629-3
10.1007/s11666-017-0649-z
10.1007/s11666-018-0689-z
10.1007/s11666-018-0701-7
10.1007/s11666-018-0752-9
10.1007/s11666-018-0803-2
10.1007/s11666-019-00835-7
10.1007/s11666-019-00865-1
10.1007/s11666-019-00936-3
10.1007/s11668-006-9002-4
10.1007/s11668-007-9045-1
10.1007/s11668-007-9077-6
10.1007/s11668-008-9140-y
10.1007/s11668-012-9571-3
10.1007/s11668-012-9601-1
10.1007/s11668-013-9667-4
10.1007/s11668-016-0129-7
10.1007/s11668-016-0154-6
10.1007/s11668-017-0271-x
10.1007/s11668-018-0408-6
10.1007/s11668-018-0487-4
10.1007/s11668-018-0499-0
10.1007/s11668-018-0530-5
10.1007/s11668-018-0560-z
10.1007/s11669-006-9001-x
10.1007/s11669-006-9009-2
10.1007/s11669-007-9199-2
10.1007/s11669-008-9258-3
10.1007/s11669-009-9499-9
10.1007/s11669-009-9533-y
10.1007/s11669-009-9557-3
10.1007/s11669-009-9567-1
10.1007/s11669-011-9853-6
10.1007/s11669-011-9873-2
10.1007/s11669-013-0237-y
10.1007/s11669-013-0274-6

10.1007/s11669-014-0292-z
10.1007/s11669-014-0327-5
10.1007/s11669-014-0346-2
10.1007/s11669-015-0390-6
10.1007/s11669-015-0416-0
10.1007/s11669-015-0444-9
10.1007/s11669-015-0445-8
10.1007/s11669-015-0447-6
10.1007/s11669-016-0461-3
10.1007/s11669-016-0481-z
10.1007/s11669-016-0486-7
10.1007/s11669-016-0490-y
10.1007/s11669-016-0513-8
10.1007/s11669-017-0520-4
10.1007/s11669-018-0620-9
10.1007/s11669-018-0657-9
10.1007/s11669-018-0699-z
10.1007/s11669-019-00742-y
10.1007/s11669-019-00753-9
10.1007/s11669-019-00772-6
10.1007/s11669-019-00775-3
10.1007/s11669-020-00787-4
10.1007/s11669-020-00795-4
10.1007/s11705-018-1720-0
10.1007/s11706-007-0036-7
10.1007/s11706-011-0126-4
10.1007/s11706-015-0277-9
10.1007/s11740-016-0690-7
10.1007/s11771-008-0108-6
10.1007/s11771-011-0653-2
10.1007/s11771-013-1453-7
10.1007/s11771-013-1858-3
10.1007/s11771-014-2011-7
10.1007/s11771-016-3179-9
10.1007/s11771-018-3711-1
10.1007/s11771-018-3848-y
10.1007/s11771-019-4070-2
10.1007/s11771-019-4112-9
10.1007/s11837-000-0109-x
10.1007/s11837-000-0112-2
10.1007/s11837-000-0113-1
10.1007/s11837-000-0114-0
10.1007/s11837-001-0080-1
10.1007/s11837-003-0157-0
10.1007/s11837-003-0158-z
10.1007/s11837-003-0188-6
10.1007/s11837-004-0040-7
10.1007/s11837-004-0073-y
10.1007/s11837-004-0198-z
10.1007/s11837-004-0199-y
10.1007/s11837-004-0200-9
10.1007/s11837-004-0201-8
10.1007/s11837-004-0202-7
10.1007/s11837-004-0251-y

10.1007/s11837-005-0082-5
10.1007/s11837-005-0094-1
10.1007/s11837-005-0232-9
10.1007/s11837-005-0233-8
10.1007/s11837-005-0234-7
10.1007/s11837-006-0064-2
10.1007/s11837-006-0066-0
10.1007/s11837-006-0067-z
10.1007/s11837-006-0069-x
10.1007/s11837-006-0079-8
10.1007/s11837-006-0226-2
10.1007/s11837-008-0085-0
10.1007/s11837-008-0086-z
10.1007/s11837-008-0087-y
10.1007/s11837-008-0088-x
10.1007/s11837-008-0089-9
10.1007/s11837-008-0090-3
10.1007/s11837-009-0026-6
10.1007/s11837-009-0103-x
10.1007/s11837-009-0104-9
10.1007/s11837-009-0106-7
10.1007/s11837-009-0143-2
10.1007/s11837-010-0074-y
10.1007/s11837-010-0150-3
10.1007/s11837-010-0153-0
10.1007/s11837-010-0154-z
10.1007/s11837-010-0155-y
10.1007/s11837-010-0156-x
10.1007/s11837-010-0177-5
10.1007/s11837-010-0183-7
10.1007/s11837-011-0224-x
10.1007/s11837-012-0234-3
10.1007/s11837-012-0241-4
10.1007/s11837-012-0246-z
10.1007/s11837-012-0474-2
10.1007/s11837-012-0475-1
10.1007/s11837-012-0494-y
10.1007/s11837-013-0551-1
10.1007/s11837-013-0612-5
10.1007/s11837-013-0680-6
10.1007/s11837-013-0753-6
10.1007/s11837-014-0935-x
10.1007/s11837-014-0938-7
10.1007/s11837-014-0998-8
10.1007/s11837-014-1066-0
10.1007/s11837-014-1096-7
10.1007/s11837-014-1109-6
10.1007/s11837-014-1157-y
10.1007/s11837-014-1175-9
10.1007/s11837-014-1181-y
10.1007/s11837-014-1184-8
10.1007/s11837-014-1196-4
10.1007/s11837-014-1239-x
10.1007/s11837-015-1399-3

10.1007/s11837-015-1414-8
10.1007/s11837-015-1430-8
10.1007/s11837-015-1455-z
10.1007/s11837-015-1484-7
10.1007/s11837-015-1517-2
10.1007/s11837-015-1523-4
10.1007/s11837-015-1560-z
10.1007/s11837-015-1628-9
10.1007/s11837-015-1635-x
10.1007/s11837-015-1639-6
10.1007/s11837-015-1672-5
10.1007/s11837-015-1690-3
10.1007/s11837-015-1764-2
10.1007/s11837-015-1772-2
10.1007/s11837-016-1812-6
10.1007/s11837-016-2019-6
10.1007/s11837-016-2072-1
10.1007/s11837-016-2096-6
10.1007/s11837-016-2097-5
10.1007/s11837-016-2100-1
10.1007/s11837-016-2225-2
10.1007/s11837-017-2264-3
10.1007/s11837-017-2300-3
10.1007/s11837-017-2307-9
10.1007/s11837-017-2403-x
10.1007/s11837-017-2582-5
10.1007/s11837-017-2638-6
10.1007/s11837-017-2642-x
10.1007/s11837-017-2677-z
10.1007/s11837-017-2703-1
10.1007/s11837-017-2706-y
10.1007/s11837-018-2802-7
10.1007/s11837-018-2818-z
10.1007/s11837-018-2866-4
10.1007/s11837-018-2950-9
10.1007/s11837-018-3100-0
10.1007/s11837-018-3109-4
10.1007/s11837-018-3114-7
10.1007/s11837-018-3134-3
10.1007/s11837-019-03550-4
10.1007/s11837-019-03580-y
10.1007/s11837-019-03621-6
10.1007/s11837-019-03694-3
10.1007/s11837-019-03857-2
10.1007/s11837-020-04012-y
10.1007/s11837-020-04054-2
10.1007/s11837-020-04080-0
10.1007/s11837-020-04081-z
10.1007/s11837-020-04119-2
10.1007/s11837-998-0248-z
10.1007/s11837-998-0310-x
10.1007/s11837-999-0004-z
10.1007/s11837-999-0006-x
10.1007/s11837-999-0007-9

10.1007/s11837-999-0074-y
10.1007/s11837-999-0092-9
10.1007/s11998-008-9103-y
10.1007/s11998-008-9106-8
10.1007/s11998-010-9277-y
10.1007/s12034-010-0046-4
10.1007/s12034-010-0074-0
10.1007/s12034-011-0070-z
10.1007/s12034-011-0074-8
10.1007/s12034-011-0110-8
10.1007/s12034-014-0110-6
10.1007/s12034-015-0867-2
10.1007/s12034-015-0986-9
10.1007/s12034-019-1984-0
10.1007/s12034-020-2070-3
10.1007/s12046-009-0009-x
10.1007/s12046-013-0169-6
10.1007/s12046-017-0647-3
10.1007/s12046-018-0850-x
10.1007/s12046-018-1047-z
10.1007/s12046-019-1207-9
10.1007/s12204-011-1114-7
10.1007/s12204-011-1143-2
10.1007/s12206-010-0307-6
10.1007/s12206-011-0426-8
10.1007/s12206-012-0505-5
10.1007/s12206-012-0506-4
10.1007/s12206-012-0508-2
10.1007/s12206-012-0523-3
10.1007/s12206-015-0131-0
10.1007/s12206-015-0345-1
10.1007/s12206-015-0911-6
10.1007/s12206-016-0217-3
10.1007/s12206-016-0233-3
10.1007/s12206-016-0907-x
10.1007/s12206-016-1027-3
10.1007/s12206-016-1218-y
10.1007/s12206-017-0133-1
10.1007/s12206-017-0447-z
10.1007/s12206-017-0629-8
10.1007/s12206-017-0926-2
10.1007/s12206-017-1040-1
10.1007/s12206-018-0311-9
10.1007/s12206-018-0518-9
10.1007/s12206-018-1010-2
10.1007/s12206-018-1013-z
10.1007/s12206-018-1023-x
10.1007/s12206-018-1201-x
10.1007/s12206-019-0641-2
10.1007/s12206-019-0818-8
10.1007/s12206-020-0227-z
10.1007/s12217-011-9297-y
10.1007/s12217-015-9481-6
10.1007/s12239-012-0061-0

10.1007/s12289-008-0052-x
10.1007/s12289-014-1163-1
10.1007/s12289-015-1279-y
10.1007/s12289-017-1361-8
10.1007/s12289-018-01461-4
10.1007/s12289-018-1438-z
10.1007/s12289-018-1446-z
10.1007/s12289-019-01483-6
10.1007/s12289-019-01486-3
10.1007/s12289-020-01548-x
10.1007/s12540-009-0051-6
10.1007/s12540-009-0391-2
10.1007/s12540-012-2012-8
10.1007/s12540-012-5008-5
10.1007/s12540-012-6002-7
10.1007/s12540-012-6005-4
10.1007/s12540-013-5003-5
10.1007/s12540-013-5026-y
10.1007/s12540-014-3004-7
10.1007/s12540-014-3017-2
10.1007/s12540-014-3018-1
10.1007/s12540-015-4230-3
10.1007/s12540-015-4245-9
10.1007/s12540-015-4627-z
10.1007/s12540-015-5296-7
10.1007/s12540-015-5497-0
10.1007/s12540-016-6007-8
10.1007/s12540-016-6110-x
10.1007/s12540-016-6218-z
10.1007/s12540-016-6305-1
10.1007/s12540-016-6345-6
10.1007/s12540-017-6109-y
10.1007/s12540-017-6264-1
10.1007/s12540-017-6313-9
10.1007/s12540-017-6388-3
10.1007/s12540-017-6516-0
10.1007/s12540-017-6526-y
10.1007/s12540-017-6846-y
10.1007/s12540-017-7052-7
10.1007/s12540-017-7089-7
10.1007/s12540-017-7359-4
10.1007/s12540-018-0056-0
10.1007/s12540-018-0073-z
10.1007/s12540-018-0106-7
10.1007/s12540-018-0121-8
10.1007/s12540-019-00265-8
10.1007/s12540-019-00274-7
10.1007/s12540-019-00289-0
10.1007/s12540-019-00312-4
10.1007/s12540-019-00316-0
10.1007/s12540-019-00342-y
10.1007/s12540-019-00476-z
10.1007/s12540-019-00483-0
10.1007/s12540-019-00534-6

10.1007/s12540-019-00571-1
10.1007/s12540-020-00615-x
10.1007/s12540-020-00616-w
10.1007/s12540-020-00655-3
10.1007/s12541-009-0040-1
10.1007/s12541-011-0043-6
10.1007/s12541-013-0152-5
10.1007/s12541-014-0420-z
10.1007/s12541-014-0494-7
10.1007/s12541-014-0526-3
10.1007/s12541-014-0626-0
10.1007/s12541-015-0041-1
10.1007/s12541-015-0102-5
10.1007/s12541-015-0220-0
10.1007/s12541-016-0143-4
10.1007/s12541-017-0009-4
10.1007/s12541-017-0033-4
10.1007/s12541-017-0067-7
10.1007/s12541-017-0197-y
10.1007/s12541-018-0047-6
10.1007/s12541-018-0137-5
10.1007/s12541-018-0196-7
10.1007/s12541-019-00025-z
10.1007/s12541-019-00093-1
10.1007/s12541-019-00145-6
10.1007/s12567-016-0124-6
10.1007/s12572-011-0026-y
10.1007/s12598-009-0038-y
10.1007/s12598-009-0039-x
10.1007/s12598-009-0121-4
10.1007/s12598-010-0055-x
10.1007/s12598-010-0140-1
10.1007/s12598-011-0201-0
10.1007/s12598-011-0202-z
10.1007/s12598-011-0294-5
10.1007/s12598-011-0308-3
10.1007/s12598-011-0309-2
10.1007/s12598-011-0310-9
10.1007/s12598-011-0311-8
10.1007/s12598-011-0312-7
10.1007/s12598-011-0313-6
10.1007/s12598-011-0314-5
10.1007/s12598-011-0319-0
10.1007/s12598-011-0320-7
10.1007/s12598-011-0322-5
10.1007/s12598-011-0323-4
10.1007/s12598-011-0324-3
10.1007/s12598-011-0325-2
10.1007/s12598-011-0327-0
10.1007/s12598-011-0328-z
10.1007/s12598-011-0362-z
10.1007/s12598-011-0363-z
10.1007/s12598-012-0466-y
10.1007/s12598-012-0493-8

10.1007/s12598-012-0494-7
10.1007/s12598-012-0495-6
10.1007/s12598-012-0554-z
10.1007/s12598-013-0072-7
10.1007/s12598-013-0106-1
10.1007/s12598-013-0199-6
10.1007/s12598-014-0254-y
10.1007/s12598-014-0256-9
10.1007/s12598-014-0340-1
10.1007/s12598-014-0410-4
10.1007/s12598-014-0419-8
10.1007/s12598-015-0482-9
10.1007/s12598-015-0487-4
10.1007/s12598-015-0529-y
10.1007/s12598-015-0544-z
10.1007/s12598-015-0551-0
10.1007/s12598-015-0597-z
10.1007/s12598-015-0646-7
10.1007/s12598-015-0659-2
10.1007/s12598-016-0713-8
10.1007/s12598-016-0718-3
10.1007/s12598-016-0751-2
10.1007/s12598-016-0754-z
10.1007/s12598-016-0755-y
10.1007/s12598-016-0761-0
10.1007/s12598-016-0764-x
10.1007/s12598-016-0809-1
10.1007/s12598-016-0823-3
10.1007/s12598-016-0848-7
10.1007/s12598-016-0862-9
10.1007/s12598-016-0867-4
10.1007/s12598-017-0877-x
10.1007/s12598-017-0931-8
10.1007/s12598-018-1016-z
10.1007/s12598-018-1077-z
10.1007/s12598-018-1093-z
10.1007/s12598-018-1123-x
10.1007/s12598-018-1171-2
10.1007/s12598-019-01292-5
10.1007/s12598-020-01393-6
10.1007/s12613-010-0342-4
10.1007/s12613-012-0635-x
10.1007/s12613-012-0636-9
10.1007/s12613-012-0661-8
10.1007/s12613-013-0691-x
10.1007/s12613-013-0807-3
10.1007/s12613-013-0852-y
10.1007/s12613-013-0853-x
10.1007/s12613-014-0865-1
10.1007/s12613-014-0930-9
10.1007/s12613-014-1017-3
10.1007/s12613-016-1214-3
10.1007/s12613-016-1266-4
10.1007/s12613-016-1281-5

10.1007/s12613-016-1326-9
10.1007/s12613-016-1362-5
10.1007/s12613-017-1375-8
10.1007/s12613-017-1403-8
10.1007/s12613-017-1424-3
10.1007/s12613-017-1448-8
10.1007/s12613-017-1451-0
10.1007/s12613-017-1461-y
10.1007/s12613-018-1617-4
10.1007/s12613-018-1627-2
10.1007/s12613-018-1643-2
10.1007/s12613-018-1654-z
10.1007/s12613-019-1726-8
10.1007/s12613-019-1727-7
10.1007/s12613-019-1730-z
10.1007/s12613-019-1756-2
10.1007/s12613-019-1757-1
10.1007/s12613-019-1768-y
10.1007/s12613-019-1774-0
10.1007/s12613-019-1802-0
10.1007/s12613-019-1817-6
10.1007/s12613-019-1946-y
10.1007/s12613-201-0422-5
10.1007/s12633-017-9549-6
10.1007/s12633-017-9568-3
10.1007/s12633-017-9728-5
10.1007/s12633-018-9952-7
10.1007/s12665-013-2646-y
10.1007/s12666-008-0008-3
10.1007/s12666-008-0064-8
10.1007/s12666-009-0034-9
10.1007/s12666-009-0089-7
10.1007/s12666-011-0006-8
10.1007/s12666-011-0011-y
10.1007/s12666-011-0115-4
10.1007/s12666-012-0160-7
10.1007/s12666-013-0317-z
10.1007/s12666-013-0362-7
10.1007/s12666-014-0383-x
10.1007/s12666-014-0398-3
10.1007/s12666-015-0550-8
10.1007/s12666-015-0591-z
10.1007/s12666-015-0605-x
10.1007/s12666-015-0619-4
10.1007/s12666-015-0641-6
10.1007/s12666-015-0712-8
10.1007/s12666-015-0760-0
10.1007/s12666-015-0768-5
10.1007/s12666-015-0772-9
10.1007/s12666-015-0774-7
10.1007/s12666-015-0802-7
10.1007/s12666-015-0817-0
10.1007/s12666-016-0848-1
10.1007/s12666-016-0852-5

10.1007/s12666-016-0894-8
10.1007/s12666-016-1031-4
10.1007/s12666-017-1045-6
10.1007/s12666-017-1063-4
10.1007/s12666-017-1077-y
10.1007/s12666-017-1087-9
10.1007/s12666-017-1120-z
10.1007/s12666-017-1139-1
10.1007/s12666-017-1147-1
10.1007/s12666-017-1201-z
10.1007/s12666-017-1217-4
10.1007/s12666-017-1234-3
10.1007/s12666-018-1291-2
10.1007/s12666-018-1369-x
10.1007/s12666-018-1371-3
10.1007/s12666-018-1390-0
10.1007/s12666-018-1391-z
10.1007/s12666-018-1394-9
10.1007/s12666-018-1395-8
10.1007/s12666-018-1474-x
10.1007/s12666-018-1532-4
10.1007/s12666-018-1543-1
10.1007/s12666-019-01579-2
10.1007/s12666-019-01693-1
10.1007/s12666-019-01713-0
10.1007/s12666-019-01857-z
10.1007/s12666-020-01921-z
10.1007/s12666-020-01954-4
10.1007/s12678-017-0368-8
10.1007/s13296-016-0003-1
10.1007/s13369-013-0536-y
10.1007/s13369-014-1354-6
10.1007/s13369-015-1766-y
10.1007/s13369-016-2305-1
10.1007/s13369-017-2422-5
10.1007/s13369-017-2594-z
10.1007/s13369-018-3287-y
10.1007/s13369-018-3629-9
10.1007/s13369-019-03783-0
10.1007/s13369-019-03963-y
10.1007/s13369-019-04111-2
10.1007/s13369-019-04112-1
10.1007/s13369-019-04171-4
10.1007/s13369-019-04240-8
10.1007/s40010-019-00642-3
10.1007/s40032-015-0188-7
10.1007/s40032-017-0411-9
10.1007/s40145-017-0234-4
10.1007/s40192-017-0090-7
10.1007/s40192-017-0101-8
10.1007/s40192-017-0103-6
10.1007/s40192-018-0111-1
10.1007/s40192-018-0115-x
10.1007/s40192-018-0119-6

10.1007/s40192-019-00129-4
10.1007/s40192-019-00130-x
10.1007/s40192-019-00134-7
10.1007/s40192-019-00137-4
10.1007/s40192-019-00148-1
10.1007/s40192-019-00149-0
10.1007/s40192-019-00157-0
10.1007/s40192-019-00161-4
10.1007/s40192-019-0124-4
10.1007/s40192-020-00170-8
10.1007/s40194-014-0144-9
10.1007/s40194-015-0254-z
10.1007/s40194-015-0266-8
10.1007/s40194-016-0349-1
10.1007/s40194-016-0388-7
10.1007/s40194-016-0415-8
10.1007/s40194-016-0420-y
10.1007/s40194-017-0473-6
10.1007/s40194-017-0477-2
10.1007/s40194-017-0503-4
10.1007/s40194-017-0508-z
10.1007/s40194-017-0514-1
10.1007/s40194-018-0549-y
10.1007/s40194-018-0579-5
10.1007/s40194-018-0662-y
10.1007/s40194-019-00740-1
10.1007/s40194-019-00760-x
10.1007/s40194-019-00761-w
10.1007/s40194-019-00784-3
10.1007/s40194-019-00808-y
10.1007/s40194-019-00824-y
10.1007/s40194-020-00891-6
10.1007/s40195-012-0249-3
10.1007/s40195-013-0028-9
10.1007/s40195-013-0199-4
10.1007/s40195-013-0252-3
10.1007/s40195-014-0037-3
10.1007/s40195-014-0047-1
10.1007/s40195-014-0069-8
10.1007/s40195-014-0090-y
10.1007/s40195-014-0108-5
10.1007/s40195-014-0116-5
10.1007/s40195-014-0119-2
10.1007/s40195-014-0121-8
10.1007/s40195-014-0149-9
10.1007/s40195-014-0150-3
10.1007/s40195-014-0169-5
10.1007/s40195-014-0179-3
10.1007/s40195-014-0185-5
10.1007/s40195-014-0186-4
10.1007/s40195-015-0211-2
10.1007/s40195-015-0230-z
10.1007/s40195-015-0241-9
10.1007/s40195-015-0277-x

10.1007/s40195-015-0298-5
10.1007/s40195-015-0323-8
10.1007/s40195-016-0414-1
10.1007/s40195-016-0420-3
10.1007/s40195-016-0442-x
10.1007/s40195-016-0494-y
10.1007/s40195-016-0499-6
10.1007/s40195-016-0500-4
10.1007/s40195-016-0514-y
10.1007/s40195-017-0541-3
10.1007/s40195-017-0546-y
10.1007/s40195-017-0563-x
10.1007/s40195-017-0566-7
10.1007/s40195-017-0596-1
10.1007/s40195-017-0598-z
10.1007/s40195-017-0607-2
10.1007/s40195-017-0614-3
10.1007/s40195-017-0634-z
10.1007/s40195-017-0635-y
10.1007/s40195-017-0646-8
10.1007/s40195-017-0669-1
10.1007/s40195-017-0676-2
10.1007/s40195-017-0678-0
10.1007/s40195-017-0679-z
10.1007/s40195-017-0681-5
10.1007/s40195-017-0693-1
10.1007/s40195-017-0697-x
10.1007/s40195-018-0705-9
10.1007/s40195-018-0716-6
10.1007/s40195-018-0724-6
10.1007/s40195-018-0729-1
10.1007/s40195-018-0770-0
10.1007/s40195-018-0779-4
10.1007/s40195-018-0786-5
10.1007/s40195-018-0790-9
10.1007/s40195-018-0814-5
10.1007/s40195-018-0815-4
10.1007/s40195-018-0827-0
10.1007/s40195-018-0834-1
10.1007/s40195-018-0837-y
10.1007/s40195-018-0839-9
10.1007/s40195-018-0861-y
10.1007/s40195-019-00891-6
10.1007/s40195-019-00894-3
10.1007/s40195-019-00918-y
10.1007/s40195-019-00931-1
10.1007/s40195-019-00936-w
10.1007/s40195-019-00959-3
10.1007/s40195-019-00986-0
10.1007/s40195-020-01004-4
10.1007/s40195-020-01009-z
10.1007/s40195-020-01024-0
10.1007/s40195-020-01030-2
10.1007/s40430-015-0422-5

10.1007/s40430-016-0568-9
10.1007/s40430-016-0659-7
10.1007/s40430-017-0734-8
10.1007/s40430-018-1042-7
10.1007/s40430-018-1045-4
10.1007/s40430-018-1156-y
10.1007/s40430-018-1257-7
10.1007/s40430-018-1294-2
10.1007/s40430-018-1302-6
10.1007/s40430-018-1378-z
10.1007/s40430-018-1408-x
10.1007/s40430-018-1526-5
10.1007/s40430-019-1669-z
10.1007/s40430-019-1703-1
10.1007/s40430-019-1809-5
10.1007/s40430-019-1813-9
10.1007/s40430-019-2031-1
10.1007/s40430-020-2180-2
10.1007/s40436-013-0050-1
10.1007/s40436-017-0185-6
10.1007/s40436-017-0192-7
10.1007/s40436-018-0242-9
10.1007/s40436-019-00259-0
10.1007/s40516-017-0043-1
10.1007/s40516-017-0050-2
10.1007/s40684-018-0027-4
10.1007/s40684-018-0033-6
10.1007/s40684-019-00125-1
10.1007/s40799-016-0078-9
10.1007/s40799-016-0162-1
10.1007/s40799-017-0221-2
10.1007/s40962-017-0180-5
10.1007/s40962-017-0198-8
10.1007/s40962-018-0220-9
10.1007/s40962-018-0255-y
10.1007/s40962-018-0286-4
10.1007/s40962-019-00338-9
10.1007/s40995-018-0604-y
10.1007/s40997-017-0090-4
10.1007/s40997-018-0197-2
10.1007/s40997-020-00357-6
10.1007/s41230-016-5117-8
10.1007/s41230-016-5124-9
10.1007/s41230-016-6044-4
10.1007/s41230-017-5125-3
10.1007/s41230-017-6063-9
10.1007/s41230-017-6106-2
10.1007/s41230-017-7017-y
10.1007/s41230-017-7146-3
10.1007/s41230-017-7150-7
10.1007/s41230-018-7048-z
10.1007/s41230-019-8080-3
10.1007/s41230-019-8113-y
10.1007/s41230-019-8135-5

10.1007/s41230-019-8142-6
10.1007/s41230-019-8161-3
10.1007/s41230-019-9072-z
10.1007/s41230-020-9100-z
10.1007/s41779-016-0012-8
10.1007/s41779-017-0105-z
10.1007/s42114-017-0010-5
10.1007/s42243-018-00220-8
10.1007/s42243-018-0076-5
10.1007/s42243-018-0214-0
10.1007/s42243-018-0219-8
10.1007/s42243-020-00379-z
10.1007/s42243-020-00391-3

10.1016/j.corsci.2016.02.029
10.1016/j.msea.2008.12.034
10.1016/j.powtec.2019.07.063
10.1016/S0043-1648(03)00163-7
10.1016/j.msea.2004.12.037
10.1016/j.applthermaleng.2016.11.039
10.1016/j.corsci.2004.06.023
10.1016/S0927-0256(03)00102-2
10.1016/j.ijfatigue.2014.04.012
10.1016/S0921-5093(01)00989-3
10.1016/j.matdes.2015.04.004
10.1016/0956-716x(90)90197-o
10.1016/j.mtcomm.2019.02.004
10.1016/1044-5803(93)90062-z
10.1016/j.apmt.2019.04.001
10.1016/j.corsci.2015.12.012
10.1016/j.dib.2018.03.084
10.1016/j.jallcom.2012.02.166
10.1016/j.jallcom.2016.01.205
10.1016/0956-7151(93)90218-h
10.1016/0010-938x(93)90097-z
10.1016/j.ceramint.2017.03.084
10.1016/S1000-9361(11)60182-9
10.1016/1359-6462(95)00464-5
10.1016/j.scriptamat.2018.10.004
10.1016/j.surfcoat.2004.08.066
10.1016/j.matchar.2018.04.027
10.1016/j.ijmachtools.2014.11.010
10.1016/j.msea.2015.07.077
10.1016/j.prostr.2019.12.058
10.1016/0025-5416(83)90001-0
10.1016/j.surfcoat.2016.12.078
10.1016/j.scriptamat.2014.10.025
10.1016/0376-4583(85)90115-3
10.1016/j.msea.2015.05.039
10.1016/j.corsci.2019.04.023
10.1016/j.ijfatigue.2018.02.035
10.1016/j.ijfatigue.2015.08.016
10.1016/j.actaastro.2017.09.015
10.1016/j.msea.2008.08.052
10.1016/j.corsci.2010.07.011
10.1016/j.jmapro.2017.06.011
10.1016/j.matchar.2014.06.016
10.1016/0022-5088(76)90159-4
10.1016/j.jallcom.2017.01.288
10.1016/j.surfcoat.2016.05.015
10.1016/j.jmatprotec.2011.03.023
10.1016/j.jallcom.2017.07.020
10.1016/j.jallcom.2012.01.142
10.1016/j.ijfatigue.2018.05.008
10.1016/0956-716x(91)90518-6
10.1016/j.jnucmat.2006.03.008
10.1016/j.msea.2006.05.091
10.1016/j.jallcom.2013.01.156

10.1016/S0966-9795(03)00023-2
10.1016/j.advengsoft.2010.12.002
10.1016/j.jallcom.2007.07.067
10.1016/j.matchar.2019.06.013
10.1016/S0257-8972(99)00603-9
10.1016/j.msea.2006.05.130
10.1016/0010-938x(89)90113-3
10.1016/j.msea.2010.01.001
10.1016/j.apsusc.2012.12.012
10.1016/j.ijfatigue.2019.05.022
10.1016/S0257-8972(96)02920-9
10.1016/j.ijfatigue.2012.02.010
10.1016/j.surfcoat.2007.06.056
10.1016/j.scriptamat.2017.09.036
10.1016/0011-2275(84)90040-7
10.1016/j.matlet.2007.05.068
10.1016/S0921-5093(00)01788-3
10.1016/j.triboint.2014.01.011
10.1016/j.ijfatigue.2010.01.015
10.1016/j.surfcoat.2005.07.082
10.1016/S1359-6454(99)00093-2
10.1016/j.surfcoat.2005.12.034
10.1016/j.msea.2014.03.048
10.1016/j.msea.2005.08.126
10.1016/j.corsci.2012.10.012
10.1016/0022-3115(85)90034-0
10.1016/0001-6160(81)90129-2
10.1016/S1359-6454(97)00346-7
10.1016/j.msea.2005.07.016
10.1016/S1359-6462(99)00158-X
10.1016/j.msea.2012.12.074
10.1016/0956-716x(95)00352-v
10.1016/j.msea.2008.09.017
10.1016/j.jmatprotec.2008.05.015
10.1016/j.msea.2005.06.001
10.1016/j.jmatprotec.2017.05.045
10.1016/j.corsci.2010.04.007
10.1016/0036-9748(85)90312-6
10.1016/S0921-5093(99)00176-8
10.1016/j.matlet.2015.10.136
10.1016/j.calphad.2018.01.001
10.1016/j.actamat.2013.03.052
10.1016/j.vacuum.2008.03.057
10.1016/j.intermet.2004.12.006
10.1016/j.corsci.2005.01.006
10.1016/j.engfailanal.2019.104224
10.1016/j.jallcom.2019.05.022
10.1016/j.msea.2010.12.010
10.1016/j.ijfatigue.2007.01.026
10.1016/j.ijplas.2015.12.008
10.1016/j.optlastec.2017.09.022
10.1016/j.jmatprotec.2009.01.012
10.1016/j.jallcom.2008.12.068
10.1016/j.jmapro.2018.03.017

10.1016/S0924-0136(01)01194-3
10.1016/j.mechmat.2015.11.015
10.1016/j.jmatprotec.2017.10.020
10.1016/j.jallcom.2019.06.249
10.1016/j.matdes.2019.108008
10.1016/j.jallcom.2018.11.166
10.1016/j.matdes.2018.08.033
10.1016/j.msea.2015.10.113
10.1016/j.msea.2018.08.022
10.1016/j.surfcoat.2014.10.033
10.1016/j.surfcoat.2006.05.045
10.1016/j.surfcoat.2012.06.093
10.1016/j.ijfatigue.2008.03.017
10.1016/j.matpr.2018.06.421
10.1016/S0921-5093(01)01712-9
10.1016/j.jallcom.2016.10.256
10.1016/S0010-938X(02)00182-8
10.1016/j.jallcom.2019.153521
10.1016/j.msea.2013.01.001
10.1016/j.jallcom.2019.153179
10.1016/j.ijmachtools.2018.12.001
10.1016/j.mechrescom.2015.12.001
10.1016/j.msea.2009.10.036
10.1016/j.msea.2018.05.044
10.1016/0360-3199(93)90175-a
10.1016/j.cja.2018.07.013
10.1016/j.jallcom.2009.02.059
10.1016/j.surfcoat.2005.07.086
10.1016/j.matlet.2016.10.118
10.1016/j.jmps.2005.09.001
10.1016/0013-7944(90)90126-2
10.1016/j.scriptamat.2010.06.019
10.1016/S0142-1123(99)00058-4
10.1016/0043-1648(83)90091-1
10.1016/j.jmatprotec.2006.10.020
10.1016/j.msea.2005.05.032
10.1016/j.intermet.2020.106745
10.1016/0036-9748(80)90099-x
10.1016/j.calphad.2015.02.005
10.1016/j.surfcoat.2006.07.256
10.1016/j.jallcom.2017.09.194
10.1016/j.msea.2018.03.073
10.1016/0167-577x(93)90126-i
10.1016/S0022-3115(00)00723-6
10.1016/0036-9748(85)90257-1
10.1016/j.jclepro.2018.01.003
10.1016/S0026-0657(97)89760-3
10.1016/j.msea.2018.06.102
10.1016/j.jallcom.2009.07.020
10.1016/j.jallcom.2012.08.069
10.1016/j.ijplas.2018.06.011
10.1016/0036-9748(86)90214-0
10.1016/1359-6462(95)00562-5
10.1016/S1000-9361(09)60247-8

10.1016/j.msea.2018.03.020
10.1016/j.tsf.2008.08.006
10.1016/j.jmatprotec.2017.03.018
10.1016/j.comptc.2012.01.021
10.1016/j.optlastec.2017.06.027
10.1016/j.corsci.2010.03.032
10.1016/j.msea.2017.10.098
10.1016/S0749-6419(99)00035-2
10.1016/j.matchar.2016.10.026
10.1016/S0927-0256(96)00079-1
10.1016/S1003-6326(10)60135-5
10.1016/j.pmatsci.2016.11.001
10.1016/j.corsci.2019.108354
10.1016/S0266-3538(97)00177-2
10.1016/j.jallcom.2017.10.263
10.1016/0022-3115(87)90470-3
10.1016/j.matdes.2009.09.020
10.1016/j.ijfatigue.2010.11.003
10.1016/0921-5093(94)90903-2
10.1016/j.matchemphys.2019.121925
10.1016/j.matchemphys.2003.08.014
10.1016/S1003-6326(15)63920-6
10.1016/j.powtec.2017.08.049
10.1016/j.actamat.2016.03.063
10.1016/j.ijfatigue.2012.04.017
10.1016/j.actamat.2016.09.017
10.1016/0013-7944(85)90109-2
10.1016/j.scriptamat.2003.11.009
10.1016/j.jallcom.2019.153337
10.1016/j.msea.2017.03.116
10.1016/j.msea.2018.01.079
10.1016/j.jmst.2018.10.011
10.1016/j.jmrt.2019.12.069
10.1016/j.jallcom.2006.07.051
10.1016/j.corsci.2011.10.021
10.1016/j.jmrt.2020.03.097
10.1016/j.corsci.2017.09.004
10.1016/j.surfcoat.2011.02.005
10.1016/j.proeng.2014.06.273
10.1016/j.jnucmat.2009.06.011
10.1016/j.corsci.2013.04.006
10.1016/0025-5416(84)90266-0
10.1016/S0167-6636(03)00033-4
10.1016/j.jallcom.2012.02.144
10.1016/j.scriptamat.2017.12.009
10.1016/j.msea.2018.09.079
10.1016/j.msea.2003.12.080
10.1016/j.vacuum.2017.01.028
10.1016/j.matchar.2017.11.047
10.1016/j.ultramic.2009.05.008
10.1016/j.matchar.2012.12.003
10.1016/j.matchar.2009.12.013
10.1016/j.msea.2006.08.137
10.1016/j.jallcom.2007.03.063

10.1016/j.surfcoat.2015.03.016
10.1016/S1005-0302(12)60079-6
10.1016/j.mseb.2004.05.034
10.1016/j.ijrmhm.2018.01.018
10.1016/j.actamat.2017.10.041
10.1016/S0921-5093(00)02042-6
10.1016/j.msea.2010.11.080
10.1016/j.compstruct.2018.05.005
10.1016/j.matlet.2016.06.029
10.1016/j.actamat.2017.06.025
10.1016/j.matchar.2019.109875
10.1016/j.camss.2017.07.004
10.1016/j.ijplas.2011.06.003
10.1016/0956-7151(94)90011-6
10.1016/0039-9140(82)80196-3
10.1016/j.surfcoat.2018.02.094
10.1016/j.scriptamat.2012.06.003
10.1016/j.msea.2015.07.007
10.1016/j.jallcom.2020.155086
10.1016/j.matlet.2018.03.081
10.1016/j.ijfatigue.2015.09.006
10.1016/0921-5093(95)09825-9
10.1016/j.scriptamat.2018.11.045
10.1016/S1006-706X(10)60015-5
10.1016/S0257-8972(00)01093-8
10.1016/j.matlet.2017.09.096
10.1016/j.msea.2016.07.018
10.1016/j.engfailanal.2019.07.008
10.1016/j.jallcom.2015.05.068
10.1016/j.matchar.2005.07.003
10.1016/j.ceramint.2017.10.219
10.1016/j.actamat.2016.12.039
10.1016/j.matchar.2019.109813
10.1016/j.ijplas.2010.06.003
10.1016/j.msea.2016.07.070
10.1016/j.jallcom.2012.02.003
10.1016/j.msea.2005.05.120
10.1016/j.msea.2017.02.104
10.1016/j.msea.2014.11.016
10.1016/j.corsci.2018.09.007
10.1016/j.scriptamat.2010.04.033
10.1016/j.ndteint.2010.05.004
10.1016/j.surfcoat.2018.08.070
10.1016/j.addma.2014.12.002
10.1016/j.corsci.2014.09.009
10.1016/j.jmart.2019.05.018
10.1016/S1003-6326(11)60891-1
10.1016/j.matdes.2017.05.044
10.1016/0749-6419(86)90002-1
10.1016/j.jallcom.2018.10.031
10.1016/j.jmatprotec.2015.07.014
10.1016/j.matchar.2019.109946
10.1016/0921-5093(91)90225-c
10.1016/j.matdes.2013.10.023

10.1016/j.msea.2015.06.093
10.1016/j.actamat.2006.05.032
10.1016/j.ijplas.2013.05.004
10.1016/j.jmst.2018.05.003
10.1016/j.msea.2018.01.100
10.1016/j.matlet.2012.09.108
10.1016/j.actamat.2019.12.038
10.1016/S1359-6462(97)00276-5
10.1016/j.surfcoat.2017.12.062
10.1016/j.msea.2012.10.018
10.1016/S0924-0136(01)00596-9
10.1016/0956-716x(92)90287-o
10.1016/0022-3115(85)90461-1
10.1016/j.actamat.2016.10.004
10.1016/j.jmapro.2018.03.036
10.1016/j.matlet.2015.07.120
10.1016/S0921-5093(98)00655-8
10.1016/j.jallcom.2019.152882
10.1016/S1350-6307(00)00043-1
10.1016/j.scriptamat.2018.04.029
10.1016/j.triboint.2017.06.013
10.1016/j.proeng.2013.03.340
10.1016/j.matdes.2018.107553
10.1016/j.jallcom.2019.06.202
10.1016/j.corsci.2009.09.032
10.1016/j.apsusc.2019.143774
10.1016/S1359-6462(98)00322-4
10.1016/j.ijrmhm.2015.08.012
10.1016/j.commatsci.2009.07.014
10.1016/0956-716x(92)90370-t
10.1016/j.apsusc.2016.04.027
10.1016/S1359-6462(97)00409-0
10.1016/0921-5093(92)90415-w
10.1016/j.apsusc.2019.05.334
10.1016/j.matdes.2014.10.020
10.1016/j.msea.2012.12.088
10.1016/j.crhy.2011.10.020
10.1016/j.corsci.2013.11.048
10.1016/S0010-938X(96)00104-7
10.1016/j.simpat.2013.11.009
10.1016/j.surfcoat.2005.04.055
10.1016/j.intermet.2007.05.008
10.1016/S0010-938X(03)00147-1
10.1016/j.matchar.2018.12.018
10.1016/j.jallcom.2017.04.253
10.1016/j.optlastec.2016.01.029
10.1016/j.dib.2017.06.055
10.1016/j.actamat.2012.09.067
10.1016/0921-5093(93)90594-5
10.1016/j.apsusc.2019.07.028
10.1016/0956-7151(94)90466-9
10.1016/j.surfcoat.2019.124923
10.1016/0956-716x(94)90480-4
10.1016/S0927-0256(97)00052-9

10.1016/j.proeng.2017.02.249
10.1016/j.triboint.2019.106144
10.1016/j.msea.2016.09.083
10.1016/j.surfcoat.2014.08.011
10.1016/j.jallcom.2011.02.174
10.1016/0010-938x(92)90006-o
10.1016/j.ijmachtools.2006.07.005
10.1016/j.msea.2017.06.051
10.1016/0022-3115(82)90521-9
10.1016/j.jeurceramsoc.2018.01.028
10.1016/S0013-7944(02)00054-1
10.1016/j.actamat.2014.10.036
10.1016/j.msea.2016.03.036
10.1016/j.msea.2019.05.002
10.1016/S1359-6462(97)00028-6
10.1016/j.msea.2016.05.063
10.1016/j.ceramint.2019.01.208
10.1016/S0921-5093(01)00987-X
10.1016/j.msea.2004.02.062
10.1016/j.jallcom.2016.08.096
10.1016/j.msea.2014.05.022
10.1016/j.commatsci.2012.05.046
10.1016/j.msea.2019.05.081
10.1016/j.msea.2019.138711
10.1016/j.surfcoat.2003.12.004
10.1016/j.addma.2018.04.032
10.1016/j.actamat.2015.08.025
10.1016/j.matdes.2019.107595
10.1016/0045-7949(95)98864-M
10.1016/0956-716x(94)90257-7
10.1016/j.ijfatigue.2019.01.007
10.1016/S0022-3115(03)00013-8
10.1016/0013-7944(85)90012-8
10.1016/j.engfailanal.2019.01.049
10.1016/j.msea.2016.10.124
10.1016/S1359-6462(99)00313-9
10.1016/j.scriptamat.2010.02.015
10.1016/j.corsci.2018.11.017
10.1016/j.ijsolstr.2014.03.037
10.1016/j.msea.2006.08.133
10.1016/j.surfcoat.2015.11.054
10.1016/j.jallcom.2019.04.045
10.1016/S0020-7683(03)00388-3
10.1016/j.corsci.2013.10.022
10.1016/S0921-5093(01)01480-0
10.1016/j.corsci.2009.03.036
10.1016/j.msea.2020.139334
10.1016/j.msea.2017.06.098
10.1016/j.actamat.2011.11.051
10.1016/j.actamat.2012.09.028
10.1016/j.jallcom.2009.10.261
10.1016/j.msea.2019.138825
10.1016/S0026-0657(96)94154-5
10.1016/j.ijpvp.2018.10.013

10.1016/0956-7151(93)90019-o
10.1016/0956-716x(92)90646-v
10.1016/j.jallcom.2019.153041
10.1016/j.actamat.2017.02.028
10.1016/S1003-6326(16)64416-3
10.1016/j.ijhydene.2006.06.058
10.1016/j.jallcom.2007.01.169
10.1016/1044-5803(95)00036-4
10.1016/j.surfcoat.2010.09.042
10.1016/j.actamat.2005.10.016
10.1016/j.actamat.2014.01.035
10.1016/j.apsusc.2019.03.150
10.1016/j.actamat.2005.05.018
10.1016/j.jmapro.2016.12.018
10.1016/j.surfcoat.2016.09.011
10.1016/0026-0657(00)93577-X
10.1016/j.ssc.2010.11.022
10.1016/j.surfcoat.2006.03.048
10.1016/j.corsci.2016.09.014
10.1016/S0020-7683(02)00409-2
10.1016/0025-5416(87)90064-4
10.1016/j.jcrysgro.2019.125246
10.1016/j.scriptamat.2005.06.021
10.1016/S0022-3115(97)00175-X
10.1016/j.proeng.2011.12.539
10.1016/0036-9748(80)90267-7
10.1016/j.msea.2014.03.014
10.1016/s1620-7742(01)01409-x
10.1016/S1359-6462(02)00050-7
10.1016/S0167-577X(99)00096-8
10.1016/j.msea.2016.05.028
10.1016/j.msea.2008.08.017
10.1016/0040-6090(92)90779-b
10.1016/0026-0800(72)90082-1
10.1016/S0257-8972(02)00498-X
10.1016/j.apsusc.2019.04.047
10.1016/j.matdes.2016.06.085
10.1016/j.surfcoat.2004.10.108
10.1016/j.msea.2004.05.053
10.1016/1359-6462(96)00062-0
10.1016/j.matchar.2005.11.005
10.1016/j.msea.2019.03.040
10.1016/j.commatsci.2018.12.053
10.1016/j.corsci.2014.11.013
10.1016/j.msea.2016.06.079
10.1016/j.msea.2017.11.026
10.1016/j.jallcom.2011.09.097
10.1016/0001-6160(88)90335-5
10.1016/0026-0800(72)90029-8
10.1016/j.msea.2014.02.073
10.1016/0956-716X(95)00114-B
10.1016/j.surfcoat.2014.01.009
10.1016/j.jestch.2016.09.023
10.1016/j.ijfatigue.2013.08.009

10.1016/0956-7151(95)00046-X
10.1016/j.jmapro.2019.11.027
10.1016/j.matlet.2007.05.023
10.1016/0010-938x(81)90024-x
10.1016/j.jallcom.2007.04.268
10.1016/j.ijfatigue.2018.06.015
10.1016/j.msea.2007.07.005
10.1016/S1359-6462(01)01127-7
10.1016/j.matdes.2015.07.046
10.1016/S0966-9795(02)00092-4
10.1016/j.jclepro.2014.06.040
10.1016/j.msea.2015.10.006
10.1016/j.cam.2003.06.011
10.1016/j.msea.2017.10.003
10.1016/j.scriptamat.2014.06.032
10.1016/j.ijfatigue.2010.09.022
10.1016/S0921-5093(97)00399-7
10.1016/j.actamat.2016.10.009
10.1016/j.commatsci.2013.11.028
10.1016/S0927-0256(01)00160-4
10.1016/0921-5093(89)90817-4
10.1016/j.matlet.2015.10.075
10.1016/j.msea.2012.12.092
10.1016/j.optlastec.2005.10.009
10.1016/j.jallcom.2015.02.177
10.1016/j.jallcom.2008.06.033
10.1016/j.ijfatigue.2016.07.002
10.1016/j.ijmachtools.2007.12.007
10.1016/j.jmatprotec.2005.05.021
10.1016/j.jallcom.2018.11.381
10.1016/j.msea.2005.04.034
10.1016/j.msea.2017.11.004
10.1016/j.jallcom.2016.12.149
10.1016/j.actamat.2019.12.009
10.1016/s0036-9748(88)80161-3
10.1016/j.ijmachtools.2015.02.011
10.1016/S1359-6462(98)00427-8
10.1016/j.msea.2017.10.091
10.1016/j.msea.2017.11.024
10.1016/j.jallcom.2016.04.237
10.1016/j.ijfatigue.2011.09.014
10.1016/j.procir.2018.05.029
10.1016/j.ijsolstr.2016.03.012
10.1016/j.matdes.2018.02.048
10.1016/j.matchemphys.2017.07.060
10.1016/j.msea.2018.04.026
10.1016/j.apsusc.2013.07.167
10.1016/j.msea.2011.12.006
10.1016/j.msea.2016.09.088
10.1016/0013-7944(92)90183-f
10.1016/j.mechmat.2017.07.017
10.1016/j.intermet.2015.01.001
10.1016/j.ceramint.2017.03.035
10.1016/0036-9748(82)90388-x

10.1016/S0257-8972(02)00585-6
10.1016/j.matchar.2012.11.008
10.1016/j.jnucmat.2014.01.032
10.1016/j.actamat.2016.06.014
10.1016/0036-9748(80)90291-4
10.1016/S1359-6454(00)00197-X
10.1016/j.proeng.2011.11.133
10.1016/j.jallcom.2018.05.146
10.1016/S0261-3069(00)00060-1
10.1016/j.scriptamat.2016.07.033
10.1016/j.matpr.2018.06.232
10.1016/j.msea.2014.07.103
10.1016/j.actamat.2015.05.033
10.1016/j.ijpvp.2010.03.007
10.1016/s0036-9748(88)80131-5
10.1016/j.matchar.2019.109915
10.1016/j.matpr.2019.06.506
10.1016/j.ijfatigue.2018.12.021
10.1016/j.msea.2007.08.047
10.1016/j.matdes.2018.06.043
10.1016/S0168-583X(02)01335-6
10.1016/S0167-577X(02)01244-2
10.1016/S0026-0657(99)80654-7
10.1016/S0257-8972(97)00477-5
10.1016/0025-5416(84)90210-6
10.1016/j.surfcoat.2003.10.011
10.1016/j.ijmachtools.2016.12.001
10.1016/j.vacuum.2020.109247
10.1016/0036-9748(86)90103-1
10.1016/j.surfcoat.2003.09.044
10.1016/j.surfcoat.2005.09.004
10.1016/S0010-938X(01)00182-2
10.1016/j.msec.2016.01.036
10.1533/9780857090829.3.263
10.1016/0040-6090(96)80072-1
10.1016/S1359-6462(00)00662-X
10.1016/j.ijfatigue.2007.01.025
10.1016/j.msea.2011.02.051
10.1016/j.jallcom.2012.06.062
10.1016/S0921-5093(99)00189-6
10.1016/j.proeng.2015.06.238
10.1016/j.corsci.2012.06.027
10.1016/j.surfcoat.2012.11.045
10.1016/j.jallcom.2008.08.118
10.1016/j.ijfatigue.2007.01.052
10.1016/j.jallcom.2018.03.120
10.1016/j.jmatprotec.2019.116503
10.1016/S0921-5093(02)00559-2
10.1016/S0013-7944(98)00003-4
10.1016/0025-5416(83)90135-0
10.1016/j.wear.2007.01.089
10.1016/j.scriptamat.2018.11.008
10.1016/S0925-8388(01)01924-7
10.1016/j.jallcom.2018.09.301

10.1016/j.addma.2016.05.003
10.1016/j.msea.2005.06.016
10.1016/j.ceramint.2015.11.064
10.1016/S0257-8972(02)00738-7
10.1016/j.cma.2019.112725
10.1016/j.matchar.2015.09.021
10.1016/j.msea.2004.01.072
10.1016/j.ceramint.2016.12.119
10.1016/0956-716x(93)90398-c
10.1016/S0925-8388(02)00076-2
10.1016/S0921-5093(01)01742-7
10.1016/j.actamat.2006.11.045
10.1016/j.mechmat.2016.11.002
10.1016/S0022-3115(01)00613-4
10.1016/j.engfracmech.2010.08.005
10.1016/j.jallcom.2015.10.192
10.1016/j.actamat.2018.07.061
10.1016/j.jallcom.2017.06.305
10.1016/j.jmapro.2019.09.041
10.1016/j.corsci.2008.06.032
10.1016/S0921-5093(97)00662-X
10.1016/0001-6160(89)90048-5
10.1016/j.optlaseng.2012.06.006
10.1016/j.msea.2016.04.089
10.1016/j.msea.2019.06.008
10.1016/j.surfcoat.2009.01.029
10.1016/S1359-6454(03)00328-8
10.1016/j.msea.2017.09.049
10.1016/j.ijmecsci.2009.02.004
10.1016/j.commatsci.2019.04.023
10.1016/0040-6090(79)90511-x
10.1016/j.matchar.2019.01.028
10.1016/j.actamat.2019.06.008
10.1016/j.msea.2016.06.002
10.1016/j.commatsci.2014.11.049
10.1016/j.jmatprotec.2019.116557
10.1016/j.matdes.2019.107633
10.1016/j.msea.2014.07.035
10.1016/j.msea.2005.01.055
10.1016/j.jmps.2012.02.001
10.1016/j.msea.2010.05.019
10.1016/j.commatsci.2015.06.019
10.1016/j.procir.2018.08.221
10.1016/j.matdes.2007.04.008
10.1016/j.matdes.2016.12.062
10.1016/j.matdes.2012.09.052
10.1016/j.msea.2007.02.047
10.1016/0273-1177(95)00158-B
10.1016/j.jmapro.2019.03.014
10.1016/j.msea.2008.01.032
10.1016/j.apsusc.2010.12.050
10.1016/j.surfcoat.2005.11.111
10.1016/j.jclepro.2016.09.212
10.1016/j.jallcom.2019.152055

10.1016/0956-716X(95)00004-F
10.1016/j.jmapro.2019.04.016
10.1016/j.surfcoat.2018.10.022
10.1016/j.vacuum.2019.108875
10.1016/j.ceramint.2019.11.247
10.1016/j.msea.2006.02.400
10.1016/0025-5416(81)90004-5
10.1016/j.jallcom.2017.12.278
10.1016/j.proeng.2011.12.541
10.1016/j.matpr.2017.08.069
10.1016/j.commatsci.2016.05.008
10.1016/j.msea.2015.04.018
10.1016/0036-9748(83)90229-6
10.1016/S0010-938X(02)00183-X
10.1016/j.surfcoat.2014.10.004
10.1016/j.corsci.2018.01.013
10.1016/S0142-1123(99)00041-9
10.1016/j.jmatprotec.2016.07.013
10.1016/S1005-0302(11)60063-7
10.1016/j.ijmecsci.2017.01.018
10.1016/S1359-6462(02)00379-2
10.1016/S0924-0136(97)00429-9
10.1016/S0921-5093(01)01491-5
10.1016/j.jallcom.2019.07.121
10.1016/j.jeurceramsoc.2007.09.053
10.1016/j.actamat.2008.04.044
10.1016/j.msea.2006.12.064
10.1016/j.msea.2005.05.024
10.1016/1359-6454(96)00011-0
10.1016/j.matdes.2016.05.059
10.1016/j.msea.2007.09.055
10.1016/j.jmps.2012.04.009
10.1016/j.apsusc.2014.10.106
10.1016/S1003-6326(11)61436-2
10.1016/j.commatsci.2019.109408
10.1016/S0257-8972(96)02995-7
10.1016/S1359-6462(98)00255-3
10.1016/j.ijfatigue.2005.06.029
10.1016/j.matdes.2005.10.001
10.1016/1044-5803(90)90021-b
10.1016/S0142-1123(98)00022-X
10.1016/j.vacuum.2016.11.003
10.1016/j.matchar.2005.11.015
10.1016/j.msea.2006.11.039
10.1016/j.surfcoat.2017.07.049
10.1016/j.scriptamat.2012.06.013
10.1016/j.ijleo.2019.163199
10.1016/j.matlet.2020.127481
10.1016/j.jallcom.2016.01.012
10.1016/j.procir.2018.08.219
10.1016/j.scriptamat.2006.01.008
10.1016/j.matchar.2018.03.005
10.1016/S0921-5093(98)00639-X
10.1016/0001-6160(80)90039-5

10.1016/j.msea.2004.10.042
10.1016/0010-938x(90)90076-h
10.1016/j.msea.2014.12.030
10.1016/S0925-8388(03)00748-5
10.1016/S0921-5093(03)00627-0
10.1016/j.matchemphys.2014.05.018
10.1016/j.jallcom.2014.11.048
10.1016/0956-7151(95)00124-E
10.1016/0921-5093(95)09804-6
10.1016/j.ijfatigue.2018.11.004
10.1016/j.commatsci.2017.08.002
10.1016/0026-0800(74)90006-8
10.1016/j.wear.2015.05.009
10.1016/j.prostr.2019.07.032
10.1016/j.msea.2014.11.041
10.1016/j.matdes.2018.08.054
10.1016/j.jmatprotec.2016.11.040
10.1016/j.actamat.2012.09.040
10.1016/j.ijfatigue.2016.11.025
10.1016/j.msea.2008.03.043
10.1016/j.measurement.2015.03.006
10.1016/j.optlaseng.2007.08.011
10.1016/j.jmatprotec.2009.07.022
10.1016/j.actamat.2004.08.007
10.1016/j.actamat.2018.07.025
10.1016/j.jmst.2018.09.013
10.1016/j.corsci.2017.01.004
10.1016/j.surfcoat.2018.03.089
10.1016/j.jallcom.2012.09.015
10.1016/j.jallcom.2019.04.194
10.1016/j.matdes.2015.07.085
10.1016/S1359-6454(01)00060-X
10.1016/1044-5803(94)00079-Z
10.1016/j.actamat.2020.02.028
10.1016/j.msea.2005.01.057
10.1016/j.proeng.2014.03.078
10.1016/S1359-6462(98)00154-7
10.1016/j.msea.2010.07.040
10.1016/j.proeng.2012.03.033
10.1016/j.actamat.2016.02.038
10.1016/j.ijfatigue.2018.03.028
10.1016/S0924-0136(01)00969-4
10.1016/j.jmst.2015.11.019
10.1016/j.surfcoat.2015.11.018
10.1016/S0921-5093(00)01005-4
10.1016/j.matdes.2019.107603
10.1016/0169-4332(94)90335-2
10.1016/j.ijfatigue.2019.105255
10.1016/j.intermet.2007.08.009
10.1016/j.optlastec.2017.03.011
10.1016/j.matdes.2007.03.011
10.1016/j.ijfatigue.2017.08.021
10.1016/S0921-5093(01)01709-9
10.1016/j.jmatprotec.2008.10.012

10.1016/j.corsci.2011.12.031
10.1016/j.matdes.2015.01.011
10.1016/j.actamat.2019.08.049
10.1016/0040-6090(80)90503-9
10.1016/S0921-5093(97)00264-5
10.1016/s0010-938x(78)80029-8
10.1016/j.msea.2006.07.033
10.1016/j.msea.2012.04.067
10.1016/j.matchar.2012.04.010
10.1016/S0043-1648(03)00134-0
10.1016/j.msea.2018.07.022
10.1016/j.surfcoat.2004.08.001
10.1016/j.engfracmech.2012.04.021
10.1016/j.jmst.2015.10.005
10.1016/j.jmatprotec.2016.04.011
10.1016/j.actamat.2017.04.029
10.1016/j.jallcom.2010.12.120
10.1016/j.optlaseng.2018.01.011
10.1016/j.matpr.2014.09.002
10.1016/j.surfcoat.2007.03.028
10.1016/j.scriptamat.2010.09.001
10.1016/j.jallcom.2017.11.316
10.1016/S1359-6462(96)00450-2
10.1016/j.cja.2016.01.005
10.1016/j.procir.2012.07.064
10.1016/S0921-5093(96)10504-9
10.1016/S1359-6454(99)00092-0
10.1016/j.msea.2017.03.105
10.1016/j.corsci.2015.05.066
10.1016/j.msea.2013.05.015
10.1016/j.msea.2017.06.044
10.1016/j.matchar.2018.03.035
10.1016/j.matchar.2015.11.021
10.1016/S0924-0136(01)00784-1
10.1016/j.cclet.2018.01.001
10.1016/j.compstruct.2017.09.004
10.1016/j.ceramint.2016.05.047
10.1016/j.jallcom.2017.10.105
10.1016/S0142-1123(98)00071-1
10.1016/j.surfcoat.2005.07.098
10.1016/S0257-8972(00)01165-8
10.1016/j.msea.2006.08.028
10.1016/j.scriptamat.2014.08.021
10.1016/j.surfcoat.2019.04.018
10.1016/j.precisioneng.2017.01.008
10.1016/0956-716x(91)90074-b
10.1016/j.msea.2016.12.068
10.1016/j.matdes.2013.10.088
10.1016/S0257-8972(01)01482-7
10.1016/1044-5803(92)90080-2
10.1016/0025-5416(83)90093-9
10.1016/j.intermet.2012.07.034
10.1016/j.msea.2015.11.053
10.1016/j.intermet.2004.07.048

10.1016/j.vacuum.2017.11.021
10.1016/j.matchar.2016.06.028
10.1016/0040-6090(77)90237-1
10.1016/j.matdes.2013.10.071
10.1016/S1003-6326(15)63587-7
10.1016/j.scriptamat.2004.03.035
10.1016/j.vacuum.2017.06.025
10.1016/j.cocom.2017.11.001
10.1016/j.actamat.2014.11.037
10.1016/j.msea.2013.10.014
10.1016/j.addma.2016.03.006
10.1016/j.msea.2016.05.031
10.1016/j.jallcom.2007.08.065
10.1016/j.matdes.2017.03.057
10.1016/S0921-5093(01)00933-9
10.1016/S0924-0136(03)00449-7
10.1016/j.commatsci.2010.10.006
10.1016/j.matdes.2018.10.042
10.1016/j.msea.2013.03.021
10.1016/S0921-5093(00)00644-4
10.1016/j.corsci.2007.09.004
10.1016/j.corsci.2003.10.025
10.1016/0966-9795(96)00023-4
10.1016/j.actamat.2014.08.033
10.1016/0956-716x(93)90380-b
10.1016/0921-5093(94)90364-6
10.1016/j.msea.2007.01.090
10.1016/j.vacuum.2003.11.001
10.1016/j.matdes.2017.03.014
10.1016/j.jallcom.2007.03.019
10.1016/j.vacuum.2016.04.032
10.1016/j.matchar.2009.03.004
10.1016/S0022-3115(00)00302-0
10.1016/j.npe.2019.03.003
10.1016/j.jmapro.2016.11.010
10.1016/j.matpr.2018.02.095
10.1016/S1003-6326(08)60120-X
10.1016/j.intermet.2004.02.021
10.1016/j.matdes.2013.12.025
10.1016/j.surfcoat.2013.08.061
10.1016/j.actamat.2019.12.014
10.1016/j.ijfatigue.2007.01.044
10.1016/j.actamat.2010.10.013
10.1016/j.msea.2019.138810
10.1016/j.msea.2017.04.016
10.1016/j.matdes.2017.06.018
10.1016/j.pnsc.2013.09.005
10.1016/j.engfracmech.2010.03.001
10.1016/j.jallcom.2017.10.120
10.1016/j.corsci.2014.06.050
10.1016/j.actamat.2014.12.023
10.1016/j.jcrysgro.2013.05.016
10.1016/j.matchar.2016.05.026
10.1016/j.msea.2011.03.069

10.1016/j.ijsolstr.2018.09.011
10.1016/j.corsci.2015.09.003
10.1016/j.ijfatigue.2005.06.032
10.1016/j.promfg.2017.07.080
10.1016/S0924-0136(03)00792-1
10.1016/j.addma.2019.100932
10.1016/j.ultramic.2020.112944
10.1016/j.engfailanal.2010.12.025
10.1016/j.corsci.2017.09.020
10.1016/j.addma.2019.100936
10.1016/j.msea.2014.03.136
10.1016/j.intermet.2010.08.019
10.1016/j.msea.2019.03.054
10.1016/j.actamat.2011.11.037
10.1016/0022-3115(90)90351-m
10.1016/j.corsci.2014.08.022
10.1016/j.actamat.2019.04.038
10.1016/j.commatsci.2017.12.037
10.1016/j.jmst.2016.01.020
10.1016/0036-9748(87)90287-0
10.1016/j.actamat.2016.06.043
10.1016/j.jallcom.2016.07.204
10.1016/j.matdes.2016.09.072
10.1016/0956-7151(91)90222-m
10.1016/j.ijimpeng.2003.11.004
10.1016/j.engfracmech.2017.04.036
10.1016/0001-6160(88)90147-2
10.1016/0001-6160(82)90140-7
10.1016/j.optlastec.2016.02.013
10.1016/j.ijpvp.2005.01.007
10.1016/S0257-8972(03)00748-5
10.1016/j.msea.2014.08.015
10.1016/j.msea.2017.03.021
10.1016/j.intermet.2017.06.011
10.1016/j.precisioneng.2015.09.012
10.1016/j.jallcom.2015.06.069
10.1016/j.matchar.2012.02.014
10.1016/j.ijimpeng.2009.01.007
10.1016/S1003-6326(16)64131-6
10.1016/j.intermet.2006.10.030
10.1016/j.cirp.2017.04.110
10.1016/S0921-5093(01)01688-4
10.1016/j.jmatprotec.2013.06.001
10.1016/0025-5416(74)90048-2
10.1016/j.jmatprotec.2004.01.006
10.1016/j.matchar.2012.11.006
10.1016/j.jallcom.2019.07.045
10.1016/j.msea.2015.02.010
10.1016/j.matchar.2004.12.014
10.1016/j.msea.2004.07.016
10.1016/j.surfcoat.2011.11.029
10.1016/j.msea.2010.05.051
10.1016/0001-6160(88)90044-2
10.1016/j.jallcom.2019.03.037

10.1016/j.jallcom.2016.05.280
10.1016/j.ijplas.2019.03.012
10.1016/j.actamat.2004.10.036
10.1016/j.matdes.2015.11.036
10.1016/j.ijmecsci.2017.04.023
10.1016/j.jnucmat.2018.12.035
10.1016/j.matlet.2007.11.002
10.1016/0013-7944(83)90137-6
10.1016/0036-9748(85)90124-3
10.1016/j.jallcom.2017.02.132
10.1016/j.actamat.2009.11.004
10.1016/j.jnucmat.2010.01.022
10.1016/j.msea.2010.02.045
10.1016/j.matchar.2016.03.019
10.1016/j.euromechsol.2011.10.001
10.1016/1359-6454(95)00374-6
10.1016/j.actamat.2015.08.041
10.1016/j.jallcom.2015.11.002
10.1016/j.msea.2017.05.072
10.1016/j.engfailanal.2006.11.041
10.1016/j.jallcom.2014.01.243
10.1016/j.msea.2006.05.088
10.1016/j.actamat.2019.07.041
10.1016/j.apsusc.2008.04.042
10.1016/S0142-1123(00)00048-7
10.1016/j.ijfatigue.2013.09.017
10.1016/j.msea.2010.04.090
10.1016/j.jmst.2017.01.027
10.1016/j.matdes.2016.06.067
10.1016/j.sab.2007.10.048
10.1016/j.corsci.2013.06.031
10.1016/j.ijplas.2011.07.003
10.1016/j.intermet.2005.08.007
10.1016/j.jmatprotec.2012.03.014
10.1016/j.physb.2017.12.014
10.1016/j.surfcoat.2011.06.008
10.1016/S0921-5093(02)00809-2
10.1016/j.matlet.2007.11.068
10.1016/j.jmatprotec.2011.09.022
10.1016/j.matchar.2014.04.009
10.1016/j.procir.2014.04.004
10.1016/j.matdes.2014.08.045
10.1016/j.msea.2015.01.060
10.1016/j.engfracmech.2006.02.002
10.1016/j.commatsci.2017.10.042
10.1016/j.msea.2008.11.066
10.1016/j.actamat.2015.11.036
10.1016/j.measurement.2019.05.032
10.1016/j.commatsci.2015.12.045
10.1016/S1005-0302(12)60016-4
10.1016/j.jallcom.2016.09.303
10.1016/j.corsci.2011.09.009
10.1016/S0921-4526(97)00519-X
10.1016/j.phpro.2015.11.054

10.1016/0025-5416(86)90173-4
10.1016/j.msea.2011.11.015
10.1016/j.jmatprotec.2009.05.031
10.1016/j.ijfatigue.2015.02.007
10.1016/j.ijpvp.2018.06.004
10.1016/S1359-6462(99)00224-9
10.1016/j.surfcoat.2013.07.060
10.1016/j.actamat.2018.10.014
10.1016/j.matdes.2013.02.022
10.1016/j.jnucmat.2017.01.013
10.1016/j.jallcom.2018.06.146
10.1016/S0301-679X(02)00142-1
10.1016/S0257-8972(02)00602-3
10.1016/S1003-6326(13)62685-0
10.1016/j.msea.2007.03.119
10.1016/S0921-5093(02)00074-6
10.1016/j.ijfatigue.2008.03.001
10.1016/j.ijhydene.2018.02.088
10.1016/j.procir.2018.05.044
10.1016/j.actamat.2015.02.005
10.1016/j.msea.2017.12.038
10.1063/2.1402104
10.1016/S0040-6031(02)00112-0
10.1016/j.matchar.2013.12.009
10.1016/j.matlet.2003.10.053
10.1016/j.ijplas.2010.09.009
10.1016/S1003-6326(11)60812-1
10.1016/0042-207x(90)90136-m
10.1016/j.corsci.2018.11.004
10.1016/j.ijmecsci.2016.05.021
10.1016/j.actamat.2013.07.039
10.1016/j.msea.2018.02.086
10.1016/j.triboint.2008.12.003
10.1016/j.surfcoat.2018.01.001
10.1016/j.matchemphys.2009.09.024
10.1016/j.matlet.2019.01.066
10.1016/j.ast.2015.12.017
10.1016/1359-6462(95)00666-4
10.1016/j.scriptamat.2018.03.044
10.1016/j.jeurceramsoc.2015.10.041
10.1016/j.jmapro.2020.03.045
10.1016/j.msea.2005.09.091
10.1016/j.cocom.2017.08.007
10.1016/j.matpr.2018.02.238
10.1016/j.msea.2012.03.006
10.1016/j.vacuum.2018.10.011
10.1016/j.msea.2016.11.054
10.1016/j.msea.2019.03.132
10.1016/j.intermet.2018.08.008
10.1016/S0921-4526(98)80042-2
10.1016/j.msea.2016.08.113
10.1016/j.vacuum.2018.05.019
10.1016/j.msea.2010.08.003
10.1016/j.ijplas.2013.08.016

10.1016/S0257-8972(01)01371-8
10.1016/j.ijrmhm.2020.105202
10.1016/S0921-5093(97)00733-8
10.1016/j.msea.2018.08.037
10.1016/j.surfcoat.2011.06.011
10.1016/S0022-3115(01)00517-7
10.1016/j.msea.2015.11.018
10.1016/j.corsci.2013.07.012
10.1016/j.surfcoat.2018.02.083
10.1016/0956-7151(93)90220-m
10.1016/j.msea.2011.04.049
10.1016/0040-6090(94)90087-6
10.1016/j.jmst.2013.09.011
10.1016/j.msea.2004.01.045
10.1016/0013-7944(86)90222-5
10.1016/j.scriptamat.2005.12.019
10.1016/j.wear.2006.10.009
10.1016/j.optlaseng.2018.03.019
10.1016/j.commatsci.2018.02.046
10.1016/j.jmatprotec.2008.09.024
10.1016/0956-716x(93)90073-2
10.1016/j.jmst.2018.07.001
10.1016/j.jallcom.2006.09.049
10.1016/S1003-6326(07)60140-X
10.1016/S1003-6326(16)64094-3
10.1016/j.jallcom.2018.09.267
10.1016/j.wear.2006.08.028
10.1016/j.msea.2016.06.018
10.1016/0039-6028(91)90434-t
10.1016/j.apsusc.2017.09.215
10.1016/j.scriptamat.2016.04.042
10.1016/j.surfcoat.2018.11.066
10.1016/j.triboint.2019.106084
10.1016/j.corsci.2011.04.020
10.1016/j.compind.2005.05.012
10.1016/j.ijplas.2007.02.005
10.1016/S0257-8972(01)01454-2
10.1016/j.msea.2011.03.035
10.1016/j.matchar.2017.04.008
10.1016/S1359-6454(02)00512-8
10.1016/j.msea.2017.07.087
10.1016/j.msea.2009.11.067
10.1016/j.jmatprotec.2016.05.003
10.1016/j.jallcom.2013.09.171
10.1016/j.scriptamat.2017.12.008
10.1016/j.msea.2013.10.055
10.1016/j.matpr.2018.06.135
10.1016/j.actamat.2014.01.056
10.1016/j.scriptamat.2010.04.017
10.1016/j.commatsci.2018.08.064
10.1016/j.ijsolstr.2020.01.004
10.1016/j.matdes.2019.108105
10.1016/j.actamat.2012.12.004
10.1016/j.matdes.2009.12.020

10.1016/S0925-8388(00)00789-1
10.1016/S0924-0136(00)00833-5
10.1016/j.ijfatigue.2018.01.005
10.1016/j.ijplas.2019.10.003
10.1016/S1359-6454(02)00523-2
10.1016/j.jallcom.2018.04.301
10.1016/j.ceramint.2017.12.001
10.1016/j.msea.2007.06.076
10.1016/j.ijfatigue.2014.05.011
10.1016/j.surfcoat.2005.11.073
10.1016/S1359-6454(96)00050-X
10.1016/j.matchar.2018.01.048
10.1016/j.actamat.2015.06.022
10.1016/j.matdes.2015.09.016
10.1016/j.corsci.2016.04.041
10.1016/j.jeurceramsoc.2014.07.029
10.1016/S0022-0248(99)00783-6
10.1016/j.jallcom.2018.05.067
10.1016/S0921-5093(00)00829-7
10.1016/j.pmatsci.2017.06.004
10.1016/S0257-8972(01)01304-4
10.1016/j.jallcom.2019.151662
10.1016/j.jeurceramsoc.2013.05.018
10.1016/0036-9748(82)90414-8
10.1016/0022-3115(92)90337-k
10.1016/1044-5803(92)90028-g
10.1016/j.surfcoat.2011.07.077
10.1016/S1359-6454(97)00309-1
10.1016/0025-5416(83)90175-1
10.1016/j.matdes.2017.03.004
10.1016/j.msea.2006.08.132
10.1016/0001-6160(88)90031-4
10.1016/0364-5916(80)90019-x
10.1016/S0921-5093(00)01530-6
10.1016/j.jallcom.2018.06.269
10.1016/j.actaastro.2007.12.059
10.1016/0257-8972(92)90140-6
10.1016/j.ijfatigue.2006.12.018
10.1016/S1000-9361(11)60159-3
10.1016/j.actamat.2019.07.024
10.1016/S0921-5093(03)00156-4
10.1016/j.actamat.2014.11.009
10.1016/j.ceramint.2017.04.109
10.1016/S1003-6326(08)60421-5
10.1016/j.pnsc.2012.10.001
10.1016/j.matpr.2019.11.327
10.1016/S0921-5093(97)00517-0
10.1016/0956-716x(90)90580-a
10.1016/j.matdes.2016.02.055
10.1016/j.jssc.2016.09.013
10.1016/j.msea.2016.03.133
10.1016/S0022-0248(01)01380-X
10.1016/S0263-4368(00)00008-1
10.1016/0921-5093(89)90388-2

10.1016/j.jallcom.2018.10.128
10.1016/j.jallcom.2015.03.157
10.1016/j.jallcom.2015.01.227
10.1016/j.jallcom.2020.154352
10.1016/j.ijfatigue.2017.12.003
10.1016/S0142-1123(98)91119-7
10.1016/0039-6028(91)90418-r
10.1016/0040-6090(80)90502-7
10.1016/0956-7151(90)90250-k
10.1016/j.jallcom.2016.11.236
10.1016/S1003-6326(08)60093-X
10.1016/j.jallcom.2017.09.164
10.1016/j.scriptamat.2017.05.027
10.1016/j.actamat.2011.07.069
10.1016/0036-9748(70)90161-4
10.1016/j.apsusc.2012.12.065
10.1016/j.jallcom.2013.07.201
10.1016/j.mprp.2020.02.002
10.1016/j.ijfatigue.2020.105572
10.1016/j.ijfatigue.2017.10.012
10.1016/j.corsci.2018.02.006
10.1016/j.actamat.2010.05.032
10.1016/j.surfcoat.2016.11.041
10.1016/j.jallcom.2017.09.067
10.1016/j.actamat.2006.02.015
10.1016/j.matdes.2013.08.093
10.1016/j.jallcom.2017.11.165
10.1016/j.jallcom.2017.04.279
10.1016/j.actamat.2017.02.022
10.1016/j.compscitech.2006.03.010
10.1016/j.jmatprotec.2006.09.018
10.1016/j.ijheatmasstransfer.2014.09.044
10.1016/S1003-6326(11)61358-7
10.1016/j.apsusc.2019.02.247
10.1016/j.matchar.2018.05.032
10.1016/j.actamat.2012.11.024
10.1016/0001-6160(83)90217-1
10.1016/j.msea.2006.11.086
10.1016/j.surfcoat.2005.12.027
10.1016/0142-1123(94)90458-8
10.1016/j.msea.2003.09.051
10.1016/j.msea.2010.09.047
10.1016/j.actamat.2020.03.056
10.1016/0301-679x(81)90041-4
10.1016/j.ijplas.2008.01.001
10.1016/j.ijfatigue.2013.06.012
10.1016/j.matpr.2018.06.247
10.1016/j.engfracmech.2018.06.026
10.1016/j.triboint.2015.11.031
10.1016/j.addma.2018.09.012
10.1016/j.scriptamat.2005.03.022
10.1016/j.cja.2017.08.014
10.1016/j.jallcom.2017.04.116
10.1016/S0261-3069(02)00002-X

10.1016/j.ijfatigue.2019.105195
10.1016/S1003-6326(06)60115-5
10.1016/j.vacuum.2015.03.022
10.1016/S1359-6462(97)00062-6
10.1016/j.surfcoat.2008.08.072
10.1016/j.rinp.2017.06.020
10.1016/j.intermet.2015.06.024
10.1016/j.corsci.2011.01.004
10.1016/0921-5093(93)90332-9
10.1016/j.cja.2015.09.006
10.1016/j.actamat.2019.03.001
10.1016/j.msea.2006.09.015
10.1016/j.surfcoat.2014.09.015
10.1016/j.matchemphys.2012.06.055
10.1016/j.msea.2006.06.124
10.1016/S0143-7496(99)00058-5
10.1016/j.ceramint.2009.12.003
10.1016/j.jallcom.2020.154438
10.1016/j.surfcoat.2017.05.038
10.1016/j.corsci.2010.09.068
10.1016/j.jallcom.2007.01.175
10.1016/j.msea.2011.02.081
10.1016/0956-7151(91)90095-i
10.1016/j.corsci.2016.09.006
10.1016/S0167-577X(03)00359-8
10.1016/j.corsci.2017.07.014
10.1016/j.intermet.2006.10.041
10.1016/j.jmrt.2019.12.084
10.1016/0022-3115(87)90486-7
10.1016/j.jmatprotec.2018.03.023
10.1016/j.msea.2015.04.093
10.1016/j.ijfatigue.2016.08.019
10.1016/j.jallcom.2018.01.042
10.1016/j.matdes.2014.04.084
10.1016/j.jallcom.2009.05.035
10.1016/S0921-5093(97)00069-5
10.1016/0304-3991(95)00148-4
10.1016/j.proeng.2017.01.141
10.1016/j.jmst.2013.09.024
10.1016/0029-5493(87)90313-x
10.1016/j.actamat.2005.04.025
10.1016/j.jallcom.2018.06.275
10.1016/j.msea.2007.04.108
10.1016/j.jmapro.2018.06.011
10.1016/j.mspro.2014.07.590
10.1016/S0925-8388(01)01676-0
10.1016/j.jmatprotec.2018.04.011
10.1016/S1003-6326(18)64674-6
10.1016/j.msea.2010.02.062
10.1016/S0261-3069(02)00005-5
10.1016/S1359-6454(98)00407-8
10.1016/j.jnucmat.2019.07.001
10.1016/S0956-716X(95)00550-F
10.1016/j.corsci.2013.10.013

10.1016/j.ijmecsci.2004.08.003
10.1016/j.triboint.2006.02.008
10.1016/j.jnucmat.2015.10.023
10.1016/j.vacuum.2006.03.024
10.1016/j.jnucmat.2013.08.012
10.1016/j.msea.2016.04.033
10.1016/j.matdes.2013.07.091
10.1016/S0921-5093(02)00037-0
10.1016/j.jallcom.2008.08.138
10.1016/j.msea.2017.06.045
10.1016/j.ijrmhm.2017.11.033
10.1016/j.optlastec.2019.105761
10.1016/S1566-1369(02)80062-0
10.1016/j.jmapro.2019.05.008
10.1016/j.surfcoat.2019.03.057
10.1016/j.msea.2007.02.020
10.1016/j.wear.2014.03.007
10.1016/j.actamat.2018.11.051
10.1016/j.calphad.2017.09.007
10.1016/j.msea.2019.05.075
10.1016/j.measurement.2017.07.016
10.1016/j.procir.2018.05.043
10.1016/j.jmatprotec.2004.04.275
10.1016/j.corsci.2019.07.006
10.1016/j.corsci.2016.03.017
10.1016/j.ijfatigue.2007.06.010
10.1016/0921-5093(91)90265-o
10.1016/j.engfracmech.2018.06.028
10.1016/j.acme.2018.05.007
10.1016/S1359-6454(98)00142-6
10.1016/j.msea.2012.09.002
10.1016/j.surfcoat.2019.01.116
10.1016/j.engfracmech.2019.106842
10.1016/S1002-0071(12)60078-1
10.1016/0956-716x(94)90129-5
10.1016/S1000-9361(08)60123-5
10.1016/j.matchar.2018.02.020
10.1016/j.msea.2018.06.032
10.1016/j.scriptamat.2013.09.005
10.1016/j.jmatprotec.2004.04.138
10.1016/j.msea.2017.03.098
10.1016/S0010-4655(02)00237-0
10.1016/j.acme.2017.05.004
10.1016/j.msea.2015.05.051
10.1016/S0924-0136(99)00430-6
10.1016/j.actamat.2013.01.001
10.1016/j.msea.2017.03.045
10.1016/j.actamat.2019.06.054
10.1016/0921-5093(94)91069-3
10.1016/j.ceramint.2013.07.023
10.1016/j.actamat.2004.03.047
10.1016/j.matdes.2018.02.020
10.1016/j.matdes.2012.10.001
10.1016/S0966-9795(99)00060-6

10.1016/j.jallcom.2015.07.104
10.1016/j.jmatprotec.2007.08.080
10.1016/S0921-5093(97)00699-0
10.1016/j.corsci.2004.09.011
10.1016/0040-6090(89)90007-2
10.1016/j.commatsci.2009.03.030
10.1016/S0010-938X(00)00132-3
10.1016/j.surfcoat.2003.10.051
10.1016/j.scriptamat.2020.01.028
10.1016/j.corsci.2013.01.041
10.1016/j.msea.2014.03.134
10.1016/j.jallcom.2007.02.096
10.1016/j.matchar.2018.03.013
10.1016/j.jeurceramsoc.2016.10.006
10.1016/j.msea.2016.12.028
10.1016/0036-9748(76)90298-2
10.1016/j.scriptamat.2013.03.026
10.1016/j.jallcom.2014.09.103
10.1016/0022-3115(93)90271-y
10.1016/j.msea.2018.02.062
10.1016/0956-716x(91)90450-f
10.1016/j.prostr.2016.06.378
10.1016/j.ijrmhm.2016.04.006
10.1016/j.engfailanal.2009.03.017
10.1016/j.msea.2008.02.008
10.1016/j.jallcom.2015.10.238
10.1016/j.matdes.2016.05.033
10.1007/s10338-008-0833-2
10.1016/0039-6028(91)90419-s
10.1016/j.surfcoat.2016.05.002
10.1016/S0257-8972(99)00008-0
10.1016/S1359-6462(97)00548-4
10.1016/j.msea.2005.10.025
10.1016/S0921-5093(03)00107-2
10.1016/S1003-6326(11)61141-2
10.1016/j.surfcoat.2006.07.244
10.1016/j.msea.2018.04.109
10.1016/j.matchar.2015.04.007
10.1016/j.msea.2004.01.030
10.1016/j.promfg.2019.06.228
10.1016/j.jallcom.2012.10.129
10.1016/j.ijfatigue.2018.05.021
10.1016/0921-5093(93)90379-s
10.1016/0921-5093(90)90234-t
10.1016/S0921-5093(03)00464-7
10.1016/j.msea.2014.01.015
10.1016/j.jmst.2019.11.015
10.1016/j.corsci.2018.08.029
10.1016/j.matchar.2011.04.003
10.1016/j.prostr.2017.11.059
10.1016/j.apsusc.2011.06.120
10.1016/j.corsci.2019.01.012
10.1016/1359-6462(95)00568-4
10.1016/S0142-1123(97)00015-7

10.1016/0257-8972(92)90199-k
10.1016/j.msea.2004.09.037
10.1016/j.infrared.2016.07.021
10.1016/0921-5093(89)90715-6
10.1016/j.jmps.2010.12.014
10.1016/j.ijsolstr.2006.11.037
10.1016/j.scriptamat.2015.10.039
10.1016/j.surfcoat.2020.125526
10.1016/j.msea.2006.07.153
10.1016/j.corsci.2013.07.008
10.1016/S0921-5093(98)01152-6
10.1016/0036-9748(89)90363-3
10.1016/0921-5093(91)90724-2
10.1016/j.jallcom.2008.01.111
10.1016/0921-5093(91)90768-i
10.1016/j.scriptamat.2004.04.034
10.1016/j.msea.2007.04.091
10.1016/j.powtec.2018.06.002
10.1016/0036-9748(89)90454-7
10.1016/j.jallcom.2019.06.196
10.1016/j.proeng.2015.12.522
10.1016/j.jeurceramsoc.2017.02.051
10.1016/j.msea.2005.09.030
10.1016/S1006-706X(14)60198-9
10.1016/j.actamat.2017.06.058
10.1016/j.matdes.2017.01.034
10.1016/j.scriptamat.2007.06.031
10.1016/S0261-3069(01)00074-7
10.1016/j.ceramint.2016.06.100
10.1016/B978-0-08-099427-7.00011-6
10.1016/j.matdes.2015.08.075
10.1016/j.msea.2015.10.099
10.1016/j.talanta.2004.09.001
10.1016/0956-716x(93)90165-o
10.1016/S0921-5093(03)00153-9
10.1016/S0921-5093(01)01264-3
10.1016/j.actamat.2007.01.036
10.1016/j.engfracmech.2016.11.016
10.1016/j.matchar.2018.07.024
10.1016/j.engfailanal.2011.03.011
10.1016/S0257-8972(00)00693-9
10.1016/0921-5093(89)90814-9
10.1016/j.surfcoat.2011.06.036
10.1016/S1359-6462(97)00373-4
10.1016/j.vacuum.2019.108938
10.1016/j.pnsc.2014.03.006
10.1016/j.jallcom.2008.07.127
10.1016/j.jnucmat.2019.02.046
10.1016/S0921-5093(96)10442-1
10.1016/1359-6454(95)00147-9
10.1016/j.corsci.2007.10.007
10.1016/j.ijfatigue.2019.03.029
10.1016/j.jallcom.2016.10.078
10.1016/j.jmst.2018.09.035

10.1016/j.jmapro.2020.03.002
10.1016/j.actamat.2008.10.029
10.1016/j.intermet.2006.03.009
10.1016/j.msea.2008.12.006
10.1016/j.ijfatigue.2016.09.015
10.1016/j.physb.2018.07.004
10.1016/j.apsusc.2017.03.116
10.1016/S0142-1123(97)87159-9
10.1016/S0010-938X(97)00167-4
10.1016/j.msea.2019.02.077
10.1016/S0263-4368(02)00016-1
10.1016/S0921-5093(01)01666-5
10.1016/j.jallcom.2015.04.184
10.1016/j.msea.2014.09.095
10.1016/0956-716X(95)00420-Z
10.1016/j.proeng.2011.12.546
10.1016/j.scriptamat.2006.07.024
10.1016/j.jallcom.2018.04.067
10.1016/j.actamat.2016.07.008
10.1016/j.intermet.2019.106558
10.1016/j.apsusc.2013.01.165
10.1016/j.matlet.2007.05.036
10.1016/j.jmatprotec.2018.11.020
10.1016/j.actamat.2010.04.015
10.1016/j.proeng.2010.03.074
10.1016/S0029-5493(96)01361-1
10.1016/1044-5803(94)00045-M
10.1016/j.jeurceramsoc.2008.06.035
10.1016/j.apsusc.2004.03.141
10.1016/S0022-3115(00)00363-9
10.1016/j.matdes.2020.108501
10.1016/j.jallcom.2019.153217
10.1016/0025-5416(76)90077-x
10.1016/j.jallcom.2010.04.183
10.1016/j.surfcoat.2006.07.241
10.1016/0169-4332(93)90330-e
10.1016/0040-6090(84)90013-0
10.1016/j.msea.2007.08.046
10.1016/j.surfcoat.2009.09.072
10.1016/j.corsci.2013.06.013
10.1016/j.corsci.2018.05.001
10.1016/j.msea.2015.09.004
10.1016/j.vacuum.2018.07.041
10.1016/j.jallcom.2019.153424
10.1016/S0022-3115(02)00804-8
10.1016/s1468-6996(01)00033-x
10.1016/j.jallcom.2008.04.087
10.1016/j.mex.2018.04.006
10.1016/j.jallcom.2019.153026
10.1016/j.ijfatigue.2004.01.002
10.1016/0042-207x(84)90171-4
10.1016/j.intermet.2005.11.030
10.1016/j.promfg.2018.06.089
10.1016/j.engfracmech.2020.106899

10.1016/S0921-5093(01)01234-5
10.1016/j.matchar.2018.06.015
10.1016/j.corsci.2017.07.021
10.1016/j.jallcom.2011.07.050
10.1016/j.promfg.2015.09.024
10.1016/j.matchar.2013.04.018
10.1016/j.wear.2007.03.011
10.1016/j.matpr.2018.02.132
10.1016/j.jmatprotec.2011.04.011
10.1016/S1005-0302(10)60141-7
10.1533/9781845693954.2.77
10.1016/j.msea.2007.10.088
10.1016/j.mechmat.2016.03.004
10.1016/j.jallcom.2013.10.095
10.1016/j.msea.2010.01.006
10.1016/j.actamat.2013.09.021
10.1016/0036-9748(82)90333-7
10.1016/j.msea.2017.06.072
10.1016/j.jare.2016.08.002
10.1016/j.matchar.2015.06.011
10.1016/j.msea.2005.08.206
10.1016/j.simpat.2016.05.001
10.1016/j.msea.2017.09.121
10.1016/j.jallcom.2010.06.030
10.1016/j.msea.2012.07.063
10.1016/j.mechmat.2019.103194
10.1016/j.ijfatigue.2017.12.020
10.1016/j.scriptamat.2010.03.006
10.1016/j.msea.2006.10.150
10.1016/0036-9748(74)90406-2
10.1016/j.actamat.2012.01.049
10.1016/j.msea.2017.02.047
10.1016/0921-5093(95)09899-2
10.1016/j.matdes.2019.107584
10.1016/j.msea.2013.10.051
10.1016/0140-6701(95)95581-0
10.1016/S0261-3069(99)00069-2
10.1016/j.jnucmat.2009.08.007
10.1016/j.ijfatigue.2019.105337
10.1016/j.matlet.2004.05.041
10.1016/j.apsusc.2006.05.027
10.1016/s1468-6996(02)00005-0
10.1016/j.matpr.2018.06.184
10.1016/j.msea.2004.01.027
10.1016/s1644-9665(12)60180-0
10.1016/j.msea.2013.10.092
10.1016/j.advengsoft.2017.10.008
10.1016/j.ceramint.2019.08.078
10.1016/j.actamat.2016.11.021
10.1016/S0925-8388(01)01573-0
10.1016/j.msea.2018.10.032
10.1016/j.actamat.2010.09.030
10.1016/j.optlaseng.2004.06.013
10.1016/S1006-706X(17)30094-8

10.1016/0036-9748(86)90245-0
10.1016/j.commatsci.2009.08.012
10.1016/s0036-9748(88)80299-0
10.1016/j.msea.2013.01.012
10.1016/S0921-5093(96)10556-6
10.1016/j.matdes.2004.10.017
10.1016/j.apsusc.2005.05.054
10.1016/0022-3115(87)90113-9
10.1016/j.apsusc.2011.06.005
10.1016/j.actamat.2017.03.067
10.1016/0025-5416(84)90262-3
10.1016/j.matdes.2018.04.032
10.1016/j.msea.2015.12.024
10.1016/j.precisioneng.2014.11.012
10.1016/j.actamat.2013.05.032
10.1016/j.jallcom.2018.07.154
10.1016/j.finel.2016.01.001
10.1016/j.engfracmech.2018.05.023
10.1016/j.msea.2017.07.025
10.1016/j.jallcom.2011.09.094
10.1016/j.jallcom.2019.01.048
10.1016/j.ijfatigue.2019.01.014
10.1016/j.surfcoat.2018.01.071
10.1016/j.intermet.2009.04.007
10.1016/j.ceramint.2016.08.013
10.1016/j.actamat.2007.05.021
10.1016/0257-8972(87)90115-0
10.1016/j.tafmec.2017.10.010
10.1016/j.jallcom.2020.154474
10.1016/j.scriptamat.2018.04.020
10.1016/j.ijfatigue.2017.07.023
10.1016/j.ijfatigue.2019.04.032
10.1016/j.ijmecsci.2018.02.042
10.1016/j.pnsc.2019.12.006
10.1016/j.ijfatigue.2019.02.003
10.1016/j.jmapro.2019.09.030
10.1016/j.ijfatigue.2008.01.004
10.1016/j.corsci.2014.03.011
10.1016/j.actamat.2008.12.026
10.1016/j.jallcom.2017.07.198
10.1016/j.ijfatigue.2007.01.034
10.1016/j.actamat.2007.09.024
10.1016/0921-5093(93)90402-z
10.1016/j.surfcoat.2010.01.024
10.1016/j.jmatprotec.2015.11.017
10.1016/S1006-706X(13)60083-7
10.1016/j.matlet.2014.01.072
10.1016/0254-0584(96)80117-9
10.1016/0010-938x(87)90093-x
10.1016/j.procir.2015.06.086
10.1016/j.ijfatigue.2016.06.023
10.1016/0921-5093(92)90133-l
10.1016/j.jallcom.2019.02.101
10.1016/S1006-706X(15)30054-6

10.1016/S0927-0256(98)00053-6
10.1016/j.wear.2019.01.090
10.1016/j.jallcom.2017.05.015
10.1016/j.actamat.2008.04.014
10.1016/j.nimb.2009.07.010
10.1016/j.jallcom.2017.01.104
10.1016/j.commatsci.2010.11.008
10.1016/j.matdes.2015.08.036
10.1016/S1359-6462(99)00261-4
10.1016/j.jallcom.2013.03.085
10.1016/j.msea.2008.10.056
10.1016/j.engfracmech.2015.12.014
10.1016/j.ijmachtools.2017.04.013
10.1016/j.triboint.2016.02.011
10.1016/j.msea.2008.08.005
10.1016/j.vacuum.2019.108942
10.1016/j.matchar.2018.04.039
10.1016/S1006-706X(14)60059-5
10.1016/S0921-5093(98)00791-6
10.1016/j.actamat.2016.05.016
10.1016/j.surfcoat.2011.07.044
10.1016/S1359-6462(00)00702-8
10.1016/j.surfcoat.2015.02.050
10.1016/j.matchar.2015.07.035
10.1016/j.jmatprotec.2018.07.034
10.1016/j.vacuum.2018.01.032
10.1016/j.ijfatigue.2016.01.024
10.1016/S1006-706X(15)30065-0
10.1016/j.msea.2012.01.049
10.1016/S0301-679X(02)00144-5
10.1016/j.ceramint.2016.06.124
10.1016/j.msea.2017.01.058
10.1016/j.applthermaleng.2016.07.167
10.1016/j.surfcoat.2017.11.008
10.1016/j.corsci.2019.05.009
10.1016/0956-7151(90)90222-3
10.1016/S0022-3115(01)00519-0
10.1016/S0921-5093(03)00329-0
10.1016/s1468-6996(01)00034-1
10.1016/j.msea.2019.138355
10.1016/j.jallcom.2017.11.074
10.1016/j.surfcoat.2007.08.025
10.1016/1359-6462(96)00109-1
10.1016/j.scriptamat.2018.04.037
10.1016/j.msea.2013.06.088
10.1016/j.matchemphys.2010.02.046
10.1016/j.msea.2012.11.063
10.1016/j.msea.2006.12.107
10.1016/j.matdes.2014.12.032
10.1016/j.msea.2019.02.009
10.1016/j.jestch.2018.03.018
10.1016/S0921-5093(97)00465-6
10.1016/S1002-0721(14)60535-4
10.1016/0921-5093(90)90151-r

10.1016/j.matchar.2018.05.010
10.1016/j.engfailanal.2016.05.033
10.1016/S1003-6326(14)63381-1
10.1016/S0921-5093(98)00546-2
10.1016/j.jallcom.2017.02.210
10.1016/0142-1123(82)90063-9
10.1016/j.matchar.2017.05.037
10.1016/j.jallcom.2019.07.284
10.1016/j.commatsci.2013.02.026
10.1016/S0254-0584(02)00316-4
10.1016/j.ceramint.2017.11.190
10.1016/j.ijheatmasstransfer.2007.05.017
10.1016/j.jallcom.2015.04.008
10.1016/j.matdes.2011.11.035
10.1016/0956-716X(95)90839-C
10.1016/j.msea.2010.10.024
10.1016/j.matchar.2020.110241
10.1016/0022-3115(91)90360-j
10.1016/j.applthermaleng.2016.12.032
10.1016/j.msea.2016.12.043
10.1016/j.procir.2013.06.083
10.1016/0956-716x(91)90076-d
10.1016/j.measurement.2019.06.006
10.1016/j.solmat.2018.12.020
10.1016/0956-716x(93)90463-3
10.1016/j.ijfatigue.2005.06.006
10.1016/j.msea.2018.08.030
10.1016/j.jallcom.2017.05.042
10.1016/j.msea.2016.03.119
10.1016/S0749-6419(98)80004-1
10.1016/j.surfcoat.2019.03.058
10.1016/j.matchar.2017.02.030
10.1016/j.surfcoat.2019.125018
10.1016/j.msea.2016.03.041
10.1016/j.msea.2019.138448
10.1016/j.ssi.2003.11.026
10.1016/j.scriptamat.2019.09.020
10.1016/0022-3115(84)90063-1
10.1016/j.msea.2011.11.066
10.1016/1044-5803(95)80108-1
10.1016/j.jallcom.2020.154012
10.1016/S0257-8972(02)00559-5
10.1016/j.scriptamat.2008.09.009
10.1016/j.msea.2017.05.091
10.1016/S0921-5093(98)00990-3
10.1016/j.jmatprotec.2018.01.014
10.1016/j.matchar.2018.03.023
10.1016/j.proeng.2013.03.229
10.1016/j.jmatprotec.2010.05.010
10.1016/j.ijfatigue.2019.04.023
10.1016/j.matdes.2019.108359
10.1016/j.jmatprotec.2006.07.009
10.1016/j.colsurfa.2014.06.033
10.1016/j.matlet.2019.05.012

10.1016/j.msea.2018.07.106
10.1016/j.jallcom.2009.04.105
10.1016/j.wear.2010.08.003
10.1016/j.jmatprotec.2019.116297
10.1016/j.jallcom.2019.05.001
10.1016/j.matdes.2013.04.024
10.1016/j.actamat.2015.04.053
10.1016/j.jmps.2016.05.019
10.1016/S1005-0302(11)60123-0
10.1016/j.scriptamat.2018.06.036
10.1016/j.addma.2018.10.046
10.1016/j.matdes.2017.11.065
10.1016/j.ijfatigue.2006.06.010
10.1016/S0022-3115(00)00308-1
10.1016/j.surfcoat.2017.07.061
10.1016/S1359-6462(99)00157-8
10.1016/j.intermet.2007.10.001
10.1016/j.jmst.2016.11.026
10.1063/2.1203109
10.1016/S1359-6454(97)00451-5
10.1016/j.surfcoat.2010.04.041
10.1016/j.proeng.2010.03.081
10.1016/j.actamat.2008.08.052
10.1016/0921-5093(92)90236-t
10.1016/S1359-6462(97)00520-4
10.1016/j.msea.2012.11.084
10.1016/j.surfcoat.2007.12.008
10.1016/0022-5088(72)90100-2
10.1016/j.jmst.2017.05.002
10.1016/S1003-6326(16)64323-6
10.1016/0167-577X(95)00172-7
10.1016/j.ijfatigue.2017.08.009
10.1016/j.optlastec.2016.07.020
10.1016/j.matchar.2010.04.007
10.1016/j.scriptamat.2014.12.019
10.1016/j.pmatsci.2012.10.001
10.1016/j.jmatprotec.2012.07.021
10.1016/j.matdes.2017.10.016
10.1016/j.jmatprotec.2007.12.091
10.1016/S1359-6462(99)00316-4
10.1016/j.jallcom.2019.152289
10.1016/j.matdes.2014.04.055
10.1016/j.jmapro.2016.10.002
10.1016/0030-3992(95)00086-0
10.1016/j.wear.2008.12.082
10.1016/j.msea.2013.08.065
10.1016/j.msea.2008.10.057
10.1016/j.measurement.2019.05.035
10.1016/j.msea.2016.09.100
10.1016/j.actamat.2018.05.067
10.1016/j.matchemphys.2018.03.061
10.1016/j.apsusc.2004.02.030
10.1016/j.matdes.2005.03.015
10.1016/j.matdes.2005.09.015

10.1016/j.jallcom.2015.07.016
10.1016/j.msea.2016.10.050
10.1016/0040-6090(79)90522-4
10.1016/0257-8972(89)90036-4
10.1016/j.surfcoat.2013.04.020
10.1016/j.advengsoft.2016.07.001
10.1016/j.proeng.2011.04.009
10.1016/S0921-5093(97)00827-7
10.1016/j.commatsci.2017.04.031
10.1016/0921-5093(94)90925-3
10.1016/j.ijmachtools.2016.05.008
10.1016/j.vacuum.2012.02.003
10.1016/j.jallcom.2009.05.007
10.1016/j.jallcom.2015.09.219
10.1016/j.matdes.2018.09.012
10.1016/j.actamat.2005.01.025
10.1016/j.actamat.2017.02.059
10.1016/0001-6160(83)90150-5
10.1016/j.surfcoat.2008.09.031
10.1016/0257-8972(90)90089-u
10.1016/j.msea.2005.08.157
10.1016/j.jallcom.2004.07.048
10.1016/j.jmbbm.2019.103496
10.1016/j.matdes.2017.05.025
10.1016/j.engfracmech.2020.106889
10.1016/j.matlet.2012.04.042
10.1016/S0167-577X(03)00078-8
10.1016/j.jallcom.2014.10.038
10.1016/j.ijmecsci.2019.01.007
10.1016/S0261-3069(03)00061-X
10.1016/j.corsci.2011.06.022
10.1016/S0955-2219(99)00028-X
10.1016/S0026-0657(99)80018-6
10.1016/j.msea.2008.06.056
10.1016/j.ceramint.2017.03.074
10.1016/j.commatsci.2016.07.023
10.1016/j.scriptamat.2019.07.028
10.1016/S1005-0302(12)60015-2
10.1016/j.msea.2014.05.044
10.1016/j.surfcoat.2005.10.037
10.1016/j.ijmecsci.2019.105126
10.1016/s0036-9748(88)80112-1
10.1016/j.jallcom.2017.10.226
10.1016/j.actamat.2005.06.013
10.1016/j.scriptamat.2003.12.013
10.1016/S0921-5093(99)00235-X
10.1016/j.ijimpeng.2019.05.001
10.1016/j.ijfatigue.2011.07.005
10.1016/j.scriptamat.2009.03.042
10.1016/j.surfcoat.2006.05.005
10.1016/j.msea.2018.08.007
10.1016/j.jmst.2019.05.003
10.1016/j.dental.2016.05.011
10.1016/j.jclepro.2018.11.104

10.1016/j.msea.2005.02.050
10.1016/0025-5416(87)90246-1
10.1016/S1000-9361(08)60072-2
10.1016/j.electacta.2012.05.016
10.1016/j.apm.2017.09.043
10.1016/j.jallcom.2019.01.237
10.1016/0013-7944(94)00274-L
10.1016/j.msea.2011.12.011
10.1016/j.engfailanal.2019.07.016
10.1016/j.scriptamat.2012.03.042
10.1016/j.jallcom.2009.02.130
10.1016/J.ENG.2017.05.011
10.1016/j.vacuum.2010.09.006
10.1016/j.msea.2018.10.033
10.1016/j.jnucmat.2012.08.012
10.1016/j.jallcom.2017.12.233
10.1016/j.corsci.2015.07.031
10.1016/j.msea.2019.138670
10.1016/j.surfcoat.2006.03.043
10.1016/j.actamat.2004.12.032
10.1016/j.jmatprotec.2015.06.026
10.1016/j.matdes.2015.05.060
10.1016/j.vacuum.2018.10.074
10.1016/j.apsusc.2012.11.070
10.1016/j.jallcom.2014.06.099
10.1016/0025-5416(82)90023-4
10.1016/j.jclepro.2015.12.102
10.1016/j.msea.2012.06.081
10.1016/j.ultramic.2014.04.001
10.1016/S0257-8972(02)00902-7
10.1016/0956-7151(92)90295-p
10.1016/j.tsf.2016.08.009
10.1016/j.scriptamat.2017.12.028
10.1016/j.jallcom.2019.01.093
10.1016/j.surfcoat.2015.05.046
10.1016/S0921-5093(99)00122-7
10.1016/j.msea.2019.138849
10.1016/j.corsci.2010.01.009
10.1016/j.ijhydene.2006.08.056
10.1016/S0142-1123(02)00162-7
10.1016/j.actamat.2015.05.052
10.1016/j.msea.2015.04.068
10.1016/j.scriptamat.2011.03.007
10.1016/j.precisioneng.2014.08.002
10.1016/j.mspro.2014.07.402
10.1016/S0921-5093(97)00341-9
10.1016/j.jallcom.2018.07.317
10.1016/j.scriptamat.2015.07.018
10.1016/S1359-6454(96)00265-0
10.1016/0921-5093(93)90470-y
10.1016/j.mechrescom.2013.10.013
10.1016/j.ceramint.2019.05.018
10.1016/j.jallcom.2016.10.019
10.1016/j.jmatprotec.2004.04.280

10.1016/j.surfcoat.2012.03.061
10.1016/j.msea.2017.09.021
10.1016/j.matdes.2015.06.089
10.1016/j.pmatsci.2009.04.002
10.1016/j.scriptamat.2008.07.031
10.1016/S1359-6454(98)00378-4
10.1016/j.msea.2016.08.121
10.1016/j.msea.2019.138631
10.1016/j.physb.2006.06.108
10.1016/j.corsci.2015.05.030
10.1016/j.surfcoat.2006.07.257
10.1016/j.jallcom.2004.08.095
10.1016/j.ijfatigue.2018.09.007
10.1016/j.msea.2007.06.041
10.1016/0921-5093(96)80002-5
10.1016/j.jallcom.2011.03.038
10.1016/j.corsci.2008.05.010
10.1016/j.scriptamat.2011.11.037
10.1016/S0921-5093(02)00128-4
10.1016/0001-6160(83)90194-3
10.1016/s1875-5372(14)60140-5
10.1016/S1003-6326(17)60176-6
10.1016/j.ceramint.2017.09.148
10.1016/S0921-5093(99)00167-7
10.1016/j.cirpj.2017.04.001
10.1016/j.actamat.2018.11.014
10.1016/j.surfcoat.2013.07.068
10.1016/0026-0657(95)93658-0
10.1016/j.matdes.2014.06.063
10.1016/j.actamat.2018.09.052
10.1016/S0142-1123(97)84398-8
10.1016/j.cossms.2011.03.001
10.1016/j.vacuum.2015.11.025
10.1016/j.jmatprotec.2018.01.022
10.1016/0956-716X(95)00234-M
10.1016/S1003-6326(07)60044-2
10.1016/0026-0800(72)90071-7
10.1016/S1359-6454(98)00172-4
10.1016/j.engfailanal.2014.09.001
10.1016/j.matpr.2020.01.562
10.1016/S0263-4368(00)00021-4
10.1016/j.scriptamat.2009.07.034
10.1016/j.jmatprotec.2008.09.014
10.1016/0257-8972(94)02398-0
10.1016/j.actamat.2017.10.029
10.1016/j.jallcom.2018.12.186
10.1016/j.actamat.2013.03.003
10.1016/j.matchar.2018.08.055
10.1016/j.cirp.2015.05.002
10.1016/j.actamat.2013.02.050
10.1016/j.matchar.2010.06.001
10.1016/j.msea.2016.03.059
10.1016/0257-8972(92)90371-g
10.1016/j.msea.2018.11.087

10.1016/j.commatsci.2008.05.030
10.1016/0013-7944(87)90047-6
10.1016/0022-3115(84)90588-9
10.1016/j.jmst.2016.08.018
10.1016/S1359-6454(00)00367-0
10.1016/j.jeurceramsoc.2019.03.002
10.1016/j.msea.2013.05.048
10.1016/j.msea.2016.12.088
10.1016/j.actamat.2010.09.035
10.1016/j.msea.2019.138477
10.1016/1044-5803(92)90089-z
10.1016/j.jmrt.2019.12.011
10.1016/j.msea.2006.08.148
10.1016/j.apsusc.2019.144477
10.1016/j.jallcom.2008.07.071
10.1016/S1359-6454(99)00456-5
10.1016/j.ijfatigue.2018.09.017
10.1016/j.seppur.2018.11.023
10.1016/j.ijrmhm.2015.03.006
10.1016/j.surfcoat.2014.04.024
10.1016/j.ijfatigue.2019.06.026
10.1016/j.msea.2012.12.015
10.1016/j.ijplas.2010.01.003
10.1016/0956-716x(91)90316-s
10.1016/j.commatsci.2008.07.021
10.1016/j.procir.2015.06.067
10.1016/j.jallcom.2019.152777
10.1016/j.jmst.2019.01.004
10.1016/S1003-6326(16)64304-2
10.1016/S0022-3115(01)00516-5
10.1016/S0029-5493(97)00064-2
10.1016/j.solmat.2015.11.024
10.1016/j.tafmec.2015.11.008
10.1016/j.jallcom.2018.09.371
10.1016/j.matchar.2018.11.014
10.1016/j.ijfatigue.2017.09.002
10.1016/j.actamat.2010.11.051
10.1016/j.triboint.2009.04.006
10.1016/j.actaastro.2012.02.028
10.1016/j.corsci.2017.12.021
10.1016/j.jmapro.2015.09.006
10.1016/j.ijfatigue.2018.12.024
10.1016/j.surfcoat.2004.09.022
10.1016/j.calphad.2012.04.003
10.1016/0169-4332(94)90339-5
10.1016/j.surfcoat.2016.01.062
10.1016/j.actamat.2005.11.004
10.1016/j.msea.2009.01.009
10.1016/j.msea.2018.06.106
10.1016/j.surfcoat.2016.02.056
10.1016/j.intermet.2006.08.015
10.1016/j.jallcom.2014.04.174
10.1016/j.procir.2018.05.068
10.1016/0026-0800(88)90018-3

10.1016/j.vacuum.2019.108878
10.1016/j.matlet.2019.127008
10.1016/j.actamat.2010.03.041
10.1016/S1003-6326(08)60050-3
10.1016/j.scriptamat.2015.02.020
10.1016/j.engfailanal.2019.01.038
10.1016/S1359-6454(01)00103-3
10.1016/j.msea.2007.04.098
10.1016/0013-7944(84)90136-x
10.1016/j.actamat.2012.03.023
10.1016/0378-4363(86)90694-7
10.1016/j.engfracmech.2015.01.027
10.1016/j.mtla.2019.100319
10.1016/j.matpr.2018.02.239
10.1016/S0257-8972(99)00503-4
10.1016/j.jallcom.2013.09.193
10.1016/j.msea.2005.08.095
10.1016/j.matchar.2019.110092
10.1016/j.ijfatigue.2011.12.011
10.1016/S1002-0071(12)60052-5
10.1016/0036-9748(84)90096-6
10.1016/0025-5416(79)90065-x
10.1016/j.proeng.2017.10.1107
10.1016/S0254-0584(02)00197-9
10.1016/j.msea.2010.07.036
10.1016/j.jallcom.2014.10.088
10.1016/0921-5093(91)90653-5
10.1016/j.matdes.2016.04.093
10.1016/j.tafmec.2013.11.002
10.1016/j.actamat.2018.06.048
10.1016/j.measurement.2017.01.057
10.1016/j.corsci.2010.06.026
10.1016/j.actamat.2009.08.053
10.1016/0022-3115(82)90524-4
10.1016/j.optlastec.2016.06.014
10.1016/j.jmst.2013.01.002
10.1016/S1572-4859(02)80014-6
10.1016/0026-0800(73)90018-9
10.1016/j.jallcom.2007.09.019
10.1016/j.actamat.2014.01.002
10.1016/j.msea.2011.12.112
10.1016/j.jallcom.2020.154590
10.1016/j.mtla.2018.08.037
10.1016/S1359-6454(01)00285-3
10.1016/j.optlastec.2019.105834
10.1016/j.jallcom.2019.152773
10.1016/j.solmat.2017.05.050
10.1016/0029-5493(88)90281-6
10.1016/j.jallcom.2019.03.366
10.1016/S0007-8506(07)62091-5
10.1016/j.actamat.2019.08.035
10.1016/j.msea.2012.03.073
10.1016/S0921-5093(03)00079-0
10.1016/S0026-0657(02)80307-1

10.1016/j.actamat.2012.05.014
10.1016/S1359-6454(96)00096-1
10.1016/j.ijfatigue.2007.09.010
10.1016/j.msea.2017.04.076
10.1016/j.pnsc.2018.01.003
10.1016/0001-6160(86)90247-6
10.1016/0026-0800(75)90040-3
10.1016/j.proeng.2014.06.260
10.1016/0036-9748(89)90138-5
10.1016/j.actamat.2017.12.020
10.1016/j.actamat.2016.09.055
10.1016/S0921-5093(97)00763-6
10.1016/j.surfcoat.2016.09.057
10.1016/j.jallcom.2018.04.313
10.1016/j.commatsci.2018.11.027
10.1016/j.commatsci.2013.10.007
10.1016/j.surfcoat.2013.10.011
10.1016/S1359-6462(99)00065-2
10.1016/j.mechmat.2019.103170
10.1016/j.measurement.2016.02.023
10.1016/j.actamat.2018.01.034
10.1016/j.intermet.2010.05.002
10.1016/j.surfcoat.2007.07.055
10.1016/s0036-9748(88)80253-9
10.1016/S1566-1369(02)80074-7
10.1016/j.measurement.2016.06.023
10.1016/j.cja.2019.09.012
10.1016/j.actamat.2012.09.013
10.1016/j.ijfatigue.2011.12.002
10.1016/1359-6454(95)00315-0
10.1016/j.msea.2005.07.027
10.1016/j.actamat.2011.07.002
10.1016/0142-9612(95)99703-0
10.1016/j.matchar.2019.01.033
10.1016/j.jmst.2018.09.005
10.1016/j.jmst.2018.02.002
10.1016/S1359-6462(99)00049-4
10.1016/S1006-706X(16)30130-3
10.1016/j.msea.2015.06.094
10.1016/j.jare.2017.05.004
10.1016/j.msea.2009.12.037
10.1016/S0921-5093(99)00453-0
10.1016/S1359-6462(97)00389-8
10.1016/S1003-6326(09)60216-8
10.1016/j.msea.2018.08.032
10.1016/j.msea.2017.06.018
10.1016/j.matchemphys.2004.01.004
10.1016/0142-1123(95)00006-F
10.1016/j.corsci.2016.04.051
10.1016/j.jallcom.2018.07.035
10.1016/j.jallcom.2006.12.116
10.1016/S1359-6462(01)01244-1
10.1016/j.actamat.2009.08.018
10.1016/j.electacta.2013.08.019

10.1016/S1359-6462(97)00064-X
10.1016/j.ijfatigue.2016.09.002
10.1016/j.jmatprotec.2015.03.031
10.1016/S0927-0256(97)00061-X
10.1016/S0142-1123(01)00031-7
10.1016/j.surfcoat.2009.02.127
10.1016/j.matdes.2017.06.007
10.1016/j.engfailanal.2010.09.024
10.1016/j.matchar.2014.12.012
10.1016/j.surfcoat.2017.09.071
10.1016/j.intermet.2016.03.006
10.1016/0956-7151(94)00432-H
10.1016/S1369-7021(06)71555-3
10.1016/j.jallcom.2018.12.211
10.1016/j.msea.2005.05.099
10.1016/j.jmapro.2018.03.002
10.1016/j.ijfatigue.2019.05.007
10.1016/j.engfracmech.2014.04.006
10.1016/S1359-6454(96)00324-2
10.1016/j.msea.2015.03.063
10.1016/j.jmatprotec.2018.10.021
10.1016/j.commatsci.2015.05.020
10.1016/j.corsci.2012.09.045
10.1016/j.msea.2012.04.008
10.1016/j.matdes.2013.04.101
10.1016/j.surfcoat.2013.08.033
10.1016/j.jallcom.2014.05.165
10.1016/S1359-6462(02)00556-0
10.1016/j.msea.2015.07.045
10.1016/j.measurement.2018.12.010
10.1016/j.msea.2016.10.122
10.1016/j.commatsci.2018.06.014
10.1016/j.surfcoat.2006.02.047
10.1016/j.pnsc.2019.01.002
10.1016/j.surfcoat.2006.04.020
10.1016/j.applthermaleng.2016.07.008
10.1016/S1359-6454(03)00105-8
10.1016/S0010-938X(97)86103-3
10.1016/j.msea.2015.04.060
10.1016/j.msea.2017.11.038
10.1016/0036-9748(73)90251-2
10.1016/j.msea.2014.09.074
10.1016/S0921-5093(97)00618-7
10.1016/j.actamat.2012.01.054
10.1016/j.actamat.2011.12.008
10.1016/j.ijpvp.2004.03.007
10.1016/j.matchar.2016.11.002
10.1016/0956-716X(95)00179-Y
10.1016/j.matdes.2010.02.021
10.1016/j.jmst.2018.09.024
10.1016/S1005-0302(12)60169-8
10.1016/j.optlastec.2018.07.018
10.1016/j.msea.2019.03.099
10.1016/j.surfcoat.2016.07.047

10.1016/j.jmapro.2019.04.033
10.1016/0022-3115(85)90056-x
10.1016/j.actamat.2014.08.049
10.1016/j.phpro.2014.08.144
10.1016/S1003-6326(14)63245-3
10.1016/0257-8972(95)02539-1
10.1016/j.msea.2017.10.085
10.1016/j.jmst.2014.11.008
10.1016/j.nimb.2015.07.080
10.1016/S0924-0136(97)00452-4
10.1016/j.jallcom.2016.11.188
10.1016/S0921-5093(01)01577-5
10.1016/j.jmst.2020.01.041
10.1016/j.mechmat.2019.04.024
10.1016/j.apsusc.2020.145423
10.1016/j.jmatprotec.2013.06.007
10.1016/j.ijfatigue.2017.01.036
10.1016/j.commatsci.2012.03.049
10.1016/j.jallcom.2014.06.172
10.1016/j.jmatprotec.2013.10.001
10.1016/j.actamat.2007.12.010
10.1016/j.ijfatigue.2020.105652
10.1016/j.jpowsour.2010.07.081
10.1016/S0142-1123(02)00043-9
10.1016/j.vacuum.2019.108849
10.1016/j.actamat.2009.06.019
10.1016/0025-5416(86)90288-0
10.1016/j.ultramic.2018.08.015
10.1016/j.jallcom.2017.07.309
10.1016/j.msea.2010.09.035
10.1016/0921-5093(89)90853-8
10.1016/j.msea.2003.11.052
10.1016/j.matchar.2014.12.008
10.1016/j.intermet.2016.04.003
10.1016/j.intermet.2017.11.005
10.1016/j.jallcom.2017.03.102
10.1016/j.jmatprotec.2012.06.017
10.1016/j.actamat.2019.02.012
10.1016/j.vacuum.2017.05.010
10.1016/j.jallcom.2017.02.013
10.1016/j.msea.2008.03.032
10.1016/j.engfracmech.2015.06.003
10.1016/0956-716x(94)90569-x
10.1016/j.surfcoat.2007.09.048
10.1016/j.actamat.2015.02.023
10.1016/j.tafmec.2015.05.008
10.1016/j.actamat.2016.01.028
10.1016/j.matdes.2010.12.014
10.1016/j.matdes.2019.108117
10.1016/j.jallcom.2017.03.259
10.1016/j.commatsci.2018.01.038
10.1016/j.vacuum.2018.02.034
10.1016/j.pnsc.2018.07.003
10.1016/j.matchar.2018.02.031

10.1016/j.ijheatmasstransfer.2017.09.062
10.1016/j.corsci.2019.108138
10.1016/j.actamat.2004.08.039
10.1016/j.actamat.2012.06.027
10.1016/j.jmst.2016.07.017
10.1016/S0749-6419(03)00077-9
10.1016/S0254-0584(02)00551-5
10.1016/j.msea.2015.12.089
10.1016/S0010-938X(97)83343-4
10.1016/j.engfailanal.2010.08.015
10.1016/j.msea.2016.03.069
10.1016/j.ijfatigue.2016.02.035
10.1016/j.jallcom.2011.10.106
10.1016/j.matchar.2015.07.027
10.1016/j.apt.2018.06.012
10.1016/S0924-0136(02)01063-4
10.1016/j.jallcom.2019.01.289
10.1016/j.msea.2016.11.074
10.1016/j.commatsci.2017.11.047
10.1016/S0955-2219(01)00234-5
10.1016/j.jmst.2013.12.010
10.1016/0956-7151(94)00357-N
10.1016/j.jallcom.2008.01.156
10.1016/j.msea.2011.12.034
10.1016/S0927-0256(97)00075-X
10.1016/j.tsf.2005.08.079
10.1016/j.msea.2018.04.073
10.1016/j.msea.2015.06.002
10.1016/S0921-5093(98)01120-4
10.1016/0043-1648(85)90108-5
10.1016/j.jmps.2017.10.005
10.1016/j.matdes.2019.107760
10.1016/0001-6160(83)90182-7
10.1016/j.matchemphys.2018.08.004
10.1016/j.matchar.2020.110200
10.1016/j.vacuum.2020.109204
10.1016/S0921-5093(98)00541-3
10.1016/j.actamat.2003.12.017
10.1016/S0921-5093(96)10572-4
10.1016/j.msea.2007.07.028
10.1016/S1359-6454(99)00083-X
10.1016/j.optlastec.2017.10.029
10.1016/j.matchar.2012.02.021
10.1016/j.jallcom.2019.04.054
10.1016/j.jallcom.2012.02.158
10.1016/j.ijplas.2013.08.009
10.1016/j.jmatprotec.2007.10.084
10.1016/1359-6462(96)00240-0
10.1016/j.actamat.2010.07.004
10.1016/j.infrared.2016.11.014
10.1016/j.jmatprotec.2015.06.001
10.1016/0001-6160(89)90141-7
10.1016/j.matchar.2016.06.001
10.1016/j.net.2018.05.009

10.1016/j.msea.2004.11.028
10.1016/j.msea.2006.12.123
10.1016/0025-5416(83)90194-5
10.1016/0022-3115(90)90005-8
10.1016/j.matlet.2014.04.185
10.1016/0257-8972(94)90107-4
10.1016/0921-5093(94)90503-7
10.1016/S0010-938X(02)00143-9
10.1016/j.addma.2017.02.002
10.1016/j.vacuum.2018.06.059
10.1016/j.actamat.2016.04.012
10.1016/j.actamat.2004.06.041
10.1016/0036-9748(86)90105-5
10.1016/j.msea.2014.02.079
10.1016/j.matdes.2015.07.136
10.1016/j.tafmec.2018.07.003
10.1016/0025-5416(73)90007-4
10.1016/j.intermet.2010.08.016
10.1016/j.ijplas.2015.03.007
10.1016/0025-5416(88)90791-4
10.1016/j.surfcoat.2007.03.029
10.1016/S0142-1123(02)00023-3
10.1016/j.matchar.2019.05.009
10.1016/j.ndteint.2007.01.003
10.1016/0001-6160(88)90120-4
10.1016/0036-9748(87)90369-3
10.1016/S0010-938X(96)00112-6
10.1016/j.intermet.2018.04.010
10.1016/j.msea.2007.02.102
10.1016/j.polymertesting.2017.07.032
10.1016/S1003-6326(16)64427-8
10.1016/j.jeurceramsoc.2012.05.002
10.1016/j.pnsc.2012.04.007
10.1016/j.msea.2009.12.035
10.1016/0921-5093(89)90734-x
10.1016/j.jallcom.2019.05.180
10.1016/j.surfcoat.2011.06.023
10.1016/j.jmatprotec.2014.07.020
10.1016/j.jmatprotec.2011.09.001
10.1016/j.intermet.2004.02.041
10.1016/j.corsci.2013.06.033
10.1016/j.ijfatigue.2011.09.003
10.1016/j.msea.2008.07.015
10.1016/0956-716x(92)90263-e
10.1016/j.surfcoat.2016.12.097
10.1016/j.corsci.2013.08.007
10.1016/j.actamat.2009.01.006
10.1016/j.surfcoat.2019.01.001
10.1016/S1006-706X(12)60115-0
10.1016/j.jnucmat.2012.11.057
10.1016/j.jallcom.2010.07.142
10.1016/j.ijfatigue.2018.02.020
10.1016/S1359-6454(02)00204-5
10.1016/j.msea.2005.02.042

10.1016/S1359-6462(98)00277-2
10.1016/j.commatsci.2018.04.043
10.1016/j.optlastec.2017.08.027
10.1016/j.surfcoat.2018.12.113
10.1016/j.msea.2005.01.048
10.1016/j.pnsc.2018.01.009
10.1016/0025-5416(87)90166-2
10.1016/j.jallcom.2013.09.078
10.1016/0036-9748(82)90254-x
10.1016/j.commatsci.2016.12.005
10.1016/j.triboint.2016.09.039
10.1016/0040-6090(82)90302-9
10.1016/j.jmst.2015.12.021
10.1016/S0257-8972(99)00342-4
10.1016/j.jallcom.2017.06.270
10.1016/j.apsusc.2015.03.157
10.1016/j.matchemphys.2006.03.012
10.1016/j.ijsolstr.2013.12.013
10.1016/j.intermet.2013.08.002
10.1016/j.jpowsour.2008.11.041
10.1016/0025-5416(87)90403-4
10.1016/j.actamat.2007.12.022
10.1016/j.powtec.2019.06.004
10.1016/j.commatsci.2019.02.017
10.1016/j.ndteint.2007.06.001
10.1016/0169-4332(93)90328-9
10.1016/j.engfailanal.2007.11.010
10.1016/0956-716x(94)90115-5
10.1016/j.matchar.2007.08.004
10.1016/0025-5416(77)90142-2
10.1016/S0257-8972(01)01478-5
10.1016/j.matdes.2017.05.023
10.1016/j.msea.2018.05.049
10.1016/j.jmatprotec.2018.07.012
10.1016/j.engfracmech.2015.08.007
10.1016/j.msea.2008.09.038
10.1016/j.msea.2017.10.024
10.1016/j.scriptamat.2019.12.006
10.1016/j.pnsc.2017.02.008
10.1016/0025-5416(87)90339-9
10.1016/j.jallcom.2009.07.046
10.1016/j.matpr.2018.02.361
10.1016/j.matdes.2013.11.075
10.1016/j.solmat.2017.02.020
10.1016/j.surfcoat.2018.06.021
10.1016/j.corsci.2014.07.053
10.1016/S1003-6326(11)60860-1
10.1016/S0925-8388(03)00321-9
10.1016/j.ijfatigue.2007.01.047
10.1016/j.actamat.2014.04.039
10.1016/j.msea.2014.06.021
10.1016/0025-5416(84)90071-5
10.1016/j.mechmat.2011.07.007
10.1016/j.scriptamat.2007.09.050

10.1016/0921-5093(94)90476-6
10.1016/S1359-6454(03)00221-0
10.1016/0921-5093(92)90290-h
10.1016/j.jallcom.2013.01.098
10.1016/0956-716x(91)90405-p
10.1016/S0263-4368(00)00030-5
10.1016/j.pnsc.2012.03.007
10.1016/j.commatsci.2011.07.034
10.1016/j.matdes.2015.06.068
10.1016/j.matchar.2019.02.021
10.1016/j.matdes.2005.08.004
10.1016/S1003-6326(15)64031-6
10.1016/0956-7151(90)90293-p
10.1016/j.scriptamat.2014.11.029
10.1016/j.jallcom.2014.05.201
10.1016/0142-1123(96)82809-X
10.1016/j.matdes.2014.12.038
10.1016/0956-7151(92)90405-4
10.1016/0039-6028(92)91037-c
10.1016/j.msea.2019.138186
10.1016/j.jallcom.2003.10.068
10.1016/j.electacta.2010.05.079
10.1016/j.corsci.2019.05.036
10.1016/j.proeng.2017.01.179
10.1016/j.jallcom.2015.12.086
10.1016/j.jallcom.2015.04.099
10.1016/S0921-5093(03)00148-5
10.1016/j.msea.2017.05.085
10.1016/j.jallcom.2017.03.179
10.1016/j.ijrmhm.2019.04.006
10.1016/j.ijplas.2020.102670
10.1016/S1044-5803(01)00112-7
10.1016/j.actamat.2019.08.020
10.1016/j.proeng.2014.12.663
10.1016/j.ijfatigue.2013.03.004
10.1016/0921-5093(89)90414-0
10.1016/j.matdes.2008.06.007
10.1016/j.jmapro.2015.06.001
10.1016/j.euromechsol.2008.07.008
10.1016/S0257-8972(96)02959-3
10.1016/j.surfcoat.2006.02.028
10.1016/j.phpro.2016.08.020
10.1016/S0966-9795(00)00036-4
10.1016/0036-9748(83)90234-x
10.1016/j.jeurceramsoc.2011.10.025
10.1016/j.jallcom.2011.12.139
10.1016/j.ress.2017.03.006
10.1016/j.commatsci.2014.10.032
10.1016/j.actamat.2016.08.038
10.1016/S0966-9795(00)00012-1
10.1016/S1003-6326(08)60330-1
10.1016/j.matpr.2018.02.224
10.1016/j.ijplas.2019.11.001
10.1016/j.jmapro.2017.02.026

10.1016/j.promfg.2018.02.132
10.1016/S0924-0136(02)00656-8
10.1016/j.msea.2018.10.077
10.1016/j.jallcom.2018.03.049
10.1016/j.pnsc.2013.03.007
10.1016/j.matdes.2019.108418
10.1016/j.ijfatigue.2005.12.006
10.1016/j.matchar.2015.10.007
10.1016/0921-5093(93)90185-h
10.1016/j.msea.2012.08.050
10.1016/j.msea.2010.08.033
10.1016/j.scriptamat.2006.07.008
10.1016/j.actamat.2007.06.042
10.1016/j.apsusc.2012.01.069
10.1016/j.jallcom.2020.154158
10.1016/j.surfcoat.2011.10.060
10.1016/j.addma.2019.100844
10.1016/j.scriptamat.2007.08.014
10.1016/j.matdes.2012.07.013
10.1016/j.jallcom.2018.08.265
10.1016/j.jallcom.2018.05.131
10.1016/0026-0800(86)90011-x
10.1016/j.jmatprotec.2004.04.132
10.1016/0029-554x(82)90593-6
10.1016/j.matdes.2019.108082
10.1016/j.msea.2013.01.023
10.1016/j.jallcom.2015.10.120
10.1016/j.actamat.2017.02.003
10.1016/j.surfcoat.2008.11.016
10.1016/j.msea.2006.10.171
10.1016/j.jallcom.2019.152907
10.1016/j.actamat.2011.01.045
10.1016/j.actamat.2007.07.019
10.1016/0026-0800(86)90009-1
10.1016/j.jallcom.2018.01.224
10.1016/j.corsci.2007.03.040
10.1016/j.jmrt.2019.01.027
10.1016/j.matdes.2013.03.030
10.1016/j.jallcom.2019.06.170
10.1016/S1359-6454(96)00389-8
10.1016/j.matdes.2016.05.124
10.1016/S0022-3115(01)00520-7
10.1016/j.actamat.2006.09.044
10.1016/S0921-5093(03)00039-X
10.1016/j.ijthermalsci.2016.04.006
10.1016/0022-3115(86)90237-0
10.1016/j.actamat.2016.08.018
10.1016/j.jallcom.2003.09.117
10.1016/j.physb.2014.07.007
10.1016/j.jallcom.2019.151784
10.1016/j.wear.2007.01.028
10.1016/j.msea.2004.01.061
10.1016/j.jmapro.2019.01.019
10.1016/j.jmapro.2015.12.004

10.1016/j.msea.2010.10.044
10.1016/j.corsci.2013.05.025
10.1016/0925-8388(95)02082-9
10.1016/0022-3115(90)90352-n
10.1016/0921-5093(92)90344-z
10.1016/j.jallcom.2018.06.112
10.1016/j.commatsci.2010.04.008
10.1016/j.msea.2011.09.004
10.1016/S0921-5093(03)00051-0
10.1016/j.intermet.2019.02.002
10.1016/j.egypro.2017.11.206
10.1016/j.surfcoat.2006.07.251
10.1016/j.jmapro.2020.02.005
10.1016/j.matdes.2015.09.098
10.1016/j.jmatprotec.2017.05.013
10.1016/j.jallcom.2019.151916
10.1016/S0925-8388(01)01018-0
10.1016/j.jmapro.2019.01.030
10.1016/j.ijfatigue.2015.11.007
10.1016/j.actamat.2013.07.038
10.1016/j.actamat.2018.05.074
10.1016/S1000-9361(09)60229-6
10.1016/B978-1-85573-424-1.50022-8
10.1016/S0921-5093(01)01036-X
10.1016/j.msea.2019.138580
10.1016/j.prostr.2018.12.123
10.1016/j.matdes.2006.03.003
10.1016/j.actamat.2007.01.013
10.1016/j.matdes.2016.09.004
10.1016/j.jallcom.2019.02.235
10.1016/j.surfcoat.2003.12.016
10.1016/j.precisioneng.2016.11.007
10.1016/0169-4332(93)90329-a
10.1016/j.commatsci.2005.12.014
10.1016/S1359-6462(00)00355-9
10.1016/j.msea.2017.10.078
10.1016/j.jmst.2013.09.013
10.1016/j.intermet.2014.02.009
10.1016/j.msea.2012.12.077
10.1016/j.calphad.2017.12.001
10.1016/j.prostr.2020.01.139
10.1016/j.intermet.2013.04.012
10.1016/0025-5416(86)90362-9
10.1016/S0257-8972(01)01480-3
10.1016/0043-1648(85)90107-3
10.1016/j.corsci.2011.10.010
10.1016/S0925-8388(03)00454-7
10.1016/j.apsusc.2014.08.049
10.1016/j.jallcom.2008.01.025
10.1016/j.jallcom.2008.10.088
10.1016/j.msea.2018.02.014
10.1016/j.msea.2013.07.079
10.1016/j.jallcom.2016.05.028
10.1016/j.corsci.2020.108468

10.1016/0025-5416(70)90050-9
10.1016/j.ijleo.2017.04.066
10.1533/9780857095152.251
10.1016/j.commatsci.2018.02.013
10.1016/j.mechmat.2018.01.007
10.1016/j.jmatprotec.2005.04.021
10.1016/S1359-6454(03)00090-9
10.1016/j.promfg.2018.06.112
10.1016/j.jallcom.2016.04.185
10.1016/S1044-5803(03)00105-0
10.1016/j.msea.2014.12.060
10.1016/0025-5416(83)90089-7
10.1016/0142-1123(96)82760-5
10.1016/j.ijfatigue.2017.11.010
10.1016/0040-6090(79)90530-3
10.1016/S1350-6307(03)00037-2
10.1016/j.msea.2005.06.028
10.1016/j.ijfatigue.2016.03.012
10.1016/j.msea.2004.01.093
10.1016/j.jallcom.2007.06.110
10.1016/j.matchar.2006.09.004
10.1016/j.msea.2013.01.070
10.1016/0921-5093(93)90425-e
10.1016/j.matdes.2017.05.001
10.1016/j.ceramint.2014.08.051
10.1016/j.actamat.2018.03.017
10.1016/j.ijsolstr.2004.05.014
10.1016/j.scriptamat.2017.12.023
10.1016/j.corsci.2019.108115
10.1016/j.corsci.2012.03.027
10.1016/j.actamat.2018.05.055
10.1016/0304-3991(89)90255-6
10.1016/j.cja.2017.12.001
10.1016/j.msea.2018.06.095
10.1016/j.physleta.2004.09.034
10.1016/j.ijmachtools.2013.10.002
10.1016/0039-9140(69)80256-0
10.1016/j.matdes.2006.09.004
10.1016/j.jallcom.2018.05.263
10.1016/j.actamat.2010.06.027
10.1016/j.msea.2016.02.007
10.1016/S1359-6462(01)01075-2
10.1016/S0257-8972(02)00398-5
10.1016/j.msea.2020.139108
10.1016/j.msea.2018.02.029
10.1016/S0010-938X(96)00113-8
10.1016/j.jallcom.2016.11.306
10.1016/j.engfailanal.2014.08.007
10.1016/j.matlet.2014.08.036
10.1016/j.jmapro.2018.06.018
10.1016/j.matdes.2012.05.004
10.1016/j.apsusc.2014.11.101
10.1016/S1359-6454(00)00361-X
10.1016/S1003-6326(11)60813-3

10.1016/j.jallcom.2016.06.165
10.1016/s0036-9748(88)80275-8
10.1016/j.jmapro.2018.11.025
10.1016/0001-6160(85)90231-7
10.1016/j.optlastec.2013.09.030
10.1016/j.msea.2017.02.064
10.1016/j.msea.2010.03.099
10.1016/S0925-8388(03)00495-X
10.1016/0956-716X(95)00057-3
10.1016/S1003-6326(11)60814-5
10.1016/j.intermet.2015.10.019
10.1016/j.procir.2015.12.133
10.1016/S1359-6462(98)00451-5
10.1016/S1359-6462(02)00504-3
10.1016/j.scriptamat.2018.04.047
10.1016/j.msea.2006.10.170
10.1016/j.calphad.2018.05.003
10.1016/j.commatsci.2018.09.003
10.1016/0036-9748(78)90096-0
10.1016/j.jnucmat.2010.07.026
10.1016/j.cma.2016.04.018
10.1016/j.msea.2009.02.005
10.1016/j.matdes.2014.06.009
10.1016/j.jallcom.2017.12.254
10.1016/j.jmapro.2018.09.016
10.1016/j.intermet.2004.03.014
10.1016/j.apsusc.2016.03.181
10.1016/j.ijplas.2015.11.001
10.1016/j.corsci.2017.07.017
10.1016/j.matdes.2005.11.020
10.1016/j.jallcom.2018.01.142
10.1016/j.actamat.2013.01.031
10.1016/j.surfcoat.2017.10.052
10.1016/j.ijmachtools.2005.11.005
10.1016/j.msea.2015.12.003
10.1016/j.actamat.2008.10.058
10.1016/j.apsusc.2013.11.111
10.1016/j.jclepro.2016.07.090
10.1016/0956-7151(92)90195-k
10.1016/j.matchar.2017.11.045
10.1016/j.ijmachtools.2010.11.003
10.1016/j.intermet.2019.106604
10.1016/0921-5093(95)10073-3
10.1016/j.actamat.2017.09.027
10.1016/j.msea.2010.12.090
10.1016/0010-938x(90)90104-d
10.1016/j.msea.2017.08.101
10.1016/j.vacuum.2013.04.025
10.1016/j.prostr.2020.01.095
10.1016/j.corsci.2011.07.032
10.1016/j.corsci.2015.02.032
10.1016/0956-716X(95)90826-6
10.1016/j.corsci.2017.03.004
10.1016/j.actamat.2014.01.021

10.1016/S0966-9795(97)00029-0
10.1016/0036-9748(84)90267-9
10.1016/0921-5093(89)90755-7
10.1016/j.matchar.2008.02.004
10.1016/j.commatsci.2006.10.014
10.1016/S0022-3115(01)00468-8
10.1016/j.ijmecsci.2019.04.046
10.1016/j.jallcom.2016.12.314
10.1016/j.apsusc.2013.01.070
10.1016/j.msea.2009.02.049
10.1016/j.matlet.2013.07.060
10.1016/j.msea.2011.10.016
10.1016/j.jallcom.2019.153431
10.1016/j.apsusc.2014.02.056
10.1016/0956-7151(94)90061-2
10.1016/0169-4332(95)00387-8
10.1016/j.ceramint.2019.01.227
10.1016/j.matpr.2018.02.313
10.1016/j.msea.2006.05.104
10.1016/j.jallcom.2016.03.044
10.1016/0013-7944(86)90033-0
10.1016/j.jallcom.2014.12.135
10.1016/j.surfcoat.2015.12.083
10.1016/j.jmst.2013.07.008
10.1016/j.surfcoat.2018.01.011
10.1016/j.jallcom.2018.08.193
10.1016/j.jeurceramsoc.2018.09.013
10.1016/0020-7403(95)00063-1
10.1016/j.surfcoat.2018.10.073
10.1016/j.mprp.2018.02.001
10.1016/j.scriptamat.2010.07.019
10.1016/j.ijmachtools.2016.06.003
10.1016/S0026-0657(00)93009-1
10.1016/j.msea.2006.04.058
10.1016/j.matdes.2014.10.071
10.1016/S0022-0248(96)00850-0
10.1016/j.actamat.2017.07.029
10.1016/j.matdes.2019.107644
10.1016/j.corsci.2016.10.001
10.1016/S0022-3115(01)00514-1
10.1016/j.surfcoat.2018.07.020
10.1016/j.jallcom.2019.04.170
10.1016/j.ijfatigue.2017.04.017
10.1016/j.ijfatigue.2016.12.036
10.1016/j.jallcom.2015.04.130
10.1016/j.jallcom.2015.01.016
10.1016/0025-5416(87)90073-5
10.1016/j.msea.2019.138046
10.1016/j.jmapro.2020.02.006
10.1016/S0025-5408(03)00076-X
10.1016/j.msea.2005.08.188
10.1016/0022-3115(84)90681-0
10.1016/j.matlet.2018.02.128
10.1016/j.jallcom.2020.154889

10.1016/j.jelechem.2011.03.009
10.1016/j.prostr.2020.01.086
10.1016/j.msea.2014.09.025
10.1016/j.msea.2017.03.039
10.1016/j.pnsc.2013.02.005
10.1016/j.actamat.2009.03.046
10.1016/j.matlet.2015.08.032
10.1016/j.msea.2008.12.002
10.1016/j.optlastec.2016.12.032
10.1016/j.optlastec.2013.03.023
10.1016/j.matchar.2008.05.003
10.1016/j.matdes.2012.02.020
10.1016/S0965-9773(97)00107-4
10.1016/j.proeng.2010.03.088
10.1016/0261-3069(96)00025-8
10.1016/j.proeng.2011.12.545
10.1016/0040-6090(81)90008-0
10.1016/j.jestch.2020.02.001
10.1016/j.jallcom.2017.01.335
10.1016/j.ress.2015.10.002
10.1016/j.msea.2013.09.015
10.1016/j.jmatprotec.2017.03.021
10.1016/S1359-6454(03)00329-X
10.1016/j.jmst.2013.04.016
10.1016/j.msea.2003.08.111
10.1016/j.actamat.2013.10.008
10.1016/j.surfcoat.2003.10.046
10.1016/j.matchar.2014.07.016
10.1016/j.jallcom.2014.05.047
10.1016/j.matdes.2015.10.022
10.1016/j.corsci.2015.03.019
10.1016/j.tsf.2011.01.033
10.1016/j.msea.2013.08.034
10.1016/S0142-1123(98)00087-5
10.1016/j.jallcom.2011.08.097
10.1016/S1359-6462(01)01049-1
10.1016/j.msea.2018.03.078
10.1016/j.jmatprotec.2017.10.026
10.1016/j.actamat.2018.04.064
10.1016/j.jallcom.2018.03.364
10.1016/0364-5916(80)90030-9
10.1016/j.jmst.2017.10.014
10.1016/j.scriptamat.2012.05.015
10.1016/0261-3069(92)90046-k
10.1016/j.actamat.2015.12.014
10.1016/j.matchar.2010.02.016
10.1016/j.surfcoat.2014.03.034
10.1016/j.jmatprotec.2007.01.017
10.1016/j.ijfatigue.2008.07.009
10.1016/j.jmatprotec.2007.05.019
10.1016/0022-3115(85)90053-4
10.1016/j.pnsc.2016.01.006
10.1016/j.matlet.2019.127006
10.1016/j.susc.2008.06.017

10.1016/j.ijfatigue.2016.05.019
10.1016/S1359-6462(99)00133-5
10.1016/j.corsci.2010.12.023
10.1016/s1001-0521(08)60132-1
10.1016/j.actamat.2019.05.061
10.1016/j.intermet.2017.04.009
10.1016/S0921-5093(02)00733-5
10.1016/j.msea.2019.138241
10.1016/j.optlaseng.2011.12.007
10.1016/0921-5093(89)90793-4
10.1016/j.msea.2017.02.062
10.1016/j.ijfatigue.2012.04.016
10.1016/j.addma.2019.101031
10.1016/0026-0800(73)90048-7
10.1016/j.msea.2019.138437
10.1016/j.matchar.2009.09.016
10.1016/j.msea.2020.139177
10.1016/S0020-7403(97)00136-7
10.1016/j.measurement.2018.11.067
10.1016/j.corsci.2009.06.001
10.1016/j.msea.2011.03.115
10.1016/j.jmatprotec.2003.07.014
10.1016/S0142-1123(98)00027-9
10.1016/j.procir.2013.03.096
10.1016/j.ijfatigue.2011.12.010
10.1016/j.matdes.2018.11.051
10.1016/j.actamat.2016.02.051
10.1016/S1359-6462(97)00444-2
10.1016/j.apsusc.2018.08.111
10.1016/S0254-0584(03)00039-7
10.1016/S0924-0136(99)00243-5
10.1016/j.jallcom.2008.03.082
10.1016/j.sna.2018.06.032
10.1016/0036-9748(86)90458-8
10.1016/j.cossms.2014.06.001
10.1016/S1359-6454(96)00223-6
10.1016/0956-7151(92)90022-7
10.1016/j.jnoncrysol.2015.05.025
10.1016/j.proeng.2011.12.540
10.1016/j.optlaseng.2018.10.010
10.1016/j.jpowsour.2008.04.025
10.1016/j.jallcom.2017.11.352
10.1016/j.dib.2016.09.042
10.1016/S1359-6454(03)00171-X
10.1016/j.ijfatigue.2016.03.017
10.1016/j.surfcoat.2014.08.082
10.1016/j.engfracmech.2007.08.002
10.1016/j.corsci.2010.01.025
10.1016/j.msea.2006.12.112
10.1016/j.pnsc.2018.07.001
10.1016/j.surfcoat.2007.05.018
10.1016/S0927-0256(96)00065-1
10.1016/j.jallcom.2015.01.215
10.1016/0956-716x(93)90531-v

10.1016/0001-6160(87)90277-x
10.1016/j.engfracmech.2010.03.040
10.1016/j.jallcom.2008.01.101
10.1016/1359-6462(96)00223-0
10.1016/j.jallcom.2016.02.241
10.1016/j.ijplas.2017.12.007
10.1016/S0921-5093(97)00234-7
10.1016/j.surfcoat.2016.07.034
10.1016/j.phpro.2016.10.001
10.1016/j.msea.2014.07.055
10.1016/j.jallcom.2020.154137
10.1016/j.intermet.2019.106526
10.1016/j.surfcoat.2018.05.086
10.1016/0921-5093(89)90537-6
10.1016/j.apm.2013.04.005
10.1016/S1003-6326(08)60005-9
10.1016/j.intermet.2008.08.007
10.1016/j.msea.2007.07.030
10.1016/j.mprp.2016.04.001
10.1016/0026-0657(95)91368-8
10.1016/j.msea.2006.12.044
10.1016/0921-5093(94)09690-2
10.1016/j.ast.2018.09.011
10.1016/j.msea.2016.08.010
10.1016/j.actamat.2016.07.022
10.1016/j.msea.2012.04.042
10.1016/0029-5493(85)90208-0
10.1016/0308-0161(94)90034-5
10.1016/S0257-8972(01)01369-X
10.1016/j.addma.2019.100881
10.1016/j.jmst.2013.02.010
10.1016/j.scriptamat.2007.03.035
10.1016/j.matlet.2014.10.075
10.1016/j.matchar.2018.10.003
10.1016/j.msea.2018.07.104
10.1016/j.jmatprotec.2019.116461
10.1016/j.matdes.2016.12.005
10.1016/j.surfcoat.2006.12.020
10.1016/j.jclepro.2020.121275
10.1016/S0257-8972(00)00519-3
10.1016/j.scriptamat.2016.08.039
10.1016/j.engfailanal.2018.10.004
10.1016/j.msea.2019.05.065
10.1016/j.engfailanal.2004.10.002
10.1016/j.msea.2010.12.004
10.1016/S0257-8972(00)00870-7
10.1016/j.jallcom.2012.12.047
10.1016/j.jallcom.2014.08.154
10.1016/j.scriptamat.2011.08.014
10.1016/j.matpr.2017.12.022
10.1016/j.jmatprotec.2005.05.005
10.1016/j.msea.2008.12.047
10.1016/j.surfcoat.2010.09.039
10.1016/j.matchemphys.2010.08.070

10.1016/j.jallcom.2015.08.224
10.1016/S0921-5093(03)00498-2
10.1016/j.actamat.2014.12.011
10.1016/0956-716X(95)00320-U
10.1016/j.jmatprotec.2014.09.002
10.1016/j.surfcoat.2003.12.020
10.1016/j.msea.2019.138153
10.1016/j.scriptamat.2004.05.049
10.1016/j.cja.2019.11.006
10.1016/j.msea.2015.09.073
10.1016/j.surfcoat.2010.03.040
10.1016/j.electacta.2005.02.099
10.1016/j.actamat.2015.04.046
10.1016/j.msea.2007.01.152
10.1016/0921-5093(92)90107-c
10.1016/j.surfcoat.2004.03.030
10.1016/j.jallcom.2018.07.071
10.1016/0036-9748(87)90140-2
10.1016/j.msea.2013.08.060
10.1016/j.ijheatmasstransfer.2019.07.053
10.1016/j.matchemphys.2008.09.051
10.1016/j.matdes.2017.09.018
10.1016/S0924-0136(99)00123-5
10.1016/S0921-5093(97)00555-8
10.1016/j.jmapro.2017.12.023
10.1016/j.msea.2007.07.055
10.1016/0039-6028(93)90402-6
10.1016/j.jmrt.2020.03.044
10.1016/j.matdes.2017.05.032
10.1016/j.surfcoat.2011.09.002
10.1016/j.scriptamat.2011.11.043
10.1016/j.msea.2011.11.030
10.1016/j.msea.2014.06.068
10.1016/j.actamat.2009.04.039
10.1016/j.matdes.2016.02.052
10.1016/j.matchar.2017.08.002
10.1016/j.ijplas.2017.04.005
10.1016/j.matdes.2019.107766
10.1016/j.surfcoat.2013.10.041
10.1016/j.jallcom.2018.08.010
10.1016/j.msea.2016.04.004
10.1016/j.jmst.2014.06.010
10.1016/j.optlastec.2017.07.018
10.1016/j.matdes.2010.11.067
10.1016/j.jmatprotec.2018.03.009
10.1016/j.actamat.2015.03.052
10.1016/j.msea.2018.04.010
10.1016/j.rinp.2019.102340
10.1016/S1003-6326(14)63218-0
10.1016/j.matchar.2019.02.038
10.1016/j.msea.2013.05.011
10.1016/j.msea.2013.07.089
10.1016/j.ijfatigue.2018.12.015
10.1016/j.msea.2011.07.075

10.1016/j.msea.2020.138997
10.1016/j.surfcoat.2016.12.105
10.1016/S0921-5093(00)01049-2
10.1016/0001-6160(86)90106-9
10.1016/j.scriptamat.2005.03.047
10.1016/S0921-5093(02)00205-8
10.1016/j.corsci.2016.12.009
10.1016/S1003-6326(16)64235-8
10.1016/j.matlet.2017.08.122
10.1016/j.ijfatigue.2006.03.001
10.1016/j.surfcoat.2019.125066
10.1016/0022-3115(85)90370-8
10.1016/j.msea.2017.01.064
10.1016/j.ijfatigue.2019.105390
10.1016/j.surfcoat.2016.11.028
10.1016/j.msea.2010.03.075
10.1016/S1003-6326(11)61366-6
10.1016/j.msea.2013.02.003
10.1016/j.msea.2010.12.037
10.1016/j.msea.2010.08.069
10.1016/j.msea.2013.09.049
10.1016/S1003-6326(15)63663-9
10.1016/j.corsci.2018.11.030
10.1016/j.corsci.2010.05.009
10.1016/S0749-6419(00)00058-9
10.1016/j.jpowsour.2017.03.059
10.1016/j.jcrysgro.2008.09.183
10.1016/j.ijfatigue.2007.01.055
10.1016/j.corsci.2015.11.037
10.1016/j.matdes.2015.08.041
10.1016/j.actamat.2015.05.050
10.1016/j.matlet.2014.12.122
10.1016/j.msea.2019.04.078
10.1016/j.corsci.2020.108494
10.1016/j.matlet.2006.06.002
10.1016/j.intermet.2017.03.001
10.1016/j.actamat.2016.04.006
10.1016/j.jmatprotec.2013.04.019
10.1016/j.corsci.2019.108179
10.1016/S1359-6462(99)00229-8
10.1016/S0257-8972(01)01363-9
10.1016/0025-5416(87)90370-3
10.1016/j.jallcom.2020.154440
10.1016/0001-6160(89)90076-x
10.1016/j.msea.2019.05.111
10.1016/j.corsci.2018.03.008
10.1016/S0921-5093(97)00332-8
10.1016/0036-9748(82)90368-4
10.1016/j.corsci.2012.04.009
10.1016/j.mtla.2019.100569
10.1016/j.jmst.2016.05.012
10.1016/j.msea.2009.04.060
10.1016/j.ijfatigue.2007.01.035
10.1016/j.msea.2015.10.023

10.1016/0026-0657(96)91233-3
10.1016/j.jmatprotec.2019.01.013
10.1016/0010-938x(93)90115-w
10.1016/j.jallcom.2017.08.287
10.1016/j.msea.2007.12.045
10.1016/j.jallcom.2012.05.120
10.1016/j.scriptamat.2012.09.032
10.1016/j.surfcoat.2011.03.067
10.1016/S0261-3069(99)00078-3
10.1016/0257-8972(87)90081-8
10.1016/j.intermet.2019.106670
10.1016/j.ijmachtools.2006.10.007
10.1016/j.jnucmat.2012.08.044
10.1016/j.commatsci.2016.11.036
10.1016/j.apsusc.2017.09.047
10.1016/j.jmst.2014.09.020
10.1016/S1005-0302(10)60059-X
10.1016/j.matlet.2007.04.022
10.1016/j.scriptamat.2007.09.049
10.1016/j.matdes.2017.05.065
10.1016/j.matdes.2012.12.073
10.1016/0013-7944(88)90095-1
10.1016/j.jart.2016.05.003
10.1016/j.matchar.2009.05.012
10.1016/j.engfailanal.2007.05.007
10.1016/j.surfcoat.2011.03.122
10.1016/j.surfcoat.2017.07.026
10.1016/j.msea.2019.138162
10.1016/s0013-7944(85)80043-6
10.1016/j.jmapro.2019.09.012
10.1016/j.ijfatigue.2005.07.022
10.1016/j.actamat.2016.10.055
10.1016/j.matpr.2017.10.116
10.1016/0013-7944(83)90126-1
10.1016/j.vacuum.2016.12.007
10.1016/j.vacuum.2018.05.041
10.1016/j.jallcom.2007.12.051
10.1016/S0167-2738(98)00243-4
10.1016/j.addma.2017.03.008
10.1016/j.msea.2020.138987
10.1016/j.ijmecsci.2018.04.055
10.1016/j.apsusc.2019.04.089
10.1016/j.msea.2011.04.083
10.1016/j.msea.2016.12.038
10.1016/j.scriptamat.2007.10.013
10.1016/j.ijfatigue.2011.01.008
10.1016/j.applthermaleng.2016.11.022
10.1016/j.surfcoat.2019.02.069
10.1016/0001-6160(89)90177-6
10.1016/j.jallcom.2018.03.335
10.1016/j.matdes.2016.11.014
10.1016/j.scriptamat.2005.05.006
10.1016/S0927-0256(00)00166-X
10.1016/j.actamat.2011.01.004

10.1016/j.matchar.2010.10.008
10.1016/s0010-938x(78)80028-6
10.1016/0036-9748(83)90347-2
10.1016/j.jmps.2017.04.008
10.1016/S1359-6462(97)00220-0
10.1016/j.matdes.2016.10.069
10.1016/S0749-6419(00)00064-4
10.1016/S1003-6326(11)60855-8
10.1016/j.msea.2006.11.045
10.1016/S0925-8388(03)00422-5
10.1016/S0308-0161(02)00090-X
10.1016/j.actamat.2020.02.059
10.1016/S0079-6425(99)00010-9
10.1016/j.apsusc.2007.01.029
10.1016/j.wear.2014.04.022
10.1016/S0921-5093(03)00517-3
10.1016/S0921-5093(01)00985-6
10.1016/j.actamat.2015.10.007
10.1016/j.proeng.2013.03.306
10.1016/j.msea.2005.08.219
10.1016/j.procir.2019.04.042
10.1016/j.msea.2010.11.003
10.1016/j.jmatprotec.2019.116308
10.1016/j.surfcoat.2012.07.091
10.1016/j.matdes.2006.04.014
10.1016/S0142-9612(99)00142-8
10.1016/S0927-0256(02)00219-7
10.1016/j.jmst.2014.07.021
10.1016/j.mser.2008.04.003
10.1016/j.scriptamat.2004.09.013
10.1016/j.jallcom.2019.03.136
10.1016/j.msea.2007.08.005
10.1016/S0142-1123(98)90224-9
10.1016/j.matdes.2005.03.018
10.1016/j.commatsci.2019.109314
10.1016/j.jallcom.2017.06.332
10.1016/j.msea.2016.11.024
10.1016/0001-6160(86)90168-9
10.1016/0025-5416(87)90085-1
10.1016/j.surfcoat.2008.06.118
10.1016/j.scriptamat.2017.04.029
10.1016/0956-716x(94)90298-4
10.1016/j.proeng.2017.08.186
10.1016/j.intermet.2020.106725
10.1016/s1468-6996(01)00058-4
10.1016/j.corsci.2015.04.002
10.1016/j.apsusc.2018.12.187
10.1016/j.jmatprotec.2008.03.008
10.1016/j.matlet.2004.01.038
10.1016/j.heliyon.2019.e01388
10.1016/S1003-6326(11)60889-3
10.1016/j.actamat.2017.02.053
10.1016/0956-716x(93)90242-k
10.1016/j.surfcoat.2013.02.049

10.1016/j.msea.2012.04.083
10.1016/j.msea.2018.03.074
10.1016/j.matchar.2018.09.020
10.1016/S1000-9361(11)60326-9
10.1016/j.msea.2017.03.069
10.1016/j.msea.2006.08.149
10.1016/j.corsci.2015.08.033
10.1016/j.actamat.2012.05.033
10.1016/0022-3115(84)90553-1
10.1016/j.msea.2007.05.079
10.1016/j.proeng.2013.03.218
10.1016/j.msea.2004.07.038
10.1016/S1359-6454(99)00217-7
10.1016/j.jallcom.2013.04.116
10.1016/0036-9748(85)90170-x
10.1016/j.euromechsol.2015.08.012
10.1016/j.surfcoat.2018.09.023
10.1016/j.matdes.2012.06.052
10.1016/j.ijmachtools.2008.03.001
10.1016/S0920-3796(97)00006-9
10.1016/j.msea.2018.11.008
10.1016/j.msea.2018.12.019
10.1016/S0167-577X(02)01382-4
10.1016/j.surfcoat.2003.10.135
10.1016/j.matchemphys.2008.05.006
10.1016/j.wear.2019.01.080
10.1016/j.commatsci.2019.05.015
10.1016/j.scriptamat.2018.05.041
10.1016/S1359-6462(99)00151-7
10.1016/S0966-9795(99)00168-5
10.1016/j.scriptamat.2018.06.033
10.1016/j.cja.2016.07.003
10.1016/j.actamat.2008.03.029
10.1016/j.triboint.2010.12.011
10.1016/j.cma.2017.10.027
10.1016/j.actamat.2005.04.041
10.1016/j.optlaseng.2018.08.004
10.1016/j.jallcom.2007.07.079
10.1016/j.commatsci.2007.12.015
10.1016/j.jcrysgro.2009.05.002
10.1016/S0167-6636(00)00071-5
10.1016/s1468-6996(01)00049-3
10.1016/j.jmatprotec.2014.08.001
10.1016/S1044-5803(99)00050-9
10.1016/j.jallcom.2018.01.347
10.1016/j.ijmachtools.2007.08.004
10.1016/S0921-5093(98)00545-0
10.1016/S0168-583X(96)00694-5
10.1016/j.addma.2018.12.019
10.1016/j.corsci.2018.12.036
10.1016/j.engfracmech.2016.04.010
10.1016/j.jallcom.2019.07.102
10.1016/S0257-8972(98)00465-4
10.1016/s1001-0521(08)60175-8

10.1016/j.surfcoat.2012.10.004
10.1016/j.intermet.2003.09.018
10.1016/j.jallcom.2016.01.250
10.1016/j.pnsc.2016.11.005
10.1016/j.intermet.2004.02.026
10.1016/j.actamat.2008.12.022
10.1016/j.engfailanal.2016.03.012
10.1016/j.proeng.2011.04.049
10.1016/j.apt.2019.07.014
10.1016/j.msea.2018.01.083
10.1016/j.surfcoat.2008.12.029
10.1016/S0142-1123(97)87135-6
10.1016/j.actamat.2017.09.003
10.1016/j.cma.2006.06.006
10.1016/S1002-0071(12)60008-2
10.1016/j.actamat.2019.02.022
10.1016/j.jallcom.2015.10.092
10.1016/S0257-8972(00)01102-6
10.1016/S1003-6326(18)64750-8
10.1016/j.surfcoat.2013.06.041
10.1016/S0749-6419(03)00059-7
10.1016/j.jallcom.2016.12.403
10.1016/j.msea.2011.01.013
10.1016/j.pnsc.2016.03.013
10.1016/j.solener.2019.09.030
10.1016/j.intermet.2012.04.002
10.1016/j.porgcoat.2007.09.039
10.1016/j.commatsci.2011.01.010
10.1016/0956-716x(94)90367-0
10.1016/0956-716x(94)90497-9
10.1016/j.surfcoat.2012.09.013
10.1016/j.msea.2006.08.092
10.1016/j.msea.2016.11.081
10.1016/S1006-706X(12)60023-5
10.1016/j.matchar.2020.110168
10.1016/S1359-6462(98)00348-0
10.1016/S0167-577X(96)00225-X
10.1016/j.corsci.2013.07.004
10.1016/j.surfcoat.2005.07.090
10.1016/S0013-7944(00)00130-2
10.1016/j.matchar.2017.08.012
10.1016/S0956-716X(99)80060-0
10.1016/S1003-6326(19)65150-2
10.1016/0036-9748(89)90289-5
10.1016/j.jmps.2019.04.016
10.1016/j.intermet.2005.01.011
10.1016/j.actamat.2015.08.053
10.1016/0921-5093(89)90750-8
10.1016/j.jmapro.2019.03.009
10.1016/j.jallcom.2019.152195
10.1016/j.jmatprotec.2017.10.040
10.1016/j.jclepro.2018.07.053
10.1016/j.surfcoat.2008.08.053
10.1016/j.scriptamat.2014.01.031

10.1016/0142-1123(95)00014-K
10.1016/j.apsusc.2010.05.040
10.1016/j.matlet.2005.12.117
10.1016/j.matlet.2017.03.123
10.1016/0040-6090(80)90510-6
10.1016/j.matlet.2008.05.068
10.1016/S1359-6462(99)00191-8
10.1016/j.msea.2010.04.062
10.1016/j.ijfatigue.2005.07.027
10.1016/S1359-6462(98)00129-8
10.1016/S0022-3115(97)00106-2
10.1016/j.surfcoat.2011.06.041
10.1016/S0921-5093(00)01789-5
10.1016/0267-6605(90)90029-u
10.1016/j.msea.2009.02.044
10.1016/S1359-6454(00)00156-7
10.1016/S0257-8972(02)00593-5
10.1016/j.rinp.2017.04.027
10.1016/S0921-5093(97)80011-1
10.1016/S0167-6636(98)00020-9
10.1016/j.optlastec.2014.12.008
10.1016/j.ultramic.2015.07.003
10.1016/j.procir.2017.12.019
10.1016/j.msea.2006.08.129
10.1016/j.surfcoat.2018.11.077
10.1016/j.jmst.2018.02.007
10.1016/j.calphad.2019.101636
10.1016/0001-6160(89)90105-3
10.1016/j.ijplas.2005.03.007
10.1016/S0925-8388(02)01077-0
10.1016/j.actamat.2020.02.012
10.1016/j.msea.2019.05.007
10.1016/0956-7151(91)90180-9
10.1016/j.surfcoat.2014.07.089
10.1016/j.procir.2012.04.064
10.1016/j.matdes.2015.05.029
10.1016/j.actamat.2011.06.041
10.1016/j.mtla.2020.100635
10.1016/j.jallcom.2018.06.308
10.1016/0036-9748(89)90475-4
10.1016/j.jallcom.2010.11.176
10.1016/j.apm.2019.07.023
10.1016/j.jmps.2017.07.018
10.1016/j.msea.2010.01.051
10.1016/j.jmatprotec.2016.07.011
10.1016/j.jallcom.2007.01.091
10.1016/j.corsci.2008.08.018
10.1016/j.compstruc.2006.08.052
10.1016/j.corsci.2020.108472
10.1016/j.prostr.2019.08.237
10.1016/0167-6636(92)90016-7
10.1016/j.phpro.2013.03.077
10.1016/S0040-6090(96)08911-0
10.1016/j.jpowsour.2006.02.028

10.1016/j.msea.2018.12.084
10.1016/j.wear.2018.08.002
10.1016/j.msea.2017.04.002
10.1016/S0041-624X(99)00199-7
10.1016/j.msea.2017.11.056
10.1016/j.apsusc.2019.02.150
10.1016/j.msea.2013.10.013
10.1016/j.msea.2006.10.162
10.1016/j.msea.2008.06.058
10.1016/j.ceramint.2018.11.227
10.1016/j.jallcom.2018.06.229
10.1016/S0921-5093(00)01318-6
10.1016/j.jallcom.2015.01.183
10.1016/0036-9748(82)90332-5
10.1016/j.jallcom.2018.11.103
10.1016/j.msea.2014.08.011
10.1016/j.ceramint.2018.01.192
10.1016/S1359-6454(96)00125-5
10.1016/j.engfailanal.2017.11.018
10.1016/j.jpcs.2019.04.030
10.1016/S1359-6454(02)00080-0
10.1016/j.jallcom.2007.12.015
10.1016/j.matpr.2017.06.357
10.1016/j.jallcom.2016.08.210
10.1016/j.surfcoat.2018.03.074
10.1016/j.ijfatigue.2007.01.045
10.1016/S0040-6090(98)01413-8
10.1016/j.jcrysgro.2019.05.027
10.1016/j.scriptamat.2011.04.023
10.1016/j.surfcoat.2017.07.059
10.1016/j.jallcom.2013.01.011
10.1016/0025-5416(78)90060-5
10.1016/j.corsci.2019.108294
10.1016/j.ijfatigue.2017.01.002
10.1016/0036-9748(83)90451-9
10.1016/j.msea.2005.02.056
10.1016/j.actamat.2019.02.017
10.1016/j.mechmat.2009.04.002
10.1016/0094-5765(78)90043-7
10.1016/0001-6160(86)90201-4
10.1016/S1566-1369(02)80071-1
10.1016/j.surfcoat.2014.04.064
10.1016/j.jallcom.2016.04.107
10.1016/j.matdes.2017.01.065
10.1016/j.optlastec.2016.04.009
10.1016/j.phpro.2014.08.170
10.1016/0036-9748(85)90274-1
10.1016/j.jallcom.2014.10.094
10.1016/j.intermet.2012.09.014
10.1016/j.matchemphys.2017.10.064
10.1016/S0920-3796(96)00701-6
10.1016/j.ijhydene.2007.07.058
10.1016/0956-716x(92)90059-n
10.1016/j.surfcoat.2006.06.026

10.1016/0025-5416(86)90108-4
10.1016/S1005-0302(12)60046-2
10.1016/j.tca.2019.01.026
10.1016/j.addma.2019.100977
10.1016/j.ijimpeng.2014.02.007
10.1016/S1566-1369(02)80085-1
10.1016/j.jallcom.2020.153923
10.1016/j.jclepro.2016.05.122
10.1016/j.apm.2019.02.033
10.1016/j.physb.2017.11.059
10.1016/S0257-8972(01)01544-4
10.1016/j.jmst.2014.06.004
10.1016/j.matpr.2018.03.004
10.1016/j.ijfatigue.2013.03.003
10.1016/j.jallcom.2019.152954
10.1016/j.jallcom.2018.01.029
10.1016/j.ceramint.2017.09.225
10.1016/j.intermet.2019.04.009
10.1016/j.msea.2006.11.136
10.1016/j.msea.2011.07.059
10.1016/j.msea.2015.03.120
10.1016/j.matchemphys.2019.122392
10.1016/j.msea.2014.10.039
10.1016/0921-5093(95)09819-4
10.1016/j.ijrmhm.2014.12.001
10.1016/j.matchar.2012.02.002
10.1016/S0257-8972(02)00590-X
10.1016/j.surfcoat.2005.08.091
10.1016/j.jmst.2015.11.010
10.1016/0956-716x(93)90482-8
10.1016/S0921-5093(97)00277-3
10.1016/j.matchar.2007.04.010
10.1016/j.jnucmat.2019.01.013
10.1016/j.ceramint.2020.02.138
10.1016/j.msea.2008.12.024
10.1016/j.msea.2013.12.046
10.1016/j.msea.2012.01.121
10.1016/j.msea.2011.11.092
10.1016/0142-1123(84)90019-7
10.1016/j.msea.2008.04.110
10.1016/j.corsci.2015.05.062
10.1016/j.matchar.2016.05.013
10.1016/j.ijfatigue.2018.06.035
10.1016/0925-8388(92)90675-y
10.1016/S1003-6326(08)60317-9
10.1016/j.ijplas.2020.102682
10.1016/j.matchar.2015.07.026
10.1016/0025-5416(87)90378-8
10.1016/j.ijplas.2018.06.014
10.1016/j.jmatprotec.2003.11.039
10.1016/j.matchar.2012.10.004
10.1016/j.matdes.2015.10.155
10.1016/S1359-6454(01)00314-7
10.1016/j.jeurceramsoc.2018.12.015

10.1016/S1359-6454(99)00145-7
10.1016/j.surfcoat.2011.12.010
10.1016/0010-938X(96)00176-X
10.1016/j.jmapro.2018.01.005
10.1016/j.msea.2013.11.062
10.1016/0022-3115(86)90206-0
10.1016/j.surfcoat.2015.02.014
10.1016/0013-7944(85)90065-7
10.1016/j.corsci.2018.02.020
10.1016/j.corsci.2019.01.007
10.1016/j.tca.2018.08.011
10.1016/S0921-5093(00)01378-2
10.1016/j.ceramint.2015.04.077
10.1016/j.jmps.2014.01.012
10.1016/j.surfcoat.2017.03.071
10.1016/0040-6090(77)90272-3
10.1016/j.jmatprotec.2015.12.012
10.1016/j.vacuum.2018.01.022
10.1016/j.msea.2008.04.135
10.1016/j.ceramint.2019.08.117
10.1016/j.ijfatigue.2018.06.014
10.1016/j.matchar.2018.06.020
10.1016/j.jmatprotec.2018.10.023
10.1016/j.jmatprotec.2017.05.041
10.1016/j.jmrt.2018.10.005
10.1016/j.net.2017.10.014
10.1016/s1006-7191(08)60074-5
10.1016/j.corsci.2019.03.022
10.1016/j.jallcom.2008.09.058
10.1016/j.jallcom.2014.08.259
10.1016/S0257-8972(02)00604-7
10.1016/j.msea.2015.01.010
10.1016/j.matpr.2019.06.206
10.1016/j.surfin.2018.05.007
10.1016/j.jnucmat.2018.03.053
10.1016/j.jsamd.2016.08.009
10.1016/S0921-5093(97)00093-2
10.1016/j.matpr.2015.05.060
10.1016/j.actamat.2012.11.041
10.1016/j.jmst.2019.08.044
10.1016/0036-9748(87)90256-0
10.1016/j.intermet.2004.06.009
10.1016/j.actamat.2019.07.011
10.1016/0029-5493(89)90114-3
10.1016/j.msea.2015.03.125
10.1016/j.matchar.2012.11.012
10.1016/j.ijmachtools.2005.02.003
10.1016/j.jallcom.2013.09.112
10.1016/j.apt.2018.05.001
10.1016/j.jmst.2019.03.023
10.1016/0956-716x(92)90104-m
10.1016/S1359-6462(96)00405-8
10.1016/j.wear.2019.203116
10.1016/j.apsusc.2012.02.056

10.1016/j.surfcoat.2011.06.044
10.1016/s1468-6996(03)00048-2
10.1016/j.corsci.2015.11.032
10.1016/0956-716X(95)00090-I
10.1016/S0013-7944(97)00006-4
10.1016/j.jallcom.2003.09.129
10.1016/j.msea.2017.12.058
10.1016/S0924-0136(00)00609-9
10.1016/S0921-5093(96)10473-1
10.1016/j.msea.2016.08.064
10.1016/j.solener.2018.06.094
10.1016/0040-6090(80)90508-8
10.1016/j.actamat.2015.11.003
10.1016/j.corsci.2011.02.033
10.1016/j.ijmachtools.2016.03.008
10.1016/0013-7944(85)90134-1
10.1016/j.ijfatigue.2007.01.042
10.1016/j.matchar.2019.109936
10.1016/j.jnucmat.2010.12.309
10.1016/j.corsci.2018.08.004
10.1016/j.matchar.2016.11.033
10.1016/j.surfcoat.2019.124933
10.1016/j.jallcom.2018.01.256
10.1016/j.triboint.2015.12.050
10.1016/0026-0800(84)90018-1
10.1016/j.msea.2011.03.019
10.1016/j.addma.2018.12.017
10.1016/0036-9748(82)90421-5
10.1016/0001-6160(82)90019-0
10.1016/j.msea.2008.03.016
10.1016/j.procir.2016.06.109
10.1016/j.triboint.2018.01.011
10.1016/j.jallcom.2008.04.091
10.1016/0956-7151(94)00352-I
10.1016/j.msea.2020.139023
10.1016/j.tsf.2007.07.131
10.1016/S0142-1123(97)82590-X
10.1016/j.cja.2019.09.003
10.1016/j.apsusc.2013.01.189
10.1016/j.msea.2016.02.013
10.1016/j.dt.2018.10.005
10.1016/j.addma.2018.09.023
10.1016/0956-716x(94)90030-2
10.1016/0169-4332(94)90340-9
10.1016/j.jallcom.2006.08.036
10.1016/j.procir.2018.08.220
10.1016/j.matlet.2016.05.188
10.1016/1359-6462(96)00065-6
10.1016/j.jmapro.2018.11.024
10.1016/0039-6028(92)91039-e
10.1016/j.ijplas.2017.11.005
10.1016/S0955-2219(96)00197-5
10.1016/S1359-6462(97)00124-3
10.1016/j.matpr.2017.07.119

10.1016/0025-5416(81)90040-9
10.1016/j.ndteint.2014.04.009
10.1016/S0924-0136(01)00489-7
10.1016/j.jallcom.2016.05.057
10.1016/j.commatsci.2017.10.022
10.1016/j.msea.2011.03.096
10.1016/j.actamat.2015.11.018
10.1016/j.ijplas.2019.102645
10.1016/S0257-8972(99)00340-0
10.1016/j.vacuum.2018.02.031
10.1016/j.intermet.2014.12.011
10.1016/j.ijmachtools.2008.12.009
10.1016/j.actamat.2014.11.020
10.1016/S0966-9795(00)00033-9
10.1016/j.actamat.2017.11.042
10.1016/j.jallcom.2019.151685
10.1016/j.surfcoat.2014.08.045
10.1016/j.engfailanal.2008.10.015
10.1016/j.corsci.2018.11.029
10.1016/j.ijplas.2003.12.005
10.1016/j.engfracmech.2016.08.018
10.1016/j.corsci.2015.10.030
10.1016/j.ceramint.2018.06.234
10.1016/S0921-5093(98)01176-9
10.1016/j.precisioneng.2018.08.017
10.1016/S1359-6454(98)00323-1
10.1016/0257-8972(92)90132-t
10.1016/j.matchar.2020.110160
10.1016/j.msea.2005.03.015
10.1016/S0013-7944(98)00031-9
10.1016/S0921-5093(01)01208-4
10.1016/j.ijmachtools.2007.04.007
10.1016/j.physb.2019.411888
10.1016/j.bsecv.2018.10.003
10.1016/j.mechmat.2019.103068
10.1016/j.ijmecsci.2011.03.004
10.1016/j.jallcom.2019.05.030
10.1016/j.msea.2018.11.027
10.1016/j.ijfatigue.2007.01.048
10.1016/j.intermet.2015.04.011
10.1016/j.jmst.2013.11.006
10.1016/j.rinp.2018.05.034
10.1016/j.commatsci.2020.109531
10.1016/j.surfcoat.2013.09.034
10.1016/j.msea.2017.05.064
10.1016/j.msea.2007.10.004
10.1016/j.commatsci.2012.10.002
10.1016/j.corsci.2013.11.034
10.1016/j.actamat.2007.06.002
10.1016/j.matchar.2017.01.020
10.1016/j.actamat.2016.08.023
10.1016/0022-5088(88)90409-2
10.1016/j.actamat.2009.05.019
10.1016/j.actamat.2004.12.007

10.1016/j.surfcoat.2009.09.071
10.1016/j.msea.2015.05.089
10.1016/j.mechmat.2019.103218
10.1016/j.msea.2007.05.056
10.1016/j.matdes.2015.03.001
10.1016/j.msea.2013.02.027
10.1016/j.matdes.2014.11.017
10.1016/j.matlet.2007.09.028
10.1016/j.actamat.2018.08.052
10.1016/j.ijplas.2012.01.010
10.1016/S1359-6454(96)00185-1
10.1016/j.triboint.2014.11.023
10.1016/j.jallcom.2003.08.078
10.1016/j.ijfatigue.2020.105486
10.1016/j.ijfatigue.2017.08.006
10.1016/j.scriptamat.2018.12.025
10.1016/j.actamat.2019.03.016
10.1016/j.msea.2007.05.077
10.1016/j.matdes.2015.07.063
10.1016/j.msea.2011.11.089
10.1016/j.msea.2014.04.054
10.1016/0956-716x(94)90511-8
10.1016/j.jct.2015.01.010
10.1016/j.jallcom.2017.01.091
10.1016/j.ndteint.2017.02.008
10.1016/S1003-6326(18)64867-8
10.1016/j.msea.2016.02.038
10.1016/j.prostr.2016.06.115
10.1016/j.optlaseng.2012.08.006
10.1016/0956-716x(93)90262-q
10.1016/j.scriptamat.2012.06.014
10.1016/j.surfcoat.2009.11.036
10.1016/1044-5803(91)90012-s
10.1016/j.actamat.2020.01.033
10.1016/j.msea.2018.01.007
10.1016/j.jallcom.2017.02.009
10.1016/j.isatra.2014.09.003
10.1016/0584-8547(81)80124-3
10.1016/0956-7151(94)90470-7
10.1016/j.corsci.2017.08.015
10.1016/j.mechmat.2009.10.001
10.1016/j.msea.2018.12.043
10.1016/j.actamat.2013.06.001
10.1016/j.ijmachtools.2004.02.016
10.1016/j.msea.2012.06.027
10.1016/j.matchar.2015.06.017
10.1016/j.msea.2015.09.023
10.1016/j.ijfatigue.2007.04.014
10.1016/j.msea.2019.06.013
10.1016/S0921-5093(98)00977-0
10.1016/j.msea.2005.02.047
10.1016/S0921-5093(02)00063-1
10.1016/S0257-8972(01)01243-9
10.1016/0022-3115(87)90505-8

10.1016/j.corsci.2015.01.050
10.1016/S0921-5093(97)00366-3
10.1016/S1359-6462(98)00456-4
10.1016/j.msea.2013.04.021
10.1016/j.msea.2015.09.070
10.1016/S0921-5093(01)01362-4
10.1016/j.actamat.2018.02.055
10.1016/j.jallcom.2017.06.302
10.1016/j.msea.2012.11.024
10.1016/j.jallcom.2003.10.089
10.1016/j.matlet.2013.10.115
10.1016/S1002-0721(12)60360-3
10.1016/j.matchar.2015.12.027
10.1016/j.jallcom.2017.11.299
10.1016/S1359-6454(01)00283-X
10.1016/j.msea.2019.01.097
10.1016/j.msea.2017.05.082
10.1016/S0924-0136(02)00302-3
10.1016/j.ijmecsci.2018.04.018
10.1016/j.jallcom.2016.07.051
10.1016/j.marstruc.2017.02.001
10.1016/j.jmst.2018.09.040
10.1016/j.optlastec.2018.09.053
10.1016/S1359-6454(99)00018-X
10.1016/j.msea.2014.01.095
10.1016/j.surfcoat.2004.02.013
10.1016/j.engfailanal.2020.104388
10.1016/0956-716x(95)00548-a
10.1016/j.matdes.2010.06.034
10.1016/j.msea.2009.01.061
10.1016/j.ultramic.2012.03.013
10.1016/j.jallcom.2009.07.141
10.1016/j.jallcom.2020.154108
10.1016/j.jallcom.2019.152863
10.1016/B978-1-85573-424-1.50015-0
10.1016/j.ceramint.2017.05.077
10.1016/S0254-0584(98)00022-4
10.1016/j.matlet.2015.06.076
10.1016/j.matchar.2005.11.007
10.1016/0167-577X(94)00270-3
10.1016/j.surfcoat.2010.08.064
10.1016/j.jallcom.2007.12.004
10.1016/j.surfcoat.2017.09.086
10.1016/j.actamat.2012.02.021
10.1016/j.actamat.2012.06.058
10.1016/s0257-8972(05)80014-3
10.1016/j.jmatprotec.2019.116474
10.1016/j.actamat.2011.06.050
10.1016/j.jallcom.2017.01.278
10.1016/j.matlet.2019.126730
10.1016/0142-1123(95)99771-2
10.1016/j.cja.2019.01.026
10.1016/j.surfcoat.2005.10.036
10.1016/j.msea.2006.04.049

10.1016/j.jallcom.2018.12.320
10.1016/j.actamat.2014.12.046
10.1016/j.msea.2009.04.026
10.1016/j.msea.2004.01.114
10.1016/S0921-5093(98)00482-1
10.1016/j.actamat.2010.09.027
10.1016/j.msea.2016.11.013
10.1016/j.matdes.2019.107983
10.1016/j.addma.2019.100998
10.1016/j.optlastec.2013.05.011
10.1016/j.stam.2006.12.001
10.1016/0257-8972(89)90037-6
10.1016/S0142-1123(02)00010-5
10.1016/S0010-938X(00)00149-9
10.1016/S1006-706X(17)30112-7
10.1016/S0927-0256(02)00257-4
10.1016/j.ijmecsci.2019.01.014
10.1016/j.actamat.2020.01.022
10.1016/j.rinp.2019.102908
10.1016/j.actamat.2014.08.047
10.1016/j.matlet.2019.127177
10.1016/j.actamat.2019.11.003
10.1016/S0921-5093(02)00173-9
10.1016/S1359-6462(02)00072-6
10.1016/j.jallcom.2013.02.100
10.1016/j.procir.2018.08.291
10.1016/j.corsci.2017.04.026
10.1016/j.jmps.2017.02.010
10.1016/S1005-0302(11)60160-6
10.1016/S0026-0657(05)00306-1
10.1016/j.msea.2019.138461
10.1016/j.jallcom.2013.10.036
10.1016/B978-008044504-5/50037-4
10.1016/j.jnucmat.2010.11.051
10.1016/j.msea.2007.01.088
10.1016/S1044-5803(01)00106-1
10.1016/j.msea.2011.09.098
10.1016/j.jmst.2017.04.012
10.1016/0168-583x(86)90026-1
10.1016/j.surfcoat.2015.03.036
10.1016/S0924-0136(03)00285-1
10.1016/0257-8972(95)02528-6
10.1016/j.msea.2006.07.016
10.1016/j.msea.2014.07.006
10.1016/S1359-6462(98)00184-5
10.1016/S0749-6419(00)00045-0
10.1016/j.matdes.2016.07.068
10.1016/j.corsci.2012.11.041
10.1016/S1359-6462(00)00473-5
10.1016/0956-716X(95)00229-0
10.1016/j.engfracmech.2008.01.009
10.1016/j.actamat.2006.06.044
10.1016/j.ceramint.2017.05.338
10.1016/j.jallcom.2014.11.091

10.1016/j.msea.2015.03.045
10.1016/j.ijfatigue.2015.11.020
10.1016/j.jmatprotec.2004.01.049
10.1016/j.msea.2008.07.046
10.1016/S0955-2219(98)00239-8
10.1016/0921-5093(95)10052-0
10.1016/j.matpr.2019.01.013
10.1016/S0921-5093(02)00681-0
10.1016/S1359-6462(02)00363-9
10.1016/j.electacta.2015.06.045
10.1016/j.msea.2019.138098
10.1016/j.msea.2019.01.101
10.1016/0025-5416(87)90093-0
10.1016/j.msea.2018.01.074
10.1016/0026-0657(95)91369-6
10.1016/j.matdes.2015.11.041
10.1016/j.msea.2015.11.058
10.1016/S0921-5093(01)00925-X
10.1016/j.jfluchem.2017.03.006
10.1016/0921-5093(95)10036-9
10.1016/j.matpr.2019.07.423
10.1016/j.surfcoat.2016.09.087
10.1016/j.procir.2015.03.053
10.1016/j.engfailanal.2004.12.027
10.1016/0010-938X(96)00183-7
10.1016/j.addma.2019.01.012
10.1016/j.jcrysgro.2014.05.016
10.1016/j.jmatprotec.2018.06.039
10.1016/j.ijfatigue.2013.11.001
10.1016/j.msea.2011.01.023
10.1016/S1359-6454(02)00042-3
10.1016/j.actamat.2018.09.013
10.1016/j.cirp.2010.03.093
10.1016/j.msea.2017.09.073
10.1016/j.msea.2019.03.013
10.1016/S0168-583X(02)01699-3
10.1016/S0921-5093(02)00594-4
10.1016/0025-5416(86)90092-3
10.1016/j.optlaseng.2016.08.005
10.1016/j.actamat.2005.12.019
10.1016/0036-9748(75)90405-6
10.1016/j.matlet.2015.03.026
10.1016/j.jmatprotec.2017.12.016
10.1016/j.jmrt.2018.05.012
10.1016/j.matchar.2017.02.006
10.1016/j.triboint.2007.04.006
10.1016/j.msea.2012.11.079
10.1016/j.actamat.2016.03.020
10.1016/j.msea.2019.06.016
10.1016/j.msea.2015.12.096
10.1016/j.matdes.2019.107915
10.1016/S0749-6419(96)00044-7
10.1016/j.intermet.2014.01.020
10.1016/j.actamat.2019.03.010

10.1016/j.msea.2016.11.063
10.1016/0026-0800(70)90045-5
10.1016/S0045-7949(99)00229-1
10.1016/j.ceramint.2015.11.008
10.1016/0001-6160(89)90286-1
10.1016/j.msea.2017.11.043
10.1016/j.surfcoat.2018.08.005
10.1016/j.wear.2009.07.002
10.1016/j.matdes.2013.01.022
10.1016/j.msea.2019.138137
10.1016/s1005-8850(08)60111-5
10.1016/j.scriptamat.2015.09.018
10.1016/j.jallcom.2013.03.241
10.1016/j.msea.2016.10.031
10.1016/j.scriptamat.2004.03.040
10.1016/j.calphad.2014.12.004
10.1016/j.wear.2009.03.021
10.1016/j.msea.2018.10.041
10.1016/S1359-6462(02)00041-6
10.1016/j.vacuum.2018.12.043
10.1016/j.msea.2016.05.089
10.1016/j.actamat.2014.12.029
10.1016/j.corsci.2012.09.046
10.1016/j.proeng.2010.03.113
10.1016/j.matchar.2017.09.016
10.1016/j.engfailanal.2016.02.016
10.1016/j.jallcom.2019.04.139
10.1016/S0921-5093(97)00698-9
10.1016/j.electacta.2008.07.056
10.1016/0036-9748(75)90291-4
10.1016/S0257-8972(03)00879-X
10.1016/j.jmst.2019.08.008
10.1016/j.jeurceramsoc.2018.10.019
10.1016/S0026-0657(96)94153-3
10.1016/j.msea.2004.07.013
10.1016/j.msea.2009.10.028
10.1016/j.msea.2017.12.025
10.1016/j.surfcoat.2007.06.041
10.1016/S0921-5093(03)00261-2
10.1016/S0924-0136(01)00646-X
10.1016/j.corsci.2018.11.021
10.1016/j.msea.2005.02.068
10.1016/0749-6419(86)90013-6
10.1016/j.jclepro.2015.05.039
10.1016/j.apsusc.2014.03.160
10.1016/j.jmst.2014.07.008
10.1016/j.jallcom.2014.11.056
10.1016/S1003-6326(19)65074-0
10.1016/S1006-706X(13)60127-2
10.1016/j.ijrmhm.2013.02.010
10.1016/j.precisioneng.2015.01.002
10.1016/0956-716x(90)90041-e
10.1016/S0921-5093(97)00761-2
10.1016/j.intermet.2017.03.013

10.1016/j.ijfatigue.2011.10.018
10.1016/j.scriptamat.2005.11.010
10.1016/0040-6090(88)90319-7
10.1016/j.jallcom.2012.02.136
10.1016/j.surfcoat.2004.07.091
10.1016/j.scriptamat.2016.08.029
10.1016/0013-7944(89)90279-8
10.1016/j.jmatprotec.2012.09.002
10.1016/j.commatsci.2018.03.015
10.1016/j.matchemphys.2011.10.045
10.1016/j.ijmecsci.2018.09.046
10.1016/j.jallcom.2015.05.251
10.1016/j.matchar.2018.08.006
10.1016/j.matpr.2019.12.228
10.1016/0956-7151(90)90223-4
10.1016/0257-8972(95)02640-1
10.1016/j.ijmecsci.2019.06.027
10.1016/j.jeurceramsoc.2014.08.029
10.1016/j.matdes.2019.107711
10.1016/0956-716x(94)90340-9
10.1016/j.actamat.2018.04.034
10.1016/j.measurement.2019.01.065
10.1016/j.intermet.2014.01.011
10.1016/j.msea.2019.138893
10.1016/j.promfg.2016.08.050
10.1016/j.msea.2019.03.124
10.1016/0956-716x(95)00341-r
10.1016/S0257-8972(97)00487-8
10.1016/S0921-5093(01)00944-3
10.1016/j.pnsc.2019.01.013
10.1016/j.electacta.2018.04.186
10.1016/j.surfcoat.2018.01.073
10.1016/j.jallcom.2014.07.099
10.1016/j.intermet.2013.12.006
10.1016/B978-0-444-63303-3.00011-0
10.1016/j.actamat.2008.03.016
10.1016/j.jallcom.2016.06.289
10.1016/0966-9795(95)00056-9
10.1016/j.jnucmat.2013.08.009
10.1016/j.intermet.2012.01.008
10.1016/j.engfracmech.2013.01.001
10.1016/j.ceramint.2012.12.005
10.1016/j.scriptamat.2018.12.023
10.1016/j.jallcom.2018.03.372
10.1016/j.jnucmat.2017.10.002
10.1016/j.rinp.2019.102593
10.1016/S0966-9795(00)00034-0
10.1016/s1001-0521(08)60118-7
10.1016/j.msea.2006.11.082
10.1016/j.wear.2014.12.008
10.1016/S0142-1123(03)00064-1
10.1016/j.ssc.2015.03.010
10.1016/j.engfailanal.2012.05.028
10.1016/j.commatsci.2004.03.003

10.1016/0168-583X(95)00499-8
10.1016/j.jallcom.2013.07.151
10.1016/S0921-5093(03)00345-9
10.1016/j.msea.2018.02.005
10.1016/j.micron.2010.03.003
10.1016/j.matdes.2014.06.064
10.1016/j.msea.2009.10.001
10.1016/j.procir.2012.05.005
10.1016/j.ssi.2004.07.040
10.1016/0257-8972(95)08341-3
10.1016/j.msea.2016.09.064
10.1016/S1003-6326(17)60082-7
10.1016/j.msea.2003.12.092
10.1016/j.scriptamat.2017.08.021
10.1016/j.jmatprotec.2012.06.003
10.1016/j.corsci.2019.04.020
10.1016/S0927-0256(99)00067-1
10.1016/j.msea.2004.02.100
10.1016/j.engfailanal.2011.03.010
10.1016/j.matpr.2015.07.109
10.1016/j.ijfatigue.2009.08.004
10.1016/j.engfailanal.2017.08.011
10.1016/j.scriptamat.2006.02.032
10.1016/0025-5416(87)90423-x
10.1016/j.matchar.2018.10.008
10.1016/j.commatsci.2018.11.024
10.1016/j.jallcom.2014.07.084
10.1016/0036-9748(89)90270-6
10.1016/j.jmatprotec.2018.12.002
10.1016/j.engfailanal.2019.04.021
10.1016/j.scriptamat.2008.04.012
10.1016/S1359-6462(97)00061-4
10.1016/j.msea.2015.06.021
10.1016/j.msea.2017.03.063
10.1016/j.surfcoat.2019.125202
10.1016/j.actamat.2009.04.033
10.1016/j.msea.2019.02.075
10.1016/j.msea.2009.03.071
10.1016/j.jallcom.2020.153983
10.1016/j.jallcom.2019.05.094
10.1016/j.actamat.2013.10.021
10.1016/S1359-6462(01)00927-7
10.1016/j.mechmat.2017.03.020
10.1016/j.corsci.2019.108150
10.1016/S1003-6326(16)64200-0
10.1016/0167-2584(86)90948-5
10.1016/S0734-743X(01)00004-5
10.1016/j.msea.2009.08.023
10.1016/j.cirp.2014.03.067
10.1016/j.engfracmech.2007.01.015
10.1016/S1359-6462(02)00595-X
10.1016/S0921-5093(02)00637-8
10.1016/j.matpr.2018.02.111
10.1016/0039-9140(94)e0037-r

10.1016/j.actamat.2010.03.017
10.1016/0956-716x(94)90130-9
10.1016/0921-5093(89)90815-0
10.1016/j.vacuum.2018.02.027
10.1016/j.actamat.2007.11.014
10.1016/j.matchar.2013.01.008
10.1016/S0924-0136(97)00432-9
10.1016/j.matchar.2004.03.007
10.1016/j.solidstatesciences.2012.11.011
10.1016/S0921-5093(01)01785-3
10.1016/j.jnucmat.2014.07.005
10.1016/0956-716x(93)90578-g
10.1016/j.optlaseng.2018.04.022
10.1016/0025-5416(87)90229-1
10.1016/j.msea.2009.01.033
10.1016/S0921-5093(97)00003-8
10.1016/j.matdes.2014.09.009
10.1016/j.jallcom.2019.01.389
10.1016/j.actamat.2008.05.041
10.1016/0257-8972(89)90084-4
10.1016/j.euromechsol.2008.10.002
10.1016/j.matlet.2013.07.073
10.1016/j.commatsci.2014.03.023
10.1016/j.corsci.2019.108169
10.1016/S0921-5093(99)00705-4
10.1016/0029-5493(92)90063-2
10.1016/j.scriptamat.2009.02.012
10.1016/j.msea.2019.138431
10.1016/j.ijrmhm.2018.07.007
10.1016/S0022-0248(01)01438-5
10.1016/0364-5916(81)90006-7
10.1016/j.surfcoat.2017.09.040
10.1016/j.electacta.2017.09.046
10.1016/j.engfailanal.2010.05.004
10.1016/0025-5416(88)90792-6
10.1016/j.jmsy.2017.01.004
10.1016/S1359-6454(02)00396-8
10.1016/j.msea.2007.05.069
10.1016/S0966-9795(99)00142-9
10.1016/j.actamat.2006.08.024
10.1016/0040-6090(77)90130-4
10.1016/j.ijheatmasstransfer.2019.118957
10.1016/0036-9748(89)90085-9
10.1016/0025-5416(80)90203-7
10.1016/j.ijfatigue.2005.01.014
10.1016/j.engfailanal.2014.07.021
10.1016/j.jallcom.2010.02.023
10.1016/j.jcrysgro.2013.11.092
10.1016/j.msea.2013.02.007
10.1016/1044-5803(90)90011-8
10.1016/j.jallcom.2019.153559
10.1016/S1359-6462(98)00178-X
10.1016/1359-6454(95)00354-1
10.1016/j.surfcoat.2006.06.001

10.1016/j.pmatsci.2018.10.004
10.1016/j.matpr.2017.01.008
10.1016/j.corsci.2003.10.024
10.1016/j.jmatprotec.2015.12.004
10.1016/j.jmatprotec.2016.02.010
10.1016/j.ceramint.2019.04.215
10.1016/j.actamat.2018.05.043
10.1016/j.actamat.2015.03.060
10.1016/0010-938x(90)90134-q
10.1016/j.actamat.2018.07.033
10.1016/0022-3115(84)90544-0
10.1016/j.msea.2010.06.023
10.1016/j.wear.2020.203191
10.1016/j.cirpj.2011.07.003
10.1016/S1003-6326(16)64274-7
10.1016/j.euromechsol.2013.06.010
10.1016/j.jallcom.2018.08.063
10.1016/0167-8442(91)90040-q
10.1016/S1359-6454(98)00022-6
10.1016/j.proeng.2017.10.1108
10.1016/j.msea.2010.11.038
10.1016/j.msea.2015.08.038
10.1016/S0257-8972(96)03127-1
10.1016/j.engfracmech.2010.02.002
10.1016/j.matdes.2013.10.085
10.1016/j.procir.2014.04.062
10.1016/j.engfailanal.2012.10.012
10.1016/0025-5416(87)90257-6
10.1016/j.surfcoat.2011.06.037
10.1016/j.msea.2011.03.044
10.1016/S0921-5093(97)00161-5
10.1016/j.matdes.2017.07.065
10.1016/j.corsci.2016.02.004
10.1016/S0921-5093(02)00169-7
10.1016/j.corsci.2018.07.032
10.1016/j.cap.2003.11.033
10.1016/S0257-8972(96)02921-0
10.1016/j.corsci.2018.01.036
10.1016/j.msea.2018.02.091
10.1016/j.optlastec.2015.12.020
10.1016/j.surfcoat.2018.08.061
10.1016/0036-9748(81)90331-8
10.1016/j.surfcoat.2015.10.071
10.1016/0001-6160(89)90054-0
10.1016/j.surfcoat.2016.05.005
10.1016/j.surfcoat.2005.09.037
10.1016/j.scriptamat.2020.02.038
10.1016/j.triboint.2019.04.042
10.1016/j.jmatprotec.2017.09.010
10.1016/j.fuel.2012.11.081
10.1016/j.ijmecsci.2019.105357
10.1016/j.jmatprotec.2004.04.416
10.1016/j.matdes.2015.09.041
10.1016/j.matdes.2015.12.152

10.1016/j.nucengdes.2013.11.054
10.1016/j.surfcoat.2013.09.022
10.1016/j.scriptamat.2015.11.026
10.1016/0257-8972(96)02856-3
10.1016/j.jallcom.2018.05.169
10.1016/j.matchar.2017.02.021
10.1016/j.msea.2019.04.080
10.1016/j.matdes.2014.12.030
10.1016/j.scriptamat.2003.09.043
10.1016/j.surfcoat.2005.02.172
10.1016/j.ijfatigue.2009.02.042
10.1016/j.msea.2013.06.004
10.1016/j.jallcom.2017.02.262
10.1016/j.matchemphys.2015.09.043
10.1016/j.matchar.2015.06.018
10.1016/j.surfcoat.2009.09.073
10.1016/j.engfailanal.2008.10.010
10.1016/j.corsci.2019.108186
10.1016/0167-577x(94)90026-4
10.1016/j.jmatprotec.2017.01.018
10.1016/j.msea.2010.10.015
10.1016/S0020-7403(02)00138-8
10.1016/j.actamat.2015.04.034
10.1016/j.msea.2006.02.457
10.1016/j.ijplas.2007.10.001
10.1016/j.addma.2017.02.003
10.1016/j.ijfatigue.2020.105575
10.1016/0025-5416(87)90288-6
10.1016/j.egypro.2013.07.106
10.1016/j.jmst.2015.11.007
10.1016/S1006-706X(15)30039-X
10.1016/j.vacuum.2016.11.012
10.1016/S1359-6454(02)00474-3
10.1016/j.msea.2020.139219
10.1016/0036-9748(82)90461-6
10.1016/j.jeurceramsoc.2011.05.047
10.1016/j.msea.2014.10.003
10.1016/j.ijsolstr.2011.02.014
10.1016/j.surfcoat.2010.07.018
10.1016/j.msea.2010.11.053
10.1016/j.matchemphys.2010.11.030
10.1016/0921-5093(89)90350-x
10.1016/1359-6462(96)00207-2
10.1016/j.surfcoat.2009.08.038
10.1016/j.ceramint.2016.05.203
10.1016/j.ceramint.2019.09.037
10.1016/0025-5416(85)90339-8
10.1016/S1000-9361(11)60288-4
10.1016/j.matchar.2017.07.041
10.1016/S1359-6462(97)00035-3
10.1016/j.msea.2005.03.021
10.1016/1359-6462(95)00554-4
10.1016/j.calphad.2018.01.005
10.1016/S0924-0136(97)02939-7

10.1016/j.pnsc.2018.11.005
10.1016/j.matchar.2008.09.002
10.1016/j.calphad.2006.07.007
10.1016/S0921-5093(03)00258-2
10.1016/j.intermet.2018.08.005
10.1016/S0965-9773(97)00026-3
10.1016/j.scriptamat.2018.03.041
10.1016/j.ijfatigue.2017.08.015
10.1016/j.msea.2014.02.046
10.1016/j.surfcoat.2013.09.028
10.1016/j.matpr.2015.07.048
10.1016/j.vacuum.2018.12.013
10.1016/j.actamat.2018.03.057
10.1016/j.proeng.2010.03.245
10.1016/j.matchar.2017.03.041
10.1016/j.jallcom.2019.01.334
10.1016/j.ijfatigue.2013.01.015
10.1016/0010-938x(78)90058-6
10.1016/j.scriptamat.2005.08.033
10.1016/j.surfcoat.2017.03.003
10.1016/j.addma.2016.11.006
10.1016/0026-0800(89)90026-8
10.1016/0010-938x(93)90331-a
10.1016/0026-0800(70)90016-9
10.1016/j.matpr.2017.12.248
10.1016/j.corsci.2013.05.027
10.1016/0079-6425(85)90001-5
10.1016/S0167-577X(98)00028-7
10.1016/j.wear.2009.05.007
10.1016/0956-716X(95)00299-B
10.1016/j.jallcom.2011.03.146
10.1016/j.corsci.2019.108396
10.1016/j.intermet.2006.10.038
10.1016/j.euromechsol.2009.12.008
10.1016/S0925-8388(01)01567-5
10.1016/j.ijfatigue.2008.04.007
10.1016/j.jallcom.2014.05.154
10.1016/j.jnucmat.2007.05.002
10.1016/S0257-8972(98)00579-9
10.1016/0749-6419(93)90016-j
10.1016/0022-3115(79)90436-7
10.1016/j.jcrysgro.2003.12.057
10.1016/j.ijfatigue.2011.08.017
10.1016/j.ijplas.2019.04.018
10.1016/j.scriptamat.2018.07.034
10.1016/j.ijfatigue.2015.07.020
10.1016/j.matdes.2011.11.007
10.1016/j.msea.2006.09.060
10.1016/j.jallcom.2019.07.056
10.1016/S1003-6326(11)61025-X
10.1016/0040-6090(81)90006-7
10.1016/j.matdes.2017.08.009
10.1016/0956-716x(93)90258-t
10.1016/0010-938x(91)90085-4

10.1016/1359-6462(96)00092-9
10.1016/j.commatsci.2014.08.045
10.1016/0956-716X(95)00038-W
10.1016/j.matpr.2018.06.308
10.1016/0921-5093(93)90345-f
10.1016/S1466-6049(99)00047-1
10.1016/j.ijfatigue.2010.02.002
10.1016/j.intermet.2006.09.009
10.1016/j.engfailanal.2013.03.018
10.1016/j.msea.2010.08.005
10.1016/j.proeng.2011.04.618
10.1016/j.intermet.2012.04.020
10.1016/j.ijplas.2017.03.010
10.1016/j.jallcom.2019.01.132
10.1016/j.msea.2008.09.107
10.1016/j.jallcom.2016.09.189
10.1016/j.jallcom.2016.01.033
10.1016/j.triboint.2016.04.016
10.1016/0013-7944(88)90080-x
10.1016/S0924-0136(97)00484-6
10.1016/j.surfcoat.2014.05.067
10.1016/0025-5416(82)90129-x
10.1016/j.msea.2011.07.072
10.1016/0025-5416(87)90089-9
10.1016/j.matchar.2014.07.013
10.1016/0257-8972(89)90038-8
10.1016/0025-5416(87)90074-7
10.1016/0025-5416(86)90180-1
10.1016/j.ndteint.2011.08.009
10.1016/j.commatsci.2018.04.053
10.1016/j.matdes.2016.05.034
10.1016/j.optlastec.2018.07.017
10.1016/0921-5093(93)90265-g
10.1016/s1001-0521(08)60154-0
10.1016/j.matchemphys.2016.04.060
10.1016/j.matchar.2019.06.009
10.1016/j.corsci.2016.02.010
10.1016/0022-3115(84)90597-x
10.1016/S0026-0657(99)81025-X
10.1016/0956-7151(94)00406-8
10.1016/j.scriptamat.2010.08.036
10.1016/j.corsci.2019.01.032
10.1016/j.commatsci.2008.09.016
10.1016/j.proeng.2011.12.543
10.1016/j.surfcoat.2016.07.100
10.1016/S0257-8972(02)00598-4
10.1016/0010-938x(82)90065-8
10.1016/j.actamat.2019.01.046
10.1016/j.mprp.2015.08.045
10.1016/j.surfcoat.2014.10.060
10.1016/j.jallcom.2015.07.055
10.1016/j.jmatprotec.2008.11.042
10.1016/S0925-8388(98)00572-6
10.1016/S0921-5093(97)00281-5

10.1016/0921-5093(95)10021-0
10.1016/j.solener.2019.10.089
10.1016/j.matchar.2013.09.019
10.1016/j.matpr.2018.02.322
10.1016/j.mtcomm.2018.06.004
10.1016/j.jmps.2015.09.015
10.1016/j.actamat.2013.12.011
10.1016/j.msea.2015.05.026
10.1016/j.jcrysgro.2010.03.041
10.1016/j.msea.2017.07.072
10.1016/j.jallcom.2010.06.112
10.1016/S1006-706X(08)60052-7
10.1016/S0927-0256(97)00193-6
10.1016/j.chemphys.2010.08.021
10.1016/j.surfcoat.2015.09.043
10.1016/j.matchar.2018.04.056
10.1016/S0167-577X(03)00370-7
10.1016/S1003-6326(15)63636-6
10.1016/j.actamat.2007.07.048
10.1016/0036-9748(89)90049-5
10.1016/j.surfcoat.2019.02.021
10.1016/j.jallcom.2014.02.004
10.1016/S0921-5093(00)01979-1
10.1016/0025-5408(88)90095-5
10.1016/j.jallcom.2013.12.040
10.1016/0921-5093(93)90381-n
10.1016/S1359-6454(00)00273-1
10.1016/j.actamat.2018.11.063
10.1016/j.msea.2015.11.036
10.1016/j.actamat.2003.08.007
10.1016/0022-3115(93)90145-o
10.1016/0025-5416(85)90189-2
10.1016/j.msea.2018.12.081
10.1016/j.scriptamat.2007.06.014
10.1016/j.jnucmat.2017.01.051
10.1016/j.msea.2006.12.166
10.1016/j.ijfatigue.2019.03.041
10.1016/j.actamat.2014.07.036
10.1016/S0013-7944(97)00142-2
10.1016/j.msea.2015.03.119
10.1016/j.surfcoat.2019.02.012
10.1016/j.jallcom.2006.05.054
10.1016/j.micron.2011.10.009
10.1016/j.msea.2011.04.029
10.1016/j.msea.2015.04.033
10.1016/j.jmatprotec.2015.06.002
10.1016/j.optlaseng.2007.03.004
10.1016/j.msea.2004.04.001
10.1016/j.jmst.2018.11.016
10.1016/j.actamat.2020.03.015
10.1016/S0143-8166(97)00108-5
10.1016/S0924-0136(99)00437-9
10.1016/S1006-706X(13)60218-6
10.1016/j.promfg.2019.05.022

10.1016/j.jmapro.2019.05.027
10.1016/S0921-5093(98)00477-8
10.1016/j.jallcom.2018.12.081
10.1016/j.ijmecsci.2014.03.010
10.1016/j.matdes.2016.05.125
10.1016/j.jallcom.2019.03.165
10.1016/j.msea.2015.04.042
10.1016/0020-0891(93)90088-o
10.1016/j.matchemphys.2015.10.017
10.1016/j.jmst.2013.04.012
10.1016/j.cma.2017.03.005
10.1016/S0257-8972(00)01162-2
10.1016/j.surfcoat.2010.08.072
10.1016/j.ijfatigue.2011.05.017
10.1016/j.proeng.2015.01.441
10.1016/0010-938x(77)90006-3
10.1016/j.jallcom.2007.04.209
10.1016/0022-3115(90)90368-w
10.1016/j.physb.2005.01.467
10.1016/j.surfcoat.2019.06.084
10.1016/j.msea.2007.11.019
10.1016/0001-6160(88)90214-3
10.1016/j.msea.2016.10.069
10.1016/S0749-6419(99)00060-1
10.1016/0921-5093(89)90308-0
10.1016/j.jallcom.2018.02.343
10.1016/j.msea.2018.02.009
10.1016/0043-1648(78)90111-4
10.1016/j.jallcom.2015.09.249
10.1016/S0963-8695(03)00007-0
10.1016/j.matdes.2018.05.022
10.1016/j.corsci.2017.02.025
10.1016/j.ast.2016.09.022
10.1016/j.jallcom.2010.06.016
10.1016/j.corsci.2019.01.008
10.1016/j.jallcom.2019.05.289
10.1016/j.matdes.2004.02.005
10.1016/j.matdes.2013.12.072
10.1016/j.ijfatigue.2015.11.004
10.1016/j.jallcom.2017.06.016
10.1016/S0022-3115(00)00180-X
10.1016/S1359-6454(01)00265-8
10.1016/0921-5093(91)90713-w
10.1016/j.ijfatigue.2017.01.015
10.1016/j.jpcs.2019.03.016
10.1016/j.ijfatigue.2019.105345
10.1016/j.commatsci.2012.05.071
10.1016/j.matchemphys.2010.04.010
10.1016/j.jallcom.2005.10.009
10.1016/S1359-6462(02)00059-3
10.1016/j.msea.2006.02.039
10.1016/j.surfcoat.2006.07.254
10.1016/j.jcrysgro.2013.11.084
10.1016/j.physleta.2014.10.027

10.1016/j.corsci.2010.11.043
10.1016/j.msea.2006.10.045
10.1016/j.vacuum.2017.09.041
10.1016/j.proeng.2013.03.250
10.1016/j.ijfatigue.2017.10.014
10.1016/S0010-938X(02)00212-3
10.1016/S1000-9361(07)60022-3
10.1016/j.corsci.2005.11.002
10.1016/j.actamat.2015.07.016
10.1016/j.apsusc.2019.03.308
10.1016/j.actamat.2012.01.051
10.1016/j.ijfatigue.2013.06.006
10.1016/S0921-5093(01)01699-9
10.1016/j.apt.2019.02.017
10.1016/j.euromechsol.2019.103895
10.1016/S0924-0136(02)00844-0
10.1016/j.corsci.2018.06.002
10.1016/j.jallcom.2010.02.153
10.1016/j.optlastec.2017.03.021
10.1016/j.actamat.2011.05.005
10.1016/S0025-5408(00)00468-2
10.1016/j.jmatprotec.2004.04.010
10.1016/S0142-1123(03)00172-5
10.1016/S1006-706X(06)60097-6
10.1016/j.jallcom.2016.02.261
10.1016/0025-5416(88)90546-0
10.1016/j.jeurceramsoc.2011.12.016
10.1016/j.msea.2010.10.104
10.1016/j.cirp.2015.04.032
10.1016/j.jallcom.2016.03.186
10.1016/j.mprp.2018.02.002
10.1016/j.ijfatigue.2017.07.021
10.1016/j.intermet.2008.08.014
10.1016/j.corsci.2014.10.030
10.1016/0025-5416(87)90467-8
10.1016/j.matchar.2019.109982
10.1016/j.ijfatigue.2012.11.006
10.1016/j.ijplas.2017.05.013
10.1016/j.matdes.2008.06.064
10.1016/0036-9748(86)90434-5
10.1016/j.ijsolstr.2017.07.033
10.1016/j.vacuum.2018.01.021
10.1016/S0924-0136(01)01024-X
10.1016/j.surfcoat.2020.125640
10.1016/j.corsci.2012.09.025
10.1016/j.jallcom.2018.01.030
10.1016/S0921-5093(00)00985-0
10.1016/j.jallcom.2019.06.070
10.1016/0956-716X(95)00186-Y
10.1016/j.surfcoat.2018.08.089
10.1016/j.matchar.2016.11.038
10.1016/j.msea.2011.01.111
10.1016/j.jmst.2015.10.019
10.1016/j.matlet.2008.01.001

10.1016/j.jallcom.2019.02.019
10.1016/j.jallcom.2019.153455
10.1016/0013-7944(85)90043-8
10.1016/j.surfcoat.2018.12.039
10.1016/j.jmatprotec.2008.03.004
10.1016/0921-5093(91)90740-e
10.1016/j.corsci.2016.11.020
10.1016/j.pnsc.2016.01.003
10.1016/j.mechmat.2016.09.010
10.1016/j.actamat.2012.06.036
10.1016/0956-7151(92)90433-f
10.1016/j.jallcom.2019.03.419
10.1016/S0026-0657(98)80292-0
10.1016/j.pnsc.2019.04.005
10.1016/S1359-6454(97)00267-X
10.1016/j.ijmachtools.2004.05.005
10.1016/j.jmatprotec.2013.04.003
10.1016/j.msea.2019.138391
10.1016/j.msea.2017.08.049
10.1016/0025-5416(88)90805-1
10.1016/S1006-706X(17)30153-X
10.1016/0921-5093(89)90800-9
10.1016/j.msea.2010.03.050
10.1016/j.proeng.2011.12.563
10.1016/S0921-5093(98)00505-X
10.1016/0026-0800(84)90005-3
10.1016/j.wear.2012.01.004
10.1016/j.matchar.2018.05.009
10.1016/S1003-6326(16)64161-4
10.1016/j.actamat.2020.02.018
10.1016/j.mtla.2020.100657
10.1016/j.actamat.2006.08.027
10.1016/j.corsci.2012.01.029
10.1016/j.msea.2018.01.109
10.1016/S1003-6326(11)60920-5
10.1016/j.matdes.2016.08.078
10.1016/j.jallcom.2006.05.106
10.1016/j.actamat.2008.05.022
10.1016/S1359-6462(99)00223-7
10.1016/j.jallcom.2016.10.093
10.1016/j.surfcoat.2019.03.012
10.1016/j.jmst.2018.02.008
10.1016/0022-3115(84)90534-8
10.1016/j.matchar.2017.04.034
10.1016/j.msea.2018.03.090
10.1016/j.ijfatigue.2013.08.025
10.1016/0010-938x(93)90336-f
10.1016/j.msea.2005.02.072
10.1016/j.engfracmech.2017.04.002
10.1016/0921-5093(94)91029-4
10.1016/0040-6090(83)90240-7
10.1016/j.optlastec.2014.03.008
10.1016/S0921-5093(01)01521-0
10.1016/S0026-0657(02)80170-9

10.1016/j.fuproc.2018.03.003
10.1016/j.matdes.2016.09.080
10.1016/j.ceramint.2015.04.012
10.1016/j.ijmachtools.2006.09.031
10.1016/j.jallcom.2017.05.253
10.1016/1359-6462(95)00636-2
10.1016/j.ijfatigue.2012.02.014
10.1016/j.ndteint.2010.06.001
10.1016/S1359-6454(97)00474-6
10.1016/j.scriptamat.2006.10.039
10.1016/j.actamat.2009.06.029
10.1016/0921-5093(96)10255-0
10.1016/j.infrared.2018.04.013
10.1016/j.proeng.2013.03.243
10.1016/j.ijplas.2017.11.003
10.1016/j.surfcoat.2005.07.101
10.1016/j.ijmachtools.2010.03.006
10.1016/j.corsci.2019.05.019
10.1016/j.actamat.2016.03.046
10.1016/j.optlastec.2018.08.003
10.1016/j.scriptamat.2008.12.040
10.1016/j.corsci.2019.108216
10.1016/j.infrared.2012.11.007
10.1016/j.corsci.2017.06.005
10.1016/j.commatsci.2014.05.055
10.1016/j.msea.2009.10.054
10.1016/j.wear.2019.202949
10.1016/S1359-6454(01)00395-0
10.1016/j.msea.2018.07.015
10.1016/j.msea.2017.01.002
10.1016/0025-5416(83)90031-9
10.1016/j.msea.2006.02.030
10.1016/j.matdes.2015.07.171
10.1016/S1359-6462(02)00194-X
10.1016/j.proeng.2013.03.314
10.1016/j.ceramint.2012.09.005
10.1016/j.biomaterials.2004.09.058
10.1016/S1003-6326(11)61474-X
10.1016/0013-7944(89)90307-x
10.1016/j.jallcom.2019.151777
10.1016/j.jallcom.2017.11.130
10.1016/j.matdes.2013.05.042
10.1016/j.ijfatigue.2011.05.011
10.1016/j.intermet.2005.10.005
10.1016/j.jmatprotec.2018.05.002
10.1016/j.surfcoat.2013.05.008
10.1016/j.msea.2017.09.009
10.1016/j.msea.2008.10.017
10.1016/j.matdes.2019.107996
10.1016/0025-5416(87)90402-2
10.1016/j.jnucmat.2010.09.013
10.1016/j.surfcoat.2010.02.049
10.1016/j.actamat.2018.08.018
10.1016/S0921-5093(96)10463-9

10.1016/S0257-8972(03)00870-3
10.1016/j.jallcom.2005.10.010
10.1016/j.jallcom.2011.04.111
10.1016/S1359-6462(02)00266-X
10.1016/j.msea.2016.07.034
10.1016/j.jmst.2014.01.001
10.1016/j.actamat.2011.11.020
10.1016/j.intermet.2012.09.008
10.1016/j.msea.2014.09.033
10.1016/j.msea.2008.04.130
10.1016/j.jallcom.2013.08.144
10.1016/j.corsci.2014.05.018
10.1016/S0921-5093(01)01235-7
10.1016/j.msea.2015.10.087
10.1016/0026-0800(84)90073-9
10.1016/0001-6160(81)90008-0
10.1016/j.matchar.2008.09.009
10.1016/j.commatsci.2005.02.013
10.1016/j.ijplas.2019.01.002
10.1016/j.matchar.2016.12.003
10.1016/0956-716x(91)90462-a
10.1016/j.wear.2012.12.035
10.1016/j.ceramint.2016.09.214
10.1016/j.matlet.2010.06.044
10.1016/0036-9748(81)90307-0
10.1016/j.jallcom.2018.09.200
10.1016/j.matdes.2012.03.024
10.1016/j.matchemphys.2016.07.001
10.1016/j.matdes.2015.03.064
10.1016/j.surfcoat.2006.08.102
10.1016/S0022-3115(03)00116-8
10.1016/j.msea.2004.04.051
10.1016/j.jallcom.2014.07.038
10.1016/j.msea.2016.04.086
10.1016/0956-716x(90)90243-a
10.1016/j.tsf.2016.05.004
10.1016/j.msea.2006.10.169
10.1016/j.rinp.2019.102426
10.1016/j.intermet.2014.07.006
10.1016/j.msea.2019.03.112
10.1016/j.matchar.2006.10.018
10.1016/j.jallcom.2016.08.148
10.1016/j.msea.2005.04.006
10.1016/0025-5416(84)90046-6
10.1016/j.actamat.2019.06.025
10.1016/j.matchemphys.2003.08.001
10.1016/j.ijfatigue.2020.105573
10.1016/S1359-6462(98)00120-1
10.1016/j.msea.2008.12.039
10.1016/j.actamat.2013.04.042
10.1016/S1359-6454(03)00326-4
10.1016/j.actamat.2007.09.042
10.1016/j.surfcoat.2013.09.062
10.1016/j.materresbull.2004.08.003

10.1016/S0142-1123(03)00146-4
10.1016/j.matdes.2013.03.081
10.1016/S0921-5093(97)00352-3
10.1533/9781845693954.4.405
10.1016/j.msea.2016.12.045
10.1016/S0921-5093(00)01288-0
10.1016/j.corsci.2012.08.010
10.1016/0927-0256(95)00053-4
10.1016/j.msea.2017.02.066
10.1016/S1359-6454(99)00123-8
10.1016/j.msea.2019.05.013
10.1016/0257-8972(91)90123-e
10.1016/j.msea.2014.07.051
10.1016/S1003-6326(16)64268-1
10.1016/j.ceramint.2018.01.238
10.1016/j.jallcom.2018.11.087
10.1016/j.ijfatigue.2015.02.014
10.1016/1359-6462(95)00577-3
10.1016/S0257-8972(97)00473-8
10.1016/j.msea.2015.10.088
10.1016/0010-938X(94)00158-3
10.1016/j.pnsc.2015.01.009
10.1016/j.surfcoat.2004.12.019
10.1016/j.dib.2018.08.144
10.1016/0025-5416(86)90321-6
10.1016/j.jmapro.2017.11.018
10.1016/j.matdes.2019.107877
10.1016/j.surfcoat.2009.02.018
10.1016/0025-5416(87)90075-9
10.1016/j.ijhydene.2007.11.022
10.1016/j.jmatprotec.2017.05.003
10.1016/s1001-0521(06)60040-5
10.1016/j.matpr.2016.06.019
10.1016/j.matpr.2018.10.218
10.1016/j.matdes.2015.07.168
10.1016/j.jmapro.2018.03.028
10.1016/S1005-0302(11)60162-X
10.1016/S1003-6326(13)62861-7
10.1016/0956-716x(91)90269-7
10.1016/S1003-6326(13)62446-2
10.1016/j.actamat.2017.06.026
10.1016/j.jallcom.2010.05.108
10.1016/S1005-0302(12)60045-0
10.1016/j.msea.2017.12.043
10.1016/j.physb.2006.06.118
10.1016/j.engfailanal.2012.07.005
10.1016/j.matchar.2019.03.011
10.1016/j.jallcom.2018.02.041
10.1016/j.surfcoat.2009.09.054
10.1016/j.surfcoat.2016.02.050
10.1016/0924-0136(94)01609-5
10.1016/0956-716x(93)90324-1
10.1016/j.ceramint.2016.01.147
10.1016/j.procir.2018.08.257

10.1016/j.msea.2018.11.126
10.1016/j.scriptamat.2010.06.048
10.1016/j.jallcom.2018.04.204
10.1016/j.commatsci.2011.07.054
10.1016/j.surfcoat.2019.125190
10.1016/j.msea.2017.09.116
10.1016/j.jallcom.2008.07.053
10.1016/j.msea.2010.04.097
10.1016/j.actamat.2010.08.035
10.1016/j.msea.2005.09.058
10.1016/j.actamat.2005.02.010
10.1016/j.jmapro.2019.07.042
10.1016/j.matchar.2019.109882
10.1016/S1359-6462(99)00177-3
10.1016/j.msea.2019.138454
10.1016/S0142-1123(98)91101-X
10.1016/j.msea.2008.02.016
10.1016/j.jallcom.2016.02.149
10.1016/0022-3115(87)90090-0
10.1016/j.intermet.2020.106711
10.1016/j.actamat.2012.07.067
10.1016/j.msea.2018.06.027
10.1016/j.surfcoat.2017.05.025
10.1016/0025-5416(82)90084-2
10.1016/j.jallcom.2018.10.084
10.1016/S0927-0248(98)00020-8
10.1016/j.actamat.2009.02.010
10.1016/j.msea.2015.05.004
10.1016/j.commatsci.2016.05.016
10.1016/j.msea.2003.09.019
10.1016/0001-6160(82)90006-2
10.1016/j.ssc.2020.113852
10.1016/j.jallcom.2018.12.232
10.1016/j.scriptamat.2009.05.037
10.1016/j.surfcoat.2004.02.025
10.1016/j.scriptamat.2005.12.033
10.1016/j.jmatprotec.2017.12.014
10.1016/0025-5416(87)90365-x
10.1016/j.compscitech.2005.05.029
10.1016/0921-5093(92)90413-u
10.1016/j.ijplas.2008.04.006
10.1016/0584-8547(72)80016-8
10.1016/j.msea.2018.08.012
10.1016/j.jallcom.2004.11.029
10.1016/j.ndteint.2008.09.008
10.1016/j.addma.2015.03.003
10.1016/j.scriptamat.2019.06.013
10.1016/j.jmatprotec.2013.03.007
10.1016/j.ijheatmasstransfer.2016.04.048
10.1016/0040-6090(83)90226-2
10.1016/S1003-6326(16)64132-8
10.1016/j.jallcom.2014.01.228
10.1016/j.surfcoat.2015.10.072
10.1016/S0955-2219(03)00369-8

10.1016/j.jallcom.2019.152936
10.1016/j.jeurceramsoc.2004.10.024
10.1016/j.msea.2004.02.091
10.1016/j.tafmec.2018.07.002
10.1016/j.jallcom.2015.04.126
10.1016/j.corsci.2009.02.004
10.1016/0921-5093(90)90171-x
10.1016/S0921-5093(03)00403-9
10.1016/j.jallcom.2018.02.268
10.1016/j.matchemphys.2018.01.076
10.1016/S0921-5093(03)00254-5
10.1016/S0921-5093(01)01765-8
10.1016/B978-185617495-4/50006-8
10.1016/j.wear.2005.01.003
10.1016/j.msea.2014.04.102
10.1016/j.electacta.2012.10.112
10.1016/j.apsusc.2018.03.137
10.1016/j.msea.2015.02.056
10.1016/j.actamat.2008.08.027
10.1016/j.ijfatigue.2015.03.022
10.1016/0257-8972(88)90120-x
10.1016/j.actamat.2007.07.057
10.1016/j.msea.2014.01.001
10.1016/j.jmst.2019.04.005
10.1016/j.mtla.2019.100423
10.1016/j.surfcoat.2005.12.030
10.1016/j.ijmecsci.2018.10.058
10.1016/j.commatsci.2018.03.006
10.1016/j.ijfatigue.2016.08.001
10.1016/j.jmatprotec.2004.08.024
10.1016/j.engfailanal.2018.08.004
10.1016/j.jallcom.2020.153698
10.1016/j.matdes.2019.108157
10.1016/0010-938x(83)90026-4
10.1016/S0924-0136(02)00359-X
10.1016/S0921-5093(01)01431-9
10.1016/0026-0800(80)90013-0
10.1016/j.jallcom.2015.09.001
10.1016/j.actamat.2009.10.002
10.1016/S0142-1123(97)83266-5
10.1016/j.msea.2014.12.092
10.1016/j.ceramint.2018.05.222
10.1016/j.jallcom.2016.03.093
10.1016/j.ijfatigue.2019.05.016
10.1016/j.surfcoat.2017.04.040
10.1016/0001-6160(89)90248-4
10.1016/S1006-706X(15)60023-1
10.1016/j.matdes.2011.07.050
10.1016/j.intermet.2005.02.006
10.1016/j.matdes.2017.01.031
10.1016/S1003-6326(14)63493-2
10.1016/S1005-0302(11)60034-0
10.1016/j.matlet.2019.01.011
10.1016/j.corsci.2018.01.026

10.1016/j.ijfatigue.2019.06.008
10.1016/j.matdes.2016.09.007
10.1016/j.corsci.2013.10.006
10.1016/j.msea.2006.05.110
10.1016/j.jmst.2016.04.017
10.1016/S0167-577X(03)00077-6
10.1016/0025-5416(85)90110-7
10.1016/j.matdes.2015.04.047
10.1016/j.ijpvp.2020.104064
10.1016/S0921-5093(97)00074-9
10.1016/j.msea.2018.09.093
10.1016/j.physc.2010.05.155
10.1016/j.engfracmech.2017.08.011
10.1016/0956-716x(93)90024-m
10.1016/j.msea.2017.12.104
10.1016/j.corsci.2005.05.005
10.1016/j.tafmec.2018.06.011
10.1016/j.corsci.2019.06.014
10.1016/j.actamat.2016.10.059
10.1016/S0921-5093(96)10384-1
10.1016/S1359-6462(01)01231-3
10.1016/j.optlastec.2012.06.043
10.1016/j.scriptamat.2006.10.002
10.1016/j.calphad.2014.03.002
10.1016/j.actamat.2014.09.053
10.1016/0013-7944(79)90135-8
10.1016/j.ijplas.2005.05.004
10.1016/j.jmps.2013.07.011
10.1016/j.conbuildmat.2019.117384
10.1016/j.matdes.2019.108429
10.1016/j.msea.2017.03.083
10.1016/0921-4526(89)90764-3
10.1016/j.msea.2017.05.113
10.1016/j.camss.2017.11.002
10.1016/0956-716x(93)90175-r
10.1016/0010-938x(85)90056-3
10.1016/j.intermet.2017.09.015
10.1016/0022-3115(84)90224-1
10.1016/j.surfcoat.2019.04.060
10.1016/j.jallcom.2014.08.233
10.1016/j.jallcom.2014.05.132
10.1016/j.memsci.2016.01.050
10.1016/0022-3115(92)90566-4
10.1016/S0257-8972(03)00009-4
10.1016/j.vacuum.2019.109069
10.1016/j.jmatprotec.2007.02.045
10.1016/j.surfcoat.2005.03.022
10.1016/0040-6090(78)90044-5
10.1016/0921-5093(94)91053-7
10.1016/j.surfcoat.2015.06.048
10.1016/j.msea.2008.04.103
10.1016/j.prostr.2020.01.129
10.1016/j.msea.2009.11.035
10.1016/0026-0800(86)90019-4

10.1016/j.engfailanal.2016.12.018
10.1016/j.commatsci.2014.03.054
10.1016/S1003-6326(18)64854-X
10.1016/j.actamat.2009.02.047
10.1016/0013-7944(85)90033-5
10.1016/j.jnucmat.2003.12.011
10.1016/j.electacta.2007.10.048
10.1016/S1003-6326(18)64692-8
10.1016/j.jeurceramsoc.2013.03.002
10.1016/j.expthermflusci.2017.04.024
10.1016/0025-5416(71)90053-x
10.1016/1359-6454(95)00423-8
10.1016/S0011-2275(03)00123-1
10.1016/1359-6454(95)00349-5
10.1016/j.msea.2014.08.001
10.1016/j.matchar.2017.04.011
10.1016/j.optlaseng.2013.01.006
10.1016/j.ijmecsci.2018.01.024
10.1016/j.jallcom.2013.09.121
10.1016/0001-6160(79)90110-x
10.1016/j.mtla.2019.100555
10.1016/j.matchar.2019.03.037
10.1016/j.jallcom.2018.01.323
10.1016/S0925-8388(00)01187-7
10.1016/j.prostr.2020.01.063
10.1016/S1000-9361(09)60244-2
10.1016/S1359-6454(99)00390-0
10.1016/j.ndteint.2006.05.001
10.1016/j.msea.2015.08.044
10.1016/j.matdes.2015.05.014
10.1016/j.msea.2006.03.011
10.1016/j.msea.2019.138361
10.1016/j.ijfatigue.2018.07.008
10.1016/j.msea.2004.07.007
10.1016/j.msea.2018.01.082
10.1016/j.vacuum.2018.04.042
10.1016/j.acme.2018.05.008
10.1016/S0029-5493(98)00287-8
10.1016/j.ijfatigue.2014.08.015
10.1016/S0167-577X(00)00119-1
10.1016/S0921-5093(01)01747-6
10.1016/j.mechmat.2017.08.008
10.1016/j.procir.2018.05.088
10.1016/j.scriptamat.2012.01.001
10.1016/j.jallcom.2010.06.168
10.1016/0956-7151(93)90170-w
10.1016/j.msea.2015.06.055
10.1016/0001-6160(88)90004-1
10.1016/j.tca.2007.09.006
10.1016/S1003-6326(18)64726-0
10.1016/j.matpr.2017.02.266
10.1016/j.rinp.2019.102828
10.1016/j.fuproc.2015.10.009
10.1016/0029-5493(93)90146-z

10.1016/j.ijfatigue.2006.03.005
10.1016/j.engfracmech.2018.05.042
10.1016/S0924-0136(01)00785-3
10.1016/0043-1648(91)90106-5
10.1016/j.jallcom.2010.06.114
10.1016/0025-5416(87)90079-6
10.1016/j.surfcoat.2006.09.081
10.1016/S1359-6462(97)00527-7
10.1016/j.commatsci.2011.07.055
10.1016/j.jcrysgro.2015.11.001
10.1016/j.jallcom.2009.05.095
10.1016/j.wear.2018.01.011
10.1016/j.matdes.2012.11.043
10.1016/j.msea.2019.04.011
10.1016/j.ijengsci.2011.03.008
10.1016/j.matdes.2016.12.026
10.1016/0040-6090(93)90429-s
10.1016/0010-938x(87)90071-0
10.1016/j.msea.2012.10.095
10.1016/j.surfcoat.2016.12.082
10.1016/j.tsf.2004.02.046
10.1016/j.jallcom.2011.02.146
10.1016/j.scriptamat.2016.11.039
10.1016/S0921-5093(97)00584-4
10.1016/j.actamat.2011.04.042
10.1016/j.scriptamat.2019.07.027
10.1016/j.jpowsour.2006.10.039
10.1016/j.msea.2003.09.009
10.1016/j.surfcoat.2014.07.096
10.1016/j.matchar.2016.03.024
10.1016/j.jnucmat.2014.08.003
10.1016/S0921-5093(01)01584-2
10.1016/j.surfcoat.2005.10.042
10.1016/j.surfcoat.2019.07.037
10.1016/j.matpr.2017.07.083
10.1016/j.ijfatigue.2016.11.015
10.1016/j.pnsc.2013.03.005
10.1016/j.corsci.2019.01.014
10.1016/j.ast.2019.105422
10.1016/j.matdes.2014.04.020
10.1016/0013-7944(84)90075-4
10.1016/j.msea.2018.01.104
10.1016/0921-5093(89)90812-5
10.1016/j.calphad.2017.06.006
10.1016/j.msea.2017.03.046
10.1016/j.ijplas.2012.09.011
10.1016/j.actamat.2008.10.039
10.1016/j.intermet.2017.04.015
10.1016/j.matlet.2012.05.057
10.1016/j.jallcom.2008.06.079
10.1016/j.engfailanal.2016.04.016
10.1016/j.jallcom.2018.04.102
10.1016/j.matlet.2011.05.021
10.1016/j.commatsci.2010.05.032

10.1016/j.matchemphys.2018.06.038
10.1016/j.jallcom.2014.08.187
10.1016/S0921-5093(00)01853-0
10.1016/j.jallcom.2020.154790
10.1016/j.triboint.2019.01.036
10.1016/j.intermet.2013.10.028
10.1016/j.scriptamat.2007.07.012
10.1016/j.msea.2017.12.034
10.1016/j.jmps.2019.07.011
10.1016/j.vacuum.2018.12.047
10.1016/j.matchar.2012.05.001
10.1016/j.jallcom.2003.11.167
10.1016/j.corsci.2014.03.002
10.1016/j.intermet.2005.11.005
10.1016/j.jnucmat.2013.06.034
10.1016/j.corsci.2008.06.033
10.1016/j.engfracmech.2013.03.022
10.1016/S0966-9795(97)00031-9
10.1016/0029-5493(84)90124-9
10.1016/j.msea.2014.06.036
10.1016/0010-938x(91)90055-t
10.1016/j.matdes.2019.107826
10.1016/j.promfg.2018.07.230
10.1016/j.ceramint.2008.01.009
10.1016/j.jallcom.2004.02.053
10.1016/j.msea.2015.10.004
10.1016/j.surfcoat.2012.04.026
10.1016/S1006-706X(08)60049-7
10.1016/j.surfcoat.2017.10.042
10.1016/0956-716x(94)90466-9
10.1016/j.optlastec.2019.105917
10.1016/j.actamat.2016.08.081
10.1016/j.matdes.2018.09.048
10.1016/j.corsci.2009.01.002
10.1016/j.msea.2006.10.048
10.1016/j.msea.2016.11.031
10.1016/j.actamat.2017.02.007
10.1016/j.jallcom.2018.11.055
10.1016/j.matchar.2017.06.021
10.1016/j.advengsoft.2015.11.006
10.1016/j.nimb.2005.12.032
10.1016/S0921-5093(96)10489-5
10.1016/j.jmatprotec.2008.10.013
10.1016/j.cirp.2009.03.109
10.1016/j.surfcoat.2004.06.003
10.1016/j.intermet.2014.06.011
10.1016/0956-716X(95)00047-Y
10.1016/S0257-8972(02)00865-4
10.1016/0956-716x(94)90294-1
10.1016/j.ijfatigue.2017.12.006
10.1016/j.actamat.2006.03.015
10.1016/j.intermet.2011.11.018
10.1016/j.actamat.2012.09.005
10.1016/j.jallcom.2018.01.406

10.1016/j.msea.2006.10.182
10.1016/j.proeng.2015.12.270
10.1016/j.msea.2006.10.186
10.1016/j.jclepro.2018.01.251
10.1016/j.surfcoat.2017.12.047
10.1016/j.scriptamat.2014.05.012
10.1016/j.msea.2006.11.152
10.1016/j.piutam.2017.06.017
10.1016/j.jallcom.2016.06.209
10.1016/j.proeng.2013.03.339
10.1016/j.jmst.2018.12.006
10.1016/j.msea.2018.04.045
10.1016/j.matdes.2013.02.024
10.1016/j.ijfatigue.2018.01.017
10.1016/S0364-5916(01)00049-9
10.1016/j.jallcom.2013.12.014
10.1016/j.jeurceramsoc.2015.02.024
10.1016/S1359-6462(00)00493-0
10.1016/S0921-5093(99)00124-0
10.1016/j.tsf.2015.12.039
10.1016/j.scriptamat.2006.03.014
10.1016/S0042-207X(02)00745-5
10.1016/j.jallcom.2018.08.144
10.1016/j.matpr.2015.07.182
10.1016/S0167-6636(97)00005-7
10.1016/0921-5093(91)90338-n
10.1016/j.msea.2014.11.099
10.1016/j.jnucmat.2015.07.045
10.1016/j.ceramint.2019.11.228
10.1016/j.jallcom.2017.01.042
10.1016/j.matdes.2014.02.041
10.1016/j.cja.2015.06.021
10.1016/j.surfcoat.2014.01.001
10.1016/j.actamat.2008.01.032
10.1016/j.jallcom.2012.05.004
10.1016/j.jallcom.2019.152940
10.1016/j.ultras.2018.05.006
10.1016/j.promfg.2016.12.038
10.1016/S1005-0302(10)60143-0
10.1016/0025-5416(88)90515-0
10.1016/S0966-9795(02)00165-6
10.1016/j.msea.2014.10.064
10.1016/j.jmrt.2020.03.068
10.1016/j.commatsci.2009.09.013
10.1016/j.solmat.2019.04.021
10.1016/j.actamat.2018.12.039
10.1016/S1006-706X(09)60017-0
10.1016/j.actamat.2013.10.069
10.1016/0001-6160(75)90073-5
10.1016/j.jallcom.2013.06.045
10.1016/j.apsusc.2014.05.043
10.1016/j.ijmecsci.2019.105194
10.1016/j.msea.2019.138791
10.1016/j.actamat.2012.12.017

10.1016/0022-3115(89)90594-1
10.1016/S1359-6454(97)00062-1
10.1016/0025-5416(86)90107-2
10.1016/j.ijhydene.2018.11.206
10.1016/j.actamat.2010.01.014
10.1016/j.ijfatigue.2011.07.009
10.1016/j.ceramint.2016.09.127
10.1016/j.procir.2019.03.277
10.1016/j.matchemphys.2005.06.042
10.1016/0025-5416(84)90182-4
10.1016/j.jallcom.2016.09.228
10.1016/j.scriptamat.2008.04.025
10.1016/j.matchar.2013.04.005
10.1016/j.jallcom.2015.11.072
10.1016/S1359-6462(97)00476-4
10.1016/S0925-8388(03)00284-6
10.1016/j.surfcoat.2016.12.118
10.1016/0022-3115(93)90012-n
10.1016/j.msea.2015.11.066
10.1016/S0022-5096(00)00006-5
10.1016/j.apsusc.2012.01.167
10.1016/j.micron.2018.01.011
10.1016/j.commatsci.2009.03.023
10.1016/0142-1123(81)90017-7
10.1016/j.msea.2019.04.100
10.1016/0036-9748(84)90200-x
10.1016/j.matchar.2017.07.021
10.1016/j.matlet.2010.08.066
10.1016/j.surfcoat.2017.04.044
10.1016/j.matchemphys.2017.09.057
10.1016/j.jmatprotec.2018.04.036
10.1016/0025-5416(83)90202-1
10.1016/j.surfcoat.2009.04.016
10.1016/j.matdes.2011.01.017
10.1016/j.intermet.2019.106659
10.1016/j.scriptamat.2018.07.041
10.1016/j.scriptamat.2016.10.012
10.1016/j.promfg.2018.07.070
10.1016/j.msea.2005.01.058
10.1016/j.cpc.2019.106863
10.1016/0956-716x(94)90240-2
10.1016/j.ijfatigue.2019.105431
10.1016/j.matchar.2019.04.018
10.1016/j.jmst.2016.01.016
10.1016/j.actamat.2014.06.020
10.1016/j.jallcom.2016.08.216
10.1016/j.jmatprotec.2013.06.025
10.1016/B978-1-85573-424-1.50032-0
10.1016/j.msea.2017.01.039
10.1016/j.corsci.2017.08.003
10.1016/j.msea.2007.03.080
10.1016/S1003-6326(18)64794-6
10.1016/S0921-5093(99)00623-1
10.1016/j.ijsolstr.2018.01.001

10.1016/S1359-6462(96)00408-3
10.1016/S0921-5093(01)01585-4
10.1016/j.jmatprotec.2018.12.007
10.1016/0001-6160(84)90098-1
10.1016/0956-7151(94)00252-D
10.1016/j.corsci.2018.08.043
10.1016/S0921-5093(03)00372-1
10.1016/j.msea.2012.02.099
10.1016/j.jallcom.2019.152935
10.1016/j.jallcom.2017.03.290
10.1016/j.actamat.2015.01.029
10.1016/j.msea.2015.06.087
10.1016/j.ceramint.2016.12.014
10.1016/j.ijfatigue.2012.12.009
10.1016/0040-6090(81)90012-2
10.1016/0025-5416(82)90130-6
10.1016/j.matdes.2018.08.039
10.1016/j.ijmachtools.2009.01.012
10.1016/j.msea.2014.02.078
10.1016/j.ijfatigue.2019.105365
10.1016/j.hydromet.2018.02.002
10.1016/0026-0800(82)90028-3
10.1016/j.ijrmhm.2009.05.009
10.1016/j.actamat.2012.05.023
10.1016/S0921-5093(01)01881-0
10.1016/j.jallcom.2016.05.122
10.1016/j.jallcom.2014.11.155
10.1016/j.matchar.2012.02.004
10.1016/S1359-6462(02)00168-9
10.1016/j.msea.2004.08.083
10.1016/S0921-5093(01)01278-3
10.1016/0010-938x(83)90029-x
10.1016/j.jcrysgro.2017.09.015
10.1016/s1468-6996(01)00060-2
10.1016/0001-6160(89)90131-4
10.1016/j.corsci.2009.01.019
10.1016/j.jallcom.2017.02.031
10.1016/j.optlastec.2019.105723
10.1016/j.tca.2013.02.004
10.1016/j.ijfatigue.2019.105400
10.1016/j.corsci.2016.09.011
10.1016/j.pnsc.2014.05.004
10.1016/j.commatsci.2013.11.003
10.1016/S0925-8388(03)00587-5
10.1016/j.msea.2019.03.115
10.1016/j.msea.2019.01.015
10.1016/j.matlet.2006.04.037
10.1016/j.msea.2008.07.052
10.1016/j.prostr.2020.01.080
10.1016/j.actamat.2012.06.028
10.1016/0308-0161(94)90062-0
10.1016/j.msea.2018.11.034
10.1016/j.intermet.2008.04.003
10.1016/j.msea.2019.05.119

10.1016/j.actamat.2008.05.013
10.1016/j.jallcom.2017.01.145
10.1016/j.jallcom.2020.153999
10.1016/j.scriptamat.2017.05.028
10.1016/j.surfcoat.2005.11.108
10.1016/j.jallcom.2019.01.288
10.1016/j.corsci.2019.108240
10.1016/j.actamat.2015.06.035
10.1016/j.jallcom.2004.08.100
10.1016/j.ijfatigue.2014.09.011
10.1016/j.jmatprotec.2007.01.001
10.1016/j.commatsci.2019.109331
10.1016/j.commatsci.2013.08.051
10.1016/j.actamat.2015.07.006
10.1016/j.procir.2018.08.297
10.1016/0001-6160(87)90083-6
10.1016/j.matchar.2015.04.018
10.1016/j.corsci.2018.01.006
10.1016/S0167-6636(00)00070-3
10.1016/j.matchar.2016.11.016
10.1016/S0026-0657(13)70202-9
10.1016/j.jallcom.2015.08.118
10.1016/j.corsci.2011.02.022
10.1016/j.mtla.2019.100570
10.1016/j.addma.2017.05.006
10.1016/j.msea.2015.06.011
10.1016/j.ssc.2014.03.012
10.1016/j.scriptamat.2011.09.001
10.1016/S0920-3796(02)00099-6
10.1016/j.actamat.2018.11.031
10.1016/j.ijfatigue.2014.02.005
10.1016/0043-1648(95)06644-6
10.1016/j.corsci.2016.03.014
10.1016/j.jallcom.2018.08.187
10.1016/j.matchar.2018.12.032
10.1016/0022-3115(87)90049-3
10.1016/j.msea.2019.03.096
10.1016/j.jallcom.2019.07.221
10.1016/j.jallcom.2018.12.312
10.1016/S1566-1369(02)80080-2
10.1016/j.matpr.2018.06.237
10.1016/j.msea.2016.09.039
10.1016/j.intermet.2019.106612
10.1016/j.surfcoat.2008.07.023
10.1016/j.matchar.2010.02.006
10.1016/j.jallcom.2014.06.071
10.1016/j.ijfatigue.2008.11.002
10.1016/j.jallcom.2017.04.022
10.1016/j.matlet.2004.12.050
10.1016/j.matpr.2017.07.093
10.1016/j.matchar.2020.110175
10.1016/0013-7944(86)90080-9
10.1016/j.electacta.2009.09.004
10.1016/j.surfcoat.2015.12.066

10.1016/j.matlet.2007.03.003
10.1016/0921-5093(96)10402-0
10.1016/j.jallcom.2016.09.245
10.1016/j.apsusc.2009.07.092
10.1016/j.msea.2011.05.033
10.1016/0308-0161(93)90094-a
10.1016/S0141-6359(01)00078-2
10.1016/j.jallcom.2015.10.198
10.1016/S1003-6326(15)63991-7
10.1016/j.msea.2019.138481
10.1016/0045-7949(92)90217-n
10.1016/j.jmapro.2020.02.007
10.1016/j.jallcom.2004.09.004
10.1016/j.actamat.2004.04.028
10.1016/S0257-8972(02)00740-5
10.1016/j.surfcoat.2013.11.034
10.1016/j.msea.2007.04.065
10.1016/S0921-5093(01)01224-2
10.1016/j.surfcoat.2017.09.035
10.1016/j.jcrysgro.2017.03.021
10.1016/j.jallcom.2013.02.042
10.1016/j.tca.2016.06.019
10.1016/j.optlaseng.2019.105941
10.1016/0304-8853(92)90304-7
10.1016/j.matdes.2008.06.033
10.1016/j.jmatprotec.2014.07.006
10.1016/j.jallcom.2017.07.066
10.1016/j.jestch.2018.03.005
10.1016/j.msea.2020.139196
10.1016/j.actamat.2014.03.001
10.1016/j.jallcom.2016.12.174
10.1016/0955-2219(96)00016-7
10.1016/j.matpr.2017.06.075
10.1016/j.jallcom.2018.10.053
10.1016/j.optlastec.2017.12.012
10.1016/j.jmatprotec.2019.04.034
10.1016/j.msea.2013.04.032
10.1016/j.corsci.2012.01.008
10.1016/j.matdes.2018.07.051
10.1016/j.matdes.2013.11.015
10.1016/j.jpowsour.2010.10.058
10.1016/S1003-6326(11)60995-3
10.1016/j.cja.2014.03.015
10.1016/j.corsci.2018.05.040
10.1016/j.actamat.2015.03.050
10.1016/0001-6160(83)90059-7
10.1016/j.engfailanal.2007.06.011
10.1016/S0921-5093(97)00142-1
10.1016/j.msea.2017.01.013
10.1016/S1003-6326(11)61434-9
10.1016/j.procir.2018.08.189
10.1016/j.cossms.2016.12.002
10.1016/S0257-8972(96)02937-4
10.1016/S0013-7944(00)00122-3

10.1016/j.msea.2018.04.038
10.1016/j.jmst.2019.03.002
10.1016/j.actamat.2014.03.052
10.1016/j.surfcoat.2017.11.037
10.1016/j.surfcoat.2018.08.015
10.1016/S1359-6462(97)00559-9
10.1016/j.msea.2012.05.071
10.1016/S1359-6462(99)00166-9
10.1016/j.jeurceramsoc.2007.08.003
10.1016/j.msea.2019.138439
10.1016/S1359-6462(97)00222-4
10.1016/S0921-5093(01)01825-1
10.1016/j.matpr.2019.11.298
10.1016/j.scriptamat.2012.08.006
10.1016/j.msea.2014.06.076
10.1016/j.ijfatigue.2008.01.001
10.1016/j.matdes.2018.04.019
10.1016/0956-7151(91)90237-u
10.1016/0956-716x(94)90284-4
10.1016/0025-5416(86)90082-0
10.1016/S0749-6419(01)00029-8
10.1016/j.actamat.2009.11.047
10.1016/j.jmrt.2014.03.003
10.1016/j.optlastec.2017.09.054
10.1016/j.actamat.2018.01.062
10.1016/j.engfailanal.2019.02.048
10.1016/S0925-8388(01)01812-6
10.1016/j.ijfatigue.2011.11.007
10.1016/j.ijfatigue.2020.105580
10.1016/j.applthermaleng.2017.06.113
10.1016/j.engfailanal.2012.10.028
10.1016/S1000-9361(09)60231-4
10.1016/0956-7151(94)00381-Q
10.1016/j.corsci.2011.05.018
10.1016/j.vacuum.2014.07.003
10.1016/j.ijfatigue.2019.105305
10.1016/j.cirpj.2016.07.005
10.1016/j.matdes.2017.07.024
10.1016/j.msea.2010.11.081
10.1016/j.matpr.2018.02.113
10.1016/j.proeng.2013.03.308
10.1016/j.msea.2005.07.004
10.1016/j.tsf.2006.10.007
10.1016/0043-1648(95)07180-6
10.1016/j.msea.2008.02.013
10.1016/0956-716x(94)90546-0
10.1016/S0921-5093(97)00457-7
10.1016/j.intermet.2020.106744
10.1016/j.surfcoat.2009.03.032
10.1016/j.msea.2003.08.008
10.1016/j.actamat.2017.05.003
10.1016/j.fuel.2013.04.005
10.1016/j.ijfatigue.2013.10.007
10.1016/j.jallcom.2013.01.129

10.1016/j.msea.2008.12.051
10.1016/0025-5416(88)90414-4
10.1016/j.ijhydene.2017.08.039
10.1016/j.jallcom.2015.09.267
10.1016/j.jallcom.2017.02.205
10.1016/j.mspro.2015.04.176
10.1016/0142-1123(96)89367-4
10.1016/j.surfcoat.2006.07.018
10.1016/j.msea.2012.02.059
10.1016/j.actamat.2008.03.046
10.1016/j.matdes.2016.06.023
10.1016/j.jestch.2016.06.010
10.1016/j.jallcom.2018.09.217
10.1016/S1359-6462(98)00337-6
10.1016/j.corsci.2013.07.041
10.1016/j.msea.2007.11.010
10.1016/j.msea.2016.05.051
10.1016/j.corsci.2014.11.043
10.1016/S0921-5093(99)00579-1
10.1016/j.matdes.2020.108605
10.1016/S0921-5093(01)01911-6
10.1016/j.ijplas.2014.03.004
10.1016/0043-1648(93)90441-n
10.1016/j.jmapro.2018.10.017
10.1016/j.matchar.2015.08.006
10.1016/j.corsci.2014.08.016
10.1016/S0257-8972(98)00664-1
10.1016/j.optlaseng.2013.10.010
10.1016/0025-5416(83)90140-4
10.1016/0013-7944(86)90127-x
10.1016/S0140-6701(02)80614-2
10.1016/S1359-6462(03)00357-9
10.1016/S1359-6462(97)00103-6
10.1016/j.mechmat.2015.01.020
10.1016/j.ijfatigue.2016.12.003
10.1016/j.actamat.2015.09.050
10.1016/j.jnucmat.2017.04.045
10.1016/0001-6160(86)90082-9
10.1016/j.matdes.2015.10.082
10.1016/S0257-8972(99)00351-5
10.1016/j.msea.2018.05.016
10.1016/j.physb.2006.05.262
10.1016/S1359-6454(97)00425-4
10.1016/j.scriptamat.2008.08.009
10.1016/j.surfcoat.2017.12.017
10.1016/j.cej.2004.01.006
10.1016/S0927-0256(03)00106-X
10.1016/j.matpr.2015.07.041
10.1016/j.msea.2013.01.035
10.1016/S0966-9795(98)00010-7
10.1016/j.jpowsour.2009.12.066
10.1016/0022-3115(81)90475-x
10.1016/j.matchar.2019.05.024
10.1016/j.optlastec.2017.10.015

10.1016/j.msea.2003.11.081
10.1016/j.jallcom.2016.08.081
10.1016/j.scriptamat.2005.04.009
10.1016/j.actamat.2017.12.032
10.1016/j.powtec.2019.10.068
10.1016/j.actamat.2017.04.060
10.1016/0921-5093(91)90375-w
10.1016/j.jallcom.2020.153948
10.1016/j.jcrysgro.2018.06.018
10.1016/0025-5416(84)90026-0
10.1016/j.solmat.2017.07.036
10.1016/j.ceramint.2018.10.252
10.1016/j.corsci.2017.02.001
10.1016/j.intermet.2012.11.008
10.1016/0026-0800(86)90006-6
10.1016/j.vacuum.2019.06.013
10.1016/S1003-6326(11)61210-7
10.1016/j.scriptamat.2017.05.013
10.1016/j.jallcom.2019.153263
10.1016/j.paerosci.2018.01.001
10.1016/S0921-5093(03)00220-X
10.1016/S0167-577X(96)00109-7
10.1016/S1005-0302(12)60148-0
10.1016/0025-5416(85)90088-6
10.1016/j.corsci.2019.05.001
10.1016/0921-5093(91)90845-e
10.1016/j.intermet.2018.06.013
10.1016/j.actamat.2018.01.050
10.1016/j.actamat.2009.06.049
10.1016/S0921-5093(99)00208-7
10.1016/j.corsci.2011.04.025
10.1016/j.jallcom.2015.02.152
10.1016/j.ijplas.2019.09.010
10.1016/j.jclepro.2017.06.186
10.1016/j.matchar.2011.06.004
10.1016/S0921-5093(97)00046-4
10.1016/j.msea.2007.09.017
10.1016/j.vacuum.2018.07.046
10.1016/0043-1648(86)90274-7
10.1016/j.actamat.2014.04.037
10.1016/j.ijmachtools.2018.03.005
10.1016/0001-6160(87)90125-8
10.1016/j.matdes.2009.11.041
10.1016/S0921-5093(00)01822-0
10.1016/j.msea.2006.08.080
10.1016/j.matdes.2015.07.030
10.1016/j.wear.2015.09.014
10.1016/j.jallcom.2015.11.082
10.1016/0921-5093(93)90701-f
10.1016/j.actamat.2007.04.008
10.1016/j.msea.2019.03.005
10.1016/j.commatsci.2004.11.005
10.1016/j.ijpvp.2015.07.001
10.1016/j.msea.2017.12.089

10.1016/j.scriptamat.2004.04.019
10.1016/S0925-8388(03)00699-6
10.1016/j.matdes.2016.05.030
10.1016/0001-6160(88)90005-3
10.1016/j.wear.2018.12.076
10.1016/j.msea.2012.06.097
10.1016/j.matchemphys.2011.05.019
10.1016/j.msea.2020.139005
10.1016/j.msea.2017.11.002
10.1016/j.jallcom.2016.10.029
10.1016/j.jmatprotec.2010.08.031
10.1016/j.jallcom.2009.03.091
10.1016/j.commatsci.2014.09.042
10.1016/j.matpr.2017.02.072
10.1016/j.surfcoat.2012.09.017
10.1016/j.apsusc.2019.07.207
10.1016/s1875-5372(12)60053-8
10.1016/j.proeng.2011.11.094
10.1016/j.matdes.2012.11.002
10.1016/0022-3115(90)90348-q
10.1016/j.optlastec.2016.01.015
10.1016/S0304-3991(97)00048-X
10.1016/j.promfg.2019.05.036
10.1016/j.jallcom.2006.08.096
10.1016/j.matchar.2015.04.011
10.1016/j.jallcom.2005.07.070
10.1016/j.corsci.2015.01.048
10.1016/S0020-7683(98)00320-5
10.1016/0308-0161(94)00046-L
10.1016/j.jallcom.2013.09.035
10.1016/j.measurement.2018.07.017
10.1016/j.matchemphys.2004.04.006
10.1016/j.msea.2013.04.087
10.1016/j.acme.2015.03.006
10.1016/j.scriptamat.2017.01.002
10.1016/j.actamat.2018.04.001
10.1016/j.matpr.2017.07.117
10.1016/0022-3115(88)90043-8
10.1016/j.cjph.2019.07.001
10.1016/j.corsci.2019.05.007
10.1016/j.measurement.2018.03.015
10.1016/j.jnucmat.2014.01.029
10.1016/j.msea.2007.03.056
10.1016/j.matdes.2017.01.075
10.1016/0022-3115(83)90327-6
10.1016/j.applthermaleng.2009.04.011
10.1016/j.actamat.2012.06.043
10.1016/j.surfcoat.2014.11.056
10.1016/j.scriptamat.2018.12.013
10.1016/j.precisioneng.2017.05.012
10.1016/0921-5093(95)03299-1
10.1016/j.matdes.2012.09.031
10.1016/j.actamat.2017.10.049
10.1016/S1359-6462(02)00092-1

10.1016/j.ijhydene.2017.06.211
10.1016/j.jallcom.2013.10.138
10.1016/S0257-8972(99)00339-4
10.1016/j.msea.2011.02.078
10.1016/j.proeng.2010.03.115
10.1016/S0963-8695(97)87312-4
10.1016/S0966-9795(98)00024-7
10.1016/j.jallcom.2018.11.289
10.1016/0308-0161(91)90033-x
10.1016/j.proeng.2010.03.146
10.1016/j.jallcom.2016.11.211
10.1016/S0924-0136(98)00401-4
10.1016/0001-6160(76)90072-9
10.1016/j.proeng.2014.12.677
10.1016/j.jallcom.2015.01.031
10.1016/j.jallcom.2016.07.003
10.1016/j.jmapro.2017.09.010
10.1016/j.actamat.2020.01.004
10.1016/j.msea.2008.08.048
10.1016/j.matdes.2014.08.035
10.1016/0013-7944(95)00038-W
10.1016/j.powtec.2015.12.017
10.1016/j.msea.2016.07.032
10.1016/j.scriptamat.2003.08.027
10.1016/j.msea.2011.12.041
10.1016/0011-2275(76)90039-4
10.1016/j.matdes.2016.05.013
10.1016/j.engfracmech.2014.06.005
10.1016/j.actamat.2018.05.025
10.1016/0956-7151(91)90147-s
10.1016/0142-1123(84)90037-9
10.1016/S1359-0286(97)80115-5
10.1016/j.tsf.2007.07.128
10.1016/S0921-5093(03)00593-8
10.1016/0142-1123(91)90359-7
10.1016/j.actamat.2014.08.035
10.1016/j.jallcom.2016.12.397
10.1016/j.jallcom.2016.07.130
10.1016/j.intermet.2016.08.002
10.1016/j.msea.2015.10.056
10.1016/j.jmst.2020.02.021
10.1016/S0026-0657(01)80746-3
10.1016/j.jallcom.2006.09.123
10.1016/j.mlblux.2019.100003
10.1016/j.jallcom.2019.06.029
10.1016/j.jmapro.2019.06.032
10.1016/j.promfg.2018.06.115
10.1016/B978-1-85573-424-1.50016-2
10.1016/j.intermet.2008.02.015
10.1016/j.corsci.2012.11.010
10.1016/j.surfcoat.2013.04.037
10.1016/j.pnsc.2014.06.003
10.1016/j.nimb.2012.12.105
10.1016/S0956-716X(99)80010-7

10.1016/0308-0161(95)00066-6
10.1016/j.ijsolstr.2017.03.025
10.1016/j.surfcoat.2007.06.044
10.1016/j.commatsci.2013.07.015
10.1016/j.egypro.2014.10.194
10.1016/S0257-8972(02)00165-2
10.1016/S0142-1123(97)00038-8
10.1016/j.calphad.2019.101733
10.1016/S1003-6326(11)60892-3
10.1016/0026-0657(96)93958-2
10.1016/j.jmps.2012.09.015
10.1016/j.commatsci.2018.09.055
10.1016/j.msea.2005.11.084
10.1016/j.msea.2017.11.035
10.1016/j.actamat.2008.04.057
10.1016/j.ceramint.2011.11.073
10.1016/j.matdes.2019.107784
10.1016/j.proeng.2015.12.280
10.1016/1044-5803(91)90003-m
10.1016/j.jallcom.2013.07.045
10.1016/S0010-938X(00)00036-6
10.1016/j.msea.2010.08.053
10.1016/j.matdes.2017.06.004
10.1016/S1003-6326(16)64266-8
10.1016/S1359-6462(02)00566-3
10.1016/j.matdes.2019.107656
10.1016/j.rinp.2016.03.011
10.1016/j.msea.2018.07.053
10.1016/j.commatsci.2017.09.036
10.1016/0257-8972(90)90087-s
10.1016/j.pnsc.2017.07.005
10.1016/j.msea.2016.02.074
10.1016/j.engfailanal.2005.10.010
10.1016/j.msea.2017.11.048
10.1016/j.msea.2006.06.014
10.1016/j.wear.2019.203012
10.1016/S1359-6454(02)00119-2
10.1016/j.surfcoat.2018.02.035
10.1016/j.vacuum.2018.08.011
10.1016/j.surfcoat.2013.03.003
10.1016/j.actamat.2012.09.058
10.1016/j.surfcoat.2017.06.050
10.1016/1359-6454(95)00382-7
10.1016/j.tafmec.2017.10.002
10.1016/S0921-5093(01)00932-7
10.1016/j.ceramint.2018.06.211
10.1016/0026-0657(95)80051-4
10.1016/j.vacuum.2019.04.055
10.1016/j.jallcom.2010.06.110
10.1016/j.matlet.2018.01.099
10.1016/j.jmapro.2020.03.049
10.1016/j.intermet.2007.03.010
10.1016/S1359-6462(00)00477-2
10.1016/S1003-6326(15)63748-7

10.1016/j.jmapro.2019.01.016
10.1016/j.calphad.2015.04.002
10.1016/j.ijsolstr.2013.09.015
10.1016/S1005-0302(10)60050-3
10.1016/j.surfcoat.2010.07.008
10.1016/j.jmatprotec.2008.08.019
10.1016/0921-5093(94)91073-1
10.1016/j.msea.2015.10.078
10.1016/j.triboint.2019.02.027
10.1016/j.jpowsour.2015.07.076
10.1016/j.jnucmat.2008.04.021
10.1016/S0921-5093(00)01470-2
10.1016/0956-716x(91)90487-1
10.1016/j.jallcom.2016.03.164
10.1016/j.ceramint.2019.07.279
10.1016/s1468-6996(01)00019-5
10.1016/S0026-0657(01)80524-5
10.1016/S1006-706X(08)60064-3
10.1016/j.actamat.2012.11.009
10.1016/j.msea.2011.01.044
10.1016/S0921-5093(01)01030-9
10.1016/j.jnoncrysol.2019.119576
10.1016/j.jallcom.2011.04.101
10.1016/j.ijmachtools.2007.02.001
10.1016/j.matdes.2014.09.062
10.1016/j.jallcom.2020.154455
10.1016/j.scriptamat.2018.09.017
10.1016/j.ijfatigue.2012.02.009
10.1016/0956-7151(92)90079-t
10.1016/0036-9748(82)90150-8
10.1016/0584-8547(78)80055-x
10.1016/j.optlastec.2016.06.008
10.1016/j.ijfatigue.2018.05.002
10.1016/j.promfg.2018.07.129
10.1016/j.actamat.2016.09.046
10.1016/j.jallcom.2018.03.397
10.1016/S1006-706X(10)60146-X
10.1016/j.measurement.2012.06.006
10.1016/S0043-1648(96)07298-5
10.1016/j.jmst.2019.05.006
10.1016/j.surfcoat.2017.06.012
10.1016/j.actamat.2016.11.018
10.1016/j.addma.2018.04.024
10.1016/j.surfcoat.2013.04.035
10.1016/j.tca.2018.02.012
10.1016/j.vacuum.2019.108863
10.1016/j.jallcom.2018.02.282
10.1016/j.scriptamat.2005.05.012
10.1016/j.prostr.2016.06.236
10.1016/j.msea.2014.01.056
10.1016/S1003-6326(11)60958-8
10.1016/j.ijfatigue.2017.08.028
10.1016/j.scriptamat.2006.10.026
10.1016/0921-5093(94)03296-3

10.1016/j.ijfatigue.2012.10.003
10.1016/j.vacuum.2017.04.030
10.1016/j.ijfatigue.2016.03.011
10.1016/j.jmmm.2007.03.210
10.1016/j.jallcom.2017.08.136
10.1016/0036-9748(89)90093-8
10.1016/j.msea.2014.01.101
10.1016/j.msea.2006.11.161
10.1016/S0584-8547(96)01570-4
10.1016/1359-6462(96)00211-4
10.1016/j.msea.2017.05.018
10.1016/S0167-6636(99)00016-2
10.1016/j.electacta.2010.09.072
10.1016/j.surfcoat.2006.07.243
10.1016/0001-6160(84)90097-x
10.1016/S0921-5093(02)00172-7
10.1016/S0921-5093(98)01018-1
10.1016/j.scriptamat.2013.09.016
10.1016/j.jallcom.2019.152382
10.1016/j.surfcoat.2018.12.089
10.1016/j.matchar.2008.03.017
10.1016/j.msea.2010.03.053
10.1016/j.vacuum.2018.03.048
10.1016/j.jallcom.2017.10.280
10.1016/S1359-6454(02)00436-6
10.1016/j.scriptamat.2015.05.016
10.1016/S1359-6454(00)00292-5
10.1016/j.actamat.2004.10.020
10.1016/j.msea.2005.10.019
10.1016/S1003-6326(11)61598-7
10.1016/j.msea.2019.138081
10.1016/j.matchar.2014.11.035
10.1016/0921-5093(91)90702-o
10.1016/S0022-3093(01)00813-4
10.1016/S1359-6454(00)00081-1
10.1016/S0022-3115(03)00076-X
10.1016/0257-8972(91)90030-z
10.1016/j.ijfatigue.2015.02.018
10.1016/S0921-5093(01)01357-0
10.1016/j.msea.2020.139255
10.1016/j.ijsolstr.2009.04.006
10.1016/j.jmatprotec.2012.10.002
10.1016/S0924-0136(02)00337-0
10.1016/j.ceramint.2007.03.008
10.1016/j.msea.2019.02.006
10.1016/0142-1123(96)89715-5
10.1016/j.actamat.2019.11.035
10.1016/j.surfcoat.2011.06.043
10.1016/j.mtla.2018.11.004
10.1016/j.vacuum.2019.109038
10.1016/j.msea.2016.10.086
10.1016/j.jallcom.2011.03.067
10.1016/0022-3115(90)90008-b
10.1016/S1003-6326(16)64215-2

10.1016/j.optlastec.2020.106117
10.1016/j.jallcom.2019.05.050
10.1016/j.surfcoat.2006.07.259
10.1016/j.ijmecsci.2018.06.023
10.1016/j.optlastec.2012.06.028
10.1016/j.actamat.2009.11.024
10.1016/j.msea.2018.04.114
10.1016/0956-716x(94)90434-0
10.1016/j.surfcoat.2007.02.015
10.1016/S1359-6454(03)00233-7
10.1016/j.jmst.2019.05.072
10.1016/S0257-8972(97)00467-2
10.1016/j.msea.2005.10.062
10.1016/j.corsci.2013.05.016
10.1016/j.matdes.2019.107796
10.1016/j.surfcoat.2014.07.052
10.1016/j.jmatprotec.2007.04.052
10.1016/j.wear.2014.11.027
10.1016/j.surfcoat.2016.09.049
10.1016/j.actamat.2016.08.068
10.1016/j.matchar.2019.109798
10.1016/j.jmps.2016.11.014
10.1016/S1359-6454(03)00355-0
10.1016/0040-6090(79)90526-1
10.1016/0001-6160(84)90224-4
10.1016/S1000-9361(11)60287-2
10.1016/j.matdes.2016.04.076
10.1016/S0921-5093(00)00763-2
10.1016/j.triboint.2018.10.050
10.1016/0036-9748(82)90460-4
10.1016/j.scriptamat.2014.11.026
10.1016/j.acme.2018.02.001
10.1016/j.matchar.2010.04.008
10.1016/S0921-5093(03)00628-2
10.1016/j.ijfatigue.2010.04.008
10.1016/j.ijfatigue.2018.08.033
10.1016/j.actamat.2019.10.033
10.1016/S0921-5093(01)00937-6
10.1016/j.rcim.2009.10.002
10.1016/0956-716x(92)90648-x
10.1016/j.corsci.2019.01.013
10.1016/S1003-6326(14)63248-9
10.1016/j.actamat.2007.01.033
10.1016/j.msea.2006.07.048
10.1016/j.actamat.2004.10.019
10.1016/j.actamat.2015.09.006
10.1016/j.nimb.2019.05.053
10.1016/0036-9748(86)90493-x
10.1016/j.corsci.2020.108634
10.1016/j.surfcoat.2008.05.013
10.1016/j.jmatprotec.2014.10.021
10.1016/j.jallcom.2017.08.137
10.1016/j.rinp.2018.12.046
10.1016/j.msea.2013.09.076

10.1016/j.commatsci.2019.03.004
10.1016/S0921-5093(02)00839-0
10.1016/j.precisioneng.2019.03.013
10.1016/j.msea.2018.11.028
10.1016/j.jallcom.2019.06.387
10.1016/j.matdes.2016.02.051
10.1016/j.commatsci.2013.12.026
10.1016/j.msea.2012.12.069
10.1016/j.jmst.2018.03.013
10.1016/j.msea.2014.12.085
10.1016/j.ndteint.2012.03.009
10.1016/j.msea.2003.09.020
10.1016/j.msea.2017.05.108
10.1016/j.ijsolstr.2004.09.037
10.1016/j.engfailanal.2019.04.023
10.1016/S1003-6326(13)62668-0
10.1016/j.jallcom.2018.03.167
10.1016/0026-0800(69)90038-x
10.1016/j.commatsci.2017.09.043
10.1016/0001-6160(86)90114-8
10.1016/j.ijfatigue.2008.03.019
10.1016/S0921-5093(99)00153-7
10.1016/j.jallcom.2014.05.085
10.1016/j.jallcom.2014.09.036
10.1016/j.msea.2014.11.026
10.1016/j.ultramic.2019.04.015
10.1016/j.scriptamat.2009.10.038
10.1016/j.surfcoat.2012.07.003
10.1016/j.msea.2010.07.062
10.1016/j.ijsolstr.2019.09.001
10.1016/j.surfcoat.2006.04.016
10.1016/j.matdes.2011.10.020
10.1016/S0010-938X(00)00137-2
10.1016/j.matchemphys.2014.11.005
10.1016/j.intermet.2019.106584
10.1016/j.surfcoat.2014.06.073
10.1016/0013-7944(82)90155-2
10.1016/j.cplett.2014.09.011
10.1016/j.matdes.2020.108529
10.1016/j.msea.2016.07.116
10.1016/0010-938x(89)90106-6
10.1016/j.matlet.2015.03.007
10.1016/S0022-0248(99)00864-7
10.1016/j.wear.2005.02.098
10.1016/j.ijhydene.2017.02.149
10.1016/j.ijheatmasstransfer.2004.03.006
10.1016/j.surfcoat.2017.01.031
10.1016/j.ijfatigue.2017.01.019
10.1016/j.apsusc.2018.01.226
10.1016/S0749-6419(01)00030-4
10.1016/j.msea.2019.01.109
10.1016/j.apsusc.2008.11.043
10.1016/j.msea.2004.03.057
10.1016/j.scriptamat.2018.05.025

10.1016/j.jallcom.2003.11.168
10.1016/j.jpowsour.2007.10.002
10.1016/j.rinp.2018.09.047
10.1016/S1359-6462(97)00018-3
10.1016/j.promfg.2016.08.004
10.1016/j.jallcom.2013.12.070
10.1016/j.ultramic.2014.10.010
10.1016/j.msea.2016.03.065
10.1016/S0008-6223(00)00112-3
10.1016/j.jallcom.2004.02.011
10.1016/S0924-0136(01)00855-X
10.1016/S0921-5093(02)00901-2
10.1016/j.matlet.2016.04.020
10.1016/j.msea.2018.10.013
10.1016/j.jallcom.2019.153301
10.1016/j.msec.2014.09.027
10.1016/j.corsci.2020.108431
10.1016/j.surfcoat.2004.04.094
10.1016/j.engfracmech.2018.05.048
10.1016/S0921-5093(00)01642-7
10.1016/j.measurement.2010.01.001
10.1016/S0921-5093(03)00245-4
10.1016/j.msea.2006.06.061
10.1016/j.matdes.2013.12.060
10.1016/j.ijheatmasstransfer.2018.08.105
10.1016/j.engfailanal.2019.104173
10.1016/j.matchar.2016.12.018
10.1016/j.msea.2004.09.062
10.1016/0010-938x(87)90042-4
10.1016/j.corsci.2010.03.020
10.1016/j.msea.2006.12.041
10.1016/0261-3069(95)90073-X
10.1016/S1359-6462(02)00436-0
10.1016/j.commatsci.2018.03.010
10.1016/j.proeng.2011.04.034
10.1016/j.jallcom.2008.10.117
10.1016/S0007-8506(07)63041-8
10.1016/j.msea.2018.01.051
10.1016/0364-5916(91)90013-a
10.1016/j.surfcoat.2019.06.020
10.1016/j.jmapro.2019.05.024
10.1016/0036-9748(87)90127-x
10.1016/B978-044482548-3/50052-4
10.1016/S0257-8972(96)03027-7
10.1016/j.actamat.2017.01.013
10.1016/S0167-8442(00)00016-1
10.1016/j.matdes.2016.01.032
10.1016/j.surfcoat.2013.10.066
10.1016/S0045-7825(97)00248-X
10.1016/j.matchar.2009.01.024
10.1016/j.intermet.2007.09.007
10.1016/j.msea.2018.08.096
10.1016/0142-1123(83)90056-7
10.1016/S1003-6326(15)64043-2

10.1016/j.matdes.2016.12.071
10.1016/j.triboint.2019.106080
10.1016/j.jallcom.2013.07.006
10.1016/j.msea.2019.138862
10.1016/j.ijfatigue.2015.07.005
10.1016/j.commatsci.2008.10.014
10.1016/j.jmapro.2018.11.016
10.1016/j.matpr.2015.08.039
10.1016/j.ijfatigue.2019.105392
10.1016/0001-6160(89)90191-0
10.1016/S0167-7322(99)00125-7
10.1016/S0167-577X(03)00423-3
10.1016/0036-9748(82)90237-x
10.1016/0956-7151(94)00337-H
10.1016/j.msea.2017.04.040
10.1016/j.msea.2009.03.090
10.1016/0142-1123(96)89690-3
10.1016/j.jmatprotec.2015.07.004
10.1016/j.actamat.2019.07.018
10.1016/S1359-6454(00)00390-6
10.1016/j.jmatprotec.2014.08.025
10.1016/j.msea.2012.04.066
10.1016/0956-716x(94)90204-6
10.1016/j.ijfatigue.2018.09.024
10.1016/j.ijfatigue.2018.07.018
10.1016/S0921-5093(01)00935-2
10.1016/j.msea.2019.03.083
10.1016/j.commatsci.2010.07.009
10.1016/S0257-8972(96)02947-7
10.1016/S0921-5093(00)02033-5
10.1016/j.asej.2018.02.001
10.1016/j.jallcom.2013.01.045
10.1016/j.msea.2007.08.083
10.1016/0010-938x(85)90061-7
10.1016/j.ijplas.2017.08.008
10.1016/j.matlet.2018.07.124
10.1016/j.corsci.2019.108171
10.1016/j.msea.2015.10.030
10.1016/S0921-5093(98)00516-4
10.1016/j.matchar.2015.06.029
10.1016/j.matdes.2018.06.024
10.1016/j.jallcom.2014.11.014
10.1016/j.jallcom.2018.09.263
10.1016/j.intermet.2014.06.004
10.1016/0022-3115(93)90037-y
10.1016/j.corsci.2019.01.003
10.1016/j.matlet.2017.12.003
10.1016/j.matchar.2011.09.006
10.1016/S0142-1123(03)00139-7
10.1016/0956-716x(94)90325-5
10.1016/0022-3115(91)90499-w
10.1016/S0142-1123(01)00131-1
10.1016/j.jallcom.2007.06.066
10.1016/j.jallcom.2019.152295

10.1016/j.matdes.2013.06.004
10.1016/j.msea.2010.01.045
10.1016/j.matpr.2019.08.159
10.1016/j.scriptamat.2017.07.023
10.1016/j.msea.2013.07.053
10.1016/j.msea.2007.01.117
10.1016/j.msea.2004.02.084
10.1016/j.ceramint.2015.10.119
10.1016/j.msea.2018.09.058
10.1016/j.jallcom.2018.11.137
10.1016/j.corsci.2017.01.011
10.1016/j.jnucmat.2012.08.040
10.1016/j.msea.2011.03.085
10.1016/S0925-8388(01)02028-X
10.1016/j.jmst.2015.11.018
10.1016/j.ceramint.2016.05.062
10.1016/S0927-0256(97)00077-3
10.1016/j.actamat.2007.08.006
10.1016/j.actamat.2017.07.044
10.1016/S1359-6462(03)00437-8
10.1016/S0921-5093(00)01829-3
10.1016/j.actamat.2003.08.014
10.1016/0921-5093(90)90147-u
10.1016/j.jallcom.2020.154583
10.1016/j.jmatprotec.2018.10.008
10.1016/0043-1648(92)90210-y
10.1016/j.surfcoat.2006.03.004
10.1016/S1003-6326(15)63582-8
10.1016/j.commatsci.2008.03.022
10.1016/0001-6160(86)90062-3
10.1016/j.msea.2006.11.016
10.1016/j.matlet.2007.04.082
10.1016/j.jcrysgro.2018.10.014
10.1016/j.actamat.2018.06.034
10.1016/j.commatsci.2017.09.061
10.1016/j.msea.2006.11.027
10.1016/j.intermet.2015.04.009
10.1016/j.actamat.2019.07.048
10.1016/j.cad.2007.11.003
10.1016/S0966-9795(97)00094-0
10.1016/j.engfailanal.2015.10.019
10.1016/0308-9126(81)90005-5
10.1016/S1003-6326(15)63832-8
10.1016/0956-7151(94)90411-1
10.1016/j.msea.2008.10.050
10.1016/j.msea.2019.138421
10.1016/S0257-8972(97)00069-8
10.1016/S1359-6462(02)00446-3
10.1016/j.matchar.2010.09.011
10.1016/j.matdes.2017.05.066
10.1016/j.surfcoat.2019.125107
10.1016/j.scriptamat.2006.06.019
10.1016/S0257-8972(01)01362-7
10.1016/j.msea.2011.10.014

10.1016/j.pnsc.2016.03.007
10.1016/S0921-5093(00)02035-9
10.1016/j.ijimpeng.2008.02.003
10.1016/j.surfcoat.2018.10.090
10.1016/j.apsusc.2015.10.096
10.1016/j.engfailanal.2018.06.007
10.1016/0040-6090(81)90007-9
10.1016/0921-5093(94)90472-3
10.1016/j.msea.2018.09.088
10.1016/j.jallcom.2019.07.204
10.1016/0026-0800(89)90033-5
10.1016/j.anucene.2012.08.020
10.1016/S0921-5093(01)01063-2
10.1016/j.matdes.2011.11.067
10.1016/j.apsusc.2016.02.162
10.1016/j.surfcoat.2008.04.070
10.1016/j.matpr.2018.06.148
10.1016/j.engfailanal.2017.12.001
10.1016/j.actamat.2018.01.055
10.1016/0956-7151(90)90036-g
10.1016/j.ijfatigue.2010.01.012
10.1016/0032-5910(78)80018-7
10.1016/j.msea.2006.05.152
10.1016/j.engfailanal.2005.07.022
10.1016/j.triboint.2010.02.008
10.1016/j.pnsc.2018.01.008
10.1016/S0142-1123(01)00049-4
10.1016/0921-5093(94)90366-2
10.1016/j.matchar.2015.01.003
10.1016/S1006-706X(13)60222-8
10.1016/j.scriptamat.2016.08.013
10.1016/0956-716x(92)90580-8
10.1016/j.wear.2013.01.103
10.1016/j.procir.2014.03.061
10.1016/j.solmat.2018.04.025
10.1016/S0257-8972(96)03014-9
10.1016/j.jmapro.2019.11.005
10.1016/j.intermet.2010.01.011
10.1016/j.chemgeo.2016.11.024
10.1016/j.tafmec.2016.03.007
10.1016/j.scriptamat.2004.12.006
10.1016/j.corsci.2017.04.015
10.1016/j.jallcom.2012.04.093
10.1016/j.ijfatigue.2014.02.023
10.1016/j.ijfatigue.2015.04.006
10.1016/j.msea.2012.02.088
10.1016/j.jmst.2013.11.013
10.1016/j.msea.2018.11.052
10.1016/j.jallcom.2019.03.298
10.1016/0010-938x(93)90249-g
10.1016/S0167-8442(96)00038-9
10.1016/S0921-4526(96)01244-6
10.1016/j.ultramic.2011.01.015
10.1016/j.surfcoat.2006.01.034

10.1016/S1359-6462(99)00284-5
10.1016/j.scriptamat.2017.04.018
10.1016/j.jallcom.2009.08.142
10.1016/j.msea.2009.11.008
10.1016/0257-8972(87)90095-8
10.1016/j.msea.2016.11.060
10.1016/j.ceramint.2015.05.119
10.1016/0956-7151(91)90293-a
10.1016/j.acme.2018.01.015
10.1016/j.msea.2006.07.053
10.1016/j.scriptamat.2008.03.021
10.1016/j.msea.2018.10.112
10.1016/j.msea.2016.09.081
10.1016/j.jallcom.2017.12.337
10.1016/j.matdes.2011.10.013
10.1016/j.msea.2007.07.038
10.1016/S0925-8388(00)00583-1
10.1016/0026-0800(82)90042-8
10.1016/0921-5093(91)90703-p
10.1016/J.ENG.2017.05.012
10.1016/j.apsusc.2010.12.097
10.1016/j.matchemphys.2012.01.086
10.1016/j.jallcom.2018.08.189
10.1016/j.msea.2013.11.037
10.1016/0921-5093(89)90713-2
10.1016/0029-1021(75)90042-0
10.1016/0921-5093(92)90099-m
10.1016/j.matchar.2018.10.031
10.1016/j.jmst.2019.11.028
10.1016/j.calphad.2019.03.013
10.1016/j.cja.2017.08.019
10.1016/j.scriptamat.2008.06.032
10.1016/1359-6462(95)00547-1
10.1016/j.msea.2018.12.087
10.1016/j.matchar.2015.01.002
10.1016/0956-716x(91)90055-6
10.1016/j.ndteint.2010.05.012
10.1016/j.ijfatigue.2015.08.001
10.1016/j.matchar.2019.109916
10.1016/j.proeng.2017.10.789
10.1016/j.actamat.2015.08.009
10.1016/j.scriptamat.2011.01.027
10.1016/j.optlaseng.2019.105854
10.1016/j.surfcoat.2019.01.112
10.1007/s11434-016-1098-7
10.1016/s0036-9748(88)80191-1
10.1016/S1359-6462(02)00355-X
10.1016/S1359-6454(96)00192-9
10.1016/S0921-5093(00)01776-7
10.1016/j.jmatprotec.2007.11.089
10.1016/j.jallcom.2018.03.124
10.1016/j.jcrysgro.2013.07.028
10.1016/j.jmps.2018.09.010
10.1016/j.phpro.2016.08.073

10.1016/j.jclepro.2016.04.070
10.1016/j.msea.2018.02.063
10.1016/j.actamat.2015.04.061
10.1016/0956-716x(90)90530-t
10.1016/0025-5416(74)90135-9
10.1016/0141-5425(88)90027-1
10.1016/j.jmst.2019.11.023
10.1016/j.jallcom.2017.11.327
10.1016/j.jallcom.2015.01.109
10.1016/j.scriptamat.2016.12.037
10.1016/j.matdes.2017.11.033
10.1016/S0921-5093(97)00472-3
10.1016/j.ijfatigue.2020.105594
10.1016/S1359-6454(03)00172-1
10.1016/j.eng.2019.03.014
10.1016/j.jnucmat.2013.09.030
10.1016/j.ijfatigue.2018.08.002
10.1016/S0257-8972(98)00667-7
10.1016/0036-9748(87)90299-7
10.1016/j.msea.2015.02.014
10.1016/0036-9748(83)90438-6
10.1016/S0925-8388(00)01221-4
10.1016/j.msea.2008.07.021
10.1016/j.intermet.2004.07.020
10.1533/9780857095169.1.142
10.1016/s1468-6996(01)00044-4
10.1016/j.msea.2018.11.040
10.1016/j.calphad.2015.10.011
10.1016/j.jmatprotec.2010.12.013
10.1016/0921-4526(95)00290-P
10.1016/j.apsusc.2013.06.153
10.1016/0956-716x(93)90265-t
10.1016/j.jeurceramsoc.2020.01.053
10.1016/S0142-1123(99)00009-2
10.1016/j.surfcoat.2010.07.108
10.1016/j.engfailanal.2008.07.013
10.1016/j.intermet.2010.10.018
10.1016/j.msea.2019.138412
10.1016/j.jnucmat.2010.01.019
10.1016/j.actamat.2008.12.030
10.1016/j.actamat.2016.01.032
10.1016/0749-6419(90)90037-f
10.1016/j.surfcoat.2018.12.050
10.1016/0022-5096(90)90020-5
10.1016/0308-0161(95)00097-6
10.1016/j.msea.2010.04.071
10.1016/j.msea.2017.11.125
10.1016/j.scriptamat.2014.07.010
10.1016/j.intermet.2009.02.008
10.1016/0142-1123(86)90008-3
10.1016/j.msea.2016.09.060
10.1016/S1044-5803(99)00042-X
10.1016/0025-5416(86)90232-6
10.1016/j.jallcom.2020.155019

10.1016/0956-716x(94)90285-2
10.1016/j.matlet.2004.05.006
10.1016/j.ijfatigue.2019.04.024
10.1016/0036-9748(80)90098-8
10.1016/j.msea.2019.138879
10.1016/S0924-0136(01)00850-0
10.1016/j.scriptamat.2006.11.002
10.1016/j.scriptamat.2006.03.054
10.1016/j.msea.2016.09.056
10.1016/j.ijpvp.2008.02.004
10.1016/j.actamat.2007.01.034
10.1016/0921-5093(95)09980-8
10.1016/j.corsci.2013.05.013
10.1016/j.ijfatigue.2016.10.011
10.1016/j.msea.2013.07.034
10.1016/0036-9748(86)90465-5
10.1016/0040-6090(90)90246-a
10.1016/0142-1123(93)90376-2
10.1016/s0151-9107(02)85003-6
10.1016/j.jmatprotec.2007.11.176
10.1016/j.corsci.2015.05.018
10.1016/0003-2670(95)00215-L
10.1016/j.surfcoat.2015.06.018
10.1016/j.msea.2010.07.035
10.1016/j.ijfatigue.2010.11.010
10.1016/j.scriptamat.2013.02.025
10.1016/j.ijfatigue.2011.05.018
10.1016/j.jmapro.2020.01.049
10.1016/j.ceramint.2008.07.005
10.1016/j.matdes.2014.05.020
10.1016/S0921-5093(98)01025-9
10.1016/j.ijhydene.2010.12.095
10.1016/0040-6090(83)90305-x
10.1016/S1006-706X(15)30110-2
10.1016/0025-5416(87)90487-3
10.1016/j.triboint.2019.105998
10.1016/j.ijpvp.2019.05.005
10.1016/S0921-5093(97)00373-0
10.1016/j.msea.2011.02.063
10.1016/0022-3115(94)90338-7
10.1016/j.actamat.2020.02.034
10.1016/j.vacuum.2018.08.020
10.1016/S0921-5093(01)00950-9
10.1016/S1359-6454(02)00265-3
10.1016/j.msea.2018.07.008
10.1016/0025-5416(72)90072-9
10.1016/0013-7944(89)90239-7
10.1016/0010-938x(93)90304-y
10.1016/0956-716x(94)90583-5
10.1016/j.intermet.2012.01.020
10.1016/j.actamat.2016.11.053
10.1016/j.actamat.2016.07.055
10.1016/S0142-1123(01)00109-8
10.1016/j.intermet.2018.10.027

10.1016/0167-8442(96)00015-8
10.1016/j.intermet.2013.09.010
10.1016/j.msea.2016.10.058
10.1016/j.ultras.2018.05.007
10.1016/j.jallcom.2017.03.358
10.1016/j.vacuum.2019.108979
10.1016/j.surfcoat.2013.01.033
10.1016/j.ceramint.2017.03.156
10.1016/S0921-5093(98)01181-2
10.1016/j.optlaseng.2017.03.004
10.1016/S1003-6326(09)60382-4
10.1016/j.actamat.2011.02.006
10.1016/j.msea.2008.04.100
10.1016/0022-3115(96)00353-4
10.1016/j.matdes.2019.107936
10.1016/j.ijmachtools.2017.07.005
10.1016/j.matdes.2004.07.025
10.1016/S0167-577X(02)00613-4
10.1016/0036-9748(84)90425-3
10.1016/0956-7151(94)90415-4
10.1016/j.matdes.2016.04.051
10.1016/j.intermet.2018.07.007
10.1016/j.jallcom.2019.05.085
10.1016/j.msea.2014.08.054
10.1016/j.jallcom.2017.02.002
10.1016/j.actamat.2008.07.024
10.1016/j.msea.2016.08.061
10.1016/j.msea.2003.10.226
10.1016/j.msea.2006.11.054
10.1016/0921-5093(93)90247-c
10.1016/j.mechmat.2019.02.009
10.1016/S1002-0071(12)60064-1
10.1016/j.jmst.2015.01.002
10.1016/j.triboint.2007.10.004
10.1016/j.actamat.2003.10.001
10.1016/j.scriptamat.2006.06.012
10.1016/j.commatsci.2017.08.005
10.1016/j.ijplas.2003.07.002
10.1016/j.msea.2011.10.124
10.1016/j.intermet.2005.11.024
10.1016/j.msea.2019.138038
10.1016/j.jallcom.2019.04.067
10.1016/j.jallcom.2015.04.095
10.1016/S0257-8972(02)00596-0
10.1016/j.msec.2017.02.046
10.1016/S1359-6462(97)00291-1
10.1016/j.msea.2004.08.072
10.1016/0956-7151(93)90132-c
10.1016/j.commatsci.2012.10.037
10.1016/j.actamat.2019.05.063
10.1016/j.surfcoat.2008.01.023
10.1016/j.ijfatigue.2016.06.006
10.1016/j.jclepro.2014.12.020
10.1016/j.ijfatigue.2012.08.012

10.1016/j.ijfatigue.2018.07.029
10.1016/j.engfracmech.2008.09.003
10.1016/j.engfailanal.2016.05.024
10.1016/0001-6160(86)90135-5
10.1016/j.surfcoat.2017.05.019
10.1016/S0043-1648(01)00885-7
10.1016/j.ultramic.2018.08.025
10.1016/j.jmst.2016.11.009
10.1016/j.msea.2009.03.086
10.1016/j.jallcom.2018.01.325
10.1016/j.matchar.2018.03.008
10.1016/j.corsci.2017.08.025
10.1016/j.vacuum.2019.109009
10.1016/j.scriptamat.2019.05.002
10.1016/j.intermet.2010.07.001
10.1016/j.jallcom.2013.03.057
10.1016/j.surfcoat.2014.01.025
10.1016/j.jallcom.2018.08.089
10.1016/0025-5416(86)90236-3
10.1016/j.actamat.2014.08.048
10.1016/j.msea.2007.06.064
10.1016/j.mechmat.2017.03.011
10.1016/j.cherd.2018.05.040
10.1016/j.pnsc.2012.07.005
10.1016/j.msea.2005.11.028
10.1016/j.msea.2020.139007
10.1016/j.apsusc.2012.10.002
10.1016/j.surfcoat.2019.124937
10.1016/j.measurement.2014.11.021
10.1016/j.calphad.2013.09.003
10.1016/j.corsci.2007.05.009
10.1016/j.msea.2019.138405
10.1016/j.jmst.2017.03.017
10.1016/j.msea.2018.11.092
10.1016/j.jeurceramsoc.2008.07.026
10.1016/0020-7403(91)90005-n
10.1016/j.matdes.2017.01.069
10.1016/j.jmst.2019.04.011
10.1016/j.surfcoat.2019.02.048
10.1016/j.jallcom.2016.06.185
10.1016/j.corsci.2014.10.042
10.1016/j.ijfatigue.2019.02.047
10.1016/S0010-938X(98)00175-9
10.1016/j.actamat.2018.07.034
10.1016/j.jallcom.2017.06.168
10.1016/j.msea.2017.12.049
10.1016/j.msea.2016.01.111
10.1016/S0257-8972(97)00480-5
10.1016/j.jallcom.2017.01.290
10.1016/j.ultras.2014.10.009
10.1016/S0142-1123(96)00072-2
10.1016/j.ijfatigue.2018.04.018
10.1016/j.actamat.2018.07.007
10.1016/j.matchar.2011.06.002

10.1016/S0257-8972(00)00890-2
10.1016/j.scriptamat.2005.08.038
10.1016/0921-5093(89)90823-x
10.1016/j.jmatprotec.2016.03.003
10.1016/0360-5442(91)90110-8
10.1016/j.jmapro.2017.10.027
10.1016/j.actamat.2010.04.008
10.1016/j.optlastec.2020.106222
10.1016/0956-716x(90)90314-7
10.1016/j.msea.2018.03.127
10.1016/S0921-5093(00)01053-4
10.1016/j.ijplas.2010.08.005
10.1016/j.intermet.2010.12.018
10.1016/j.prostr.2017.11.075
10.1016/j.mechmat.2020.103347
10.1016/j.jallcom.2006.01.126
10.1016/0025-5416(84)90161-7
10.1016/j.commatsci.2019.109279
10.1016/S0045-7825(01)00348-6
10.1016/0022-3115(90)90439-t
10.1016/j.msea.2006.07.133
10.1016/j.jmapro.2018.05.034
10.1016/j.jallcom.2016.03.140
10.1016/S0043-1648(02)00299-5
10.1016/j.msea.2019.138874
10.1016/S0142-1123(97)82658-8
10.1016/j.scriptamat.2004.06.013
10.1016/j.msea.2008.03.040
10.1016/j.ijmecsci.2017.05.036
10.1016/j.msea.2016.03.030
10.1016/j.matchar.2019.109998
10.1016/j.tafmec.2018.09.003
10.1016/j.optlastec.2018.03.020
10.1016/0895-7177(90)90317-G
10.1016/j.corsci.2019.108121
10.1016/0956-716x(93)90536-2
10.1016/j.msea.2008.02.019
10.1016/0257-8972(90)90145-3
10.1016/j.scriptamat.2020.02.010
10.1016/j.vacuum.2017.10.017
10.1016/S1359-6454(96)00402-8
10.1016/S0257-8972(96)03117-9
10.1016/j.jmapro.2017.04.024
10.1016/j.surfcoat.2010.08.111
10.1016/j.ijmachtools.2011.08.018
10.1016/S1006-706X(11)60054-X
10.1016/j.engfracmech.2006.10.021
10.1016/j.msea.2016.03.081
10.1016/S1359-6454(99)00404-8
10.1016/S1359-6454(97)00017-7
10.1016/j.apsusc.2014.08.017
10.1016/0036-9748(89)90468-7
10.1016/0025-5416(74)90202-x
10.1016/S1044-5803(98)00013-8

10.1016/S0921-5093(96)10507-4
10.1016/j.jmatprotec.2015.08.001
10.1016/j.actamat.2017.07.037
10.1016/j.msea.2018.10.063
10.1016/j.jmapro.2017.04.003
10.1016/j.precisioneng.2019.05.010
10.1016/0022-3115(93)90078-d
10.1016/j.matdes.2015.05.004
10.1016/0036-9748(87)90396-6
10.1016/0040-6090(77)90128-6
10.1016/0921-5093(94)03313-7
10.1016/S0921-5093(99)00012-X
10.1016/j.matlet.2007.08.032
10.1016/j.engfracmech.2004.02.002
10.1016/j.actamat.2014.05.040
10.1016/S1003-6326(18)64817-4
10.1016/j.matdes.2016.11.056
10.1016/j.actamat.2017.09.063
10.1016/j.surfcoat.2008.07.018
10.1016/0022-3115(85)90165-5
10.1016/j.msea.2011.07.053
10.1016/0956-716X(95)00446-3
10.1016/j.nucengdes.2013.11.045
10.1016/S1003-6326(16)64417-5
10.1016/j.ijmachtools.2009.07.002
10.1016/0025-5416(83)90199-4
10.1016/j.msea.2014.10.041
10.1016/j.corsci.2016.04.026
10.1016/0921-5093(90)90092-h
10.1016/j.matdes.2015.01.009
10.1016/j.ijplas.2017.05.006
10.1016/j.mtcomm.2020.100958
10.1016/S0010-938X(00)00011-1
10.1016/j.actamat.2007.12.021
10.1016/1044-5803(91)90040-b
10.1016/S0921-5093(01)01965-7
10.1016/j.scriptamat.2003.11.030
10.1016/j.msea.2013.05.064
10.1016/j.msea.2018.04.004
10.1016/j.jallcom.2019.151740
10.1016/j.jallcom.2014.09.195
10.1016/j.surfcoat.2018.10.091
10.1016/j.surfcoat.2013.10.059
10.1016/S0003-2670(01)01406-4
10.1016/j.proeng.2013.03.256
10.1016/j.corsci.2011.05.026
10.1016/j.matdes.2017.06.047
10.1016/j.surfcoat.2007.06.014
10.1016/S0925-8388(01)01905-3
10.1016/j.proeng.2011.12.542
10.1016/j.matlet.2008.10.047
10.1016/j.msea.2008.01.025
10.1016/j.optlaseng.2010.03.005
10.1016/j.surfcoat.2011.04.084

10.1016/S0257-8972(02)00595-9
10.1016/j.actamat.2005.10.009
10.1016/j.scriptamat.2017.03.007
10.1016/j.actamat.2008.07.015
10.1016/j.jnucmat.2013.04.056
10.1016/j.matdes.2018.10.038
10.1016/j.ijfatigue.2007.01.059
10.1016/j.matchar.2012.09.012
10.1016/j.corsci.2014.10.025
10.1016/j.msea.2009.09.016
10.1016/j.ijmachtools.2020.103529
10.1016/j.ijmecsci.2019.105277
10.1016/S0043-1648(01)00676-7
10.1016/j.jpowsour.2006.02.099
10.1016/0010-938x(85)90035-6
10.1016/j.corsci.2017.01.015
10.1016/S0966-9795(00)00081-9
10.1016/j.jmps.2016.05.016
10.1016/j.msea.2005.02.038
10.1016/0010-938x(88)90115-1
10.1016/S0020-7683(00)00309-7
10.1016/j.matchar.2015.09.011
10.1016/S0022-3115(01)00558-X
10.1016/0036-9748(85)90022-5
10.1016/j.commatsci.2014.12.035
10.1016/j.optlastec.2018.09.011
10.1016/j.msea.2007.05.082
10.1016/j.jallcom.2015.01.154
10.1016/j.actamat.2017.07.027
10.1016/j.corsci.2012.10.002
10.1016/0142-1123(83)90026-9
10.1016/j.wear.2019.01.085
10.1016/j.scriptamat.2016.04.029
10.1016/0257-8972(95)02448-4
10.1016/S0142-1123(01)00120-7
10.1016/j.jnucmat.2012.11.027
10.1016/j.jallcom.2017.03.070
10.1016/j.jallcom.2015.02.226
10.1016/j.corsci.2016.11.006
10.1016/j.ijfatigue.2019.105452
10.1016/j.apsusc.2014.11.126
10.1016/j.matpr.2019.07.257
10.1016/j.scriptamat.2012.06.023
10.1016/j.intermet.2004.07.041
10.1016/j.intermet.2004.07.032
10.1016/j.jallcom.2017.09.327
10.1016/j.jmrt.2018.03.006
10.1016/0142-1123(96)82906-9
10.1016/j.msea.2007.01.111
10.1016/j.matdes.2014.08.007
10.1016/j.msea.2006.05.030
10.1016/j.nimb.2008.09.023
10.1016/j.corsci.2015.04.017
10.1016/0001-6160(88)90241-6

10.1016/j.jmatprotec.2007.11.264
10.1016/j.surfcoat.2015.08.054
10.1016/j.matlet.2015.08.107
10.1016/j.jeurceramsoc.2016.02.011
10.1016/j.commatsci.2003.12.002
10.1016/j.scriptamat.2017.04.008
10.1016/j.ceramint.2016.04.018
10.1016/j.engfracmech.2017.07.027
10.1016/j.jallcom.2013.12.107
10.1016/j.msea.2005.12.030
10.1016/0921-5093(91)90690-o
10.1016/j.msea.2006.06.066
10.1016/j.surfcoat.2007.02.044
10.1016/S1006-706X(15)30046-7
10.1016/j.proeng.2010.03.201
10.1016/j.ijfatigue.2020.105579
10.1016/S0167-577X(99)00101-9
10.1016/j.msea.2013.07.037
10.1016/j.taml.2018.04.001
10.1016/j.msea.2019.138165
10.1016/j.matdes.2018.02.053
10.1016/j.jmatprotec.2017.09.011
10.1016/j.surfcoat.2018.05.059
10.1016/j.jmatprotec.2016.10.016
10.1016/j.msea.2019.138694
10.1016/j.engfracmech.2011.04.006
10.1016/j.optlastec.2018.08.018
10.1016/j.jallcom.2013.12.224
10.1016/j.jmst.2015.07.004
10.1016/S0257-8972(01)01483-9
10.1016/0956-716x(91)90410-3
10.1016/s1468-6996(01)00018-3
10.1016/j.jmst.2012.12.010
10.1016/j.matlet.2010.05.011
10.1016/j.msea.2018.11.139
10.1016/S0966-9795(01)00021-8
10.1016/j.prostr.2020.01.101
10.1016/j.matdes.2018.02.001
10.1016/j.engfailanal.2018.11.004
10.1016/j.actamat.2016.07.005
10.1016/j.engfailanal.2018.07.023
10.1016/S1003-6326(16)64303-0
10.1016/S1359-6462(98)00251-6
10.1016/S0266-8920(01)00014-5
10.1016/0921-5093(91)90273-p
10.1016/j.msea.2005.03.029
10.1016/j.prostr.2020.01.089
10.1016/j.intermet.2004.04.042
10.1016/j.surfcoat.2011.12.033
10.1016/j.corsci.2015.12.007
10.1016/S1006-706X(16)30164-9
10.1016/0921-5093(94)90995-4
10.1016/j.ijfatigue.2010.05.003
10.1016/j.calphad.2003.12.002

10.1016/S0142-1123(98)00056-5
10.1016/0001-6160(83)90062-7
10.1016/j.surfcoat.2015.12.046
10.1016/0921-5093(92)90358-8
10.1016/0956-716X(95)00266-X
10.1016/j.actamat.2015.07.048
10.1016/S1359-6454(98)00126-8
10.1016/S0013-7944(97)00137-9
10.1016/0142-1123(96)82740-X
10.1016/j.jallcom.2008.02.046
10.1016/S0043-1648(01)00889-4
10.1016/j.ast.2007.12.002
10.1016/0956-7151(90)90115-w
10.1016/j.jallcom.2017.10.230
10.1016/j.ijfatigue.2015.03.020
10.1016/j.ultras.2017.02.002
10.1016/j.scriptamat.2011.09.042
10.1016/0026-0657(95)91380-7
10.1016/j.surfcoat.2014.12.040
10.1016/j.surfcoat.2007.11.034
10.1016/j.intermet.2004.06.003
10.1016/0167-577x(94)90126-0
10.1016/j.commatsci.2013.04.005
10.1016/j.matlet.2015.12.104
10.1016/0045-7949(85)90187-7
10.1016/0921-5093(93)90421-a
10.1016/j.jeurceramsoc.2008.02.028
10.1016/j.ijplas.2019.02.015
10.1016/j.vacuum.2020.109310
10.1016/j.corsci.2016.09.010
10.1016/j.scriptamat.2019.06.033
10.1016/j.actamat.2009.01.027
10.1016/j.vacuum.2014.03.013
10.1016/S1359-6462(01)00953-8
10.1016/j.apsusc.2008.08.043
10.1016/j.surfcoat.2016.01.012
10.1016/j.jnucmat.2013.03.083
10.1016/S1006-706X(06)60032-0
10.1016/0956-7151(92)90300-4
10.1016/S0257-8972(02)00469-3
10.1016/S0013-7944(96)00057-4
10.1016/S0925-8388(03)00186-5
10.1016/j.ijhydene.2017.11.081
10.1016/j.jnucmat.2011.02.047
10.1016/0140-6701(95)95586-T
10.1016/j.ijfatigue.2018.10.012
10.1016/j.engfailanal.2014.01.019
10.1016/j.jallcom.2014.12.096
10.1016/0036-9748(89)90039-2
10.1016/0921-5093(89)90789-2
10.1016/0257-8972(87)90145-9
10.1016/j.jallcom.2013.09.020
10.1016/j.eswa.2008.12.054
10.1016/j.msea.2018.03.013

10.1016/S1359-6462(98)00124-9
10.1016/j.vacuum.2018.08.004
10.1016/j.engfracmech.2015.01.002
10.1016/j.jallcom.2011.07.057
10.1016/j.corsci.2013.08.003
10.1016/j.engfailanal.2014.06.003
10.1016/j.ceramint.2017.01.153
10.1016/S0169-4332(97)00003-2
10.1016/j.mprp.2016.12.058
10.1016/j.mtcomm.2019.100838
10.1016/j.actamat.2018.03.037
10.1016/j.matpr.2018.02.141
10.1016/j.corsci.2016.01.019
10.1016/S0921-5093(99)00236-1
10.1016/j.surfcoat.2019.125317
10.1016/S0921-5093(00)01793-7
10.1016/j.calphad.2018.11.001
10.1016/j.msea.2013.11.049
10.1016/j.jallcom.2019.152447
10.1016/j.corsci.2010.01.030
10.1016/j.wear.2017.02.025
10.1016/j.matlet.2015.02.084
10.1016/j.jallcom.2017.07.027
10.1016/j.msea.2005.10.056
10.1016/j.matdes.2005.12.008
10.1016/j.msea.2014.10.071
10.1016/j.matchar.2018.02.038
10.1016/j.surfcoat.2003.06.023
10.1016/j.commatsci.2007.02.008
10.1016/j.matdes.2016.06.062
10.1016/j.jallcom.2015.12.053
10.1016/S0266-3538(01)00161-0
10.1016/j.jallcom.2017.04.080
10.1016/S0921-5093(00)01941-9
10.1016/j.matdes.2015.10.027
10.1016/S0142-1123(99)00081-X
10.1016/j.calphad.2019.101649
10.1016/j.msea.2019.138646
10.1016/j.msea.2008.02.056
10.1016/j.actamat.2015.04.035
10.1016/j.jallcom.2018.08.032
10.1016/0040-6090(82)90578-8
10.1016/j.msea.2007.09.008
10.1016/j.msea.2014.06.118
10.1016/j.surfcoat.2012.08.100
10.1016/j.matchar.2017.06.019
10.1016/S1003-6326(10)60241-5
10.1016/j.ijfatigue.2020.105639
10.1016/S1000-9361(07)60020-X
10.1016/S0921-5093(97)00025-7
10.1016/j.msea.2018.08.072
10.1016/S0142-1123(01)00053-6
10.1016/0167-8442(91)90013-a
10.1016/0010-938x(87)90072-2

10.1016/j.corsci.2016.05.011
10.1016/j.matchar.2017.05.032
10.1016/j.jclepro.2016.04.074
10.1016/j.matchemphys.2014.09.047
10.1016/j.jallcom.2014.09.012
10.1016/j.msea.2016.03.079
10.1016/j.jallcom.2019.153325
10.1016/S1359-6454(99)00355-9
10.1016/0040-6090(82)90580-6
10.1016/j.corsci.2004.08.009
10.1016/j.jallcom.2018.07.162
10.1016/j.msea.2017.12.086
10.1016/j.actamat.2009.08.002
10.1016/0266-3538(94)90201-1
10.1016/s1468-6996(01)00070-5
10.1016/j.ijfatigue.2012.09.014
10.1016/j.msea.2012.04.109
10.1016/j.powtec.2020.04.010
10.1016/S1359-6454(99)00473-5
10.1016/j.scriptamat.2016.10.013
10.1016/S0142-1123(03)00170-1
10.1016/j.surfcoat.2003.09.066
10.1016/j.ijsolstr.2016.06.021
10.1016/0921-5093(93)90456-o
10.1016/j.mprp.2016.06.002
10.1016/j.mtcomm.2019.100631
10.1016/j.ceramint.2015.10.117
10.1016/j.cattod.2006.07.056
10.1016/j.scriptamat.2015.01.014
10.1016/j.cirp.2011.03.094
10.1016/j.surfcoat.2013.01.021
10.1016/S0749-6419(00)00032-2
10.1016/1359-6462(95)00510-2
10.1016/j.mprp.2016.10.065
10.1016/j.actamat.2019.10.019
10.1016/j.msea.2017.09.087
10.1016/j.jallcom.2019.02.055
10.1016/j.optlaseng.2015.03.009
10.1016/S0143-8166(99)00031-7
10.1016/j.jpowsour.2005.09.065
10.1016/j.corsci.2018.11.026
10.1016/S1566-1369(03)80005-5
10.1016/j.actamat.2012.01.042
10.1016/j.jmapro.2018.04.005
10.1016/j.actamat.2016.09.005
10.1016/j.procir.2018.08.279
10.1016/j.actamat.2012.10.012
10.1016/j.matchar.2017.06.017
10.1016/j.msea.2011.03.034
10.1016/j.prostr.2020.01.031
10.1016/j.scriptamat.2005.09.007
10.1016/0956-716x(93)90181-q
10.1016/S1006-706X(11)60013-7
10.1016/j.apsusc.2010.05.098

10.1016/S0963-8695(98)00069-3
10.1016/0036-9748(82)90284-8
10.1016/j.surfcoat.2019.05.001
10.1016/S0257-8972(03)00767-9
10.1016/j.intermet.2012.07.010
10.1016/j.msea.2017.08.118
10.1016/S0921-5093(97)00753-3
10.1016/S0022-3115(02)00796-1
10.1016/j.intermet.2011.04.011
10.1016/j.jmatprotec.2006.11.002
10.1016/j.measurement.2013.11.018
10.1016/j.procir.2018.09.002
10.1016/j.surfcoat.2004.10.056
10.1016/0956-716X(95)00115-C
10.1016/j.actamat.2017.05.074
10.1016/j.ijpvp.2007.06.005
10.1016/j.surfcoat.2008.06.094
10.1016/0025-5416(87)90366-1
10.1016/j.jallcom.2016.12.325
10.1016/j.msea.2012.01.059
10.1016/j.actamat.2007.06.005
10.1016/j.jpowsour.2005.07.086
10.1016/0001-6160(83)90202-x
10.1016/j.msea.2006.05.032
10.1016/S1359-6454(97)00116-X
10.1016/j.probengmech.2013.08.004
10.1016/j.actamat.2004.11.038
10.1016/j.jallcom.2011.12.082
10.1016/j.msea.2014.03.074
10.1016/S0167-6636(01)00071-0
10.1016/0001-6160(87)90068-x
10.1016/S1359-6462(97)00156-5
10.1016/S1369-7021(07)70022-6
10.1016/0956-716X(95)00325-P
10.1016/j.ijmecsci.2018.07.033
10.1016/j.surfcoat.2003.08.071
10.1016/j.jallcom.2016.06.120
10.1016/j.tsf.2011.01.047
10.1016/j.matchar.2017.02.016
10.1016/j.apsusc.2015.01.033
10.1016/j.matchar.2017.07.024
10.1016/0022-3115(84)90132-6
10.1016/j.intermet.2011.06.020
10.1016/j.jallcom.2016.09.007
10.1016/j.msea.2015.06.070
10.1016/j.cirp.2015.05.004
10.1016/j.intermet.2019.106543
10.1016/j.actamat.2014.07.002
10.1016/j.surfcoat.2003.12.003
10.1016/j.jallcom.2019.03.286
10.1016/j.surfcoat.2016.10.014
10.1016/j.matpr.2019.12.370
10.1016/j.scriptamat.2014.09.010
10.1016/j.actamat.2012.04.002

10.1016/0026-0657(00)92882-0
10.1016/j.scriptamat.2005.11.049
10.1016/0921-5093(94)09589-0
10.1016/j.msea.2014.06.095
10.1016/j.msea.2015.06.065
10.1016/j.msea.2004.11.017
10.1016/S0257-8972(00)00724-6
10.1016/j.jallcom.2017.10.076
10.1016/j.ndteint.2016.05.004
10.1016/0026-0800(88)90006-7
10.1016/j.jestch.2016.07.003
10.1016/j.matdes.2008.07.011
10.1016/j.scriptamat.2009.03.008
10.1016/j.vacuum.2018.07.034
10.1016/0956-716x(94)90554-1
10.1016/j.matlet.2015.01.089
10.1016/j.surfcoat.2015.08.067
10.1016/j.corsci.2015.12.016
10.1016/j.optlastec.2018.05.003
10.1016/j.actamat.2011.10.033
10.1016/j.actamat.2018.09.049
10.1016/S1003-6326(11)61435-0
10.1016/j.msea.2006.10.035
10.1016/j.electacta.2007.05.043
10.1016/j.surfcoat.2017.12.065
10.1016/j.actamat.2013.07.028
10.1016/0257-8972(87)90194-0
10.1016/j.cirp.2012.03.100
10.1016/j.apsusc.2020.145936
10.1016/j.ijfatigue.2017.04.003
10.1016/j.jallcom.2011.02.156
10.1016/j.ijfatigue.2007.01.051
10.1016/j.apsusc.2016.01.088
10.1016/j.wear.2015.07.010
10.1016/j.ceramint.2012.05.047
10.1016/S0921-5093(97)80019-6
10.1016/j.powtec.2019.07.011
10.1016/j.msea.2015.05.102
10.1016/j.scriptamat.2019.02.038
10.1016/0142-9612(94)90239-9
10.1016/j.intermet.2014.07.021
10.1016/S0966-9795(03)00104-3
10.1016/S0013-4686(03)00006-9
10.1016/j.optlastec.2017.01.016
10.1016/j.prostr.2020.01.085
10.1016/j.actamat.2015.01.063
10.1016/j.matdes.2014.11.002
10.1016/j.actamat.2012.08.032
10.1016/j.scriptamat.2007.07.001
10.1016/j.vacuum.2020.109284
10.1016/j.matdes.2019.108445
10.1016/j.apsusc.2015.10.023
10.1016/j.jmapro.2019.10.007
10.1016/j.actamat.2010.10.031

10.1016/j.cja.2016.01.009
10.1016/j.ceramint.2010.11.033
10.1016/S0257-8972(99)00305-9
10.1016/j.msea.2016.05.117
10.1016/j.corsci.2009.08.011
10.1016/j.ceramint.2011.12.076
10.1016/0036-9748(89)90452-3
10.1016/j.actamat.2016.05.003
10.1016/1359-6462(96)00079-6
10.1016/j.msea.2019.138087
10.1016/0022-3115(81)90166-5
10.1016/j.wear.2017.01.070
10.1016/S0921-5093(00)02039-6
10.1016/S0026-0657(97)80113-0
10.1016/j.intermet.2019.03.012
10.1016/j.prostr.2017.11.054
10.1016/S0301-4215(00)00140-3
10.1016/0257-8972(86)90086-1
10.1016/j.mtcomm.2020.101018
10.1016/j.matchar.2012.07.018
10.1016/j.matpr.2016.09.017
10.1016/j.surfcoat.2019.02.024
10.1016/j.msea.2017.03.081
10.1016/S0921-5093(01)01758-0
10.1016/j.surfcoat.2009.08.021
10.1016/0010-938x(91)90025-k
10.1016/S1003-6326(11)61288-0
10.1016/j.vacuum.2019.108857
10.1016/j.ijfatigue.2010.02.003
10.1016/j.surfcoat.2013.02.026
10.1016/j.jallcom.2013.10.180
10.1016/j.msea.2011.10.054
10.1016/S0921-5093(00)00839-X
10.1016/j.tsf.2014.02.051
10.1016/j.ijfatigue.2009.01.023
10.1016/j.actamat.2019.06.041
10.1016/j.actamat.2019.07.002
10.1016/j.jeurceramsoc.2019.10.007
10.1016/j.scriptamat.2003.12.029
10.1016/0924-0136(95)01786-0
10.1016/S0257-8972(01)01427-X
10.1016/j.corsci.2015.05.026
10.1016/j.ijplas.2018.07.006
10.1016/S0921-5093(97)00848-4
10.1016/S0921-5093(03)00218-1
10.1016/j.ceramint.2018.11.112
10.1016/S0921-5093(97)00709-0
10.1016/j.msea.2003.12.005
10.1016/s1468-6996(00)00022-x
10.1016/j.actamat.2016.02.067
10.1016/j.matpr.2017.11.368
10.1016/j.ijmecsci.2014.08.007
10.1016/j.cja.2013.07.026
10.1016/j.msea.2017.04.065

10.1016/j.msea.2012.08.088
10.1016/j.msea.2008.05.031
10.1016/j.scriptamat.2003.08.029
10.1016/s1468-6996(02)00039-6
10.1016/j.matdes.2014.02.052
10.1016/j.msea.2019.138886
10.1016/j.matlet.2019.127042
10.1016/j.msea.2015.05.065
10.1016/j.ijfatigue.2017.03.003
10.1016/j.jallcom.2017.12.265
10.1016/j.corsci.2020.108582
10.1016/0142-1123(86)90024-1
10.1016/j.actamat.2004.01.011
10.1016/S0257-8972(03)00871-5
10.1016/j.jct.2008.09.007
10.1016/j.jallcom.2011.04.001
10.1016/j.corsci.2019.108166
10.1016/j.actamat.2009.10.051
10.1016/j.jmst.2020.01.050
10.1016/S1359-6454(02)00138-6
10.1016/j.msea.2012.05.077
10.1016/0956-716x(92)90593-4
10.1016/j.msea.2012.02.005
10.1016/0042-207X(96)00074-7
10.1016/j.ijmachtools.2019.04.004
10.1016/j.msea.2017.05.089
10.1016/j.actamat.2013.04.071
10.1016/j.commatsci.2005.10.010
10.1016/j.surfcoat.2016.10.022
10.1016/j.commatsci.2018.05.056
10.1016/j.actamat.2017.05.072
10.1016/0036-9748(85)90273-x
10.1016/j.jmst.2013.12.019
10.1016/j.actamat.2009.11.037
10.1016/j.fusengdes.2014.08.012
10.1016/j.proeng.2011.04.331
10.1016/S0921-5093(99)00196-3
10.1016/0736-5845(88)90081-6
10.1016/j.vacuum.2016.11.007
10.1016/j.proeng.2014.11.012
10.1016/j.matdes.2018.06.058
10.1016/j.corsci.2010.08.022
10.1016/j.commatsci.2015.01.007
10.1016/0921-5093(89)90539-x
10.1016/j.matchar.2018.02.039
10.1016/j.matchemphys.2016.07.013
10.1016/j.jmapro.2017.12.011
10.1016/j.msea.2005.01.005
10.1016/j.msea.2005.09.067
10.1016/0921-5093(89)90310-9
10.1016/j.jallcom.2014.12.279
10.1016/j.procir.2016.02.344
10.1016/j.mineng.2016.09.009
10.1016/j.ijfatigue.2013.11.020

10.1016/j.jmatprotec.2014.10.019
10.1016/j.matdes.2018.09.006
10.1016/j.msea.2010.08.050
10.1016/j.jksues.2019.01.002
10.1016/j.ijmecsci.2018.04.050
10.1016/j.scriptamat.2019.05.037
10.1016/S1005-0302(10)60142-9
10.1016/j.intermet.2006.01.045
10.1016/j.acme.2018.05.001
10.1016/j.scriptamat.2017.09.005
10.1016/j.optlastec.2018.06.042
10.1016/j.msea.2012.06.013
10.1016/0956-716x(92)90177-g
10.1016/j.triboint.2017.10.005
10.1016/j.jmapro.2018.07.022
10.1016/j.surfcoat.2017.04.073
10.1016/j.actamat.2013.08.048
10.1016/j.surfcoat.2018.03.037
10.1016/0036-9748(85)90201-7
10.1016/0921-5093(89)90787-9
10.1016/j.msea.2012.07.099
10.1016/S0257-8972(02)00843-5
10.1016/j.msea.2013.04.072
10.1016/S0040-6090(00)01198-6
10.1016/j.ijleo.2018.07.004
10.1016/0257-8972(92)90131-s
10.1016/j.jmmm.2004.11.248
10.1016/S0257-8972(99)00343-6
10.1016/S1003-6326(06)60342-7
10.1016/j.engfracmech.2018.03.019
10.1016/j.wear.2017.01.049
10.1016/j.msea.2012.09.001
10.1016/j.actamat.2018.07.020
10.1016/j.msea.2015.05.031
10.1016/j.matlet.2015.03.142
10.1016/j.addma.2019.06.009
10.1016/j.actamat.2016.11.009
10.1016/S0921-5093(98)00795-3
10.1016/j.msea.2017.11.102
10.1016/j.scriptamat.2018.07.042
10.1016/j.jmatprotec.2018.04.004
10.1016/S0921-5093(01)01230-8
10.1016/j.addma.2019.100874
10.1016/j.apsusc.2019.04.070
10.1016/S1359-6462(97)00033-X
10.1016/j.cirp.2007.05.077
10.1016/j.scriptamat.2018.05.051
10.1016/j.msea.2009.04.020
10.1016/j.surfcoat.2009.02.007
10.1016/s1468-6996(02)00043-8
10.1016/j.surfcoat.2008.07.024
10.1016/j.matdes.2019.107912
10.1016/S1359-6454(00)00099-9
10.1016/j.actamat.2018.01.049

10.1016/j.jmapro.2018.03.046
10.1016/j.msea.2019.138042
10.1016/0022-3115(96)80010-9
10.1016/S0263-2241(98)00017-7
10.1016/j.addma.2018.05.030
10.1016/j.scriptamat.2019.07.047
10.1016/j.msea.2016.10.107
10.1016/j.msea.2006.11.101
10.1016/j.surfcoat.2019.05.060
10.1016/j.surfcoat.2014.06.011
10.1016/0026-0800(89)90018-9
10.1016/j.addma.2018.03.031
10.1016/j.jmst.2019.11.017
10.1533/9781845690786.126
10.1016/j.matchar.2018.02.021
10.1016/S1359-6462(01)01130-7
10.1016/s1251-8069(97)83174-0
10.1016/j.intermet.2008.06.011
10.1016/j.ultramic.2014.09.001
10.1016/j.jmatprotec.2018.08.031
10.1016/j.proeng.2014.10.031
10.1016/j.engfailanal.2019.07.053
10.1016/j.surfcoat.2018.01.006
10.1016/j.msea.2011.02.072
10.1016/j.jmrt.2018.11.006
10.1016/j.jmst.2020.01.010
10.1016/j.msea.2017.03.031
10.1016/j.actamat.2016.01.042
10.1016/S0007-8506(07)60158-9
10.1016/j.scriptamat.2015.12.033
10.1016/0257-8972(87)90116-2
10.1016/0040-6090(78)90391-7
10.1016/j.ijsolstr.2013.03.016
10.1016/j.jmatprotec.2007.07.015
10.1016/S1270-9638(02)00003-2
10.1016/j.corsci.2019.108093
10.1016/S1359-6454(96)00178-4
10.1016/s0036-9748(88)80012-7
10.1016/j.jallcom.2016.09.177
10.1016/j.jallcom.2014.08.041
10.1016/j.matdes.2016.08.083
10.1016/j.msea.2017.05.109
10.1016/j.matlet.2018.10.039
10.1016/j.jallcom.2020.154699
10.1016/j.msea.2016.12.036
10.1016/0921-5093(95)10013-X
10.1016/S1003-6326(16)64291-7
10.1016/j.msea.2005.07.066
10.1016/S1359-6462(98)00375-3
10.1016/0036-9748(86)90224-3
10.1016/S1359-6454(97)00084-0
10.1016/j.vacuum.2018.07.047
10.1016/j.matpr.2016.09.016
10.1016/j.engfailanal.2003.05.019

10.1016/j.actamat.2017.06.037
10.1016/j.commatsci.2006.03.026
10.1016/j.addma.2018.02.018
10.1016/0254-0584(83)90047-0
10.1016/0921-5093(92)90421-v
10.1016/j.ceramint.2015.04.047
10.1016/0921-5093(89)90818-6
10.1016/j.matdes.2016.09.075
10.1016/j.ceramint.2019.07.294
10.1016/j.msea.2007.09.061
10.1016/S0966-9795(98)00130-7
10.1016/j.actamat.2011.10.046
10.1016/j.commatsci.2009.08.008
10.1016/j.scriptamat.2016.06.019
10.1016/j.triboint.2019.106155
10.1016/j.matchar.2013.02.006
10.1016/j.corsci.2020.108475
10.1016/j.corsci.2014.04.039
10.1016/j.euromechsol.2018.03.010
10.1016/j.jallcom.2019.01.329
10.1016/j.jallcom.2016.11.208
10.1016/j.matchar.2013.12.012
10.1016/j.surfcoat.2004.12.005
10.1016/j.commatsci.2018.01.021
10.1016/j.msea.2004.10.039
10.1016/j.msea.2019.05.037
10.1016/j.matchar.2011.05.001
10.1016/j.msea.2013.02.002
10.1016/j.jallcom.2013.11.084
10.1016/j.corsci.2011.10.017
10.1016/S0142-1123(97)84395-2
10.1016/0025-5416(87)90374-0
10.1016/j.engfailanal.2015.07.023
10.1016/j.promfg.2018.07.272
10.1016/0001-6160(86)90198-7
10.1016/j.actamat.2016.12.072
10.1016/j.wear.2011.01.064
10.1016/j.ijfatigue.2020.105539
10.1016/j.vacuum.2018.07.005
10.1016/j.msea.2016.11.040
10.1016/S0921-5093(98)00867-3
10.1016/j.ndteint.2015.12.008
10.1016/j.surfcoat.2010.07.064
10.1016/j.jallcom.2016.12.109
10.1016/j.msea.2010.09.065
10.1016/S0026-0657(01)80725-6
10.1016/0001-6160(88)90003-x
10.1016/j.scriptamat.2006.05.013
10.1016/j.ijsolstr.2017.08.021
10.1016/j.scriptamat.2013.11.001
10.1016/S1359-6462(98)00380-7
10.1016/j.surfcoat.2008.08.054
10.1016/S1003-6326(18)64919-2
10.1016/j.msea.2017.09.066

10.1016/j.corsci.2016.09.007
10.1016/j.msea.2007.07.020
10.1016/S1359-6462(97)00341-2
10.1016/j.jmapro.2019.10.030
10.1016/j.intermet.2013.11.022
10.1016/j.supflu.2015.10.020
10.1016/j.fluid.2017.10.006
10.1016/j.jallcom.2004.08.031
10.1016/j.jmst.2019.05.025
10.1016/j.surfcoat.2006.09.076
10.1016/0257-8972(87)90114-9
10.1016/j.matchar.2017.05.022
10.1016/j.msea.2018.02.017
10.1016/j.matchar.2018.05.020
10.1016/j.ndteint.2020.102279
10.1016/j.msec.2016.10.072
10.1016/j.ijleo.2019.163735
10.1016/j.corsci.2016.01.005
10.1016/S1359-6462(97)00337-0
10.1016/j.msea.2007.03.100
10.1016/j.carbon.2016.06.014
10.1016/j.msea.2004.05.048
10.1016/j.ijfatigue.2017.03.021
10.1016/0026-0800(87)90063-2
10.1016/j.surfcoat.2016.08.059
10.1016/j.matchar.2017.09.029
10.1016/0025-5416(85)90282-4
10.1016/j.surfcoat.2016.10.076
10.1016/0257-8972(94)02289-3
10.1016/S1359-6462(00)00498-X
10.1016/j.corsci.2014.01.020
10.1016/j.surfcoat.2014.07.030
10.1016/j.msea.2015.09.022
10.1016/0022-3115(84)90688-3
10.1016/S1566-1369(02)80090-5
10.1016/S0257-8972(01)01348-2
10.1016/0025-5416(86)90101-1
10.1016/S0167-577X(02)00689-4
10.1016/j.ijplas.2017.06.007
10.1016/j.matdes.2016.12.074
10.1016/j.surfcoat.2015.03.057
10.1016/j.msea.2007.05.061
10.1016/j.msea.2004.01.109
10.1016/j.actamat.2012.01.039
10.1016/j.jcrysgro.2018.07.036
10.1016/j.surfcoat.2011.12.019
10.1016/j.matchemphys.2007.03.017
10.1016/S0921-5093(97)00847-2
10.1016/j.ijpvp.2011.06.010
10.1016/s1005-8850(08)60278-9
10.1016/j.jallcom.2016.05.231
10.1016/S0956-716X(99)80074-0
10.1016/0921-5093(91)90272-o
10.1016/j.matdes.2010.04.019

10.1016/j.microrel.2008.01.008
10.1016/j.tafmec.2011.11.006
10.1016/j.msea.2008.01.023
10.1016/j.scriptamat.2015.07.030
10.1016/S1003-6326(13)62423-1
10.1016/j.actamat.2014.11.016
10.1016/j.matdes.2013.01.055
10.1016/j.intermet.2006.10.042
10.1016/0013-7944(93)90062-w
10.1016/j.matlet.2006.06.023
10.1016/j.msea.2014.03.063
10.1016/j.msea.2019.138530
10.1016/j.matpr.2018.02.140
10.1016/j.msea.2018.09.034
10.1016/j.euromechsol.2018.07.008
10.1016/S0921-5093(99)00359-7
10.1016/S0921-5093(97)00258-X
10.1016/j.jallcom.2016.08.194
10.1016/j.scriptamat.2017.07.006
10.1016/j.msea.2016.12.095
10.1016/S0924-0136(97)02906-3
10.1016/0001-6160(88)90283-0
10.1016/j.commatsci.2018.06.019
10.1016/0025-5416(87)90538-6
10.1016/j.msea.2019.138044
10.1016/S0921-5093(97)00732-6
10.1016/j.apsusc.2012.04.172
10.1016/j.ijfatigue.2019.05.023
10.1016/S0925-8388(03)00524-3
10.1016/S0924-0136(02)00270-4
10.1016/j.msea.2012.07.012
10.1016/j.acme.2019.04.001
10.1016/0257-8972(87)90122-8
10.1016/j.jallcom.2017.05.233
10.1016/j.corsci.2018.02.022
10.1016/j.electacta.2008.06.076
10.1016/0956-716x(94)90208-9
10.1016/j.ijfatigue.2016.06.009
10.1016/j.ijfatigue.2007.04.003
10.1016/j.surfcoat.2010.08.154
10.1016/j.ijplas.2018.10.003
10.1016/j.jallcom.2017.01.283
10.1016/j.matpr.2018.06.080
10.1016/0001-6160(87)90180-5
10.1016/S0921-5093(03)00478-7
10.1016/j.msea.2020.138918
10.1016/S0921-5093(02)00837-7
10.1016/0956-716x(93)90149-m
10.1016/0956-716x(93)90222-e
10.1016/j.commatsci.2014.04.009
10.1016/S0921-5093(97)00243-8
10.1016/j.scriptamat.2006.03.048
10.1016/B978-0-08-047561-5.00011-7
10.1016/j.jallcom.2017.01.179

10.1016/j.jmatprotec.2004.09.081
10.1016/j.pnsc.2015.01.010
10.1016/S0022-3115(00)00711-X
10.1016/j.actamat.2018.07.038
10.1016/j.actamat.2018.08.014
10.1016/j.ijfatigue.2016.02.018
10.1016/0040-6090(85)90169-5
10.1016/j.matdes.2012.12.061
10.1016/j.engfailanal.2008.07.005
10.1016/j.msea.2007.05.033
10.1016/j.calphad.2016.08.002
10.1016/j.measurement.2006.06.019
10.1016/j.msea.2006.04.091
10.1016/j.actamat.2016.12.012
10.1016/j.ijplas.2017.09.006
10.1016/j.msea.2016.08.030
10.1016/j.tsf.2008.08.124
10.1016/j.mseb.2006.02.019
10.1016/S0921-5093(99)00067-2
10.1016/j.optlastec.2017.05.038
10.1016/S0043-1648(01)00680-9
10.1016/j.commatsci.2016.05.036
10.1016/j.matlet.2018.06.002
10.1016/j.matchar.2017.03.038
10.1016/0142-1123(96)82743-5
10.1016/S0921-5093(99)00454-2
10.1016/j.msea.2008.04.064
10.1016/0956-7151(92)90476-u
10.1016/j.msea.2017.02.015
10.1016/j.surfcoat.2019.06.099
10.1016/j.jmst.2017.04.009
10.1016/j.msea.2016.08.105
10.1016/S0921-5093(01)01788-9
10.1016/0022-3115(84)90065-5
10.1016/S0168-9002(99)00144-8
10.1016/S0924-0136(97)00178-7
10.1016/j.matchar.2008.08.013
10.1016/j.msea.2008.10.030
10.1016/j.surfcoat.2017.02.024
10.1016/j.msea.2020.139085
10.1016/0013-7944(91)90233-q
10.1016/0921-5093(89)90058-0
10.1016/j.trpro.2018.02.027
10.1016/j.msea.2014.03.075
10.1016/S1006-706X(13)60117-X
10.1016/j.tsf.2012.02.006
10.1016/S0921-5093(99)00214-2
10.1016/S1005-0302(11)60036-4
10.1016/S1000-9361(11)60447-0
10.1016/j.surfcoat.2018.02.052
10.1016/j.ijmecsci.2019.06.021
10.1016/j.engfracmech.2019.106493
10.1016/j.jocs.2018.10.005
10.1016/j.msea.2015.12.054

10.1016/S0921-5093(98)00953-8
10.1016/0025-5416(86)90341-1
10.1016/j.ijmecsci.2018.09.044
10.1016/0921-5093(91)90252-i
10.1016/j.surfcoat.2014.08.083
10.1016/j.jallcom.2014.03.170
10.1016/j.matpr.2019.10.102
10.1016/j.msea.2018.05.013
10.1016/S1359-6454(02)00359-2
10.1016/j.scriptamat.2015.10.001
10.1016/j.ijfatigue.2016.05.020
10.1016/S0022-3115(02)00874-7
10.1016/j.msea.2016.08.106
10.1016/0010-938x(85)90011-3
10.1016/0010-938x(92)90146-t
10.1016/j.matpr.2019.11.115
10.1016/j.tws.2016.07.002
10.1016/j.jallcom.2018.09.203
10.1016/j.simpat.2013.09.008
10.1016/j.jallcom.2015.12.157
10.1016/0013-7944(79)90060-2
10.1016/0142-1123(88)90040-0
10.1016/0029-5493(84)90131-6
10.1016/j.energy.2019.115994
10.1016/j.wear.2018.12.054
10.1016/S1006-706X(12)60024-7
10.1016/j.mechmat.2010.07.005
10.1016/j.scriptamat.2017.10.005
10.1016/j.jallcom.2014.02.001
10.1016/S0921-5093(02)00125-9
10.1016/j.ijfatigue.2013.05.014
10.1016/0040-6090(84)90010-5
10.1016/j.matdes.2017.05.014
10.1016/j.actamat.2004.09.007
10.1016/0257-8972(94)90081-7
10.1016/j.jallcom.2013.01.132
10.1016/0013-7944(86)90097-4
10.1016/j.actamat.2013.06.019
10.1016/j.jmatprotec.2019.04.037
10.1016/0956-716x(94)90216-x
10.1016/j.msea.2004.01.108
10.1016/j.procir.2018.05.051
10.1016/j.jnucmat.2009.02.029
10.1016/j.ijfatigue.2013.04.026
10.1016/j.pmatsci.2012.08.002
10.1016/j.scriptamat.2017.10.034
10.1016/j.actamat.2004.01.023
10.1016/j.jmst.2016.08.014
10.1016/j.ijfatigue.2017.04.007
10.1016/0025-5416(87)90264-3
10.1016/j.scriptamat.2004.03.017
10.1016/j.msea.2011.08.031
10.1016/j.msea.2012.08.135
10.1016/j.matlet.2012.11.078

10.1016/S0022-0248(97)00134-6
10.1016/j.msea.2017.01.005
10.1016/S0921-5093(99)00502-X
10.1016/0013-7944(73)90056-8
10.1016/0165-1633(86)90061-4
10.1016/j.ijfatigue.2012.12.005
10.1016/S1359-6454(00)00045-8
10.1016/S0968-4328(00)00083-4
10.1016/j.actamat.2016.07.023
10.1016/0036-9748(84)90329-6
10.1016/S1006-706X(17)30078-X
10.1016/j.ijfatigue.2015.03.015
10.1016/S0924-0136(01)00983-9
10.1016/j.jallcom.2013.05.154
10.1016/j.jallcom.2017.04.276
10.1016/j.corsci.2004.06.008
10.1016/j.msea.2016.01.037
10.1016/S1359-6462(02)00559-6
10.1016/j.ijfatigue.2012.06.010
10.1016/B978-044482548-3/50074-3
10.1016/j.msea.2014.06.105
10.1016/S0042-207X(02)00746-7
10.1016/j.msea.2019.02.088
10.1016/0022-3115(88)90152-3
10.1016/j.matchar.2018.03.039
10.1016/j.ultramic.2017.11.004
10.1016/j.msea.2016.07.067
10.1016/j.pnsc.2017.06.010
10.1016/j.ultramic.2004.11.014
10.1016/j.scriptamat.2017.02.037
10.1016/1350-6307(95)00019-M
10.1016/j.corsci.2019.01.029
10.1016/j.msea.2007.06.010
10.1016/j.msea.2017.08.034
10.1016/j.jallcom.2016.03.077
10.1016/j.jmapro.2018.12.023
10.1016/j.jmatprotec.2018.04.015
10.1016/S1003-6326(12)61688-4
10.1016/j.jallcom.2013.12.262
10.1016/j.matchar.2017.01.021
10.1016/S1359-6454(98)00043-3
10.1016/s1468-6996(01)00047-x
10.1016/j.intermet.2004.07.037
10.1016/0921-5093(89)90737-5
10.1016/j.msea.2014.03.093
10.1016/j.cirpj.2010.11.003
10.1016/S1359-6462(98)00437-0
10.1016/j.corsci.2015.07.033
10.1016/j.msea.2016.12.029
10.1016/j.corsci.2013.04.022
10.1016/0036-9748(86)90242-5
10.1016/j.scriptamat.2011.10.007
10.1016/j.calphad.2018.12.014
10.1016/j.msea.2004.11.013

10.1016/S0263-4368(98)00026-2
10.1016/S0921-5093(01)01349-1
10.1016/j.procir.2017.03.330
10.1016/S0963-8695(00)00074-8
10.1016/j.jmst.2020.03.023
10.1016/j.wear.2011.01.043
10.1016/S0921-5093(98)00766-7
10.1016/j.actamat.2017.07.002
10.1016/j.jallcom.2019.01.263
10.1016/j.jallcom.2016.07.159
10.1016/j.addma.2018.06.014
10.1016/j.jmst.2019.04.015
10.1016/S0167-577X(00)00279-2
10.1016/j.jcrysgro.2017.02.008
10.1016/0001-6160(84)90154-8
10.1016/j.wavemoti.2007.09.008
10.1016/j.msea.2007.07.052
10.1016/j.pnsc.2016.12.001
10.1016/j.ijsolstr.2016.02.031
10.1016/j.ceramint.2006.09.016
10.1016/j.measurement.2010.03.012
10.1016/j.matlet.2011.11.066
10.1016/0956-716x(91)90315-r
10.1016/S1359-6462(00)00512-1
10.1016/S0925-8388(01)01842-4
10.1016/j.jallcom.2017.07.067
10.1016/j.actamat.2012.09.077
10.1016/j.msea.2013.05.028
10.1016/S1359-6454(03)00224-6
10.1016/j.jallcom.2007.07.108
10.1016/S1003-6326(11)61011-X
10.1016/j.jnucmat.2017.10.062
10.1016/j.jallcom.2008.08.058
10.1016/j.jmst.2013.06.002
10.1016/j.wear.2008.11.004
10.1016/j.optlaseng.2018.07.010
10.1016/j.matpr.2018.03.029
10.1016/j.matdes.2016.08.041
10.1016/j.corsci.2019.108347
10.1016/j.msea.2014.06.015
10.1016/j.wear.2019.05.037
10.1016/j.ijsolstr.2016.03.011
10.1016/S1359-6462(03)00045-9
10.1016/j.jmst.2017.11.027
10.1016/j.actamat.2016.10.048
10.1016/j.matchemphys.2012.11.004
10.1016/0036-9748(83)90287-9
10.1016/S1359-6462(00)00670-9
10.1016/j.intermet.2020.106772
10.1016/j.msea.2013.09.025
10.1016/S0966-9795(00)00040-6
10.1016/j.jmst.2018.11.015
10.1016/0022-3115(92)90441-m
10.1016/S1359-6454(96)00258-3

10.1016/j.ijplas.2020.102659
10.1016/j.jallcom.2019.05.348
10.1016/j.jallcom.2015.12.185
10.1016/j.surfcoat.2017.06.054
10.1016/j.scriptamat.2017.08.039
10.1016/0022-3115(85)90057-1
10.1016/j.jmatprotec.2012.09.006
10.1016/0025-5416(87)90084-x
10.1016/0956-7151(93)90389-a
10.1016/j.jallcom.2018.11.386
10.1016/S1359-6462(03)00051-4
10.1016/s0920-2307(89)80006-4
10.1016/j.scriptamat.2019.03.027
10.1016/j.matdes.2014.11.043
10.1016/j.scriptamat.2007.06.003
10.1016/j.corsci.2010.09.049
10.1016/0921-5093(93)90510-l
10.1016/j.jallcom.2018.12.049
10.1016/j.commatsci.2012.08.019
10.1016/j.powtec.2018.05.047
10.1016/j.jcrysgro.2017.09.020
10.1016/j.ceramint.2019.02.115
10.1016/j.jallcom.2007.03.047
10.1016/j.addma.2016.09.001
10.1016/j.matchar.2015.10.027
10.1016/s1468-6996(01)00003-1
10.1016/j.msea.2006.03.038
10.1016/s1001-0521(08)60156-4
10.1016/j.actamat.2016.02.005
10.1016/j.ijplas.2018.04.009
10.1016/j.ijbiomac.2014.08.039
10.1016/j.surfcoat.2014.10.055
10.1016/j.jallcom.2008.03.095
10.1016/j.scriptamat.2006.08.060
10.1016/j.jallcom.2007.04.252
10.1016/j.matchar.2006.07.013
10.1016/0039-9140(74)80218-3
10.1016/j.matchar.2016.03.016
10.1016/j.cja.2015.06.023
10.1016/j.jnucmat.2012.12.015
10.1016/j.ijfatigue.2014.08.011
10.1016/j.scriptamat.2017.01.005
10.1016/S0257-8972(97)00486-6
10.1016/j.matdes.2019.108244
10.1016/j.msea.2019.02.096
10.1016/j.actamat.2003.11.032
10.1016/j.msea.2004.10.005
10.1016/j.scriptamat.2006.02.044
10.1016/j.intermet.2013.09.014
10.1016/0921-5093(95)80008-5
10.1016/S1359-6454(01)00438-4
10.1016/j.msea.2010.12.088
10.1016/j.matlet.2020.127533
10.1016/j.ijpvp.2007.11.010

10.1016/j.msea.2003.09.029
10.1016/S1359-6454(01)00384-6
10.1016/j.corsci.2019.108388
10.1016/j.corsci.2019.108291
10.1016/j.jallcom.2019.152755
10.1016/j.optlaseng.2018.04.016
10.1016/j.ssc.2007.12.015
10.1016/j.surfcoat.2015.07.043
10.1016/S0921-5093(97)00268-2
10.1016/j.ijmecsci.2019.05.009
10.1016/j.matlet.2011.07.031
10.1016/j.jnucmat.2018.11.040
10.1016/j.msea.2018.03.087
10.1016/j.actamat.2014.10.034
10.1016/j.ijfatigue.2009.02.039
10.1016/j.jmrt.2018.12.006
10.1016/j.ijimpeng.2004.09.007
10.1016/j.scriptamat.2020.03.034
10.1533/9781845694470.3.491
10.1016/j.intermet.2009.05.006
10.1016/S0927-0256(97)00073-6
10.1016/0956-7151(94)90256-9
10.1016/j.corsci.2011.10.040
10.1016/j.msea.2015.06.091
10.1016/j.jmapro.2018.12.025
10.1016/j.matlet.2017.09.053
10.1016/j.matlet.2018.03.106
10.1016/j.msea.2019.138230
10.1016/j.surfcoat.2018.07.069
10.1016/0013-7944(88)90111-7
10.1016/j.ijfatigue.2014.02.013
10.1016/S0254-0584(98)00005-4
10.1016/j.prostr.2019.05.002
10.1016/j.msea.2005.03.082
10.1016/S0257-8972(99)00434-X
10.1016/0022-3115(92)90438-q
10.1016/j.corsci.2018.08.025
10.1016/j.msea.2012.03.092
10.1016/j.matdes.2015.05.055
10.1016/j.apm.2007.11.008
10.1016/j.msea.2008.06.054
10.1016/j.matdes.2019.107599
10.1016/S0043-1648(01)00575-0
10.1016/S0921-5093(01)01587-8
10.1016/S1359-6454(96)00139-5
10.1016/j.matpr.2018.04.149
10.1016/j.jallcom.2003.07.038
10.1016/j.msea.2006.05.094
10.1016/j.msea.2007.03.012
10.1016/j.jallcom.2008.11.121
10.1016/j.matdes.2016.12.024
10.1016/j.matdes.2017.04.020
10.1016/j.ijhydene.2014.08.143
10.1016/0168-7336(90)80015-c

10.1016/j.scriptamat.2018.10.015
10.1016/j.msea.2008.04.097
10.1016/S0927-0256(97)00067-0
10.1016/j.procir.2018.08.239
10.1016/j.powtec.2015.04.073
10.1016/j.msea.2014.10.031
10.1016/j.proeng.2014.06.266
10.1016/0956-716x(90)90501-7
10.1016/j.cja.2019.01.007
10.1016/j.msea.2016.04.081
10.1016/S1359-6462(97)00012-2
10.1016/0040-6090(86)90414-1
10.1016/j.calphad.2004.01.001
10.1016/j.jmps.2019.02.012
10.1016/j.promfg.2019.06.118
10.1016/S1359-6462(97)00208-X
10.1016/S1006-706X(08)60041-2
10.1016/0001-6160(89)90053-9
10.1016/j.actamat.2005.11.016
10.1016/j.msea.2017.11.097
10.1016/0025-5416(78)90197-0
10.1016/S0921-5093(99)00202-6
10.1016/j.micron.2016.12.006
10.1016/j.msea.2006.03.005
10.1016/j.surfcoat.2011.11.015
10.1016/j.corsci.2010.02.022
10.1016/S1359-6454(01)00383-4
10.1016/j.ijfatigue.2016.10.015
10.1016/j.surfcoat.2018.08.024
10.1016/0142-1123(89)90056-x
10.1016/j.msea.2007.11.092
10.1016/j.actamat.2018.03.059
10.1016/S0921-5093(01)01700-2
10.1016/j.ijplas.2009.08.001
10.1016/j.actamat.2017.06.031
10.1016/j.jallcom.2018.09.025
10.1016/j.actamat.2012.04.043
10.1016/j.surfcoat.2015.11.026
10.1016/j.ijfatigue.2014.01.018
10.1016/0921-5107(95)03016-6
10.1016/j.actamat.2016.05.024
10.1016/j.msea.2017.05.075
10.1016/j.jallcom.2015.05.192
10.1016/j.corsci.2006.12.022
10.1016/j.msea.2017.10.092
10.1016/j.mtla.2020.100632
10.1016/S1005-0302(12)60080-2
10.1016/j.matchar.2005.01.001
10.1016/S0167-577X(98)00031-7
10.1016/j.addma.2018.12.001
10.1016/j.corsci.2019.02.013
10.1016/j.jallcom.2019.02.041
10.1016/j.msea.2019.02.054
10.1016/j.msea.2009.06.014

10.1016/j.matdes.2017.04.102
10.1016/0308-0161(95)00091-7
10.1016/j.msea.2018.10.020
10.1016/j.surfcoat.2015.06.038
10.1016/j.jeurceramsoc.2014.02.031
10.1016/S1359-6454(01)00005-2
10.1016/j.surfcoat.2008.08.063
10.1016/0036-9748(82)90428-8
10.1016/j.matchemphys.2012.11.021
10.1016/j.jmst.2016.09.010
10.1016/1359-6462(95)00489-0
10.1016/0013-7944(94)90035-3
10.1016/j.prostr.2019.05.037
10.1016/0308-0161(93)90041-q
10.1016/j.engfailanal.2008.02.004
10.1016/0022-3115(92)90348-o
10.1016/S0921-5093(01)01032-2
10.1016/j.jallcom.2019.05.143
10.1016/j.surfcoat.2011.04.081
10.1016/0025-5416(76)90119-1
10.1016/j.corsci.2014.12.015
10.1016/j.cossms.2014.03.001
10.1016/0025-5416(87)90061-9
10.1016/j.msea.2006.12.129
10.1016/S0257-8972(00)00872-0
10.1016/j.jallcom.2009.07.086
10.1016/j.jnucmat.2019.151742
10.1016/j.vacuum.2013.01.001
10.1016/j.matchar.2018.04.057
10.1016/j.matchar.2016.01.003
10.1016/j.matdes.2014.12.047
10.1016/j.msea.2016.10.048
10.1016/0257-8972(87)90100-9
10.1016/S1003-6326(08)60222-8
10.1016/j.ijfatigue.2010.01.003
10.1016/j.ijhydene.2010.06.071
10.1016/1044-5803(90)90009-9
10.1016/j.scriptamat.2011.06.002
10.1016/0921-5093(95)10053-9
10.1016/j.actamat.2009.01.040
10.1016/j.jallcom.2017.05.130
10.1016/j.apsusc.2016.05.143
10.1016/j.surfcoat.2009.04.031
10.1016/j.apsusc.2014.11.076
10.1016/j.matchar.2017.07.007
10.1016/j.jmatprotec.2008.01.062
10.1016/0022-3115(95)00093-3
10.1016/j.proeng.2017.01.174
10.1016/j.scriptamat.2018.06.037
10.1016/j.jmatprotec.2019.02.014
10.1016/j.physleta.2010.05.065
10.1016/j.precisioneng.2018.05.003
10.1016/0956-716x(92)90340-k
10.1016/j.mechmat.2017.02.003

10.1016/S0921-5093(01)01589-1
10.1016/j.msea.2013.09.013
10.1016/0921-5093(89)90827-7
10.1016/j.surfcoat.2011.03.034
10.1016/S0026-0657(99)80651-1
10.1016/j.matdes.2013.04.034
10.1016/j.msea.2006.04.025
10.1016/j.matchar.2018.03.027
10.1016/j.pnsc.2015.01.007
10.1016/j.jallcom.2016.05.331
10.1016/j.jmst.2017.01.029
10.1016/j.optlastec.2009.03.008
10.1016/S0921-5093(99)00083-0
10.1016/0921-5093(94)91075-8
10.1016/j.jnucmat.2019.01.051
10.1016/S0257-8972(99)00470-3
10.1016/j.jallcom.2018.03.072
10.1016/j.scriptamat.2012.06.022
10.1016/j.physb.2014.09.012
10.1016/j.surfcoat.2011.03.028
10.1016/j.matdes.2019.107863
10.1016/j.jeurceramsoc.2016.09.029
10.1016/j.msea.2006.07.042
10.1016/S0927-0256(96)00062-6
10.1016/1359-6462(96)00298-9
10.1016/j.jmapro.2017.11.028
10.1016/j.jmapro.2017.09.024
10.1016/j.msea.2007.10.074
10.1016/j.cirp.2018.03.011
10.1016/j.promfg.2015.09.032
10.1016/j.surfcoat.2019.124940
10.1016/B978-0-444-53770-6.00022-8
10.1016/0308-0161(91)90036-2
10.1016/j.ijmachtools.2011.04.006
10.1016/j.apsusc.2015.10.176
10.1016/j.jallcom.2016.11.316
10.1016/0036-9748(80)90325-7
10.1016/S0022-3115(03)00006-0
10.1016/j.commatsci.2014.10.011
10.1016/j.apsusc.2014.09.129
10.1016/j.ijplas.2014.09.001
10.1533/9780857097552.2.250
10.1016/j.wear.2011.02.022
10.1016/j.jmst.2019.04.018
10.1016/S0968-4328(02)00015-X
10.1016/j.calphad.2018.12.002
10.1016/0025-5416(84)90166-6
10.1016/j.msea.2013.11.031
10.1016/j.msea.2016.03.080
10.1016/j.actamat.2014.04.056
10.1016/j.optlastec.2018.01.042
10.1016/j.corsci.2014.02.015
10.1016/j.corsci.2015.09.020
10.1016/j.jallcom.2013.07.055

10.1016/S0142-1123(01)00039-1
10.1016/j.msea.2019.138158
10.1016/S1359-6454(00)00038-0
10.1016/0956-716X(95)00232-K
10.1016/S0142-1123(97)82541-8
10.1016/S0257-8972(00)00991-9
10.1016/j.ijfatigue.2020.105517
10.1016/j.matchar.2007.10.006
10.1016/j.jmatprotec.2007.04.049
10.1016/j.scriptamat.2013.03.015
10.1016/S0043-1648(99)00192-1
10.1016/j.rinp.2019.01.056
10.1016/j.ijfatigue.2016.08.009
10.1016/j.actamat.2015.01.050
10.1016/j.matdes.2018.04.040
10.1016/j.msea.2010.05.027
10.1016/S0168-583X(02)01685-3
10.1016/j.jallcom.2019.151758
10.1016/0956-7151(92)90004-x
10.1016/j.tsf.2009.03.148
10.1016/j.jallcom.2019.03.080
10.1016/j.jmst.2018.09.014
10.1016/j.ijheatmasstransfer.2016.08.028
10.1016/j.ijfatigue.2007.01.057
10.1016/j.vacuum.2017.08.040
10.1016/j.ijfatigue.2014.02.020
10.1016/0749-6419(88)90024-1
10.1016/j.ijfatigue.2015.09.007
10.1016/0956-7151(91)90038-3
10.1016/j.jallcom.2018.03.366
10.1016/j.ijpvp.2010.03.015
10.1016/j.wear.2005.08.003
10.1016/j.actamat.2019.05.058
10.1016/j.jmapro.2019.11.028
10.1016/j.materresbull.2013.09.017
10.1016/j.corsci.2013.09.010
10.1016/j.surfcoat.2012.01.060
10.1016/0040-6090(92)90874-b
10.1016/0022-3115(88)90177-8
10.1016/j.msea.2003.10.124
10.1016/j.msea.2012.09.017
10.1016/j.jallcom.2019.07.103
10.1016/j.optlastec.2019.105662
10.1016/j.jallcom.2016.04.099
10.1016/j.actamat.2014.04.028
10.1016/j.jmatprotec.2017.09.014
10.1016/S0921-5093(03)00108-4
10.1016/j.ijhydene.2013.11.035
10.1016/j.electacta.2012.02.055
10.1016/j.msea.2015.05.023
10.1016/j.ijmecsci.2018.02.003
10.1016/j.jmatprotec.2006.09.029
10.1016/j.intermet.2011.09.012
10.1016/j.ijfatigue.2019.02.022

10.1016/j.matpr.2019.12.194
10.1016/0140-6701(95)95576-Q
10.1016/j.msea.2019.03.035
10.1016/0921-5093(94)09721-6
10.1016/j.actamat.2013.10.017
10.1016/j.msea.2015.03.043
10.1016/j.calphad.2018.03.008
10.1016/0921-5093(93)90430-m
10.1016/j.proeng.2010.03.226
10.1016/j.msea.2006.02.415
10.1016/j.surfcoat.2007.06.053
10.1016/j.matdes.2016.06.022
10.1016/j.jmatprotec.2015.12.021
10.1016/S0749-6419(97)00066-1
10.1016/j.matdes.2015.09.036
10.1016/0956-716x(94)90435-9
10.1016/0308-0161(91)90101-7
10.1016/j.scriptamat.2005.09.017
10.1016/j.tsf.2005.12.041
10.1016/j.ijfatigue.2013.07.014
10.1016/j.jmst.2016.06.026
10.1016/j.msea.2012.03.041
10.1016/1359-6462(95)00522-6
10.1016/j.matpr.2019.10.128
10.1016/j.wear.2014.04.021
10.1016/j.actamat.2007.09.039
10.1016/S0921-5093(97)00856-3
10.1016/S0041-624X(02)00139-7
10.1016/S1359-6462(98)00387-X
10.1016/j.jallcom.2008.06.038
10.1016/S0924-0136(99)00365-9
10.1016/j.surfcoat.2007.04.092
10.1016/0956-716x(94)90307-7
10.1016/j.matdes.2019.108443
10.1016/j.jallcom.2016.09.230
10.1016/0026-0800(76)90011-2
10.1016/j.optlaseng.2011.07.001
10.1016/j.jallcom.2014.08.246
10.1016/j.matchar.2010.06.003
10.1016/j.actamat.2009.08.022
10.1016/S0257-8972(02)00702-8
10.1016/0025-5416(78)90151-9
10.1016/j.matchar.2019.03.034
10.1016/j.corsci.2011.10.037
10.1016/j.jnucmat.2011.10.050
10.1016/j.addma.2016.05.007
10.1016/S0045-7825(03)00415-8
10.1016/j.msea.2012.05.067
10.1016/j.matchar.2016.07.007
10.1016/j.commatsci.2010.02.028
10.1016/0257-8972(87)90098-3
10.1016/j.actamat.2020.02.049
10.1016/j.apsusc.2011.07.026
10.1016/j.jallcom.2016.02.122

10.1016/j.msea.2010.07.018
10.1016/j.ndteint.2019.05.003
10.1016/j.matchemphys.2013.09.016
10.1016/j.msea.2017.04.109
10.1016/j.engfailanal.2020.104504
10.1016/j.pnsc.2012.10.004
10.1016/j.jallcom.2019.07.002
10.1016/j.ijfatigue.2013.10.011
10.1016/j.matchar.2018.01.025
10.1016/j.matlet.2014.09.128
10.1016/j.jallcom.2020.154618
10.1016/j.promfg.2016.08.105
10.1016/S0257-8972(01)01370-6
10.1016/0921-5093(92)90258-3
10.1016/j.jmst.2019.07.021
10.1016/S1003-6326(11)61361-7
10.1016/S0266-3538(01)00197-X
10.1016/0142-1123(95)99751-U
10.1016/j.jnucmat.2011.02.003
10.1016/j.physb.2004.01.082
10.1016/S0142-1123(02)00056-7
10.1016/j.ijimpeng.2016.11.005
10.1016/0924-4247(93)80055-1
10.1016/j.actamat.2013.09.018
10.1016/S1359-6462(97)00264-9
10.1016/j.jallcom.2017.03.158
10.1016/j.actamat.2017.04.006
10.1016/S0257-8972(03)00790-4
10.1016/j.matdes.2016.03.050
10.1016/S1359-6454(01)00410-4
10.1016/j.ijrmhm.2018.10.001
10.1016/S1359-6454(97)00163-8
10.1016/j.scriptamat.2010.11.025
10.1016/0013-7944(95)00115-8
10.1016/0001-6160(88)90055-7
10.1016/j.surfcoat.2007.08.014
10.1016/j.ceramint.2011.07.054
10.1016/j.jmatprotec.2005.12.007
10.1016/S0257-8972(97)00481-7
10.1016/S0924-0136(02)00237-6
10.1016/j.surfcoat.2019.06.024
10.1016/0921-5093(89)90055-5
10.1016/j.pnsc.2015.09.001
10.1016/j.actamat.2008.02.022
10.1016/j.calphad.2019.101681
10.1016/j.ultramic.2015.06.001
10.1016/j.scriptamat.2014.11.009
10.1016/j.matpr.2018.02.337
10.1016/j.optlastec.2020.106244
10.1016/0036-9748(82)90363-5
10.1016/j.jallcom.2009.01.057
10.1016/0022-0248(93)90845-n
10.1016/j.matdes.2015.10.003
10.1016/j.jallcom.2006.11.214

10.1016/S0257-8972(97)00483-0
10.1016/j.actamat.2018.12.061
10.1016/j.jallcom.2017.06.003
10.1016/S1044-5803(99)00052-2
10.1016/j.bsecv.2016.01.001
10.1016/j.corsci.2015.11.025
10.1016/j.ultramic.2019.112817
10.1016/S1359-6454(98)00328-0
10.1016/S0142-1123(02)00009-9
10.1016/j.msea.2010.08.091
10.1016/j.msea.2016.03.044
10.1016/S0020-7683(99)00330-3
10.1016/S1003-6326(13)62595-9
10.1016/j.surfcoat.2005.03.030
10.1016/j.vacuum.2017.07.025
10.1016/j.engfailanal.2012.07.007
10.1016/0036-9748(81)90132-0
10.1016/S0257-8972(02)00482-6
10.1016/0956-716x(92)90698-e
10.1016/j.msea.2019.138607
10.1016/j.ultramic.2015.03.012
10.1016/j.surfcoat.2012.08.083
10.1016/j.jmatprotec.2005.02.101
10.1016/j.jallcom.2003.10.072
10.1016/j.msea.2017.09.014
10.1016/0036-9748(82)90177-6
10.1016/j.msea.2016.07.068
10.1016/j.surfcoat.2011.01.031
10.1016/0957-4174(91)90041-c
10.1016/j.matdes.2015.09.143
10.1016/j.scriptamat.2017.04.016
10.1016/j.msea.2006.10.181
10.1016/j.actamat.2010.11.042
10.1016/j.corsci.2008.09.006
10.1016/j.msea.2020.139038
10.1016/j.jallcom.2018.03.314
10.1016/0956-7151(95)00113-A
10.1016/S0142-1123(99)00036-5
10.1016/j.matlet.2013.03.023
10.1016/j.jmatprotec.2007.10.051
10.1016/S1359-6454(01)00065-9
10.1016/j.matchar.2019.109802
10.1016/j.jmatprotec.2019.116266
10.1016/j.matlet.2012.01.028
10.1016/j.mtla.2019.100544
10.1016/j.surfcoat.2006.01.029
10.1016/j.prostr.2019.12.034
10.1016/j.matchar.2016.07.025
10.1016/j.msea.2016.09.098
10.1016/j.scriptamat.2016.09.013
10.1016/j.jallcom.2016.07.240
10.1016/j.surfcoat.2016.09.012
10.1016/j.matchar.2013.05.017
10.1016/0025-5416(79)90088-0

10.1016/j.msea.2013.11.016
10.1016/S0921-5093(97)00009-9
10.1016/S1359-6462(03)00369-5
10.1016/j.matlet.2007.07.058
10.1016/j.comptc.2016.10.010
10.1016/S0925-8388(03)00497-3
10.1016/j.surfcoat.2013.04.044
10.1016/j.vacuum.2016.04.033
10.1016/j.scriptamat.2014.01.030
10.1016/j.engfracmech.2020.106933
10.1016/0010-938x(89)90100-5
10.1016/j.msea.2016.08.081
10.1016/0169-4332(88)90045-1
10.1016/0036-9748(82)90479-3
10.1016/S0261-3069(97)87186-5
10.1016/j.ijfatigue.2009.03.013
10.1016/j.jallcom.2014.02.011
10.1016/j.jnucmat.2014.11.093
10.1016/j.vacuum.2018.07.014
10.1016/0257-8972(91)90022-o
10.1016/0036-9748(82)90415-x
10.1016/j.ceramint.2017.11.109
10.1016/j.msea.2014.11.019
10.1016/S0026-0657(98)80227-0
10.1016/j.msea.2008.10.059
10.1016/j.matlet.2009.09.035
10.1016/j.intermet.2010.11.019
10.1016/j.procir.2018.08.097
10.1016/S1003-6326(14)63110-1
10.1016/j.msea.2016.09.077
10.1016/j.ceramint.2017.09.189
10.1016/1359-6454(95)00285-5
10.1016/j.surfcoat.2006.07.245
10.1016/j.msea.2014.03.015
10.1016/0956-716x(94)90255-0
10.1016/j.wear.2019.01.023
10.1016/j.msea.2012.06.041
10.1016/j.jallcom.2010.02.192
10.1016/j.wear.2010.12.035
10.1016/0022-3115(87)90105-x
10.1016/j.msea.2016.11.096
10.1016/j.ijfatigue.2018.04.009
10.1016/j.msea.2014.03.088
10.1016/j.ijpvp.2012.11.002
10.1016/j.msea.2017.09.067
10.1016/j.corsci.2018.12.002
10.1016/j.materresbull.2014.04.049
10.1016/j.matdes.2017.07.028
10.1016/j.intermet.2019.106556
10.1016/j.jcou.2019.07.007
10.1016/j.jmatprotec.2017.12.026
10.1016/j.corsci.2019.108228
10.1016/j.ijplas.2020.102709
10.1016/j.msea.2008.08.013

10.1016/j.msea.2006.05.134
10.1016/j.solener.2018.01.036
10.1016/j.jcrysgro.2016.01.036
10.1016/j.surfcoat.2003.06.021
10.1016/j.corsci.2015.08.011
10.1016/j.msea.2017.06.032
10.1016/0956-716x(92)90233-5
10.1016/S1359-6462(02)00456-6
10.1016/j.jallcom.2018.10.118
10.1016/j.scriptamat.2014.02.011
10.1016/j.matchar.2018.02.008
10.1016/j.electacta.2006.08.070
10.1016/j.intermet.2018.10.006
10.1016/j.msea.2010.10.061
10.1016/j.ceramint.2018.01.036
10.1016/j.mspro.2012.06.038
10.1016/j.msea.2009.04.055
10.1016/j.intermet.2017.05.012
10.1016/j.msea.2015.03.004
10.1016/S0257-8972(97)00264-8
10.1016/S1359-6454(98)00377-2
10.1016/j.ijfatigue.2006.09.002
10.1016/j.msea.2015.09.001
10.1016/j.jallcom.2012.08.024
10.1016/j.phpro.2011.03.014
10.1016/j.matdes.2012.06.003
10.1016/j.actamat.2014.03.064
10.1016/j.ijleo.2016.09.039
10.1016/0025-5408(91)90050-v
10.1016/j.matchar.2020.110285
10.1016/j.matdes.2018.07.007
10.1016/j.ijfatigue.2009.07.003
10.1016/j.surfcoat.2008.04.049
10.1016/j.jmatprotec.2018.11.032
10.1016/j.jnucmat.2015.09.053
10.1016/j.matchar.2019.01.018
10.1016/j.ijfatigue.2018.09.019
10.1016/j.matchemphys.2019.121749
10.1016/j.scriptamat.2016.10.035
10.1016/j.conbuildmat.2017.05.041
10.1016/S1003-6326(19)64964-2
10.1016/j.surfcoat.2018.11.055
10.1016/j.msea.2005.01.012
10.1016/j.actamat.2009.11.038
10.1016/j.msea.2006.06.069
10.1016/0010-938x(80)90009-8
10.1016/j.ijfatigue.2019.105258
10.1016/j.measurement.2019.05.089
10.1016/j.corsci.2019.01.023
10.1016/j.actamat.2007.11.031
10.1016/j.jallcom.2016.02.115
10.1016/j.jallcom.2017.04.037
10.1016/j.actamat.2019.04.031
10.1016/j.msea.2007.09.097

10.1016/j.actamat.2016.03.077
10.1016/j.jallcom.2013.07.163
10.1016/j.jmst.2020.01.026
10.1016/j.matdes.2016.04.079
10.1016/j.mtla.2018.09.018
10.1016/j.sna.2019.111571
10.1016/j.msea.2018.11.144
10.1016/j.jmatprotec.2017.03.032
10.1016/j.dib.2019.104923
10.1016/S0924-0136(00)00700-7
10.1016/j.scriptamat.2011.10.022
10.1016/j.msea.2017.08.058
10.1016/j.engfailanal.2010.11.012
10.1016/j.actamat.2017.09.047
10.1016/j.scriptamat.2004.09.006
10.1016/j.msea.2019.138435
10.1016/j.actamat.2019.10.011
10.1016/0036-9748(86)90431-x
10.1016/j.commatsci.2016.04.001
10.1016/j.ijsolstr.2014.12.012
10.1016/1359-6454(95)00385-1
10.1016/j.msea.2012.12.081
10.1016/S0045-7825(02)00319-5
10.1016/j.matdes.2016.08.055
10.1016/j.actamat.2006.09.026
10.1016/S0040-6031(01)00751-1
10.1016/j.actamat.2018.07.013
10.1016/0013-7944(92)90077-r
10.1016/j.msea.2016.10.095
10.1016/0022-3115(93)90056-5
10.1016/j.msea.2006.07.160
10.1016/0956-7151(93)90358-y
10.1016/j.wear.2020.203208
10.1016/j.msea.2015.12.094
10.1016/j.msea.2006.07.094
10.1016/j.ijfatigue.2015.07.011
10.1016/j.jallcom.2007.01.176
10.1016/s0036-9748(88)80230-8
10.1016/j.jmapro.2018.10.001
10.1016/0921-5093(90)90335-z
10.1016/j.surfcoat.2014.04.046
10.1016/j.msea.2018.07.077
10.1016/S0142-1123(98)91022-2
10.1016/j.actamat.2008.12.015
10.1016/j.msea.2004.01.094
10.1016/0257-8972(95)02536-7
10.1016/j.powtec.2015.09.023
10.1016/j.jnucmat.2015.05.007
10.1016/j.actamat.2008.05.015
10.1016/S1359-6462(00)00453-X
10.1016/j.scriptamat.2012.11.035
10.1016/S0966-9795(01)00134-0
10.1016/S0921-5093(01)01236-9
10.1016/j.jallcom.2013.03.102

10.1016/j.msea.2004.05.062
10.1016/j.ijfatigue.2015.10.027
10.1016/j.ijfatigue.2019.105373
10.1016/j.msea.2014.09.046
10.1016/j.ijfatigue.2017.04.016
10.1016/j.engfailanal.2004.09.010
10.1016/j.jiec.2013.02.039
10.1016/j.engfracmech.2014.02.005
10.1016/j.scriptamat.2010.02.019
10.1016/S1006-706X(13)60072-2
10.1016/S0921-5093(99)00506-7
10.1016/j.jallcom.2019.152157
10.1016/j.ijfatigue.2012.10.018
10.1016/S0041-624X(99)00140-7
10.1016/j.matchar.2018.09.019
10.1016/j.msea.2011.09.075
10.1016/j.jmps.2011.08.001
10.1016/0025-5416(87)90266-7
10.1016/j.corsci.2016.01.024
10.1016/j.msea.2009.09.035
10.1016/0304-3991(95)00144-1
10.1016/0022-3115(88)90337-6
10.1016/j.msea.2005.07.039
10.1016/S1359-6454(02)00395-6
10.1016/j.jallcom.2014.09.112
10.1016/j.optlastec.2012.11.003
10.1016/j.ijfatigue.2019.105303
10.1016/j.jallcom.2019.04.147
10.1016/j.jallcom.2017.11.004
10.1016/j.matchar.2019.109831
10.1016/s0036-9748(88)80309-0
10.1016/j.jallcom.2019.01.214
10.1016/j.msea.2007.06.031
10.1016/j.actamat.2015.05.025
10.1016/0010-938x(89)90110-8
10.1016/j.cja.2013.04.002
10.1016/S0921-5093(02)00742-6
10.1016/0257-8972(93)90260-u
10.1016/S1359-6454(96)00399-0
10.1016/j.msea.2013.01.029
10.1016/0257-8972(92)90144-y
10.1016/j.jallcom.2016.07.031
10.1016/S1359-6454(96)00087-0
10.1016/j.msea.2019.05.080
10.1016/j.ndteint.2018.01.003
10.1016/j.ijpvp.2007.06.012
10.1016/0010-938x(93)90320-g
10.1016/j.surfcoat.2006.07.242
10.1016/j.intermet.2017.12.020
10.1016/j.calphad.2019.04.004
10.1016/j.ijfatigue.2018.07.017
10.1016/j.pnsc.2014.10.008
10.1016/j.engfailanal.2015.11.057
10.1016/j.jallcom.2017.11.188

10.1016/S0749-6419(98)00054-0
10.1016/j.ijfatigue.2016.07.014
10.1016/j.msea.2011.10.039
10.1016/j.intermet.2019.02.017
10.1016/j.msea.2007.06.066
10.1016/j.msea.2004.01.081
10.1016/S1003-6326(07)60004-1
10.1016/j.applthermaleng.2005.01.012
10.1016/j.commatsci.2016.03.038
10.1016/j.msea.2014.01.026
10.1016/0036-9748(82)90422-7
10.1016/j.matdes.2008.05.006
10.1016/j.physleta.2018.03.031
10.1016/j.msea.2014.09.075
10.1016/j.ijfatigue.2017.07.008
10.1016/0029-5493(89)90084-8
10.1016/j.apsusc.2007.03.009
10.1016/j.jallcom.2006.07.002
10.1016/j.matdes.2012.06.053
10.1016/j.jallcom.2019.04.017
10.1016/j.apsusc.2009.04.170
10.1016/j.jmst.2018.11.026
10.1016/j.intermet.2017.07.018
10.1016/j.vacuum.2018.06.068
10.1016/j.msea.2009.06.011
10.1016/0022-5088(78)90168-6
10.1016/S1359-6454(97)00035-9
10.1016/j.optlastec.2015.07.015
10.1016/S1003-6326(11)61363-0
10.1016/S1000-9361(11)60043-5
10.1016/j.jallcom.2015.04.037
10.1533/9780857096500.2.85
10.1016/j.jallcom.2019.151646
10.1016/0036-9748(85)90062-6
10.1016/j.msea.2006.12.036
10.1016/j.cja.2015.12.016
10.1016/j.jallcom.2010.08.086
10.1016/j.ssi.2004.09.045
10.1016/j.msea.2009.10.068
10.1016/j.surfcoat.2018.03.063
10.1016/j.corsci.2008.08.024
10.1016/j.jmapro.2020.04.002
10.1016/j.ijplas.2011.04.001
10.1016/j.matchar.2018.08.018
10.1016/j.ijfatigue.2018.11.018
10.1016/j.scriptamat.2006.04.033
10.1016/j.jallcom.2015.11.129
10.1016/0257-8972(92)90012-y
10.1016/j.jmatprotec.2018.10.039
10.1016/j.sab.2008.02.004
10.1016/j.jmps.2004.11.002
10.1016/0010-938x(93)90324-a
10.1016/j.matdes.2017.02.023
10.1016/j.calphad.2003.12.003

10.1016/j.jmst.2019.09.020
10.1016/j.jallcom.2008.07.204
10.1016/S1359-6454(99)00107-X
10.1016/0924-0136(95)01979-0
10.1016/j.ijpvp.2009.04.002
10.1016/j.msea.2014.11.010
10.1016/j.jmatprotec.2008.08.009
10.1016/j.ijfatigue.2008.03.033
10.1016/0040-6090(80)90504-0
10.1016/S0026-0657(97)87027-0
10.1016/j.msea.2019.138073
10.1016/j.eml.2017.09.007
10.1016/j.engfailanal.2010.09.017
10.1016/j.corsci.2018.08.050
10.1016/j.solener.2014.09.050
10.1016/j.jallcom.2011.05.029
10.1016/j.jallcom.2018.02.133
10.1016/j.matlet.2018.07.112
10.1016/j.ndteint.2018.11.008
10.1016/j.actamat.2014.02.034
10.1016/j.intermet.2014.05.018
10.1016/S1359-6454(98)00206-7
10.1016/j.scriptamat.2006.01.003
10.1016/S0263-4368(98)00057-2
10.1016/j.actamat.2019.08.005
10.1016/j.ijfatigue.2013.11.026
10.1016/j.actamat.2007.05.006
10.1016/S0921-4526(99)01541-0
10.1016/j.surfcoat.2005.08.140
10.1016/j.matchar.2018.03.012
10.1016/S0142-1123(97)00056-X
10.1016/j.wear.2014.02.007
10.1016/0025-5416(82)90167-7
10.1016/0010-938x(90)90162-x
10.1016/j.matlet.2011.02.020
10.1016/j.matdes.2006.03.026
10.1016/j.msea.2012.04.037
10.1016/j.jallcom.2007.11.083
10.1016/j.jallcom.2006.06.073
10.1016/1359-6462(96)00256-4
10.1016/j.jallcom.2016.05.181
10.1016/s1251-8069(97)89078-1
10.1016/j.corsci.2016.06.020
10.1016/j.surfcoat.2006.07.212
10.1016/j.jeurceramsoc.2013.10.013
10.1016/0956-7151(94)00470-3
10.1016/j.measurement.2019.107125
10.1016/S1003-6326(11)60704-8
10.1016/S0921-5093(98)01054-5
10.1016/0956-716x(91)90297-e
10.1016/0956-716x(90)90260-n
10.1016/j.intermet.2007.07.001
10.1016/S1359-6454(00)00172-5
10.1016/j.jmst.2020.01.047

10.1016/S0924-0136(97)00073-3
10.1016/S0308-0161(98)00099-4
10.1016/j.msea.2004.06.003
10.1016/j.msea.2010.12.094
10.1016/0013-7944(83)90053-x
10.1016/0022-3115(83)90336-7
10.1016/0022-3115(85)90269-7
10.1016/j.matdes.2017.12.020
10.1016/j.jallcom.2020.154529
10.1016/j.engfailanal.2013.03.025
10.1016/j.ijfatigue.2019.105247
10.1016/j.optlastec.2019.105984
10.1016/j.jallcom.2016.07.068
10.1016/j.msea.2017.05.003
10.1016/j.msea.2012.04.065
10.1016/j.msea.2007.02.069
10.1016/j.matdes.2016.07.078
10.1016/j.cirp.2018.03.021
10.1016/j.msea.2018.11.057
10.1016/j.matpr.2016.03.024
10.1016/j.actamat.2016.10.065
10.1016/j.matchar.2004.11.003
10.1016/0013-7944(90)90374-p
10.1016/j.intermet.2004.03.009
10.1016/0921-5093(91)90602-j
10.1016/j.matdes.2018.04.034
10.1016/0029-5493(93)90040-g
10.1016/S1359-835X(00)00035-X
10.1016/j.jmapro.2017.12.015
10.1016/j.cossms.2006.07.002
10.1016/S1359-6454(01)00290-7
10.1016/j.matpr.2018.06.296
10.1016/j.matchar.2015.11.015
10.1016/0025-5416(88)90570-8
10.1016/j.msea.2020.139139
10.1016/j.msea.2008.12.032
10.1016/j.surfcoat.2014.11.053
10.1016/j.corsci.2019.108281
10.1016/j.matchemphys.2003.11.025
10.1016/j.surfcoat.2003.09.002
10.1016/j.surfcoat.2016.10.047
10.1016/j.msea.2008.01.043
10.1016/j.matdes.2011.01.018
10.1016/j.commatsci.2015.09.054
10.1016/S1359-6462(97)00537-X
10.1016/j.engfailanal.2020.104434
10.1016/j.jallcom.2020.154182
10.1016/j.jmatprotec.2018.02.033
10.1016/S1359-6462(01)01210-6
10.1016/j.jallcom.2020.153735
10.1016/j.mtcomm.2019.100545
10.1016/j.msea.2014.01.029
10.1016/j.vacuum.2017.05.024
10.1016/j.jallcom.2016.05.173

10.1016/0956-7151(90)90309-5
10.1016/S1003-6326(19)64943-5
10.1016/0956-716x(90)90511-e
10.1016/j.tws.2019.04.004
10.1016/j.surfcoat.2007.06.039
10.1016/0921-5093(93)90264-f
10.1016/S0026-0657(02)80605-1
10.1016/j.jallcom.2015.09.044
10.1016/j.msea.2018.07.078
10.1016/j.corsci.2018.10.019
10.1016/j.msea.2014.12.010
10.1016/j.ijmecsci.2015.05.013
10.1016/j.msea.2020.138967
10.1016/S0010-938X(99)00139-0
10.1016/S1359-6462(98)00446-1
10.1016/j.jallcom.2013.04.042
10.1016/j.ijfatigue.2019.105279
10.1016/S1006-706X(10)60030-1
10.1016/j.jallcom.2017.06.063
10.1016/j.surfcoat.2004.08.007
10.1016/j.ultramic.2012.06.017
10.1016/S1003-6326(11)61007-8
10.1016/j.msea.2016.04.032
10.1016/j.commatsci.2019.01.013
10.1016/S0921-5093(01)00999-6
10.1016/S0925-8388(02)00956-8
10.1016/0010-938x(88)90037-6
10.1016/j.matlet.2015.02.116
10.1016/S0956-716X(95)00548-A
10.1016/j.msea.2007.10.076
10.1016/S0142-1123(02)00055-5
10.1016/j.surfcoat.2016.12.096
10.1016/j.msea.2007.07.050
10.1016/S0013-7944(01)00021-2
10.1016/j.matpr.2017.07.234
10.1016/j.matdes.2017.04.062
10.1016/j.mineng.2018.11.041
10.1016/j.corsci.2016.03.018
10.1016/0010-938x(85)90032-0
10.1016/j.msea.2011.01.060
10.1016/j.surfcoat.2007.06.017
10.1016/0921-5093(93)90601-a
10.1016/0010-938x(90)90146-v
10.1016/j.actamat.2014.05.039
10.1016/j.scriptamat.2019.04.001
10.1016/j.msea.2020.139105
10.1016/j.intermet.2004.04.006
10.1016/j.jmrt.2018.05.023
10.1016/j.msea.2010.05.070
10.1016/j.corsci.2009.01.003
10.1016/j.msea.2007.11.045
10.1016/0036-9748(86)90142-0
10.1016/j.mechmat.2006.01.005
10.1016/j.jnucmat.2014.05.033

10.1016/j.surfcoat.2011.04.017
10.1016/j.actamat.2009.08.019
10.1016/j.surfcoat.2010.04.050
10.1016/j.matpr.2018.12.135
10.1016/0036-9748(87)90345-0
10.1016/j.matchemphys.2010.11.025
10.1016/j.jallcom.2018.06.107
10.1016/0956-716x(92)90221-y
10.1016/j.msea.2016.08.029
10.1016/j.ijfatigue.2014.09.009
10.1016/j.jallcom.2015.09.118
10.1016/j.jallcom.2016.09.052
10.1016/j.msea.2003.10.319
10.1016/j.ijfatigue.2018.06.033
10.1016/j.jallcom.2017.11.166
10.1016/j.jmps.2013.07.003
10.1016/j.intermet.2012.12.001
10.1016/0025-5416(82)90063-5
10.1016/j.scriptamat.2017.03.031
10.1016/0025-5416(87)90068-1
10.1016/j.jmapro.2017.01.012
10.1016/j.mseb.2009.12.022
10.1016/j.matpr.2019.01.012
10.1016/j.jpcs.2019.109166
10.1016/S0257-8972(99)00346-1
10.1016/j.matdes.2012.06.011
10.1016/j.jfluchem.2018.07.007
10.1016/j.ijplas.2006.03.003
10.1016/j.matdes.2014.03.013
10.1016/j.scriptamat.2017.05.036
10.1016/j.ijplas.2018.06.002
10.1016/s1875-5372(09)60007-2
10.1016/j.actamat.2006.06.041
10.1016/j.surfcoat.2010.11.026
10.1016/j.jmatprotec.2007.10.058
10.1016/j.triboint.2016.03.025
10.1016/j.msea.2008.07.069
10.1016/j.actamat.2019.08.034
10.1016/j.msea.2019.03.064
10.1016/j.corsci.2018.01.032
10.1016/j.proeng.2016.01.221
10.1016/j.corsci.2009.03.039
10.1016/S1359-6462(98)00223-1
10.1016/j.vacuum.2016.11.034
10.1016/j.ijfatigue.2019.105394
10.1016/j.jallcom.2017.05.013
10.1016/j.msea.2016.12.046
10.1016/0022-3115(94)90202-x
10.1016/j.mechmat.2006.01.006
10.1016/j.actamat.2018.11.047
10.1016/j.msea.2010.08.089
10.1016/j.mtla.2019.100508
10.1016/j.addma.2018.10.015
10.1016/j.jmps.2018.05.015

10.1016/j.ijplas.2006.07.001
10.1016/0955-2219(90)90019-c
10.1016/0308-0161(92)90017-a
10.1016/S0257-8972(95)02852-8
10.1016/0010-938x(85)90113-1
10.1016/j.msea.2015.11.042
10.1016/0038-1098(90)91027-e
10.1016/j.commatsci.2020.109586
10.1016/j.matchemphys.2015.07.007
10.1016/j.jmst.2018.04.001
10.1016/j.msea.2008.04.122
10.1016/j.ultramic.2007.02.010
10.1016/j.jallcom.2009.08.025
10.1016/j.ijfatigue.2018.05.003
10.1016/S0921-5093(99)00183-5
10.1016/j.ceramint.2015.09.115
10.1016/j.apsusc.2004.03.142
10.1016/j.apsusc.2005.04.038
10.1016/j.jallcom.2016.03.264
10.1016/j.actamat.2018.10.028
10.1016/0925-8388(95)01914-6
10.1016/0921-5093(94)09717-8
10.1016/j.matdes.2012.01.008
10.1016/j.msea.2015.12.102
10.1016/j.jallcom.2018.02.221
10.1016/j.msea.2016.05.032
10.1016/j.matchemphys.2017.07.017
10.1016/j.jmst.2017.03.023
10.1016/S1359-6462(01)01236-2
10.1016/0025-5416(86)90175-8
10.1016/j.surfcoat.2018.07.035
10.1016/j.addma.2019.100919
10.1016/j.actamat.2011.07.030
10.1016/0921-5093(89)90407-3
10.1016/j.vacuum.2018.08.064
10.1016/0026-0657(95)92301-2
10.1016/j.tafmec.2006.11.007
10.1016/0022-3115(92)90327-h
10.1016/S0167-2738(03)00160-7
10.1016/j.matdes.2017.08.059
10.1016/j.apsusc.2017.02.245
10.1016/j.jallcom.2019.07.003
10.1016/j.jnucmat.2011.01.122
10.1016/j.jmatprotec.2006.04.072
10.1016/j.actamat.2008.10.010
10.1016/S0254-0584(01)00527-2
10.1016/j.actamat.2019.12.047
10.1016/j.msea.2005.11.031
10.1016/j.matdes.2017.02.088
10.1016/j.msea.2016.07.041
10.1016/j.engfailanal.2005.12.002
10.1016/S1359-6462(02)00506-7
10.1016/j.matchemphys.2013.09.022
10.1016/S1359-6454(99)00029-4

10.1016/S0167-577X(97)00197-3
10.1016/S0924-0136(01)00700-2
10.1016/j.ijmachtools.2012.08.001
10.1016/j.jallcom.2015.10.071
10.1016/j.ijsolstr.2015.02.023
10.1016/j.matchemphys.2015.08.057
10.1016/j.jallcom.2016.01.222
10.1016/j.matpr.2018.02.212
10.1016/j.apsusc.2009.03.040
10.1016/j.proeng.2011.04.103
10.1016/S1003-6326(11)61206-5
10.1016/S1359-6462(00)00459-0
10.1016/j.actamat.2004.10.004
10.1016/j.msea.2012.06.034
10.1016/j.msea.2011.11.060
10.1016/j.surfcoat.2004.05.008
10.1016/j.engfracmech.2019.106582
10.1016/S0924-4247(97)01683-X
10.1016/0036-9748(83)90338-1
10.1016/j.actamat.2018.05.032
10.1016/0921-5093(90)90007-p
10.1016/s1006-7191(08)60110-6
10.1016/S0921-5093(02)00686-X
10.1016/S0257-8972(97)00579-3
10.1016/S1359-6454(98)00329-2
10.1016/j.msea.2006.03.097
10.1016/S0921-5093(99)00455-4
10.1016/j.surfcoat.2006.08.107
10.1016/j.matdes.2015.08.035
10.1016/j.msea.2004.05.052
10.1016/j.jallcom.2015.03.076
10.1016/S0749-6419(98)00071-0
10.1016/j.corsci.2014.03.034
10.1016/j.ijfatigue.2013.10.006
10.1016/j.actamat.2009.08.013
10.1016/S0042-207X(02)00509-2
10.1016/S1359-6454(97)00005-0
10.1016/S1005-0302(11)60035-2
10.1016/j.surfcoat.2010.04.059
10.1016/j.jallcom.2014.09.139
10.1016/0142-1123(92)90005-w
10.1016/j.optlastec.2010.06.015
10.1016/j.corsci.2018.02.041
10.1016/0956-716x(94)90060-4
10.1016/0001-6160(78)90104-9
10.1016/j.commatsci.2013.02.022
10.1016/j.addma.2018.05.019
10.1016/j.matdes.2016.03.006
10.1016/j.ijrmhm.2012.04.010
10.1016/j.surfcoat.2016.07.072
10.1016/j.matpr.2020.03.137
10.1016/j.jallcom.2008.07.019
10.1016/0261-3069(90)90207-z
10.1016/j.jmps.2017.05.012

10.1016/j.jallcom.2006.01.111
10.1016/j.msea.2016.03.091
10.1016/j.jallcom.2007.05.092
10.1016/j.actamat.2019.09.040
10.1016/j.commatsci.2018.04.032
10.1016/j.jmatprotec.2015.09.046
10.1016/j.jcrysgro.2017.12.040
10.1016/0921-5093(89)90752-1
10.1016/j.compositesa.2006.02.012
10.1016/j.jnucmat.2013.04.023
10.1016/j.jallcom.2017.09.107
10.1016/S0257-8972(98)00614-8
10.1016/j.corsci.2019.06.015
10.1016/j.ijhydene.2015.07.053
10.1016/j.jallcom.2009.02.115
10.1016/j.jallcom.2004.02.052
10.1016/j.corsci.2015.01.004
10.1016/j.jmatprotec.2019.04.033
10.1016/j.msea.2015.07.058
10.1016/j.jallcom.2017.09.042
10.1016/0956-716X(94)00018-D
10.1016/0167-577x(92)90089-3
10.1016/j.csefa.2017.04.002
10.1016/j.surfcoat.2004.07.107
10.1016/j.jnucmat.2013.09.007
10.1016/j.jpowsour.2010.12.104
10.1016/s0036-9748(88)80143-1
10.1016/j.wear.2018.12.064
10.1016/j.scriptamat.2011.05.024
10.1016/0921-5093(89)90802-2
10.1016/j.jallcom.2016.09.258
10.1016/0013-7944(91)90102-7
10.1016/j.msea.2019.138214
10.1016/j.colsurfa.2009.06.027
10.1016/j.surfcoat.2007.07.021
10.1016/j.intermet.2014.04.008
10.1016/0025-5416(87)90388-0
10.1016/j.jallcom.2015.02.010
10.1016/j.jallcom.2019.153324
10.1016/j.jmst.2013.05.009
10.1016/0169-4332(95)00386-X
10.1016/j.msea.2014.09.118
10.1016/j.ijfatigue.2016.01.027
10.1016/j.msea.2004.03.098
10.1016/j.msea.2010.04.005
10.1016/j.actamat.2008.04.056
10.1016/j.ijmecsci.2015.06.020
10.1016/S0022-0248(00)00759-4
10.1016/j.commatsci.2014.03.019
10.1016/S1003-6326(11)61482-9
10.1016/j.actamat.2017.12.045
10.1016/0921-5093(89)90618-7
10.1016/j.msea.2018.10.084
10.1016/j.msea.2010.06.081

10.1016/j.jmatprotec.2017.07.020
10.1016/S1003-6326(11)60853-4
10.1016/S1006-706X(10)60009-X
10.1016/j.corsci.2013.11.052
10.1016/0956-716X(95)00480-J
10.1016/j.jnucmat.2015.11.026
10.1016/j.surfcoat.2019.07.065
10.1016/j.surfcoat.2008.10.031
10.1016/S1005-0302(11)60151-5
10.1016/j.matchemphys.2013.11.014
10.1016/0308-0161(95)00108-5
10.1016/j.scriptamat.2015.06.016
10.1016/j.scriptamat.2017.06.021
10.1016/j.actamat.2020.03.058
10.1016/j.actamat.2011.04.025
10.1016/0040-6090(82)90583-1
10.1016/S0921-5093(98)01046-6
10.1016/j.actamat.2017.06.024
10.1016/j.pnsc.2013.06.009
10.1016/j.msea.2013.01.032
10.1016/j.ijpvp.2014.01.002
10.1016/j.actamat.2014.04.075
10.1016/j.msea.2017.05.052
10.1016/j.scriptamat.2008.06.053
10.1016/j.surfcoat.2010.01.031
10.1016/j.msea.2014.07.093
10.1016/0022-3115(83)90054-5
10.1016/0921-4526(95)00283-F
10.1016/S0921-5093(03)00225-9
10.1016/j.actamat.2016.07.014
10.1016/j.nanoms.2019.11.003
10.1016/j.msea.2016.07.061
10.1016/j.msea.2014.12.112
10.1016/j.corsci.2019.108127
10.1016/j.jmapro.2019.07.016
10.1016/j.jallcom.2017.12.334
10.1016/j.jallcom.2019.05.292
10.1016/j.jmst.2020.02.009
10.1016/j.matchar.2019.110066
10.1016/j.msea.2017.01.093
10.1016/j.matchemphys.2012.03.055
10.1016/j.msea.2012.01.065
10.1016/j.jallcom.2018.10.385
10.1016/j.matpr.2018.04.131
10.1016/0956-716x(95)00550-f
10.1016/j.surfcoat.2018.05.094
10.1016/j.jmst.2016.05.007
10.1016/j.engfailanal.2007.11.009
10.1016/j.matchar.2019.03.036
10.1016/j.actamat.2012.09.002
10.1016/S0921-5093(99)00701-7
10.1016/j.commatsci.2011.12.026
10.1016/j.actamat.2017.06.021
10.1016/j.msea.2019.04.020

10.1016/j.jallcom.2014.01.223
10.1016/j.engfracmech.2009.09.005
10.1016/j.scriptamat.2003.10.016
10.1016/j.corsci.2016.01.008
10.1016/j.msea.2007.07.012
10.1016/j.surfcoat.2016.12.106
10.1016/j.eswa.2012.12.025
10.1016/j.msea.2014.06.080
10.1016/j.msea.2004.03.005
10.1016/S0925-8388(00)00810-0
10.1016/j.pmatsci.2009.03.010
10.1016/j.commatsci.2013.09.066
10.1016/j.msea.2011.09.091
10.1016/j.intermet.2009.12.012
10.1016/j.ijfatigue.2007.01.037
10.1016/S0013-7944(98)00082-4
10.1016/j.matlet.2005.06.050
10.1016/j.ijplas.2008.06.005
10.1016/j.msea.2019.138282
10.1016/S1044-5803(97)80029-0
10.1016/0142-1123(96)82905-7
10.1016/s0378-7753(96)02353-1
10.1016/0013-7944(85)90161-4
10.1016/j.ceramint.2018.03.197
10.1016/j.surfcoat.2011.06.060
10.1016/j.matpr.2017.07.266
10.1016/0025-5416(85)90091-6
10.1016/j.jallcom.2010.07.196
10.1016/j.electacta.2017.05.149
10.1016/j.jallcom.2018.08.168
10.1016/s1001-0521(06)60106-x
10.1016/0921-5093(95)10071-7
10.1016/j.ijfatigue.2016.11.008
10.1016/0956-716X(95)00120-K
10.1016/j.msea.2008.04.067
10.1016/j.scriptamat.2016.09.031
10.1016/j.matdes.2017.08.049
10.1016/j.msea.2004.02.042
10.1016/j.matchar.2018.11.023
10.1016/j.jmps.2014.11.015
10.1016/j.engfailanal.2015.07.041
10.1016/j.msea.2012.02.054
10.1016/j.proeng.2013.03.263
10.1016/j.ijheatmasstransfer.2009.06.042
10.1016/0142-1123(91)90005-j
10.1016/S1359-6454(03)00110-1
10.1016/j.intermet.2011.01.004
10.1016/S0167-577X(02)00951-5
10.1016/j.addma.2018.05.034
10.1016/j.physb.2012.03.021
10.1016/j.surfcoat.2009.02.008
10.1016/j.msea.2013.07.081
10.1016/0921-5093(89)90743-0
10.1016/j.msea.2011.01.072

10.1016/S0921-5093(98)00957-5
10.1016/j.jmatprotec.2010.07.027
10.1016/j.engfracmech.2015.12.030
10.1016/0022-3115(94)90306-9
10.1016/j.jeurceramsoc.2019.11.073
10.1016/0257-8972(93)90179-r
10.1016/j.actamat.2015.01.040
10.1016/j.actamat.2007.10.006
10.1016/S0921-5093(96)10476-7
10.1016/0921-5093(89)90824-1
10.1016/j.msea.2015.12.042
10.1016/j.actamat.2017.02.051
10.1016/S0925-8388(00)00862-8
10.1016/j.actamat.2016.02.025
10.1016/S0921-5093(98)00849-1
10.1016/S0026-0657(01)80713-X
10.1016/j.msea.2019.02.012
10.1016/S0020-7683(03)00183-5
10.1016/j.ijfatigue.2016.06.018
10.1016/j.jallcom.2010.03.070
10.1016/j.msea.2017.08.093
10.1016/j.matchemphys.2019.01.038
10.1016/j.calphad.2008.06.005
10.1016/j.matdes.2017.06.069
10.1016/j.crhy.2018.10.002
10.1016/S0921-5093(01)01882-2
10.1016/0029-5493(91)90289-t
10.1016/S0025-5408(01)00780-2
10.1016/j.msea.2018.06.104
10.1016/j.jmatprotec.2003.09.008
10.1016/S0254-0584(02)00109-8
10.1016/j.apsusc.2012.07.064
10.1016/j.actamat.2010.02.020
10.1016/j.jfluchem.2008.05.008
10.1016/j.prostr.2020.01.113
10.1016/j.precisioneng.2018.10.002
10.1016/j.finel.2016.07.007
10.1016/j.matpr.2019.07.227
10.1016/S1003-6326(18)64678-3
10.1016/j.jpowsour.2003.10.017
10.1016/S0022-3115(02)01088-7
10.1016/S0013-7944(00)00123-5
10.1016/j.ijhydene.2018.08.179
10.1016/j.ijplas.2018.06.005
10.1016/0036-9748(75)90230-6
10.1016/j.apsusc.2014.01.148
10.1016/S0257-8972(02)00783-1
10.1016/j.jnucmat.2008.08.044
10.1016/S0921-5093(99)00258-0
10.1016/j.corsci.2007.05.021
10.1016/j.jallcom.2019.07.316
10.1016/S1359-6454(00)00303-7
10.1016/S0020-7683(98)00321-7
10.1016/0022-3115(93)90263-x

10.1016/j.surfcoat.2006.07.246
10.1016/j.msea.2018.05.078
10.1016/S0022-3115(01)00518-9
10.1016/S1359-6462(97)00531-9
10.1016/j.matchemphys.2008.10.037
10.1016/j.jmatprotec.2019.116398
10.1016/0029-5493(92)90219-1
10.1016/j.simpat.2019.02.006
10.1016/j.msea.2004.03.079
10.1016/j.ijfatigue.2007.01.040
10.1016/j.calphad.2018.02.006
10.1016/0956-716X(95)00388-C
10.1016/j.corsci.2015.05.034
10.1016/j.matdes.2017.05.028
10.1016/j.ijfatigue.2011.05.009
10.1016/S1003-6326(15)63621-4
10.1016/S0257-8972(00)00952-X
10.1016/j.procir.2018.08.095
10.1016/j.matlet.2012.01.104
10.1016/S1359-6462(99)00094-9
10.1016/j.jmatprotec.2003.11.046
10.1016/j.actamat.2015.09.060
10.1016/j.msea.2019.138034
10.1016/j.jmapro.2017.11.021
10.1016/j.corsci.2008.01.033
10.1016/j.jmatprotec.2004.04.199
10.1016/j.matchemphys.2007.11.041
10.1016/j.jallcom.2018.02.295
10.1016/j.jallcom.2017.06.206
10.1016/S0924-0136(96)02834-8
10.1016/0026-0800(86)90008-x
10.1016/j.msea.2018.10.040
10.1016/j.jallcom.2018.05.096
10.1016/S1359-6462(01)01198-8
10.1016/0025-5416(82)90136-7
10.1016/S0010-938X(02)00127-0
10.1016/j.vacuum.2016.06.016
10.1016/0169-4332(94)90280-1
10.1016/j.msea.2007.09.062
10.1016/j.surfcoat.2017.12.003
10.1016/j.matchar.2007.08.023
10.1016/j.matdes.2015.08.114
10.1016/0001-6160(89)90049-7
10.1016/j.jnucmat.2017.10.052
10.1016/j.surfcoat.2012.07.099
10.1016/0921-5093(90)90344-3
10.1016/0956-716X(95)00243-O
10.1016/j.jallcom.2018.02.039
10.1016/S0167-577X(96)00266-2
10.1016/j.jeurceramsoc.2019.01.028
10.1016/j.jmst.2018.04.016
10.1016/j.engfailanal.2012.07.014
10.1016/j.vacuum.2016.12.022
10.1016/j.actamat.2018.04.044

10.1016/j.jallcom.2009.01.100
10.1016/j.ijmachtools.2012.05.005
10.1016/j.matchar.2016.03.009
10.1016/j.ijfatigue.2008.03.021
10.1016/j.intermet.2006.10.008
10.1016/S1002-0071(12)60059-8
10.1016/j.actamat.2008.02.035
10.1016/j.actamat.2017.02.066
10.1016/0257-8972(95)02511-1
10.1016/j.cirp.2010.03.011
10.1016/j.jallcom.2019.152097
10.1016/S0921-5093(01)01957-8
10.1016/j.jcrysgro.2019.04.010
10.1016/j.msea.2019.02.089
10.1016/j.matdes.2016.01.045
10.1016/0036-9748(83)90390-3
10.1016/S0921-5093(98)01127-7
10.1016/j.msea.2008.04.128
10.1016/j.surfcoat.2012.05.005
10.1016/j.jnucmat.2007.08.015
10.1016/0013-7944(92)90004-x
10.1016/j.calphad.2019.03.007
10.1016/j.msea.2010.03.107
10.1016/j.engfracmech.2020.106948
10.1016/j.msea.2013.05.062
10.1016/0956-716x(94)90258-5
10.1016/j.ultramic.2015.04.021
10.1016/0010-938x(91)90082-z
10.1016/1359-6454(95)00274-X
10.1016/j.matchar.2017.09.032
10.1016/S1003-6326(11)60890-X
10.1016/j.surfcoat.2011.05.005
10.1016/0026-0800(84)90007-7
10.1016/S1359-6454(98)00085-8
10.1016/j.jallcom.2007.11.122
10.1016/j.jallcom.2013.09.047
10.1016/j.matdes.2012.02.002
10.1016/j.corsci.2012.08.035
10.1016/j.commatsci.2018.03.016
10.1016/j.ijplas.2018.09.006
10.1016/s0010-938x(78)80027-4
10.1016/j.jallcom.2018.12.070
10.1016/j.intermet.2011.09.003
10.1016/S1003-6326(14)63154-X
10.1016/j.matpr.2017.07.053
10.1016/j.apsusc.2015.02.068
10.1016/j.ijmecsci.2019.105139
10.1016/j.jmatprotec.2013.06.008
10.1016/j.matchar.2005.04.008
10.1016/j.msea.2007.08.002
10.1016/j.apsusc.2013.09.075
10.1016/j.scriptamat.2009.03.011
10.1016/j.corsci.2004.05.008
10.1016/j.jallcom.2019.152714

10.1016/j.jallcom.2015.10.148
10.1016/j.matdes.2016.10.072
10.1016/S0927-0256(97)00062-1
10.1016/j.actamat.2015.06.034
10.1016/j.corsci.2018.02.007
10.1016/0010-938x(84)90015-5
10.1016/0036-9748(80)90001-0
10.1016/j.msea.2017.12.082
10.1016/j.corsci.2014.04.001
10.1016/0001-6160(89)90016-3
10.1016/j.scriptamat.2007.07.033
10.1016/j.matchar.2018.02.007
10.1016/j.commatsci.2011.09.016
10.1016/j.msea.2016.05.114
10.1016/j.actamat.2009.09.037
10.1016/j.jallcom.2019.05.223
10.1016/j.procir.2018.08.200
10.1016/j.matdes.2014.10.086
10.1016/S0921-5093(02)00465-3
10.1016/j.jmatprotec.2013.09.006
10.1016/0022-3115(85)90219-3
10.1016/j.msea.2010.06.073
10.1016/j.jallcom.2014.10.118
10.1016/0168-583X(95)00187-5
10.1016/j.prostr.2020.01.096
10.1016/j.msea.2014.12.113
10.1016/j.apsusc.2010.03.002
10.1016/j.matlet.2019.03.034
10.1016/j.jclepro.2015.07.045
10.1016/S1003-6326(14)63405-1
10.1016/j.msea.2009.04.044
10.1016/j.msea.2008.04.089
10.1016/j.ijplas.2019.04.009
10.1016/j.matchemphys.2008.11.055
10.1016/j.surfcoat.2011.06.042
10.1016/j.jallcom.2014.12.270
10.1016/j.msea.2004.09.069
10.1016/j.jmatprotec.2012.11.024
10.1016/j.msea.2008.04.105
10.1016/j.ijfatigue.2009.02.030
10.1016/j.matchar.2018.05.017
10.1016/j.intermet.2019.106559
10.1016/j.msea.2018.10.095
10.1016/j.matdes.2018.01.013
10.1016/S0257-8972(02)00656-4
10.1016/j.ijfatigue.2011.11.018
10.1016/j.ceramint.2018.01.047
10.1016/j.tafmec.2019.102453
10.1016/j.ijfatigue.2013.09.008
10.1016/j.matchar.2019.03.015
10.1016/S1003-6326(06)60321-X
10.1016/j.matchar.2008.08.012
10.1016/j.jallcom.2013.05.046
10.1016/S0921-5093(00)01471-4

10.1016/0022-3115(89)90224-9
10.1016/j.msea.2006.05.099
10.1016/j.surfcoat.2016.12.069
10.1016/j.jmatprotec.2011.06.019
10.1016/j.matdes.2007.10.002
10.1016/j.msea.2005.06.023
10.1016/j.scriptamat.2007.12.023
10.1016/j.apm.2012.03.028
10.1016/S1003-6326(20)65248-7
10.1016/j.corsci.2019.108359
10.1016/j.jallcom.2014.09.114
10.1016/S1002-0071(12)60089-6
10.1016/j.matchar.2018.03.051
10.1016/j.msea.2006.11.068
10.1016/j.jmapro.2016.09.001
10.1016/j.jmst.2017.12.017
10.1016/0025-5416(83)90134-9
10.1016/j.probengmech.2013.03.004
10.1016/j.jmst.2013.02.007
10.1016/j.msea.2011.01.118
10.1016/j.msea.2011.07.019
10.1016/S1003-6326(14)63380-X
10.1016/j.jallcom.2015.07.213
10.1016/j.molliq.2017.02.106
10.1016/S0026-0657(99)80024-1
10.1016/j.matchar.2005.10.011
10.1016/j.matchar.2016.09.039
10.1016/1044-5803(93)90003-e
10.1016/j.precisioneng.2017.12.005
10.1016/j.msea.2017.08.011
10.1016/j.actamat.2018.07.064
10.1016/0001-6160(84)90223-2
10.1016/S0010-938X(03)00184-7
10.1016/j.jmst.2017.11.031
10.1016/S1359-6454(98)00362-0
10.1016/j.actamat.2018.09.059
10.1016/j.actamat.2005.05.041
10.1016/j.actamat.2019.08.059
10.1016/S1003-6326(14)63066-1
10.1016/j.msea.2017.03.038
10.1016/S0921-5093(98)01083-1
10.1016/j.commatsci.2019.05.043
10.1016/s1359-6454(04)00387-8
10.1016/S0167-577X(03)00530-5
10.1016/j.jallcom.2017.03.029
10.1016/j.actamat.2016.08.035
10.1016/j.proeng.2011.11.092
10.1016/j.matdes.2014.12.020
10.1016/j.jcrysgro.2011.08.011
10.1016/j.msea.2017.03.085
10.1016/j.corsci.2020.108643
10.1016/S0921-5093(01)01624-0
10.1016/j.vacuum.2018.08.047
10.1016/0025-5416(87)90060-7

10.1016/0022-3115(87)90275-3
10.1016/0956-7151(94)00407-9
10.1016/0040-6090(79)90525-x
10.1016/0261-3069(90)90002-2
10.1016/j.surfcoat.2018.09.025
10.1016/j.ijfatigue.2016.11.033
10.1016/j.jallcom.2007.12.023
10.1016/S1359-6462(97)00549-6
10.1016/j.corsci.2018.10.001
10.1016/S1006-706X(11)60012-5
10.1016/j.intermet.2017.02.017
10.1016/S1006-706X(10)60031-3
10.1016/S0924-0136(01)01123-2
10.1016/j.matlet.2015.01.133
10.1016/0025-5416(83)90039-3
10.1016/j.jmst.2020.02.001
10.1016/j.corsci.2018.04.045
10.1016/j.procs.2018.07.118
10.1016/j.vacuum.2019.01.040
10.1016/j.surfcoat.2012.10.047
10.1016/0001-6160(87)90177-5
10.1016/0026-0800(80)90002-6
10.1016/j.jallcom.2019.01.250
10.1016/j.jmst.2019.05.026
10.1016/S0921-5093(00)01725-1
10.1016/j.jallcom.2019.152358
10.1016/j.calphad.2019.101729
10.1016/j.msea.2018.10.123
10.1016/j.msea.2011.12.060
10.1016/j.matdes.2015.08.038
10.1016/j.jallcom.2016.09.190
10.1016/j.calphad.2012.04.001
10.1016/j.ijlmm.2019.10.002
10.1016/S1002-0071(12)60035-5
10.1016/j.scriptamat.2011.10.006
10.1016/j.jnucmat.2018.12.018
10.1016/0924-0136(94)01602-W
10.1016/j.jmps.2005.11.005
10.1016/j.matchar.2015.02.001
10.1016/j.jmst.2019.08.007
10.1016/j.matpr.2017.06.039
10.1016/j.matlet.2018.09.126
10.1016/j.jallcom.2017.02.007
10.1016/j.scriptamat.2017.12.001
10.1016/j.actamat.2011.02.022
10.1016/j.commatsci.2010.05.031
10.1016/0040-6090(80)90506-4
10.1016/S0257-8972(02)00468-1
10.1016/j.ijmachtools.2015.10.001
10.1016/j.msea.2007.11.017
10.1016/S0921-5093(99)00739-X
10.1016/j.actamat.2019.03.013
10.1016/S1003-6326(11)60961-8
10.1016/j.ultramic.2010.11.013

10.1016/j.proeng.2017.10.1136
10.1016/0167-8442(91)90039-m
10.1016/S0020-7683(00)00261-4
10.1016/S0142-1123(96)00026-6
10.1016/j.jeurceramsoc.2013.06.021
10.1016/j.actamat.2010.09.029
10.1016/j.msea.2018.01.096
10.1016/0267-6605(91)90057-m
10.1016/0956-716x(92)90281-i
10.1016/j.msea.2015.09.040
10.1016/j.cja.2017.06.013
10.1016/j.actamat.2016.01.038
10.1016/j.matchar.2016.02.004
10.1016/j.ijplas.2019.09.002
10.1016/j.surfcoat.2019.03.081
10.1016/j.msea.2008.04.046
10.1016/j.triboint.2019.02.049
10.1016/j.corsci.2019.06.030
10.1016/j.actamat.2006.05.034
10.1016/S1005-0302(10)60036-9
10.1016/j.ceramint.2019.06.071
10.1016/S1003-6326(08)60264-2
10.1016/j.ijimpeng.2006.09.065
10.1016/j.engfailanal.2011.06.017
10.1016/j.jallcom.2017.02.065
10.1016/j.msea.2018.11.064
10.1016/j.ijfatigue.2016.11.021
10.1016/S1359-6462(97)00165-6
10.1016/j.matpr.2019.07.736
10.1016/j.scriptamat.2003.10.028
10.1016/j.actamat.2013.01.004
10.1016/j.tafmec.2017.05.011
10.1016/j.scriptamat.2006.04.032
10.1016/j.actamat.2006.11.047
10.1016/0956-716x(92)90378-r
10.1016/j.msea.2014.07.060
10.1016/j.corsci.2016.04.023
10.1016/j.optlaseng.2013.06.021
10.1016/0025-5416(87)90256-4
10.1016/j.measurement.2014.09.012
10.1016/S0925-8388(96)03067-8
10.1016/j.msea.2012.07.068
10.1016/j.corsci.2015.07.008
10.1016/j.scriptamat.2012.01.025
10.1016/j.engfracmech.2003.11.008
10.1016/j.matpr.2018.06.422
10.1016/j.jallcom.2016.03.187
10.1016/j.jallcom.2019.06.153
10.1016/j.triboint.2018.04.013
10.1016/j.matchar.2008.01.021
10.1016/j.msea.2014.01.033
10.1016/j.msea.2004.03.017
10.1016/j.matchar.2012.06.008
10.1016/0921-4526(92)90546-5

10.1016/j.matpr.2018.06.402
10.1016/j.msea.2018.04.037
10.1016/j.msea.2006.12.160
10.1016/j.matdes.2009.08.018
10.1016/j.commatsci.2019.05.009
10.1016/j.msea.2019.138847
10.1016/j.msea.2014.07.031
10.1016/j.msea.2007.11.059
10.1016/j.intermet.2015.06.006
10.1016/S0921-5093(01)01469-1
10.1016/j.actamat.2015.12.008
10.1016/j.surfcoat.2009.07.026
10.1016/j.triboint.2018.09.006
10.1016/S1005-0302(12)60044-9
10.1016/j.matdes.2018.06.010
10.1016/j.jmst.2015.10.016
10.1016/j.jallcom.2019.07.073
10.1016/j.actamat.2014.08.005
10.1016/j.ast.2017.08.011
10.1016/0036-9748(85)90121-8
10.1016/0022-3115(94)90255-0
10.1016/j.msea.2014.12.094
10.1016/j.scriptamat.2004.05.047
10.1016/j.commatsci.2019.109194
10.1016/j.scriptamat.2008.10.021
10.1016/j.intermet.2004.07.031
10.1016/j.matdes.2016.01.022
10.1016/S1000-9361(07)60013-2
10.1016/j.scriptamat.2014.10.015
10.1016/S1003-6326(19)65052-1
10.1016/j.msea.2018.03.066
10.1016/j.precisioneng.2018.03.007
10.1016/j.intermet.2017.05.010
10.1016/0376-4583(81)90137-0
10.1016/j.msea.2014.10.007
10.1016/j.jre.2018.03.002
10.1016/j.matchemphys.2004.10.050
10.1016/0036-9748(85)90021-3
10.1016/j.surfcoat.2005.03.047
10.1016/j.compstruc.2013.01.009
10.1016/j.msea.2017.08.035
10.1016/0026-0657(95)93639-4
10.1016/S0927-0256(99)00056-7
10.1016/j.jmst.2019.02.006
10.1016/j.jallcom.2018.11.417
10.1016/j.scriptamat.2019.01.041
10.1016/j.msea.2014.09.007
10.1016/j.tafmec.2016.07.013
10.1016/j.apsusc.2011.03.010
10.1016/0921-5093(96)10400-7
10.1016/j.jallcom.2005.08.013
10.1016/j.intermet.2005.11.032
10.1016/S0257-8972(99)00341-2
10.1016/S0924-0136(00)00654-3

10.1016/j.msea.2018.05.005
10.1016/j.msea.2018.12.045
10.1016/j.msea.2005.08.181
10.1016/j.apsusc.2011.02.067
10.1016/j.msea.2005.02.034
10.1016/0956-716x(91)90037-2
10.1016/j.intermet.2013.10.022
10.1016/j.electacta.2005.03.034
10.1016/j.surfcoat.2017.05.034
10.1016/j.msea.2017.03.042
10.1016/j.solmat.2016.10.008
10.1016/j.corsci.2016.03.027
10.1016/j.jallcom.2003.10.074
10.1016/j.surfcoat.2010.07.079
10.1016/S1350-6307(00)00035-2
10.1016/j.jmps.2017.04.001
10.1016/j.corsci.2012.05.018
10.1016/j.cja.2018.01.002
10.1016/j.matdes.2019.108122
10.1016/j.msea.2011.05.083
10.1016/j.carbon.2009.10.038
10.1016/j.jmapro.2016.05.001
10.1016/S0167-577X(00)00361-X
10.1016/j.jeurceramsoc.2010.03.015
10.1016/j.scriptamat.2010.12.028
10.1016/0956-716x(93)90452-x
10.1016/j.addma.2019.03.019
10.1016/0022-3115(89)90586-2
10.1016/j.actamat.2017.03.038
10.1016/S0257-8972(02)00155-X
10.1016/S1359-6454(01)00003-9
10.1016/j.jallcom.2018.07.145
10.1016/S1359-6454(00)00119-1
10.1016/s1468-6996(00)00005-x
10.1016/j.ijimpeng.2011.02.006
10.1016/j.jallcom.2011.05.102
10.1016/j.matchar.2019.03.001
10.1016/j.jmatprotec.2012.06.010
10.1016/0921-5093(92)90102-7
10.1016/j.mtcomm.2018.01.009
10.1016/j.vacuum.2020.109379
10.1016/j.jmatprotec.2006.12.035
10.1016/j.cja.2017.07.013
10.1016/0956-7151(92)90470-y
10.1016/0956-716x(91)90300-p
10.1016/j.actamat.2017.02.070
10.1016/j.ssc.2016.08.013
10.1016/j.taap.2014.05.002
10.1016/0921-5093(89)90306-7
10.1016/j.actamat.2013.04.002
10.1016/S0927-0256(96)00075-4
10.1016/j.commatsci.2017.05.009
10.1016/j.msea.2007.01.114
10.1016/0956-7151(94)90396-4

10.1016/j.commatsci.2012.02.003
10.1016/j.surfcoat.2011.08.031
10.1016/j.ceramint.2015.08.064
10.1016/S0257-8972(03)00466-3
10.1016/j.jallcom.2017.02.251
10.1016/j.actamat.2014.06.048
10.1016/0921-5093(93)90680-d
10.1016/j.procir.2019.03.280
10.1016/j.matdes.2012.06.039
10.1016/j.matdes.2017.10.065
10.1016/j.triboint.2019.105986
10.1016/j.matchar.2005.12.013
10.1016/j.jallcom.2015.03.064
10.1016/j.actamat.2012.02.028
10.1016/j.corsci.2010.08.013
10.1016/j.intermet.2008.04.006
10.1016/j.apsusc.2015.06.155
10.1016/j.tafmec.2018.11.005
10.1016/j.jallcom.2011.05.095
10.1016/j.jallcom.2014.02.068
10.1016/j.corsci.2015.07.007
10.1016/j.sna.2016.05.048
10.1016/0040-6090(87)90280-x
10.1016/0142-1123(96)89679-4
10.1016/j.ijfatigue.2017.01.001
10.1016/j.ultramic.2015.04.015
10.1016/0029-5493(92)90258-w
10.1016/j.ijfatigue.2016.10.020
10.1016/j.surfcoat.2012.09.044
10.1016/j.matdes.2016.02.109
10.1016/S0924-0136(97)00214-8
10.1016/S1044-5803(99)00030-3
10.1016/j.ijrmhm.2015.02.012
10.1016/j.ijfatigue.2016.04.013
10.1016/j.jksus.2017.06.005
10.1016/j.optlastec.2016.01.010
10.1016/j.engfailanal.2009.01.010
10.1016/j.msea.2015.04.031
10.1016/j.mseb.2006.03.011
10.1016/j.matchar.2017.09.014
10.1016/j.msea.2005.06.033
10.1016/j.jallcom.2014.06.060
10.1016/0025-5416(88)90258-3
10.1016/j.surfcoat.2015.03.022
10.1016/S0921-5093(98)00764-3
10.1016/j.msea.2014.06.003
10.1016/j.jclepro.2016.09.216
10.1016/j.promfg.2019.12.057
10.1016/j.scriptamat.2020.03.014
10.1016/j.msea.2015.03.121
10.1016/j.msea.2018.12.078
10.1016/j.jmapro.2018.03.025
10.1016/j.calphad.2013.06.008
10.1016/j.jallcom.2016.02.011

10.1016/S1359-6454(00)00078-1
10.1016/j.msea.2006.02.001
10.1016/j.scriptamat.2013.12.013
10.1016/j.msea.2015.06.044
10.1016/0036-9748(89)90467-5
10.1016/j.surfcoat.2005.07.109
10.1016/j.surfcoat.2016.03.019
10.1016/0921-5093(94)90557-6
10.1016/j.scriptamat.2016.04.014
10.1016/j.jallcom.2019.06.136
10.1016/j.scriptamat.2006.02.039
10.1016/0013-7944(79)90029-8
10.1016/S0921-5093(97)00728-4
10.1016/j.matchar.2011.03.012
10.1016/j.vacuum.2018.07.050
10.1016/j.ijmachtools.2006.01.010
10.1016/j.corsci.2010.09.045
10.1016/j.matchar.2009.01.023
10.1016/j.ceramint.2018.01.094
10.1016/j.surfcoat.2011.03.017
10.1016/S1005-0302(11)60033-9
10.1016/j.actamat.2018.12.053
10.1016/0956-7151(92)90045-g
10.1016/j.msea.2006.09.053
10.1016/j.promfg.2019.06.070
10.1016/j.matchar.2011.03.008
10.1016/j.actamat.2015.10.006
10.1016/j.proeng.2011.04.410
10.1016/j.jallcom.2008.08.062
10.1016/j.actamat.2008.04.037
10.1016/j.msea.2005.03.105
10.1016/j.surfcoat.2007.11.013
10.1016/j.matchemphys.2012.03.002
10.1016/S0921-5093(01)01355-7
10.1016/j.scriptamat.2003.11.025
10.1016/0165-1110(90)90029-b
10.1016/j.msea.2017.01.110
10.1016/j.msea.2019.05.113
10.1016/0956-716X(95)00062-Z
10.1016/j.jmps.2008.09.006
10.1016/S0921-5093(97)00335-3
10.1016/j.jnucmat.2011.05.029
10.1016/j.matchar.2006.02.016
10.1016/0025-5416(77)90167-7
10.1016/j.matdes.2009.05.041
10.1016/j.jnucmat.2013.09.055
10.1016/j.msea.2011.02.012
10.1016/j.jallcom.2019.05.230
10.1016/j.engfracmech.2010.02.005
10.1016/S0925-8388(97)00257-0
10.1016/s0151-9107(99)80075-0
10.1016/j.surfcoat.2018.01.002
10.1016/j.msea.2013.10.034
10.1016/S0254-0584(03)00037-3

10.1016/S1006-706X(10)60117-3
10.1016/j.matdes.2015.06.123
10.1016/j.scriptamat.2019.12.023
10.1016/j.jmatprotec.2019.04.027
10.1016/j.proeng.2013.08.272
10.1016/j.scriptamat.2019.11.019
10.1016/j.msea.2014.11.038
10.1016/0921-5093(90)90343-2
10.1016/j.ceramint.2015.02.027
10.1016/j.matchar.2009.08.008
10.1016/j.ijfatigue.2014.06.001
10.1016/j.msea.2017.08.044
10.1016/j.engfailanal.2015.05.018
10.1016/j.jmrt.2020.03.026
10.1016/j.actamat.2017.10.040
10.1016/j.jmst.2015.11.009
10.1016/j.msea.2020.139185
10.1016/1044-5803(92)90019-e
10.1016/j.procir.2017.03.323
10.1016/j.intermet.2010.04.005
10.1016/j.jnucmat.2006.01.011
10.1016/j.apsusc.2013.02.136
10.1016/j.msea.2017.04.077
10.1016/j.jallcom.2016.12.183
10.1016/j.scriptamat.2007.01.034
10.1016/j.msea.2014.07.059
10.1016/j.actamat.2013.01.052
10.1016/S0261-3069(99)00057-6
10.1016/j.matchar.2013.11.003
10.1016/j.msea.2007.09.003
10.1016/j.scriptamat.2007.12.017
10.1016/0025-5416(87)90474-5
10.1016/S0921-5093(99)00713-3
10.1016/S1000-9361(11)60451-2
10.1016/j.jallcom.2017.03.355
10.1016/S0955-2219(96)00086-6
10.1016/j.matchar.2006.03.010
10.1016/S0010-938X(97)00110-8
10.1016/j.est.2017.05.007
10.1016/0036-9748(85)90018-3
10.1016/j.pnsc.2018.06.001
10.1016/S1359-6462(99)00394-2
10.1016/j.apsusc.2009.07.098
10.1016/0010-938x(92)90138-s
10.1016/1359-6462(96)00161-3
10.1016/j.matpr.2018.09.044
10.1016/j.jmatprotec.2006.03.165
10.1016/S1003-6326(15)63611-1
10.1016/j.ijfatigue.2016.10.019
10.1016/j.finel.2013.10.007
10.1016/j.matdes.2017.07.009
10.1016/j.corsci.2014.12.016
10.1016/j.corsci.2008.12.016
10.1016/j.matdes.2013.11.034

10.1016/S1006-706X(10)60160-4
10.1016/0921-5093(89)90420-6
10.1016/j.surfcoat.2004.09.028
10.1016/j.engfracmech.2019.106800
10.1016/j.engfracmech.2018.04.047
10.1016/j.matdes.2015.12.157
10.1016/S1359-6462(99)00112-8
10.1016/j.jallcom.2013.11.024
10.1016/1044-5803(92)90096-z
10.1016/j.ijheatmasstransfer.2018.01.127
10.1016/j.jallcom.2018.07.087
10.1016/j.apsusc.2015.12.198
10.1016/S0921-5093(02)00251-4
10.1016/j.jallcom.2017.12.295
10.1016/S1002-0071(12)60088-4
10.1016/j.msea.2004.01.014
10.1016/j.jmrt.2019.09.027
10.1016/j.msea.2010.06.046
10.1016/j.ijsolstr.2011.03.011
10.1016/j.jeurceramsoc.2016.10.030
10.1016/j.ceramint.2013.07.083
10.1016/j.scriptamat.2008.07.004
10.1016/0925-8388(95)01524-8
10.1016/j.msea.2016.05.034
10.1016/j.surfcoat.2015.03.015
10.1016/0261-3069(93)90042-t
10.1016/j.msea.2019.138327
10.1016/j.scriptamat.2010.01.044
10.1016/j.surfcoat.2014.09.003
10.1016/0956-716x(91)90471-c
10.1016/0142-1123(95)99753-W
10.1016/j.msea.2004.03.075
10.1016/S1359-6462(99)00088-3
10.1016/j.msea.2016.08.101
10.1016/S0921-5093(97)00132-9
10.1016/j.matdes.2014.05.019
10.1016/j.msea.2012.02.020
10.1016/0043-1648(88)90208-6
10.1016/j.msea.2016.06.075
10.1016/j.msea.2004.01.075
10.1016/j.jallcom.2013.03.086
10.1016/j.jallcom.2007.09.083
10.1016/j.actamat.2018.07.002
10.1016/S0167-6636(03)00032-2
10.1016/j.msea.2016.01.058
10.1016/0308-0161(92)90058-n
10.1016/j.energy.2004.07.017
10.1016/0025-5416(87)90252-7
10.1016/j.msea.2015.03.031
10.1016/0921-5093(90)90285-b
10.1016/S0921-5093(00)00975-8
10.1016/0013-7944(89)90143-4
10.1016/j.matdes.2015.11.102
10.1016/j.ceramint.2014.06.030

10.1016/j.matchar.2018.06.029
10.1016/S1003-6326(11)61027-3
10.1016/0001-6160(85)90165-8
10.1016/j.ijfatigue.2009.03.009
10.1016/j.actamat.2013.02.028
10.1016/S0257-8972(00)01169-5
10.1016/S0257-8972(00)00690-3
10.1016/j.solmat.2016.07.029
10.1016/j.msea.2019.03.103
10.1016/j.ijrmhm.2016.02.006
10.1016/j.ijfatigue.2007.01.013
10.1016/j.jallcom.2006.05.019
10.1016/j.vacuum.2013.03.020
10.1016/0921-5093(91)90825-8
10.1016/0036-9748(83)90472-6
10.1016/S0921-5093(97)00432-2
10.1016/S0026-0657(03)80404-6
10.1016/j.msea.2012.07.032
10.1016/j.msea.2013.06.021
10.1016/S0021-9614(02)00361-0
10.1016/j.matchar.2011.12.009
10.1016/j.msea.2007.12.052
10.1016/j.ijmecsci.2019.105090
10.1016/j.corsci.2019.108400
10.1016/j.pnsc.2019.08.006
10.1016/j.matdes.2012.09.011
10.1016/j.msea.2019.01.113
10.1016/j.jallcom.2014.05.037
10.1016/j.msea.2007.05.080
10.1016/S1359-6454(96)00151-6
10.1016/j.matdes.2012.03.036
10.1016/S0921-5093(96)10494-9
10.1016/j.jallcom.2006.11.152
10.1016/S0921-5093(02)00068-0
10.1016/j.surfcoat.2017.06.006
10.1016/j.msea.2019.138484
10.1016/j.msea.2004.01.049
10.1016/j.jallcom.2019.153259
10.1016/j.wear.2017.09.015
10.1016/j.matdes.2014.12.049
10.1016/S0925-8388(02)00804-6
10.1016/j.jallcom.2018.01.159
10.1016/j.mex.2016.03.002
10.1016/S0257-8972(02)00858-7
10.1016/j.engfailanal.2003.05.014
10.1016/S0921-5093(01)00995-9
10.1016/S0196-8904(96)00133-1
10.1016/j.jallcom.2015.04.237
10.1016/j.apsusc.2018.10.237
10.1016/1359-6462(95)00579-X
10.1016/j.jnucmat.2016.06.038
10.1016/j.pnsc.2016.08.001
10.1016/0956-716x(92)90204-r
10.1016/j.ijfatigue.2017.03.001

10.1016/S0921-5093(97)00576-5
10.1016/j.matchar.2019.02.016
10.1016/S1001-0742(11)61067-X
10.1016/0025-5416(79)90157-5
10.1016/0025-5416(80)90117-2
10.1016/j.corsci.2012.12.021
10.1016/0029-5493(92)90164-q
10.1016/S1003-6326(15)63704-9
10.1016/0921-5093(89)90792-2
10.1016/j.scriptamat.2005.10.030
10.1016/0025-5416(84)90177-0
10.1016/j.msea.2013.01.043
10.1016/0921-5093(89)90730-2
10.1016/0001-6160(77)90092-x
10.1016/j.actamat.2016.07.007
10.1016/j.corsci.2011.04.024
10.1016/j.ijfatigue.2004.06.008
10.1016/j.surfcoat.2007.05.086
10.1016/j.msea.2016.09.015
10.1016/j.calphad.2008.09.014
10.1016/S0169-4332(02)00780-8
10.1016/j.jmrt.2019.12.028
10.1016/S0257-8972(99)00333-3
10.1016/j.jallcom.2015.07.017
10.1016/j.scriptamat.2004.10.039
10.1016/j.jmapro.2020.01.050
10.1016/j.ijmecsci.2019.105016
10.1016/j.actbio.2017.01.085
10.1016/j.commatsci.2016.03.017
10.1016/j.jallcom.2018.08.261
10.1016/j.cirp.2016.04.013
10.1016/S0966-9795(96)00078-7
10.1016/j.proeng.2017.10.1067
10.1016/S1005-0302(11)60105-9
10.1016/j.corsci.2015.05.033
10.1016/j.matdes.2015.09.152
10.1016/0966-9795(95)00053-4
10.1016/j.jallcom.2019.152222
10.1016/j.jmrt.2018.02.008
10.1016/j.jcrysgro.2015.06.003
10.1016/S0257-8972(03)00085-9
10.1016/j.ijfatigue.2010.08.016
10.1016/j.matchar.2017.12.035
10.1016/j.msea.2010.11.044
10.1016/j.msea.2016.03.074
10.1016/j.apsusc.2012.10.065
10.1016/j.jmatprotec.2020.116678
10.1016/j.matchar.2012.02.019
10.1016/j.optlaseng.2019.06.006
10.1016/j.commatsci.2018.02.015
10.1016/j.matlet.2020.127321
10.1016/0040-6090(79)90521-2
10.1016/0308-0161(94)90140-6
10.1016/S0026-0657(01)80714-1

10.1016/j.jallcom.2008.06.066
10.1016/j.engfracmech.2015.03.026
10.1016/j.commatsci.2009.03.004
10.1016/j.actamat.2017.05.038
10.1016/j.msea.2009.09.011
10.1016/j.jmst.2020.01.003
10.1016/S1359-6454(03)00381-1
10.1016/j.calphad.2007.12.006
10.1016/j.msea.2003.06.003
10.1016/j.matchemphys.2010.12.025
10.1016/j.surfcoat.2016.04.042
10.1016/S1003-6326(06)60122-2
10.1016/j.jallcom.2019.152827
10.1016/j.msea.2010.09.083
10.1016/j.msea.2012.07.057
10.1016/j.spmi.2009.01.001
10.1016/S0921-5093(02)00753-0
10.1016/j.jallcom.2019.153320
10.1016/S0966-9795(98)00032-6
10.1016/j.msea.2007.05.108
10.1016/j.intermet.2006.08.016
10.1016/j.surfcoat.2017.07.013
10.1016/j.jallcom.2018.07.218
10.1016/S0257-8972(02)00909-X
10.1016/j.wear.2017.07.008
10.1016/j.intermet.2018.05.011
10.1016/0001-6160(85)90018-5
10.1016/j.ijsolstr.2017.07.031
10.1016/S1359-6454(99)00287-6
10.1016/S0890-6955(96)00075-2
10.1016/0142-1123(96)82811-8
10.1016/j.msea.2019.138267
10.1016/j.actamat.2017.02.058
10.1016/0022-3115(92)90416-i
10.1016/1359-6462(95)00681-8
10.1016/j.surfcoat.2015.03.043
10.1016/j.actamat.2013.07.018
10.1016/j.msea.2016.07.123
10.1016/j.corsci.2019.108187
10.1016/j.matchar.2014.06.019
10.1016/j.jallcom.2015.07.095
10.1016/j.pnsc.2018.04.009
10.1016/j.ijplas.2018.05.002
10.1016/j.msea.2017.02.038
10.1016/j.jmapro.2017.10.014
10.1016/S1572-4859(96)80003-9
10.1016/j.jmatprotec.2007.12.116
10.1016/j.procir.2019.04.150
10.1016/j.ijpvp.2014.07.007
10.1016/j.matdes.2014.08.059
10.1016/j.msea.2019.138785
10.1016/j.jallcom.2008.11.062
10.1016/0263-4368(95)94023-R
10.1016/j.proeng.2010.03.150

10.1016/j.pnsc.2017.12.003
10.1016/S0955-2219(00)00162-X
10.1016/j.jallcom.2007.06.033
10.1016/j.actamat.2010.04.018
10.1016/j.ijfatigue.2012.11.008
10.1016/j.triboint.2016.05.027
10.1016/j.matchemphys.2016.06.037
10.1016/j.vacuum.2011.12.024
10.1016/j.mtla.2018.05.010
10.1016/j.addma.2018.12.018
10.1016/0022-3115(96)00352-2
10.1016/j.actamat.2018.03.035
10.1016/0025-5416(87)90398-3
10.1016/0921-5093(95)09794-5
10.1016/j.jallcom.2015.03.075
10.1016/S0921-5093(99)00102-1
10.1016/j.corsci.2019.07.010
10.1016/1044-5803(91)90042-3
10.1016/j.ijsolstr.2017.06.014
10.1016/j.matchar.2008.01.013
10.1016/j.surfcoat.2010.11.054
10.1016/j.scriptamat.2012.01.008
10.1016/j.jmapro.2019.04.029
10.1016/j.actamat.2014.05.033
10.1016/j.commatsci.2018.11.006
10.1016/j.ijfatigue.2013.02.006
10.1016/S1359-6454(01)00095-7
10.1016/S0013-7944(97)00092-1
10.1016/j.msea.2020.139104
10.1016/j.msea.2013.07.066
10.1016/0010-938x(92)90065-b
10.1016/j.matdes.2019.107793
10.1016/0025-5416(85)90427-6
10.1016/j.corsci.2018.07.005
10.1016/j.msea.2012.04.117
10.1016/j.jallcom.2015.09.165
10.1016/j.mtla.2019.100278
10.1016/j.jmapro.2019.08.011
10.1016/j.msea.2019.138894
10.1016/j.actamat.2015.03.048
10.1016/j.ijfatigue.2012.10.013
10.1016/j.scriptamat.2016.05.032
10.1016/S1270-9638(99)00108-X
10.1016/j.intermet.2007.07.014
10.1016/j.jmatprotec.2013.06.021
10.1016/j.commatsci.2012.06.030
10.1016/j.stam.2004.03.004
10.1016/j.csefa.2016.11.002
10.1016/j.surfcoat.2012.08.071
10.1016/j.msea.2014.04.069
10.1016/j.ijrmhm.2016.05.007
10.1016/j.surfcoat.2013.10.022
10.1016/0036-9748(89)90124-5
10.1016/0025-5416(82)90057-x

10.1016/j.surfcoat.2006.07.247
10.1016/0921-5093(96)10270-7
10.1016/j.actamat.2003.11.012
10.1016/j.commatsci.2016.08.023
10.1016/S1359-6454(00)00111-7
10.1016/j.msea.2010.07.039
10.1016/j.ijfatigue.2018.09.001
10.1016/j.corsci.2019.108293
10.1016/j.matdes.2010.07.040
10.1016/S1002-0721(10)60415-2
10.1016/j.ijplas.2016.10.001
10.1016/j.jmatprotec.2006.03.112
10.1016/j.msea.2006.02.277
10.1016/0022-3115(88)90065-7
10.1016/j.msea.2010.06.005
10.1016/0025-5416(77)90189-6
10.1016/j.msea.2017.02.063
10.1016/S1006-706X(15)30068-6
10.1016/j.msea.2015.11.026
10.1016/j.commatsci.2015.03.003
10.1016/S1359-6454(02)00397-X
10.1016/j.mfglet.2019.09.003
10.1016/j.measurement.2007.01.003
10.1016/S1359-6454(98)00123-2
10.1016/j.scriptamat.2009.08.036
10.1016/j.actamat.2018.10.032
10.1016/j.matdes.2018.05.064
10.1016/j.corsci.2017.08.013
10.1016/j.optlastec.2016.12.013
10.1016/S0921-5093(97)00394-8
10.1016/S0921-5093(96)10492-5
10.1016/0168-583X(95)01428-4
10.1016/j.corsci.2016.07.020
10.1016/0025-5416(81)90186-5
10.1016/j.jallcom.2014.05.104
10.1016/j.matlet.2008.07.032
10.1016/j.actamat.2008.10.042
10.1016/j.jmatprotec.2004.04.273
10.1016/j.jmps.2012.04.003
10.1016/0956-7151(94)00383-S
10.1016/j.msea.2014.03.060
10.1016/j.matchar.2015.05.001
10.1016/j.heliyon.2019.e02618
10.1016/S1359-6454(01)00352-4
10.1016/j.colsurfa.2019.123709
10.1016/j.apsusc.2019.02.179
10.1016/j.scriptamat.2016.04.037
10.1016/j.surfcoat.2013.06.101
10.1016/j.corsci.2017.04.004
10.1016/S0925-8388(02)01034-4
10.1016/0029-5493(88)90236-1
10.1016/j.vacuum.2016.07.009
10.1016/j.surfcoat.2018.12.034
10.1016/j.apsusc.2019.144495

10.1016/0956-7151(95)00089-E
10.1016/S0921-5093(02)00669-X
10.1016/j.scriptamat.2019.11.058
10.1016/j.actamat.2005.03.017
10.1016/j.matdes.2014.04.059
10.1016/j.surfcoat.2008.06.122
10.1016/j.actamat.2016.06.055
10.1016/j.matchar.2018.12.023
10.1016/0013-7944(85)90064-5
10.1016/S0924-0136(02)01047-6
10.1016/j.ceramint.2019.07.235
10.1016/S0925-8388(99)00371-0
10.1016/j.ijfatigue.2020.105528
10.1016/j.actamat.2008.11.005
10.1016/S0010-938X(02)00039-2
10.1016/j.surfcoat.2017.01.041
10.1016/j.msea.2012.09.088
10.1016/j.matpr.2018.06.212
10.1016/0010-938x(96)00097-2
10.1016/j.msea.2010.11.088
